# Ecology meets reproductive medicine in HIV prevention: the case for geography-informed approaches for bacterial vaginosis in Africa

**DOI:** 10.3389/frph.2024.1431306

**Published:** 2024-11-27

**Authors:** Jo-Ann S. Passmore, Sinaye Ngcapu, Serah Gitome, Brian R. Kullin, Kirsten Welp, Darren P. Martin, Disebo Potloane, Monalisa T. Manhanzva, Moses M. Obimbo, Katherine Gill, Mellissa Le Fevre, Anna-Ursula Happel, Heather B. Jaspan, Margaret Kasaro, Elizabeth A. Bukusi

**Affiliations:** ^1^ Institute of Infectious Diseases and Molecular Medicine (IDM), University of Cape Town, Cape Town, South Africa; ^2^ National Health Laboratory Service (NHLS), Cape Town, South Africa; ^3^ Centre for the AIDS Program of Research in South Africa (CAPRISA), Durban, South Africa; ^4^ Centre for Microbiology Research, Kenya Medical Research Institute (KEMRI), Nairobi, Kenya; ^5^ Department of Human Anatomy and Medical Physiology, University of Nairobi, Nairobi, Kenya; ^6^ Desmond Tutu HIV Centre, University of Cape Town, Cape Town, South Africa; ^7^ Division of Immunology, Department of Pathology, University of Cape Town, Cape Town, South Africa; ^8^ Seattle Children’s Hospital, Seattle, WA, United States; ^9^ University of North Carolina Global Projects Zambia, Lusaka, Zambia; ^10^ Department of Obstetrics and Gynaecology, University of Zambia School of Medicine, Lusaka, Zambia; ^11^ Department of Obstetrics and Gynecology, University of North Carolina School of Medicine, Chapel Hill, NC, United States; ^12^ Departments of Global Health and Obstetrics and Gynaecology, University of Washington, Seattle, WA, United States

**Keywords:** female, bacterial vaginosis, HIV, inflammation, probiotics, geography, host genetics, Africa

## Abstract

**Purpose of review:**

Women in Africa bear the burden of the HIV epidemic, which has been associated with the high prevalence of bacterial vaginosis (BV) in the region. However, little progress has been made in finding an effective cure for BV. Drawing on advances in microbiome-directed therapies for gastrointestinal disorders, similar live-biotherapeutic based approaches for BV treatment are being evaluated. Here, we summarize current knowledge regarding vaginal microbiota in BV, explore geographical differences in vaginal microbiota, and argue that novel BV therapeutics should be tailored specifically to meet the needs of African women.

**Recent findings:**

Cervicovaginal microbiota dominated by *Lactobacillus crispatus* are optimal, although these are uncommon in African women. Besides socio-behavioural and environmental influences on the vaginal microbiota, host and microbial genetic traits should be considered, particularly those relating to glycogen metabolism. Novel microbiome-directed approaches being developed to treat BV should employ transfers of multiple microbial strains to ensure sustained colonization and BV cure.

**Summary:**

Improving the efficacy and durability of BV treatment with microbiome-directed therapies by appropriately accounting for host and microbial genetic factors, could potentially reduce the risk of HIV infection in African women.

## Introduction

Women living in Africa arguably represent the highest degrees of diversity in genetics, culture, environment, diet and access to resources, yet reproductive health barriers encountered by African women remain a low priority on the global health agenda. Despite wide access to HIV testing and treatment (1), African women continue to bear the burden of the ongoing HIV epidemic (2). While risks of HIV acquisition outcomes are determined by an array of socio-behavioural, economic and biomedical factors, the attributable risk associated with each differ regionally (3). One of these risk factors is bacterial vaginosis (BV), a common dysbiosis of vaginal microorganisms in reproductive-aged women (4).

The microbiome has emerged as a crucial factor in human health, impacting susceptibility to pathogens (5), poor reproductive outcomes, cancer, metabolic diseases, allergies, autism, and obesity (6). Recognising the transformative potential of microbiome-targeted therapeutics, funding agencies in the global north developed strategic plans for investing in microbiome research (7, 8), yielding large volumes of publicly available data pertaining to wealthy industrialised nations (9). Relatively little progress has been made in understanding the relationships between microbial variability and health in other parts of the world. The context and locations of these “missing microbiomes” have major implications for disease management in African populations and the global community (10). Put simply, answering critical questions relating to the roles that geography, diet, socioeconomic status, and antibiotic use play in shaping the reproductive microbiomes of African women requires microbiome data from Africa.

The purpose of this review is to summarize what is known about the vaginal microbiome in relation to BV, to evaluate whether geographical differences exist in the composition of vaginal microbiota and host interactions with components of the microbiota, and to consider the importance of geography in developing novel BV-treatment modalities that address the unmet needs of African women.

## Shifting from simplicity to complexity is bad in the vaginal niche

Resilience in ecosystems frequently correlates with diversity (11). In the reproductive tract, however, low diversity colonization with *Lactobacillus* species (*L. crispatus*, *L. jensenii*, *L. gasseri*, *L. mucosae*, and *L. vaginalis*) is considered optimal, with high diversity being associated with BV (12). Protective mechanisms used by vaginal *Lactobacillus* spp. to prevent colonization by other commensals and pathogens include competitive exclusion, production of lactic acid, bacteriocins, and biosurfactants (13). Lactic acid lowers vaginal pH, and enhances the structural integrity of the mucosal barrier (14). While low pH excludes competitors, *Lactobacillus* spp. are not acidophiles: they are simply less susceptible to acid than other bacterial species in the vagina (15). *Lactobacillus* spp. differ in their abilities to lower pH and inhibit other strains (16–18). Unlike other *Lactobacillus* spp., *L. iners,* is found in both optimal and dysbiotic microbial states, harbors a cytolysin (inerolysin) and does not produce D-lactic acid (12). In this ecosystem, estrogen gives *Lactobacillus* spp*.* an advantage (19).

Ecosystems frequently shift in composition in response to changes in the environment, and should be studied holistically, considering interactions between all the ecosystem's components (20); including those components present at low relative abundance. The finding that a “key” group of species - *Lactobacillus* spp. - dominate most healthy vaginal microbiomes suggests an important functional role in the ecosystem (20). It is commonly assumed that other non-key species are “passengers” that do not significantly alter the dynamics or function of the ecosystem, with the key species “driving” ecosystem processes such as the maintenance of species diversity and/or stability (9). However, Greenbaum et al. (20) argued that “rarer” microbial taxa occurring during optimal vaginal ecological states may influence the dynamics of the vaginal ecosystem, being “seed banks” poised for proliferation and outgrowth once environmental conditions change (menses, pregnancy, menopause).

In the absence of *Lactobacillus* spp. dominance, the vaginal microbiota shift to a high-diversity state, comprising a diverse assortment of strict and facultative anaerobic bacteria, including *Gardnerella* spp., *Prevotella* spp., and *Fannyhessea vaginae* (13, 21). These diverse anaerobes can form complex biofilms, likely comprising *G. vaginalis* as an “anchor species”, synergistically fostering the outgrowth of other BV-associated anaerobes (22–24). Both *Prevotella* and *Gardnerella* species produce sialidases that degrade cervicovaginal mucus, allowing better contact between the vaginal microbiota and the epithelial barrier (25, 26). BV-associated anaerobes produce a complex array of biogenic amines (such as cadaverine, putrescine, and tyramine), which slows the growth of most vaginal *Lactobacillus* spp. and reduces the production of lactic acid by vaginal *Lactobacillus* spp (27). *G. vaginalis* also produces cytotoxic compounds such as vaginolysin, which trigger epithelial immune responses and NF-κB activation (26). *G. vaginalis* may also differ geographically in terms of prevalence, genetic diversity, antimicrobial resistance profiles, and strain distribution (28, 29), that need to be considered when developing treatment protocols for BV.

## Factors influencing the vaginal microbiome and BV risk

Many socio-behavioural and biomedical risk factors have been defined for BV, including menses, menstrual practices, antibiotics, sexual behaviours, contraceptives, hygiene practices, and partner characteristics [reviewed elsewhere (30)]. Vaginal practices are complex and vary regionally, based on the social and cultural norms, sometimes including intravaginal insertion of commercial products, chemicals, and/or natural products (31–34). In some regions, lubricated sex is preferred, while in others dry sex is preferred (35). Some studies have associated having new or multiple sexual partners and frequent condomless intercourse with a higher risk of BV (36, 37). Condomless intercourse and recent exposure to semen have been associated with reduced *Lactobacillus* spp. prevalence, increased *P. bivia* and *G. vaginalis* prevalence, and increased BV recurrence (38–41). Furthermore, condomless sex with an uncircumcised male partner may further exacerbate risk for BV (42). Female vaginal microbiota often resembles her partners’ and uncircumcised males generally have penile microbiota dominated by anaerobes such as *Finegoldia, Prevotella, Dialister*, and *Peptoniphilus* (43). Thus, male circumcision practices in various geographies and cultures may influence risk for BV.

## Is BV the same globally?

While a cervicovaginal microbiota dominated by *L. crispatus* is considered optimal, African women appear to have more diverse bacterial communities, including those dominated by *L. iners* (4, 21, 42, 44, 45). These associations appear to persist when controlling for sociodemographic factors and sexual practices (46, 47), suggesting that host genetics may influence vaginal microbiome composition (48). Studies of gut microbiota have shown that the composition and function of the gut microbiome are heritable and transferable (49–53). Bubier et al. (54) summarized SNPs in >100 host genes associated with bacterial abundance in twins or from GWAS data. Of those affecting *Lactobacillus* spp. abundance, >50% of the SNPs were located on chromosomes 1–3 and 11, with many of the genes located on these chromosomes involved in sugar and/or lipid metabolism (Table 1).

**Table 1:** Chromosomes with highest density of SNPs influencing lactobacilliaceae colonization.

Chromosome	# SNPs	Gene	Product	Process
1	7	FPGT	Fucose-1-phosphate guanylyltransferase	Fructose and mannose metabolism
		GALE	UDP-galactose-4-epimerase	Galactose metabolism
		MAN1A2	Mannosyl-oligosaccharide 1,2-alpha-mannosidase IB	
		SLC2A1	Glucose transporter 1	Glucose transport (blood-brain barrier)
		SPRR1A	Cornifin-A	Squamous differentiation
		SPRR1B	Cornifin-B	Squamous differentiation
2	15	GFPT1	Glutamine-fructose-6-phosphate transaminase 1	Enzyme participating in glutamate and amino sugars metabolism
		GCKR	Glucokinase regulator	Sugar isomerase: regulatory protein that inhibits glucokinase in liver and pancreatic islet cells
		LEPQTL1	Leptin, serum levels of	Regulate long-term energy balance; correlate with the amount of energy reserves (triglycerides) stored in adipose tissue
		COL3A1	Collagen, type III, alpha 1	Provide structural support to the extracellular space of connective tissues
		COL4A3	Collagen, type IV, alpha 3	Major structural component of basement membranes
		COL4A4	Collagen, type IV, alpha 4	Major structural component of basement membranes
		COL5A2	Collagen, type V, alpha 2	Fibrillar collagens
		MGAT5	Mannosyl (alpha-1,6-)-glycoprotein beta-1,6-N-acetyl-glucosaminyltransferase	Involved in the synthesis of protein-bound and lipid-bound oligosaccharides
		HADHA	Part of an enzyme complex called mitochondrial trifunctional protein	Mitochondrial trifunctional protein required to metabolize long-chain fatty acids
		ABCG5/8	ABC transporter proteins (sterolin-1 and -2)	Regulates sterol absorption and excretion
3	14	COL7A1	Collagen, type VII, alpha 1	Functions as an anchoring fibril between the dermal-epidermal junction in basement membrane
		GMPPB	GDP-mannose pyrophosphorylase B	Enzyme catalyzes conversion of mannose-1-phosphate and GTP to GDP-mannose, involved in N-linked oligosaccharides production
		ADIPOQ	Adiponectin	Protein hormone involved in regulating glucose levels and fatty acid breakdown
		GYG1	Glycogenin-1	Involved in the biosynthesis of glycogen
		BTD	Amidohydrolase biotinidase	Propionic acidemia
		SI	Sucrase-isomaltase	Bifunctional glucosidase
		CACT	Carnitine-acylcarnitine translocase	Enzyme responsible for passive transport of carnitine and carnitine-fatty acid complexes and across the inner mitochondrial membrane as part of the carnitine shuttle system
11	10	ACAT1	Acetyl-Coenzyme A acetyltransferase 1 (acetoacetyl Coenzyme A thiolase)	Converts intracellular free cholesterol into cholesteryl esters for storage in lipid droplets
		APOA4	Apolipoprotein A-IV	Major protein of high-density lipoproteins; implicated in regulating lipid absorption and metabolism, food intake, and glucose metabolism
		BGNT1	N-acetyl-lactosaminide beta-1,3-N-acetyl-glucosaminyl transferase	Key enzyme for core-2 O-glycans biosynthesis; belongs to the family of glycosyltransferases; participates in 4 metabolic pathways: keratan sulfate biosynthesis, glycosphingolipid biosynthesis - neo-lactoseries, glycan structures - biosynthesis 1, and glycan structures - biosynthesis 2
		INS	Insulin gene	Produces insulin
		CPT1A	Carnitine palmitoyltransferase 1A	Mitochondrial enzyme responsible for the formation of acyl carnitines by catalyzing transfer of acyl group of a long-chain fatty acyl-CoA from coenzyme A to l-carnitine

Evidence that the vaginal microbiomes of monozygotic twins are more similar to each other than to their mothers or sisters (55, 56), argues for a role of host genetics in determining microbiome structure. However, it is difficult to disentangle the relative contributions of genetic and environmental factors to overall microbiome structure. For example, a meta-analysis that included 2,748 twins concluded that 31% of reproductive traits were heritable (57), while also highlighting the fact that reproductive traits were one of the trait types most influenced by the environment Nevertheless, if *L. crispatus* heritability is indeed influenced in part by host genetics, this may have implications for probiotic effectiveness in improving vaginal health in diverse populations (42).

## Missing African vaginal strain genomic data in global databases

**Figure 1:**
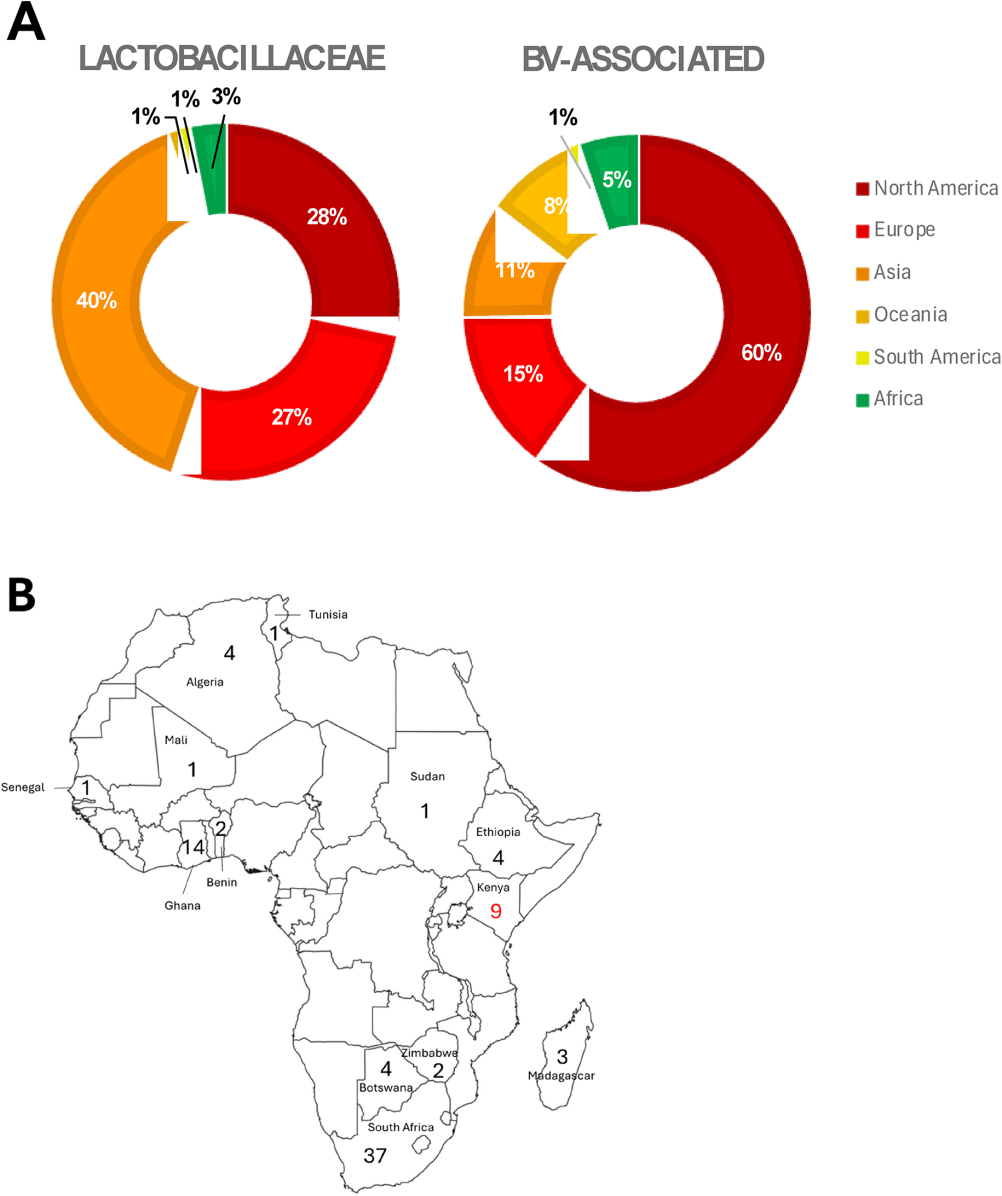
Proportion of publicly available whole genome sequences of *Lactobacillaceae* (*n* = 3084) and BV-associated organisms (BV; *n* = 185), according to **(A)** region: North America (dark red), Europe (bright red), Asia (deep orange), Oceania (light orange), South America (yellow), and Africa (green). BV-associated organisms included *Prevotella* spp., *Gardnerella* spp., and *Fannyhessea* spp. only. **(B)** Within Africa, the numbers of *Lactobacillaceae* genomes (black numbers) and BV-associated organism genomes (red numbers) from different countries are shown.

Recently, Bloom et al. (58) published an associated vaginal genome catalogue, comprising ∼1,200 *Lactobacillus* spp. genomes and metagenome-assembled genomes from >300 women across four continents, including Africa. Despite this useful resource, the NCBI RefSeq assembly database currently contains only 3,084 *Lactobacillaceae* and 185 BV-associated whole genome sequences (we focused just on *Prevotella, Gardnerella* and *Fannyhessea* only for this review; Figure 1; Supplementary Table S1). Of these, most (81% and 91% for *Lactobacillaceae* and BV-associated bacteria, respectively) were from samples collected in the global north and few were derived from vaginal samples.

Better understanding these elusive missing African vaginal genomic data is and will be important moving forward. An example of this is demonstrated in a study by Lithgow et al. (59), where *Lactobacillus*-dominant African women were 3-fold more likely to be colonized by *L. crispatus* isolates lacking the gene involved in glycogen degradation, *pulA*, than European or North America women (60, 61). Glycogen is a key host-provided nutrient that supports vaginal lactobacilli and their fermentative lactic acid production (60). The findings of Lithgow et al. (59) may be critical for our understanding of BV in Africa, suggesting that *pulA* gene loss explains reductions in *L. crispatus* abundance, pullulanase activity and D-lactic acid levels.

## Progress in improving BV treatment

BV is still diagnosed clinically based on symptoms [presence of clue cells [shed epithelial cells coated with BV-associated microbes, evident by Gram stain], vaginal pH >4.5, discharge and a “fishy” odor - known as Amsel criteria (62)] or Nugent scoring (63). However, BV is frequently asymptomatic (64), particularly in Africa (65, 66). Defining the complex ecology of BV using more sensitive molecular approaches (molecular-BV) has proved invaluable in understanding the dysbiosis and identifying new targets for therapy (21).

Symptomatic BV is treated with metronidazole or clindamycin, with treatment focusing on selectively halting the proliferation of BV-associated microorganisms to restore “optimal” vaginal microbes (67). Following treatment, vaginal microbiota shift to *L*. *iners* rather than *L. crispatus* dominance, primarily driven by a massive reduction in BV-associated bacterial abundance (68, 69). BV treatment outcomes following antibiotics appear to be better in women from the US than those living in Africa (70). The vaginal microbiota composition and structure prior to BV treatment is known to influence treatment outcome, such that women with more vaginal bacterial diversity pre-treatment are more likely to experience treatment failure (71). *G. vaginalis* resistant to metronidazole may be a factor underlying different treatment outcomes (72), particularly within biofilms (73, 74), although this has not been systematically compared geographically.

BV recurrence is frequent, with >50% women who clear BV relapsing within six months (75, 76). Some studies have shown that *G. vaginalis* (77) and *F. vaginae* (78) strains that recolonize after initial BV treatment have an increased resistance to subsequent courses of antibiotic treatment. Bannatyne et al. (79) showed that metronidazole susceptibility in *G. vaginalis* strains declined sequentially, with almost all isolates being sensitive after the first course of treatment, and sensitivity reducing by 20%–30% for each subsequent metronidazole treatment (79). In another cross-sectional study, 40% of *P. bivia* isolates, 14% of *P. amnii* and 58% of *P. timonensis* isolates were resistant to clindamycin (80). The extent to which African strains possess antibiotic resistance is yet to be determined.

Several promising novel treatment approaches are currently being investigated for BV treatment. For example, to address post-treatment re-colonization with *L. iners*, Zhu et al. recently showed that oleic acid and other unsaturated long-chain fatty acids, enhance metronidazole-mediated cure rates; by selectively inhibiting *L. iners*, while enhancing *L. crispatus* growth (81). Other combinatorial approaches to enhance metronidazole efficacy have been developed, such as a vaginally inserted ring product that sustainably releases either metronidazole alone (82), or with dapivirine (for HIV prevention) (83). Endolysins, enzymes produced by bacteriophages to degrade bacterial cell walls and disrupt biofilms, are being tested to treat BV, specifically targeting *G. vaginalis* (84). While these approaches are currently in preclinical and *in vitro* study phases, and their efficacies are yet to be tested in humans, they do represent promising avenues for further research to enhance current BV treatment strategies.

## Vaginal microbiome transplants and lessons from the gut

Studies using faecal microbiome transplantation have shown that some donor microbiome-associated phenotypes can be transferred to recipients. Microbiome transplant between obese and lean mice (85), and between lean and obese human donors into mice (86) have demonstrated these phenomena. Faecal microbiome transplant from healthy donors to individuals with autism spectrum disorder (87) and multiple sclerosis (89) have been shown to reduce disease severity, and is now standard-of-care for patients with recurrent *Clostridioides difficile* infections (88). From these studies, it is evident that the clinical benefits of treatment are only sustained if there is successful stable colonization of donor microbiota within the new host (89, 90). Host genetic factors are thought to prevent successful engraftment in recipients who experience only transient colonization, suggesting these ecologically sensitive approaches should factor in a complex set of phenotypes for donor-recipient pairs to ensure successful and sustained colonization (91).

Vaginal microbiome transplantation similarly involves transfer of vaginal fluid from healthy donors with well characterized optimal vaginal microbiota to recipient women with BV (92). The feasibility of transplanting the vaginal microbiome between women to protect against BV has been implied by evidence from women who have sex with women, where both female partners have low risk of BV and relatively stable concordant vaginal microbiota (93). Vaginal microbiome transplantation was first trialled in a small cohort of women with BV in Israel in 2019 (94) with four out of five recipients of the vaginal microbiome transplant showing promising results. In this first-in-human study, donors and recipients shared similar genetic backgrounds, as well as similar socio-behavioural characteristics so the impact of genetic and cultural diversity cannot be extrapolated. Although Mitchell et al. (95) discusses the potential risks of vaginal microbiome transplantation, which necessitate strict safety precautions, a clear benefit is that the “whole” vaginal environment is transferred between donor and recipient, including exact mixtures of vaginal microbes and molecules produced by both hosts and microbes that were associated with health in the donor. This likely assists in the colonization of beneficial bacteria while working against BV-associated bacteria. Understanding the main functional components that need to be transferred to ensure the success of this approach is critical in developing new vaginal microbiome-targeted therapies.

## Simplifying transplantation using probiotics/live-biotherapeutics

Probiotics were defined by the WHO as “live microorganisms that, when administered in adequate amounts, confer a health benefit to the host” while the US FDA introduced the term “live biotherapeutic product”, defined as “a biological product that contains live organisms; is applicable to the prevention, treatment or cure of a disease or condition of humans; and is not a vaccine.” Moving from complex transplantation of entire vaginal microbial communities, the effectiveness of simple single or multi-strain live biopharmaceutical products/probiotics have been tested for treating BV: either with or without pre-treatment (“weeding”) with antibiotics (96). Evidence synthesized from >30 clinical trials that tested different probiotics for treating BV suggests that *Lactobacillus* strain, its origin, route of administration and pre-treatment status of participants are important determinants of treatment outcomes (96). Many trials tested single *Lactobacillus* strain-containing probiotics, frequently not vaginal in origin, raising questions about whether one strain would fit all the possible genetic and immunological permutations of all potential recipients (97), regardless of geography and genetics (98).

Two clinical trials have tested vaginally-delivered *L. crispatus* CTV-05 (LACTIN-V), after metronidazole treatment (99, 100). Both trials showed that the product was generally safe and acceptable to women, significantly decreased recurrence of BV and increased *L. crispatus* colonization among recipients. Short-term cure rates of 100%, and long-term cure rates of 70% were achieved, with BV by Amsel's criteria being the endpoint. However, efficacy of LACTIN-V appeared to depend on the extent to which metronidazole “cured” BV (101), particularly the extent of *G. vaginalis* clearance (102). Other factors that influenced efficacy of LACTIN-V included condomless sex or having menses, suggesting that semen and menstrual blood affected CTV-05 colonization (100). While these products are promising, the lack of microbiota data from Africa (in its diversity) limits evidence-based live therapeutic product formulation and subsequent clinical trials. No clinical studies have yet evaluated the effect of live biotherapeutics for BV treatment on reducing HIV infections.

## Conclusion

The importance of a healthy vaginal microbiome and the potential benefits of specifically tailored probiotics that contain beneficial *Lactobacillus* strains is clear. The new approaches being developed aim to maintain a healthy vaginal environment following BV treatment, that may reduce HIV risk in women. It is critical to focus on Africa to reveal and harness our “missing microbes”, as these will provide the foundations upon which microbiome-centred reproductive health solutions will be built. If appropriately focused on regionally-responsive microbes, these new approaches will have the greatest probability of being sustainable and efficacious for all Africa's women.
