# Supplementary material for: Ecology meets reproductive medicine in HIV prevention: the case for geography-informed approaches for bacterial vaginosis in Africa
Source: Front Reprod Health. 2024 Nov 27;6:1431306. doi: 10.3389/frph.2024.1431306 (PMC11631894; doi:10.3389/frph.2024.1431306)
Supplement: Supplementary file 1 [file Table1.docx]

**Supplementary Material**

Supplementary Table 1A. RefSeq Country Counts used to generate Figure 1 – page 1

Supplementary Table 1B. All RefSeq genome sources used to generate Figure 1 – page 4

Supplementary Table 1C. RefSeq *Lactobacillaceae* sources – page 117

Supplementary Table 1D. RefSeq *Prevotella* spp. sources – page 119

Supplementary Table 1E. RefSeq *Gardnerella* spp. sources – page 120

Supplementary Table 1F. RefSeq *Fannyhessea vaginae* sources – page 122

**Supplementary 1A. RefSeq Country Counts used to generate Figure 1**

| Taxon | Country | Count |
| --- | --- | --- |
| Lactobacillaceae | USA | 761 |
| Lactobacillaceae | China | 721 |
| Lactobacillaceae | Missing | 264 |
| Lactobacillaceae | South Korea | 204 |
| Lactobacillaceae | France | 112 |
| Lactobacillaceae | Switzerland | 82 |
| Lactobacillaceae | Italy | 81 |
| Lactobacillaceae | United Kingdom | 65 |
| BV-associated | USA | 60 |
| Lactobacillaceae | Russia | 58 |
| Lactobacillaceae | Canada | 47 |
| Lactobacillaceae | India | 46 |
| Lactobacillaceae | Germany | 45 |
| BV-associated | USA | 44 |
| Lactobacillaceae | Japan | 43 |
| Lactobacillaceae | South Africa | 37 |
| Lactobacillaceae | Netherlands | 36 |
| Lactobacillaceae | Thailand | 36 |
| Lactobacillaceae | Ireland | 33 |
| Lactobacillaceae | Greece | 31 |
| Lactobacillaceae | Spain | 30 |
| Lactobacillaceae | Taiwan | 21 |
| Lactobacillaceae | Argentina | 19 |
| BV-associated | China | 17 |
| Lactobacillaceae | Croatia | 15 |
| Lactobacillaceae | Ghana | 14 |
| Lactobacillaceae | Viet Nam | 14 |
| BV-associated | Australia | 14 |
| Lactobacillaceae | Brazil | 13 |
| Lactobacillaceae | Finland | 13 |
| Lactobacillaceae | Czech Republic | 12 |
| BV-associated | Missing | 12 |
| Lactobacillaceae | Bulgaria | 11 |
| Lactobacillaceae | Denmark | 11 |
| Lactobacillaceae | Egypt | 11 |
| Lactobacillaceae | Pakistan | 10 |
| Lactobacillaceae | Slovakia | 10 |
| Lactobacillaceae | Lithuania | 9 |
| BV-associated | Kenya | 9 |
| Lactobacillaceae | Indonesia | 8 |
| Lactobacillaceae | Israel | 8 |
| Lactobacillaceae | New Zealand | 8 |
| Lactobacillaceae | Sweden | 8 |
| BV-associated | Belgium | 8 |
| Lactobacillaceae | Georgia | 7 |
| Lactobacillaceae | Kenya | 7 |
| Lactobacillaceae | Nigeria | 7 |
| Lactobacillaceae | Papua New Guinea | 7 |
| Lactobacillaceae | Poland | 7 |
| Lactobacillaceae | Slovenia | 7 |
| Lactobacillaceae | Turkey | 7 |
| Lactobacillaceae | Belgium | 6 |
| Lactobacillaceae | Mexico | 6 |
| Lactobacillaceae | Mongolia | 6 |
| Lactobacillaceae | Australia | 5 |
| Lactobacillaceae | Algeria | 4 |
| Lactobacillaceae | Botswana | 4 |
| Lactobacillaceae | Ethiopia | 4 |
| Lactobacillaceae | Iran | 4 |
| Lactobacillaceae | Kazakhstan | 4 |
| BV-associated | Sweden | 4 |
| BV-associated | United Kingdom | 4 |
| Lactobacillaceae | Ecuador | 3 |
| Lactobacillaceae | Madagascar | 3 |
| Lactobacillaceae | Malaysia | 3 |
| Lactobacillaceae | Belarus | 2 |
| Lactobacillaceae | Benin | 2 |
| Lactobacillaceae | Chile | 2 |
| Lactobacillaceae | Hong Kong | 2 |
| Lactobacillaceae | Iraq | 2 |
| Lactobacillaceae | Korea | 2 |
| Lactobacillaceae | Norway | 2 |
| Lactobacillaceae | Philippines | 2 |
| Lactobacillaceae | Serbia | 2 |
| Lactobacillaceae | Singapore | 2 |
| Lactobacillaceae | Tajikistan | 2 |
| Lactobacillaceae | Zimbabwe | 2 |
| BV-associated | Germany | 2 |
| BV-associated | Canada | 2 |
| BV-associated | Russia | 2 |
| BV-associated | Portugal | 2 |
| Lactobacillaceae | Armenia | 1 |
| Lactobacillaceae | Bangladesh | 1 |
| Lactobacillaceae | Hungary | 1 |
| Lactobacillaceae | India | 1 |
| Lactobacillaceae | Lebanon | 1 |
| Lactobacillaceae | Mali | 1 |
| Lactobacillaceae | Peru | 1 |
| Lactobacillaceae | Portugal | 1 |
| Lactobacillaceae | Senegal | 1 |
| Lactobacillaceae | Sudan | 1 |
| Lactobacillaceae | Tunisia | 1 |
| Lactobacillaceae | USA | 1 |
| BV-associated | Japan | 1 |
| BV-associated | Bangladesh | 1 |
| BV-associated | Denmark | 1 |
| BV-associated | France | 1 |
| BV-associated | Italy | 1 |

**Supplementary Table 1B. All RefSeq genome sources used to generate Figure 1**

| RefSeq_Assembly_Accession | Species | Country | Region | Group | Probiotic_BV |
| --- | --- | --- | --- | --- | --- |
| GCF_000613765.1 | Prevotella amnii | Sweden | Europe | Prevotella | BV-associated |
| GCF_000613345.1 | Prevotella disiens | USA | North America | Prevotella | BV-associated |
| GCF_003609775.1 | Prevotella melaninogenica | Japan | Asia | Prevotella | BV-associated |
| GCF_946223055.1 | Prevotella melaninogenica | USA | North America | Prevotella | BV-associated |
| GCF_946997305.1 | Prevotella disiens | USA | North America | Prevotella | BV-associated |
| GCF_963524475.1 | Prevotella melaninogenica | Australia | Oceania | Prevotella | BV-associated |
| GCF_938034465.1 | Prevotella melaninogenica | Germany | Europe | Prevotella | BV-associated |
| GCF_901875335.1 | Prevotella melaninogenica | Bangladesh | Asia | Prevotella | BV-associated |
| GCF_902399785.1 | Prevotella disiens | China | Asia | Prevotella | BV-associated |
| GCF_000144405.1 | Prevotella melaninogenica | USA | North America | Prevotella | BV-associated |
| GCF_000177355.1 | Prevotella amnii | Missing | Missing | Prevotella | BV-associated |
| GCF_000177315.1 | Prevotella bivia | Missing | Missing | Prevotella | BV-associated |
| GCF_000179675.1 | Prevotella disiens | Belgium | Europe | Prevotella | BV-associated |
| GCF_000262545.1 | Prevotella bivia | USA | North America | Prevotella | BV-associated |
| GCF_000467875.1 | Prevotella disiens | USA | North America | Prevotella | BV-associated |
| GCF_000378745.1 | Prevotella amnii | Sweden | Europe | Prevotella | BV-associated |
| GCF_000163035.1 | Prevotella melaninogenica | Canada | North America | Prevotella | BV-associated |
| GCF_000759045.1 | Prevotella bivia | USA | North America | Prevotella | BV-associated |
| GCF_000759315.1 | Prevotella amnii | USA | North America | Prevotella | BV-associated |
| GCF_000759245.1 | Prevotella bivia | Missing | Missing | Prevotella | BV-associated |
| GCF_000759165.1 | Prevotella bivia | USA | North America | Prevotella | BV-associated |
| GCF_000759305.1 | Prevotella melaninogenica | Missing | Missing | Prevotella | BV-associated |
| GCF_000759225.1 | Prevotella disiens | USA | North America | Prevotella | BV-associated |
| GCF_001065995.1 | Prevotella bivia | USA | North America | Prevotella | BV-associated |
| GCF_001546565.2 | Prevotella bivia | USA | North America | Prevotella | BV-associated |
| GCF_001574405.1 | Prevotella bivia | USA | North America | Prevotella | BV-associated |
| GCF_001553225.1 | Prevotella amnii | Missing | Missing | Prevotella | BV-associated |
| GCF_002208725.2 | Prevotella melaninogenica | USA | North America | Prevotella | BV-associated |
| GCF_003437625.1 | Prevotella disiens | China | Asia | Prevotella | BV-associated |
| GCF_013267595.1 | Prevotella melaninogenica | USA | North America | Prevotella | BV-associated |
| GCF_018128045.1 | Prevotella melaninogenica | USA | North America | Prevotella | BV-associated |
| GCF_018128065.1 | Prevotella melaninogenica | USA | North America | Prevotella | BV-associated |
| GCF_018128005.1 | Prevotella melaninogenica | USA | North America | Prevotella | BV-associated |
| GCF_018127945.1 | Prevotella melaninogenica | USA | North America | Prevotella | BV-associated |
| GCF_018127965.1 | Prevotella melaninogenica | USA | North America | Prevotella | BV-associated |
| GCF_018127925.1 | Prevotella melaninogenica | USA | North America | Prevotella | BV-associated |
| GCF_018127905.1 | Prevotella melaninogenica | USA | North America | Prevotella | BV-associated |
| GCF_018127885.1 | Prevotella melaninogenica | USA | North America | Prevotella | BV-associated |
| GCF_019375935.1 | Prevotella melaninogenica | Australia | Oceania | Prevotella | BV-associated |
| GCF_019375815.1 | Prevotella melaninogenica | Australia | Oceania | Prevotella | BV-associated |
| GCF_019375775.1 | Prevotella melaninogenica | Australia | Oceania | Prevotella | BV-associated |
| GCF_019375745.1 | Prevotella melaninogenica | Australia | Oceania | Prevotella | BV-associated |
| GCF_019375715.1 | Prevotella melaninogenica | Australia | Oceania | Prevotella | BV-associated |
| GCF_019391945.1 | Prevotella melaninogenica | Australia | Oceania | Prevotella | BV-associated |
| GCF_019375575.1 | Prevotella melaninogenica | Australia | Oceania | Prevotella | BV-associated |
| GCF_019391815.1 | Prevotella melaninogenica | Australia | Oceania | Prevotella | BV-associated |
| GCF_019375605.1 | Prevotella melaninogenica | Australia | Oceania | Prevotella | BV-associated |
| GCF_019375525.1 | Prevotella melaninogenica | Australia | Oceania | Prevotella | BV-associated |
| GCF_019391775.1 | Prevotella melaninogenica | Australia | Oceania | Prevotella | BV-associated |
| GCF_019375485.1 | Prevotella melaninogenica | Australia | Oceania | Prevotella | BV-associated |
| GCF_019391735.1 | Prevotella melaninogenica | Australia | Oceania | Prevotella | BV-associated |
| GCF_020735905.1 | Prevotella melaninogenica | Germany | Europe | Prevotella | BV-associated |
| GCF_030219105.2 | Prevotella bivia | USA | North America | Prevotella | BV-associated |
| GCF_030223945.1 | Prevotella bivia | USA | North America | Prevotella | BV-associated |
| GCF_030219305.1 | Prevotella bivia | China | Asia | Prevotella | BV-associated |
| GCF_943913865.1 | Hoylesella timonensis | USA | North America | Prevotella | BV-associated |
| GCF_946998675.1 | Hoylesella timonensis | USA | North America | Prevotella | BV-associated |
| GCF_947041285.1 | Hoylesella timonensis | Denmark | Europe | Prevotella | BV-associated |
| GCF_963510825.1 | Hoylesella timonensis | USA | North America | Prevotella | BV-associated |
| GCF_000455445.1 | Hoylesella timonensis | France | Europe | Prevotella | BV-associated |
| GCF_000177055.1 | Hoylesella timonensis | USA | North America | Prevotella | BV-associated |
| GCF_000762405.1 | Hoylesella timonensis | USA | North America | Prevotella | BV-associated |
| GCF_002894165.1 | Hoylesella timonensis | USA | North America | Prevotella | BV-associated |
| GCF_002871515.1 | Hoylesella timonensis | USA | North America | Prevotella | BV-associated |
| GCF_015548615.1 | Hoylesella timonensis | USA | North America | Prevotella | BV-associated |
| GCF_001042655.1 | Gardnerella vaginalis | USA | North America | Gardnerella | BV-associated |
| GCF_943912865.1 | uncultured Gardnerella sp. | USA | North America | Gardnerella | BV-associated |
| GCF_947280595.1 | uncultured Gardnerella sp. | USA | North America | Gardnerella | BV-associated |
| GCF_940796375.1 | Gardnerella sp. Marseille-Q2328 | Missing | Missing | Gardnerella | BV-associated |
| GCF_902373565.1 | uncultured Gardnerella sp. | Italy | Europe | Gardnerella | BV-associated |
| GCF_000213955.1 | Gardnerella vaginalis | Missing | Missing | Gardnerella | BV-associated |
| GCF_000214315.1 | Gardnerella vaginalis | Missing | Missing | Gardnerella | BV-associated |
| GCF_000263435.1 | Gardnerella vaginalis | USA | North America | Gardnerella | BV-associated |
| GCF_000263535.1 | Gardnerella vaginalis | USA | North America | Gardnerella | BV-associated |
| GCF_000263555.1 | Gardnerella vaginalis | USA | North America | Gardnerella | BV-associated |
| GCF_000263475.1 | Gardnerella vaginalis | USA | North America | Gardnerella | BV-associated |
| GCF_000263495.1 | Gardnerella vaginalis | USA | North America | Gardnerella | BV-associated |
| GCF_000263595.1 | Gardnerella vaginalis | USA | North America | Gardnerella | BV-associated |
| GCF_000263615.1 | Gardnerella pickettii | USA | North America | Gardnerella | BV-associated |
| GCF_000263515.1 | Gardnerella pickettii | USA | North America | Gardnerella | BV-associated |
| GCF_000263635.1 | Gardnerella greenwoodii | USA | North America | Gardnerella | BV-associated |
| GCF_000263655.1 | Gardnerella vaginalis | USA | North America | Gardnerella | BV-associated |
| GCF_000414625.1 | Gardnerella pickettii | USA | North America | Gardnerella | BV-associated |
| GCF_000414585.1 | Gardnerella pickettii | USA | North America | Gardnerella | BV-associated |
| GCF_000414445.1 | Gardnerella vaginalis | USA | North America | Gardnerella | BV-associated |
| GCF_000414525.1 | Gardnerella vaginalis | USA | North America | Gardnerella | BV-associated |
| GCF_000414645.1 | Gardnerella vaginalis | USA | North America | Gardnerella | BV-associated |
| GCF_000414705.1 | Gardnerella vaginalis | USA | North America | Gardnerella | BV-associated |
| GCF_000414685.1 | Gardnerella vaginalis | USA | North America | Gardnerella | BV-associated |
| GCF_000414465.1 | Gardnerella vaginalis | USA | North America | Gardnerella | BV-associated |
| GCF_000414605.1 | Gardnerella pickettii | USA | North America | Gardnerella | BV-associated |
| GCF_000165615.1 | Gardnerella greenwoodii | Missing | Missing | Gardnerella | BV-associated |
| GCF_000165635.1 | Gardnerella vaginalis | Missing | Missing | Gardnerella | BV-associated |
| GCF_003369965.1 | Gardnerella vaginalis | Kenya | Africa | Gardnerella | BV-associated |
| GCF_003369895.1 | Gardnerella vaginalis | Kenya | Africa | Gardnerella | BV-associated |
| GCF_003369935.1 | Gardnerella vaginalis | Kenya | Africa | Gardnerella | BV-associated |
| GCF_003369875.1 | Gardnerella vaginalis | Canada | North America | Gardnerella | BV-associated |
| GCF_001049785.1 | Gardnerella vaginalis | USA | North America | Gardnerella | BV-associated |
| GCF_001563665.1 | Gardnerella vaginalis | Missing | Missing | Gardnerella | BV-associated |
| GCF_001546445.1 | Gardnerella pickettii | USA | North America | Gardnerella | BV-associated |
| GCF_001546455.1 | Gardnerella vaginalis | USA | North America | Gardnerella | BV-associated |
| GCF_001546485.1 | Gardnerella vaginalis | USA | North America | Gardnerella | BV-associated |
| GCF_001278345.1 | Gardnerella vaginalis | USA | North America | Gardnerella | BV-associated |
| GCF_003408835.1 | Gardnerella vaginalis | Kenya | Africa | Gardnerella | BV-associated |
| GCF_003408745.1 | Gardnerella vaginalis | Belgium | Europe | Gardnerella | BV-associated |
| GCF_003408785.1 | Gardnerella vaginalis | Kenya | Africa | Gardnerella | BV-associated |
| GCF_003408775.1 | Gardnerella vaginalis | Kenya | Africa | Gardnerella | BV-associated |
| GCF_003408845.1 | Gardnerella vaginalis | Kenya | Africa | Gardnerella | BV-associated |
| GCF_001660735.1 | Gardnerella vaginalis | USA | North America | Gardnerella | BV-associated |
| GCF_001660755.1 | Gardnerella vaginalis | USA | North America | Gardnerella | BV-associated |
| GCF_001641215.1 | Gardnerella sp. 30-4 | USA | North America | Gardnerella | BV-associated |
| GCF_001660745.1 | Gardnerella sp. 26-12 | USA | North America | Gardnerella | BV-associated |
| GCF_002896555.1 | Gardnerella vaginalis | USA | North America | Gardnerella | BV-associated |
| GCF_002894085.1 | Gardnerella sp. KA00735 | USA | North America | Gardnerella | BV-associated |
| GCF_002894105.1 | Gardnerella vaginalis | USA | North America | Gardnerella | BV-associated |
| GCF_002894125.1 | Gardnerella sp. DNF01162 | USA | North America | Gardnerella | BV-associated |
| GCF_001913835.1 | Gardnerella vaginalis | USA | North America | Gardnerella | BV-associated |
| GCF_002206225.1 | Gardnerella vaginalis | USA | North America | Gardnerella | BV-associated |
| GCF_003426405.1 | Gardnerella piotii | Belgium | Europe | Gardnerella | BV-associated |
| GCF_003426385.1 | Gardnerella piotii | Belgium | Europe | Gardnerella | BV-associated |
| GCF_003585655.1 | Gardnerella vaginalis | Kenya | Africa | Gardnerella | BV-associated |
| GCF_003585755.1 | Gardnerella vaginalis | Kenya | Africa | Gardnerella | BV-associated |
| GCF_002884835.1 | Gardnerella vaginalis | USA | North America | Gardnerella | BV-associated |
| GCF_002871635.1 | Gardnerella swidsinskii | USA | North America | Gardnerella | BV-associated |
| GCF_002884775.1 | Gardnerella greenwoodii | USA | North America | Gardnerella | BV-associated |
| GCF_002861165.1 | Gardnerella vaginalis | USA | North America | Gardnerella | BV-associated |
| GCF_002862045.1 | Gardnerella vaginalis | USA | North America | Gardnerella | BV-associated |
| GCF_002862015.1 | Gardnerella vaginalis | USA | North America | Gardnerella | BV-associated |
| GCF_002861965.1 | Gardnerella vaginalis | USA | North America | Gardnerella | BV-associated |
| GCF_002862005.1 | Gardnerella vaginalis | USA | North America | Gardnerella | BV-associated |
| GCF_002861975.1 | Gardnerella vaginalis | USA | North America | Gardnerella | BV-associated |
| GCF_002861945.1 | Gardnerella vaginalis | USA | North America | Gardnerella | BV-associated |
| GCF_002861925.1 | Gardnerella vaginalis | USA | North America | Gardnerella | BV-associated |
| GCF_002861905.1 | Gardnerella pickettii | USA | North America | Gardnerella | BV-associated |
| GCF_002861885.1 | Gardnerella pickettii | USA | North America | Gardnerella | BV-associated |
| GCF_003397705.1 | Gardnerella swidsinskii | Russia | Europe | Gardnerella | BV-associated |
| GCF_003397685.1 | Gardnerella vaginalis | USA | North America | Gardnerella | BV-associated |
| GCF_003293675.1 | Gardnerella leopoldii | Belgium | Europe | Gardnerella | BV-associated |
| GCF_003397585.1 | Gardnerella piotii | Belgium | Europe | Gardnerella | BV-associated |
| GCF_003397605.1 | Gardnerella vaginalis | Belgium | Europe | Gardnerella | BV-associated |
| GCF_003812765.1 | Gardnerella vaginalis | Missing | Missing | Gardnerella | BV-associated |
| GCF_013315115.1 | Gardnerella vaginalis | USA | North America | Gardnerella | BV-associated |
| GCF_013315075.1 | Gardnerella vaginalis | USA | North America | Gardnerella | BV-associated |
| GCF_013315085.1 | Gardnerella vaginalis | USA | North America | Gardnerella | BV-associated |
| GCF_013315045.1 | Gardnerella vaginalis | USA | North America | Gardnerella | BV-associated |
| GCF_013315025.1 | Gardnerella vaginalis | USA | North America | Gardnerella | BV-associated |
| GCF_013315005.1 | Gardnerella vaginalis | USA | North America | Gardnerella | BV-associated |
| GCF_014857145.1 | Gardnerella vaginalis | Russia | Europe | Gardnerella | BV-associated |
| GCF_023277725.1 | Gardnerella vaginalis | China | Asia | Gardnerella | BV-associated |
| GCF_023277705.1 | Gardnerella swidsinskii | China | Asia | Gardnerella | BV-associated |
| GCF_023277685.1 | Gardnerella vaginalis | China | Asia | Gardnerella | BV-associated |
| GCF_023277665.1 | Gardnerella vaginalis | China | Asia | Gardnerella | BV-associated |
| GCF_023277645.1 | Gardnerella vaginalis | China | Asia | Gardnerella | BV-associated |
| GCF_023277625.1 | Gardnerella vaginalis | China | Asia | Gardnerella | BV-associated |
| GCF_023277605.1 | Gardnerella vaginalis | China | Asia | Gardnerella | BV-associated |
| GCF_023277585.1 | Gardnerella piotii | China | Asia | Gardnerella | BV-associated |
| GCF_023277565.1 | Gardnerella vaginalis | China | Asia | Gardnerella | BV-associated |
| GCF_023016205.1 | Gardnerella vaginalis | United Kingdom | Europe | Gardnerella | BV-associated |
| GCF_023016185.1 | Gardnerella vaginalis | United Kingdom | Europe | Gardnerella | BV-associated |
| GCF_023016245.1 | Gardnerella vaginalis | United Kingdom | Europe | Gardnerella | BV-associated |
| GCF_023016225.1 | Gardnerella vaginalis | United Kingdom | Europe | Gardnerella | BV-associated |
| GCF_029226345.1 | Gardnerella pickettii | Portugal | Europe | Gardnerella | BV-associated |
| GCF_029207615.1 | Gardnerella greenwoodii | Portugal | Europe | Gardnerella | BV-associated |
| GCF_030218185.1 | Gardnerella vaginalis | USA | North America | Gardnerella | BV-associated |
| GCF_030217865.1 | Gardnerella vaginalis | USA | North America | Gardnerella | BV-associated |
| GCF_030216615.1 | Gardnerella vaginalis | USA | North America | Gardnerella | BV-associated |
| GCF_030215405.1 | Gardnerella vaginalis | USA | North America | Gardnerella | BV-associated |
| GCF_030213965.1 | Gardnerella vaginalis | USA | North America | Gardnerella | BV-associated |
| GCF_030230275.1 | Gardnerella swidsinskii | USA | North America | Gardnerella | BV-associated |
| GCF_030233905.1 | Gardnerella vaginalis | USA | North America | Gardnerella | BV-associated |
| GCF_030228445.1 | Gardnerella vaginalis | USA | North America | Gardnerella | BV-associated |
| GCF_030228365.1 | Gardnerella vaginalis | USA | North America | Gardnerella | BV-associated |
| GCF_900445305.1 | Fannyhessea vaginae | Sweden | Europe | Fannyhessea | BV-associated |
| GCF_963510035.1 | Fannyhessea vaginae | USA | North America | Fannyhessea | BV-associated |
| GCF_963512215.1 | Fannyhessea vaginae | USA | North America | Fannyhessea | BV-associated |
| GCF_963513095.1 | Fannyhessea vaginae | USA | North America | Fannyhessea | BV-associated |
| GCF_963513155.1 | Fannyhessea vaginae | USA | North America | Fannyhessea | BV-associated |
| GCF_000179715.1 | Fannyhessea vaginae | Belgium | Europe | Fannyhessea | BV-associated |
| GCF_001049775.1 | Fannyhessea vaginae | Sweden | Europe | Fannyhessea | BV-associated |
| GCF_001562845.1 | Fannyhessea vaginae | USA | North America | Fannyhessea | BV-associated |
| GCF_019400185.1 | Fannyhessea vaginae | China | Asia | Fannyhessea | BV-associated |
| GCF_019400195.1 | Fannyhessea vaginae | China | Asia | Fannyhessea | BV-associated |
| GCF_019400135.1 | Fannyhessea vaginae | China | Asia | Fannyhessea | BV-associated |
| GCF_019400085.1 | Fannyhessea vaginae | China | Asia | Fannyhessea | BV-associated |
| GCF_019400095.1 | Fannyhessea vaginae | China | Asia | Fannyhessea | BV-associated |
| GCF_018228745.1 | Lacticaseibacillus rhamnosus | China | Asia | Lactobacillaceae | Probiotic |
| GCF_002025085.1 | Lacticaseibacillus rhamnosus | Hong Kong | Asia | Lactobacillaceae | Probiotic |
| GCF_037118725.1 | Lacticaseibacillus rhamnosus | Viet Nam | Asia | Lactobacillaceae | Probiotic |
| GCF_029011275.1 | Lacticaseibacillus rhamnosus | USA | North America | Lactobacillaceae | Probiotic |
| GCF_029010255.1 | Lacticaseibacillus rhamnosus | USA | North America | Lactobacillaceae | Probiotic |
| GCF_022220485.1 | Lacticaseibacillus rhamnosus | China | Asia | Lactobacillaceae | Probiotic |
| GCF_035928125.1 | Lacticaseibacillus rhamnosus | China | Asia | Lactobacillaceae | Probiotic |
| GCF_031594435.1 | Lacticaseibacillus rhamnosus | China | Asia | Lactobacillaceae | Probiotic |
| GCF_024610975.1 | Lacticaseibacillus rhamnosus | China | Asia | Lactobacillaceae | Probiotic |
| GCF_031593775.1 | Lacticaseibacillus rhamnosus | China | Asia | Lactobacillaceae | Probiotic |
| GCF_026427555.1 | Lacticaseibacillus rhamnosus | China | Asia | Lactobacillaceae | Probiotic |
| GCF_008017355.1 | Lacticaseibacillus rhamnosus | Armenia | Europe | Lactobacillaceae | Probiotic |
| GCF_036454445.1 | Lacticaseibacillus rhamnosus | Tunisia | Africa | Lactobacillaceae | Probiotic |
| GCF_030227945.1 | Lacticaseibacillus rhamnosus | USA | North America | Lactobacillaceae | Probiotic |
| GCF_030233995.1 | Lacticaseibacillus rhamnosus | USA | North America | Lactobacillaceae | Probiotic |
| GCF_030224725.1 | Lacticaseibacillus rhamnosus | USA | North America | Lactobacillaceae | Probiotic |
| GCF_030217425.1 | Lacticaseibacillus rhamnosus | USA | North America | Lactobacillaceae | Probiotic |
| GCF_030217405.1 | Lacticaseibacillus rhamnosus | USA | North America | Lactobacillaceae | Probiotic |
| GCF_030217985.1 | Lacticaseibacillus rhamnosus | USA | North America | Lactobacillaceae | Probiotic |
| GCF_002848015.1 | Lacticaseibacillus rhamnosus | USA | North America | Lactobacillaceae | Probiotic |
| GCF_028878345.1 | Lacticaseibacillus rhamnosus | Italy | Europe | Lactobacillaceae | Probiotic |
| GCF_004798455.1 | Lacticaseibacillus rhamnosus | India | Asia | Lactobacillaceae | Probiotic |
| GCF_032335515.1 | Lacticaseibacillus rhamnosus | Spain | Europe | Lactobacillaceae | Probiotic |
| GCF_030361185.1 | Lacticaseibacillus rhamnosus | Russia | Europe | Lactobacillaceae | Probiotic |
| GCF_003129615.1 | Lacticaseibacillus rhamnosus | China | Asia | Lactobacillaceae | Probiotic |
| GCF_015377485.1 | Lacticaseibacillus rhamnosus | China | Asia | Lactobacillaceae | Probiotic |
| GCF_033802705.1 | Lacticaseibacillus rhamnosus | China | Asia | Lactobacillaceae | Probiotic |
| GCF_036595865.1 | Lacticaseibacillus rhamnosus | Thailand | Asia | Lactobacillaceae | Probiotic |
| GCF_036595825.1 | Lacticaseibacillus rhamnosus | Thailand | Asia | Lactobacillaceae | Probiotic |
| GCF_002960215.1 | Lacticaseibacillus rhamnosus | China | Asia | Lactobacillaceae | Probiotic |
| GCF_035594055.1 | Lacticaseibacillus rhamnosus | India | Asia | Lactobacillaceae | Probiotic |
| GCF_035334605.1 | Lacticaseibacillus rhamnosus | Taiwan | Asia | Lactobacillaceae | Probiotic |
| GCF_015160815.1 | Lacticaseibacillus rhamnosus | India | Asia | Lactobacillaceae | Probiotic |
| GCF_001981725.1 | Lacticaseibacillus rhamnosus | Missing | Europe | Lactobacillaceae | Probiotic |
| GCF_018458775.1 | Lacticaseibacillus rhamnosus | USA | North America | Lactobacillaceae | Probiotic |
| GCF_002027355.1 | Lacticaseibacillus rhamnosus | Australia | Oceania | Lactobacillaceae | Probiotic |
| GCF_001645615.1 | Lacticaseibacillus rhamnosus | Kazakhstan | Asia | Lactobacillaceae | Probiotic |
| GCF_000235785.1 | Lacticaseibacillus rhamnosus | Canada | North America | Lactobacillaceae | Probiotic |
| GCF_013377685.1 | Lacticaseibacillus rhamnosus | China | Asia | Lactobacillaceae | Probiotic |
| GCF_022509255.1 | Lacticaseibacillus rhamnosus | Pakistan | Asia | Lactobacillaceae | Probiotic |
| GCF_020826335.1 | Lacticaseibacillus rhamnosus | South Korea | Asia | Lactobacillaceae | Probiotic |
| GCF_002076955.1 | Lacticaseibacillus rhamnosus | Poland | Europe | Lactobacillaceae | Probiotic |
| GCF_000712515.1 | Lacticaseibacillus rhamnosus | Missing | Europe | Lactobacillaceae | Probiotic |
| GCF_000712505.1 | Lacticaseibacillus rhamnosus | Missing | Europe | Lactobacillaceae | Probiotic |
| GCF_025631035.1 | Lacticaseibacillus rhamnosus | Canada | North America | Lactobacillaceae | Probiotic |
| GCF_002406715.1 | Lacticaseibacillus rhamnosus | Canada | North America | Lactobacillaceae | Probiotic |
| GCF_002406745.1 | Lacticaseibacillus rhamnosus | Canada | North America | Lactobacillaceae | Probiotic |
| GCF_023913535.1 | Lacticaseibacillus rhamnosus | China | Asia | Lactobacillaceae | Probiotic |
| GCF_002406795.1 | Lacticaseibacillus rhamnosus | Canada | North America | Lactobacillaceae | Probiotic |
| GCF_013342125.1 | Lacticaseibacillus rhamnosus | Missing | North America | Lactobacillaceae | Probiotic |
| GCF_027692365.1 | Lacticaseibacillus rhamnosus | China | Asia | Lactobacillaceae | Probiotic |
| GCF_027692625.1 | Lacticaseibacillus rhamnosus | China | Asia | Lactobacillaceae | Probiotic |
| GCF_024718295.1 | Lacticaseibacillus rhamnosus | USA | North America | Lactobacillaceae | Probiotic |
| GCF_028878245.1 | Lacticaseibacillus rhamnosus | Italy | Europe | Lactobacillaceae | Probiotic |
| GCF_900636875.1 | Lacticaseibacillus rhamnosus | Missing | Europe | Lactobacillaceae | Probiotic |
| GCF_030269985.1 | Lacticaseibacillus rhamnosus | Pakistan | Asia | Lactobacillaceae | Probiotic |
| GCF_014639015.1 | Lacticaseibacillus rhamnosus | Egypt | Africa | Lactobacillaceae | Probiotic |
| GCF_030939245.1 | Lacticaseibacillus rhamnosus | Bulgaria | Europe | Lactobacillaceae | Probiotic |
| GCF_028878115.1 | Lacticaseibacillus rhamnosus | Italy | Europe | Lactobacillaceae | Probiotic |
| GCF_028878145.1 | Lacticaseibacillus rhamnosus | Italy | Europe | Lactobacillaceae | Probiotic |
| GCF_032841215.1 | Lacticaseibacillus rhamnosus | Greece | Europe | Lactobacillaceae | Probiotic |
| GCF_028878125.1 | Lacticaseibacillus rhamnosus | Italy | Europe | Lactobacillaceae | Probiotic |
| GCF_030480385.1 | Lacticaseibacillus rhamnosus | India | Asia | Lactobacillaceae | Probiotic |
| GCF_013167115.1 | Lacticaseibacillus rhamnosus | China | Asia | Lactobacillaceae | Probiotic |
| GCF_004125475.1 | Lacticaseibacillus rhamnosus | Taiwan | Asia | Lactobacillaceae | Probiotic |
| GCF_000311965.1 | Lacticaseibacillus rhamnosus | Missing | Oceania | Lactobacillaceae | Probiotic |
| GCF_000311945.1 | Lacticaseibacillus rhamnosus | Missing | Oceania | Lactobacillaceae | Probiotic |
| GCF_001656785.1 | Lacticaseibacillus rhamnosus | Missing | Europe | Lactobacillaceae | Probiotic |
| GCF_001656535.1 | Lacticaseibacillus rhamnosus | Missing | Europe | Lactobacillaceae | Probiotic |
| GCF_001656815.1 | Lacticaseibacillus rhamnosus | Missing | Europe | Lactobacillaceae | Probiotic |
| GCF_001656835.1 | Lacticaseibacillus rhamnosus | Missing | Europe | Lactobacillaceae | Probiotic |
| GCF_001656845.1 | Lacticaseibacillus rhamnosus | Missing | Europe | Lactobacillaceae | Probiotic |
| GCF_002103185.1 | Lacticaseibacillus rhamnosus | Finland | Europe | Lactobacillaceae | Probiotic |
| GCF_002103215.1 | Lacticaseibacillus rhamnosus | Finland | Europe | Lactobacillaceae | Probiotic |
| GCF_001657205.1 | Lacticaseibacillus rhamnosus | Missing | Europe | Lactobacillaceae | Probiotic |
| GCF_009805825.1 | Lacticaseibacillus rhamnosus | Finland | Europe | Lactobacillaceae | Probiotic |
| GCF_002103155.1 | Lacticaseibacillus rhamnosus | Finland | Europe | Lactobacillaceae | Probiotic |
| GCF_001656765.1 | Lacticaseibacillus rhamnosus | Missing | Europe | Lactobacillaceae | Probiotic |
| GCF_001657195.1 | Lacticaseibacillus rhamnosus | Missing | Europe | Lactobacillaceae | Probiotic |
| GCF_001656545.1 | Lacticaseibacillus rhamnosus | Missing | Europe | Lactobacillaceae | Probiotic |
| GCF_001656575.1 | Lacticaseibacillus rhamnosus | Missing | Europe | Lactobacillaceae | Probiotic |
| GCF_001656895.1 | Lacticaseibacillus rhamnosus | Missing | Europe | Lactobacillaceae | Probiotic |
| GCF_001656915.1 | Lacticaseibacillus rhamnosus | Missing | Europe | Lactobacillaceae | Probiotic |
| GCF_001656585.1 | Lacticaseibacillus rhamnosus | Missing | Europe | Lactobacillaceae | Probiotic |
| GCF_001656925.1 | Lacticaseibacillus rhamnosus | Missing | Europe | Lactobacillaceae | Probiotic |
| GCF_001656945.1 | Lacticaseibacillus rhamnosus | Missing | Europe | Lactobacillaceae | Probiotic |
| GCF_001656605.1 | Lacticaseibacillus rhamnosus | Missing | Europe | Lactobacillaceae | Probiotic |
| GCF_001656975.1 | Lacticaseibacillus rhamnosus | Missing | Europe | Lactobacillaceae | Probiotic |
| GCF_001656635.1 | Lacticaseibacillus rhamnosus | Missing | Europe | Lactobacillaceae | Probiotic |
| GCF_001656655.1 | Lacticaseibacillus rhamnosus | Missing | Europe | Lactobacillaceae | Probiotic |
| GCF_001656995.1 | Lacticaseibacillus rhamnosus | Missing | Europe | Lactobacillaceae | Probiotic |
| GCF_001656675.1 | Lacticaseibacillus rhamnosus | Missing | Europe | Lactobacillaceae | Probiotic |
| GCF_001656685.1 | Lacticaseibacillus rhamnosus | Missing | Europe | Lactobacillaceae | Probiotic |
| GCF_001657055.1 | Lacticaseibacillus rhamnosus | Missing | Europe | Lactobacillaceae | Probiotic |
| GCF_001657075.1 | Lacticaseibacillus rhamnosus | Missing | Europe | Lactobacillaceae | Probiotic |
| GCF_001657085.1 | Lacticaseibacillus rhamnosus | Missing | Europe | Lactobacillaceae | Probiotic |
| GCF_001656715.1 | Lacticaseibacillus rhamnosus | Missing | Europe | Lactobacillaceae | Probiotic |
| GCF_001657115.1 | Lacticaseibacillus rhamnosus | Missing | Europe | Lactobacillaceae | Probiotic |
| GCF_001657135.1 | Lacticaseibacillus rhamnosus | Missing | Europe | Lactobacillaceae | Probiotic |
| GCF_001656735.1 | Lacticaseibacillus rhamnosus | Missing | Europe | Lactobacillaceae | Probiotic |
| GCF_001657165.1 | Lacticaseibacillus rhamnosus | Missing | Europe | Lactobacillaceae | Probiotic |
| GCF_026966975.1 | Lacticaseibacillus rhamnosus | USA | North America | Lactobacillaceae | Probiotic |
| GCF_026967015.1 | Lacticaseibacillus rhamnosus | USA | North America | Lactobacillaceae | Probiotic |
| GCF_026966995.1 | Lacticaseibacillus rhamnosus | USA | North America | Lactobacillaceae | Probiotic |
| GCF_026967025.1 | Lacticaseibacillus rhamnosus | USA | North America | Lactobacillaceae | Probiotic |
| GCF_026967035.1 | Lacticaseibacillus rhamnosus | USA | North America | Lactobacillaceae | Probiotic |
| GCF_026967075.1 | Lacticaseibacillus rhamnosus | USA | North America | Lactobacillaceae | Probiotic |
| GCF_026967135.1 | Lacticaseibacillus rhamnosus | USA | North America | Lactobacillaceae | Probiotic |
| GCF_026967125.1 | Lacticaseibacillus rhamnosus | USA | North America | Lactobacillaceae | Probiotic |
| GCF_026967105.1 | Lacticaseibacillus rhamnosus | USA | North America | Lactobacillaceae | Probiotic |
| GCF_026967335.1 | Lacticaseibacillus rhamnosus | USA | North America | Lactobacillaceae | Probiotic |
| GCF_026967315.1 | Lacticaseibacillus rhamnosus | USA | North America | Lactobacillaceae | Probiotic |
| GCF_026967355.1 | Lacticaseibacillus rhamnosus | USA | North America | Lactobacillaceae | Probiotic |
| GCF_026967345.1 | Lacticaseibacillus rhamnosus | USA | North America | Lactobacillaceae | Probiotic |
| GCF_026967395.1 | Lacticaseibacillus rhamnosus | USA | North America | Lactobacillaceae | Probiotic |
| GCF_026967405.1 | Lacticaseibacillus rhamnosus | USA | North America | Lactobacillaceae | Probiotic |
| GCF_004125465.1 | Lacticaseibacillus rhamnosus | Taiwan | Asia | Lactobacillaceae | Probiotic |
| GCF_018449495.1 | Lacticaseibacillus rhamnosus | China | Asia | Lactobacillaceae | Probiotic |
| GCF_004125455.1 | Lacticaseibacillus rhamnosus | Taiwan | Asia | Lactobacillaceae | Probiotic |
| GCF_004125395.1 | Lacticaseibacillus rhamnosus | Taiwan | Asia | Lactobacillaceae | Probiotic |
| GCF_004010975.1 | Lacticaseibacillus rhamnosus | Taiwan | Asia | Lactobacillaceae | Probiotic |
| GCF_003129645.1 | Lacticaseibacillus rhamnosus | China | Asia | Lactobacillaceae | Probiotic |
| GCF_033977045.1 | Lacticaseibacillus rhamnosus | South Korea | Asia | Lactobacillaceae | Probiotic |
| GCF_002286235.1 | Lacticaseibacillus rhamnosus | South Korea | Asia | Lactobacillaceae | Probiotic |
| GCF_000508405.1 | Lacticaseibacillus rhamnosus | India | Asia | Lactobacillaceae | Probiotic |
| GCF_003046115.1 | Lacticaseibacillus rhamnosus | Turkey | Asia | Lactobacillaceae | Probiotic |
| GCF_001044025.1 | Lacticaseibacillus rhamnosus | Missing | Europe | Lactobacillaceae | Probiotic |
| GCF_009742715.1 | Lacticaseibacillus rhamnosus | Missing | Europe | Lactobacillaceae | Probiotic |
| GCF_000418495.1 | Lacticaseibacillus rhamnosus | Missing | Europe | Lactobacillaceae | Probiotic |
| GCF_000418475.1 | Lacticaseibacillus rhamnosus | Missing | Europe | Lactobacillaceae | Probiotic |
| GCF_000160175.1 | Lacticaseibacillus rhamnosus | Missing | North America | Lactobacillaceae | Probiotic |
| GCF_032465875.1 | Lacticaseibacillus rhamnosus | United Kingdom | Europe | Lactobacillaceae | Probiotic |
| GCF_032465975.1 | Lacticaseibacillus rhamnosus | United Kingdom | Europe | Lactobacillaceae | Probiotic |
| GCF_032465895.1 | Lacticaseibacillus rhamnosus | Missing | Europe | Lactobacillaceae | Probiotic |
| GCF_032465915.1 | Lacticaseibacillus rhamnosus | Belgium | Europe | Lactobacillaceae | Probiotic |
| GCF_032465935.1 | Lacticaseibacillus rhamnosus | Denmark | Europe | Lactobacillaceae | Probiotic |
| GCF_032465955.1 | Lacticaseibacillus rhamnosus | Denmark | Europe | Lactobacillaceae | Probiotic |
| GCF_032465995.1 | Lacticaseibacillus rhamnosus | Sweden | Europe | Lactobacillaceae | Probiotic |
| GCF_017795605.1 | Lacticaseibacillus rhamnosus | South Korea | Asia | Lactobacillaceae | Probiotic |
| GCF_000026525.1 | Lacticaseibacillus rhamnosus | Missing | Europe | Lactobacillaceae | Probiotic |
| GCF_027857765.1 | Lacticaseibacillus rhamnosus | Georgia | Asia | Lactobacillaceae | Probiotic |
| GCF_027857695.1 | Lacticaseibacillus rhamnosus | Georgia | Asia | Lactobacillaceae | Probiotic |
| GCF_027857795.1 | Lacticaseibacillus rhamnosus | Georgia | Asia | Lactobacillaceae | Probiotic |
| GCF_027857735.1 | Lacticaseibacillus rhamnosus | Georgia | Asia | Lactobacillaceae | Probiotic |
| GCF_027857715.1 | Lacticaseibacillus rhamnosus | Georgia | Asia | Lactobacillaceae | Probiotic |
| GCF_027857655.1 | Lacticaseibacillus rhamnosus | Georgia | Asia | Lactobacillaceae | Probiotic |
| GCF_027857665.1 | Lacticaseibacillus rhamnosus | Georgia | Asia | Lactobacillaceae | Probiotic |
| GCF_029961125.1 | Lacticaseibacillus rhamnosus | India | Asia | Lactobacillaceae | Probiotic |
| GCF_030876785.1 | Lacticaseibacillus rhamnosus | Missing | North America | Lactobacillaceae | Probiotic |
| GCF_000784395.1 | Lacticaseibacillus rhamnosus | Thailand | Asia | Lactobacillaceae | Probiotic |
| GCF_000784375.1 | Lacticaseibacillus rhamnosus | Thailand | Asia | Lactobacillaceae | Probiotic |
| GCF_000784405.1 | Lacticaseibacillus rhamnosus | Thailand | Asia | Lactobacillaceae | Probiotic |
| GCF_001991035.1 | Lacticaseibacillus rhamnosus | Brazil | South America | Lactobacillaceae | Probiotic |
| GCF_025195045.1 | Lacticaseibacillus rhamnosus | China | Asia | Lactobacillaceae | Probiotic |
| GCF_016653515.1 | Lacticaseibacillus rhamnosus | China | Asia | Lactobacillaceae | Probiotic |
| GCF_000735255.1 | Lacticaseibacillus rhamnosus | Russia | Europe | Lactobacillaceae | Probiotic |
| GCF_030034775.1 | Lacticaseibacillus rhamnosus | Russia | Europe | Lactobacillaceae | Probiotic |
| GCF_015238575.1 | Lacticaseibacillus rhamnosus | China | Asia | Lactobacillaceae | Probiotic |
| GCF_003433395.1 | Lacticaseibacillus rhamnosus | Missing | Missing | Lactobacillaceae | Probiotic |
| GCF_030286545.1 | Lacticaseibacillus rhamnosus | South Africa | Africa | Lactobacillaceae | Probiotic |
| GCF_901971795.1 | Lacticaseibacillus rhamnosus | Spain | Europe | Lactobacillaceae | Probiotic |
| GCF_901971785.1 | Lacticaseibacillus rhamnosus | Spain | Europe | Lactobacillaceae | Probiotic |
| GCF_020844065.2 | Lacticaseibacillus rhamnosus | USA | North America | Lactobacillaceae | Probiotic |
| GCF_009429065.1 | Lacticaseibacillus rhamnosus | South Korea | Asia | Lactobacillaceae | Probiotic |
| GCF_021300595.1 | Lacticaseibacillus rhamnosus | Russia | Europe | Lactobacillaceae | Probiotic |
| GCF_002238035.1 | Lacticaseibacillus rhamnosus | Argentina | South America | Lactobacillaceae | Probiotic |
| GCF_008727835.1 | Lacticaseibacillus rhamnosus | China | Asia | Lactobacillaceae | Probiotic |
| GCF_024397415.1 | Lacticaseibacillus rhamnosus | New Zealand | Oceania | Lactobacillaceae | Probiotic |
| GCF_000173255.4 | Lacticaseibacillus rhamnosus | New Zealand | Oceania | Lactobacillaceae | Probiotic |
| GCF_001756565.1 | Lacticaseibacillus rhamnosus | India | Asia | Lactobacillaceae | Probiotic |
| GCF_002762445.1 | Lacticaseibacillus rhamnosus | India | Asia | Lactobacillaceae | Probiotic |
| GCF_024665595.1 | Lacticaseibacillus rhamnosus | Canada | North America | Lactobacillaceae | Probiotic |
| GCF_000026505.1 | Lacticaseibacillus rhamnosus | USA | North America | Lactobacillaceae | Probiotic |
| GCF_003353455.1 | Lacticaseibacillus rhamnosus | South Korea | Asia | Lactobacillaceae | Probiotic |
| GCF_028475085.1 | Lacticaseibacillus rhamnosus | USA | North America | Lactobacillaceae | Probiotic |
| GCF_031626835.1 | Lacticaseibacillus rhamnosus | Italy | Europe | Lactobacillaceae | Probiotic |
| GCF_029874435.1 | Lacticaseibacillus rhamnosus | Lebanon | Asia | Lactobacillaceae | Probiotic |
| GCF_031202655.1 | Lacticaseibacillus rhamnosus | China | Asia | Lactobacillaceae | Probiotic |
| GCF_005864245.1 | Lacticaseibacillus rhamnosus | Switzerland | Europe | Lactobacillaceae | Probiotic |
| GCF_018966895.1 | Lacticaseibacillus rhamnosus | Russia | Europe | Lactobacillaceae | Probiotic |
| GCF_029269765.1 | Lacticaseibacillus rhamnosus | Turkey | Asia | Lactobacillaceae | Probiotic |
| GCF_000712495.1 | Lacticaseibacillus rhamnosus | Missing | Europe | Lactobacillaceae | Probiotic |
| GCF_002287945.1 | Lacticaseibacillus rhamnosus | Missing | Europe | Lactobacillaceae | Probiotic |
| GCF_003061625.1 | Lacticaseibacillus rhamnosus | USA | North America | Lactobacillaceae | Probiotic |
| GCF_003052965.1 | Lacticaseibacillus rhamnosus | USA | North America | Lactobacillaceae | Probiotic |
| GCF_003052985.1 | Lacticaseibacillus rhamnosus | USA | North America | Lactobacillaceae | Probiotic |
| GCF_003061605.1 | Lacticaseibacillus rhamnosus | USA | North America | Lactobacillaceae | Probiotic |
| GCF_003052925.1 | Lacticaseibacillus rhamnosus | USA | North America | Lactobacillaceae | Probiotic |
| GCF_003061565.1 | Lacticaseibacillus rhamnosus | USA | North America | Lactobacillaceae | Probiotic |
| GCF_003061645.1 | Lacticaseibacillus rhamnosus | USA | North America | Lactobacillaceae | Probiotic |
| GCF_003061705.1 | Lacticaseibacillus rhamnosus | USA | North America | Lactobacillaceae | Probiotic |
| GCF_003052945.1 | Lacticaseibacillus rhamnosus | USA | North America | Lactobacillaceae | Probiotic |
| GCF_003061665.1 | Lacticaseibacillus rhamnosus | USA | North America | Lactobacillaceae | Probiotic |
| GCF_032248855.1 | Lacticaseibacillus rhamnosus | South Korea | Asia | Lactobacillaceae | Probiotic |
| GCF_014155845.1 | Lacticaseibacillus rhamnosus | Ireland | Europe | Lactobacillaceae | Probiotic |
| GCF_030490305.1 | Lacticaseibacillus rhamnosus | South Korea | Asia | Lactobacillaceae | Probiotic |
| GCF_024053515.2 | Lacticaseibacillus rhamnosus | South Korea | Asia | Lactobacillaceae | Probiotic |
| GCF_024158105.2 | Lacticaseibacillus rhamnosus | South Korea | Asia | Lactobacillaceae | Probiotic |
| GCF_030929905.1 | Lacticaseibacillus rhamnosus | Argentina | South America | Lactobacillaceae | Probiotic |
| GCF_036583945.1 | Lacticaseibacillus rhamnosus | China | Asia | Lactobacillaceae | Probiotic |
| GCF_004167055.1 | Lacticaseibacillus rhamnosus | USA | North America | Lactobacillaceae | Probiotic |
| GCF_001005625.1 | Lacticaseibacillus rhamnosus | France | Europe | Lactobacillaceae | Probiotic |
| GCF_000932035.1 | Lacticaseibacillus rhamnosus | Kazakhstan | Asia | Lactobacillaceae | Probiotic |
| GCF_024442295.1 | Lacticaseibacillus rhamnosus | China | Asia | Lactobacillaceae | Probiotic |
| GCF_025189365.1 | Lacticaseibacillus rhamnosus | France | Europe | Lactobacillaceae | Probiotic |
| GCF_025189465.1 | Lacticaseibacillus rhamnosus | Egypt | Africa | Lactobacillaceae | Probiotic |
| GCF_025189545.1 | Lacticaseibacillus rhamnosus | Egypt | Africa | Lactobacillaceae | Probiotic |
| GCF_025189285.1 | Lacticaseibacillus rhamnosus | France | Europe | Lactobacillaceae | Probiotic |
| GCF_025189305.1 | Lacticaseibacillus rhamnosus | France | Europe | Lactobacillaceae | Probiotic |
| GCF_025189415.1 | Lacticaseibacillus rhamnosus | France | Europe | Lactobacillaceae | Probiotic |
| GCF_025212175.1 | Lacticaseibacillus rhamnosus | Spain | Europe | Lactobacillaceae | Probiotic |
| GCF_025189405.1 | Lacticaseibacillus rhamnosus | France | Europe | Lactobacillaceae | Probiotic |
| GCF_025189315.1 | Lacticaseibacillus rhamnosus | Missing | Missing | Lactobacillaceae | Probiotic |
| GCF_025189565.1 | Lacticaseibacillus rhamnosus | Egypt | Africa | Lactobacillaceae | Probiotic |
| GCF_025189525.1 | Lacticaseibacillus rhamnosus | Egypt | Africa | Lactobacillaceae | Probiotic |
| GCF_025189505.1 | Lacticaseibacillus rhamnosus | Egypt | Africa | Lactobacillaceae | Probiotic |
| GCF_025189475.1 | Lacticaseibacillus rhamnosus | Egypt | Africa | Lactobacillaceae | Probiotic |
| GCF_025190795.1 | Lacticaseibacillus rhamnosus | Egypt | Africa | Lactobacillaceae | Probiotic |
| GCF_025189345.1 | Lacticaseibacillus rhamnosus | Missing | Missing | Lactobacillaceae | Probiotic |
| GCF_025189425.1 | Lacticaseibacillus rhamnosus | France | Europe | Lactobacillaceae | Probiotic |
| GCF_025189605.1 | Lacticaseibacillus rhamnosus | France | Europe | Lactobacillaceae | Probiotic |
| GCF_025189585.1 | Lacticaseibacillus rhamnosus | Egypt | Africa | Lactobacillaceae | Probiotic |
| GCF_025189385.1 | Lacticaseibacillus rhamnosus | France | Europe | Lactobacillaceae | Probiotic |
| GCF_025189265.1 | Lacticaseibacillus rhamnosus | France | Europe | Lactobacillaceae | Probiotic |
| GCF_019990845.1 | Lacticaseibacillus rhamnosus | Hungary | Europe | Lactobacillaceae | Probiotic |
| GCF_018141205.1 | Lacticaseibacillus rhamnosus | USA | North America | Lactobacillaceae | Probiotic |
| GCF_014212185.1 | Lacticaseibacillus rhamnosus | Bulgaria | Europe | Lactobacillaceae | Probiotic |
| GCF_000226235.1 | Lacticaseibacillus rhamnosus | China | Asia | Lactobacillaceae | Probiotic |
| GCF_030237645.1 | Lacticaseibacillus rhamnosus | China | Asia | Lactobacillaceae | Probiotic |
| GCF_030237905.1 | Lacticaseibacillus rhamnosus | China | Asia | Lactobacillaceae | Probiotic |
| GCF_900070175.1 | Lacticaseibacillus rhamnosus | Missing | Europe | Lactobacillaceae | Probiotic |
| GCF_001368735.1 | Lacticaseibacillus rhamnosus | Missing | Europe | Lactobacillaceae | Probiotic |
| GCF_009679265.1 | Lacticaseibacillus rhamnosus | USA | North America | Lactobacillaceae | Probiotic |
| GCF_009679295.1 | Lacticaseibacillus rhamnosus | USA | North America | Lactobacillaceae | Probiotic |
| GCF_009679355.1 | Lacticaseibacillus rhamnosus | USA | North America | Lactobacillaceae | Probiotic |
| GCF_009679345.1 | Lacticaseibacillus rhamnosus | USA | North America | Lactobacillaceae | Probiotic |
| GCF_009679335.1 | Lacticaseibacillus rhamnosus | USA | North America | Lactobacillaceae | Probiotic |
| GCF_009679395.1 | Lacticaseibacillus rhamnosus | USA | North America | Lactobacillaceae | Probiotic |
| GCF_009679405.1 | Lacticaseibacillus rhamnosus | USA | North America | Lactobacillaceae | Probiotic |
| GCF_009679255.1 | Lacticaseibacillus rhamnosus | USA | North America | Lactobacillaceae | Probiotic |
| GCF_008831425.1 | Lacticaseibacillus rhamnosus | Missing | Missing | Lactobacillaceae | Probiotic |
| GCF_009720565.1 | Lacticaseibacillus rhamnosus | Missing | Missing | Lactobacillaceae | Probiotic |
| GCF_001988935.1 | Lacticaseibacillus rhamnosus | Kenya | Africa | Lactobacillaceae | Probiotic |
| GCF_016599675.1 | Lacticaseibacillus rhamnosus | Bulgaria | Europe | Lactobacillaceae | Probiotic |
| GCF_002406705.1 | Lacticaseibacillus rhamnosus | Canada | North America | Lactobacillaceae | Probiotic |
| GCF_000233755.1 | Lacticaseibacillus rhamnosus | Missing | Missing | Lactobacillaceae | Probiotic |
| GCF_000011045.1 | Lacticaseibacillus rhamnosus | USA | North America | Lactobacillaceae | Probiotic |
| GCF_000235865.1 | Lacticaseibacillus rhamnosus | Missing | Missing | Lactobacillaceae | Probiotic |
| GCF_001831235.1 | Lacticaseibacillus rhamnosus | Missing | Missing | Lactobacillaceae | Probiotic |
| GCF_001831215.1 | Lacticaseibacillus rhamnosus | United Kingdom | Europe | Lactobacillaceae | Probiotic |
| GCF_001831225.1 | Lacticaseibacillus rhamnosus | United Kingdom | Europe | Lactobacillaceae | Probiotic |
| GCF_001590655.1 | Lacticaseibacillus rhamnosus | Australia | Oceania | Lactobacillaceae | Probiotic |
| GCF_001831275.1 | Lacticaseibacillus rhamnosus | Missing | Missing | Lactobacillaceae | Probiotic |
| GCF_018286375.1 | Lacticaseibacillus rhamnosus | USA | North America | Lactobacillaceae | Probiotic |
| GCF_003573615.1 | Lacticaseibacillus rhamnosus | India | Asia | Lactobacillaceae | Probiotic |
| GCF_013425665.1 | Lacticaseibacillus rhamnosus | USA | North America | Lactobacillaceae | Probiotic |
| GCF_013425735.1 | Lacticaseibacillus rhamnosus | USA | North America | Lactobacillaceae | Probiotic |
| GCF_013425605.1 | Lacticaseibacillus rhamnosus | USA | North America | Lactobacillaceae | Probiotic |
| GCF_013425645.1 | Lacticaseibacillus rhamnosus | USA | North America | Lactobacillaceae | Probiotic |
| GCF_013425655.1 | Lacticaseibacillus rhamnosus | USA | North America | Lactobacillaceae | Probiotic |
| GCF_013425555.1 | Lacticaseibacillus rhamnosus | USA | North America | Lactobacillaceae | Probiotic |
| GCF_013425725.1 | Lacticaseibacillus rhamnosus | USA | North America | Lactobacillaceae | Probiotic |
| GCF_013425615.1 | Lacticaseibacillus rhamnosus | USA | North America | Lactobacillaceae | Probiotic |
| GCF_013425545.1 | Lacticaseibacillus rhamnosus | USA | North America | Lactobacillaceae | Probiotic |
| GCF_013425565.1 | Lacticaseibacillus rhamnosus | USA | North America | Lactobacillaceae | Probiotic |
| GCF_013425775.1 | Lacticaseibacillus rhamnosus | USA | North America | Lactobacillaceae | Probiotic |
| GCF_029543065.1 | Lacticaseibacillus rhamnosus | China | Asia | Lactobacillaceae | Probiotic |
| GCF_001068015.1 | Lacticaseibacillus rhamnosus | USA | North America | Lactobacillaceae | Probiotic |
| GCF_001068195.1 | Lacticaseibacillus rhamnosus | USA | North America | Lactobacillaceae | Probiotic |
| GCF_001067885.1 | Lacticaseibacillus rhamnosus | USA | North America | Lactobacillaceae | Probiotic |
| GCF_001067625.1 | Lacticaseibacillus rhamnosus | USA | North America | Lactobacillaceae | Probiotic |
| GCF_001066715.1 | Lacticaseibacillus rhamnosus | USA | North America | Lactobacillaceae | Probiotic |
| GCF_001067335.1 | Lacticaseibacillus rhamnosus | USA | North America | Lactobacillaceae | Probiotic |
| GCF_001067215.1 | Lacticaseibacillus rhamnosus | USA | North America | Lactobacillaceae | Probiotic |
| GCF_001067025.1 | Lacticaseibacillus rhamnosus | USA | North America | Lactobacillaceae | Probiotic |
| GCF_001066975.1 | Lacticaseibacillus rhamnosus | USA | North America | Lactobacillaceae | Probiotic |
| GCF_001065365.1 | Lacticaseibacillus rhamnosus | USA | North America | Lactobacillaceae | Probiotic |
| GCF_001063655.1 | Lacticaseibacillus rhamnosus | USA | North America | Lactobacillaceae | Probiotic |
| GCF_000699985.1 | Lacticaseibacillus rhamnosus | Russia | Europe | Lactobacillaceae | Probiotic |
| GCF_004122925.1 | Lacticaseibacillus rhamnosus | China | Asia | Lactobacillaceae | Probiotic |
| GCF_002158925.1 | Lacticaseibacillus rhamnosus | Korea | Asia | Lactobacillaceae | Probiotic |
| GCF_902166035.1 | Lacticaseibacillus rhamnosus | United Kingdom | Europe | Lactobacillaceae | Probiotic |
| GCF_001044405.1 | Lacticaseibacillus rhamnosus | Russia | Europe | Lactobacillaceae | Probiotic |
| GCF_001064785.1 | Lacticaseibacillus rhamnosus | USA | North America | Lactobacillaceae | Probiotic |
| GCF_022802755.1 | Lacticaseibacillus rhamnosus | Australia | Oceania | Lactobacillaceae | Probiotic |
| GCF_001064515.1 | Lacticaseibacillus rhamnosus | USA | North America | Lactobacillaceae | Probiotic |
| GCF_022802735.1 | Lacticaseibacillus rhamnosus | Australia | Oceania | Lactobacillaceae | Probiotic |
| GCF_001044415.1 | Lacticaseibacillus rhamnosus | Russia | Europe | Lactobacillaceae | Probiotic |
| GCF_000814485.1 | Lacticaseibacillus rhamnosus | Russia | Europe | Lactobacillaceae | Probiotic |
| GCF_000743075.1 | Lacticaseibacillus rhamnosus | Russia | Europe | Lactobacillaceae | Probiotic |
| GCF_028322835.1 | Lacticaseibacillus rhamnosus | USA | North America | Lactobacillaceae | Probiotic |
| GCF_001062955.1 | Lacticaseibacillus rhamnosus | USA | North America | Lactobacillaceae | Probiotic |
| GCF_001062885.1 | Lacticaseibacillus rhamnosus | USA | North America | Lactobacillaceae | Probiotic |
| GCF_016887825.1 | Lacticaseibacillus rhamnosus | Italy | Europe | Lactobacillaceae | Probiotic |
| GCF_000801045.1 | Lacticaseibacillus rhamnosus | Russia | Europe | Lactobacillaceae | Probiotic |
| GCF_016887865.1 | Lacticaseibacillus rhamnosus | Italy | Europe | Lactobacillaceae | Probiotic |
| GCF_015549085.1 | Lacticaseibacillus rhamnosus | USA | North America | Lactobacillaceae | Probiotic |
| GCF_015558325.1 | Lacticaseibacillus rhamnosus | USA | North America | Lactobacillaceae | Probiotic |
| GCF_015548835.1 | Lacticaseibacillus rhamnosus | USA | North America | Lactobacillaceae | Probiotic |
| GCF_015668455.1 | Lacticaseibacillus rhamnosus | USA | North America | Lactobacillaceae | Probiotic |
| GCF_028323345.1 | Lacticaseibacillus rhamnosus | USA | North America | Lactobacillaceae | Probiotic |
| GCF_028320325.1 | Lacticaseibacillus rhamnosus | USA | North America | Lactobacillaceae | Probiotic |
| GCF_028321125.1 | Lacticaseibacillus rhamnosus | USA | North America | Lactobacillaceae | Probiotic |
| GCF_028321165.1 | Lacticaseibacillus rhamnosus | USA | North America | Lactobacillaceae | Probiotic |
| GCF_028322895.1 | Lacticaseibacillus rhamnosus | USA | North America | Lactobacillaceae | Probiotic |
| GCF_028322035.1 | Lacticaseibacillus rhamnosus | USA | North America | Lactobacillaceae | Probiotic |
| GCF_015557825.1 | Lacticaseibacillus rhamnosus | USA | North America | Lactobacillaceae | Probiotic |
| GCF_028322785.1 | Lacticaseibacillus rhamnosus | USA | North America | Lactobacillaceae | Probiotic |
| GCF_028322725.1 | Lacticaseibacillus rhamnosus | USA | North America | Lactobacillaceae | Probiotic |
| GCF_028322705.1 | Lacticaseibacillus rhamnosus | USA | North America | Lactobacillaceae | Probiotic |
| GCF_028321085.1 | Lacticaseibacillus rhamnosus | USA | North America | Lactobacillaceae | Probiotic |
| GCF_028321835.1 | Lacticaseibacillus rhamnosus | USA | North America | Lactobacillaceae | Probiotic |
| GCF_015549225.1 | Lacticaseibacillus rhamnosus | USA | North America | Lactobacillaceae | Probiotic |
| GCF_006151905.1 | Lacticaseibacillus rhamnosus | China | Asia | Lactobacillaceae | Probiotic |
| GCF_900248175.1 | Lacticaseibacillus rhamnosus | Missing | Europe | Lactobacillaceae | Probiotic |
| GCF_925285305.1 | Lacticaseibacillus rhamnosus | Missing | Europe | Lactobacillaceae | Probiotic |
| GCF_925301965.1 | Lacticaseibacillus rhamnosus | Missing | Europe | Lactobacillaceae | Probiotic |
| GCF_925281885.1 | Lacticaseibacillus rhamnosus | Missing | Europe | Lactobacillaceae | Probiotic |
| GCF_925297845.1 | Lacticaseibacillus rhamnosus | Missing | Europe | Lactobacillaceae | Probiotic |
| GCF_925281845.1 | Lacticaseibacillus rhamnosus | Missing | Europe | Lactobacillaceae | Probiotic |
| GCF_900604925.1 | Lacticaseibacillus rhamnosus | Canada | North America | Lactobacillaceae | Probiotic |
| GCF_901830405.1 | Lacticaseibacillus rhamnosus | Belgium | Europe | Lactobacillaceae | Probiotic |
| GCF_902381635.1 | Lacticaseibacillus rhamnosus | Missing | Missing | Lactobacillaceae | Probiotic |
| GCF_002849515.1 | Lacticaseibacillus rhamnosus | USA | North America | Lactobacillaceae | Probiotic |
| GCF_036255815.1 | Lactiplantibacillus plantarum | China | Asia | Lactobacillaceae | Probiotic |
| GCF_027920405.1 | Lactiplantibacillus plantarum | China | Asia | Lactobacillaceae | Probiotic |
| GCF_020881935.1 | Lactiplantibacillus plantarum | China | Asia | Lactobacillaceae | Probiotic |
| GCF_001296095.1 | Lactiplantibacillus plantarum | China | Asia | Lactobacillaceae | Probiotic |
| GCF_008016415.1 | Lactiplantibacillus plantarum | Missing | Missing | Lactobacillaceae | Probiotic |
| GCF_013377705.1 | Lactiplantibacillus plantarum | China | Asia | Lactobacillaceae | Probiotic |
| GCF_000338115.2 | Lactiplantibacillus plantarum | China | Asia | Lactobacillaceae | Probiotic |
| GCF_001581895.1 | Lactiplantibacillus plantarum | China | Asia | Lactobacillaceae | Probiotic |
| GCF_003627335.1 | Lactiplantibacillus plantarum | China | Asia | Lactobacillaceae | Probiotic |
| GCF_003589725.1 | Lactiplantibacillus plantarum | China | Asia | Lactobacillaceae | Probiotic |
| GCF_029590535.1 | Lactiplantibacillus plantarum | China | Asia | Lactobacillaceae | Probiotic |
| GCF_003627355.1 | Lactiplantibacillus plantarum | China | Asia | Lactobacillaceae | Probiotic |
| GCF_015693925.1 | Lactiplantibacillus plantarum | China | Asia | Lactobacillaceae | Probiotic |
| GCF_029834415.1 | Lactiplantibacillus plantarum | China | Asia | Lactobacillaceae | Probiotic |
| GCF_030578335.1 | Lactiplantibacillus plantarum | Thailand | Asia | Lactobacillaceae | Probiotic |
| GCF_033792285.1 | Lactiplantibacillus plantarum | Madagascar | Africa | Lactobacillaceae | Probiotic |
| GCF_033792335.1 | Lactiplantibacillus plantarum | Madagascar | Africa | Lactobacillaceae | Probiotic |
| GCF_023973045.1 | Lactiplantibacillus plantarum | Belarus | Europe | Lactobacillaceae | Probiotic |
| GCF_028446625.1 | Lactiplantibacillus plantarum | China | Asia | Lactobacillaceae | Probiotic |
| GCF_030253605.1 | Lactiplantibacillus plantarum | China | Asia | Lactobacillaceae | Probiotic |
| GCF_004123035.1 | Lactiplantibacillus plantarum | China | Asia | Lactobacillaceae | Probiotic |
| GCF_004028295.1 | Lactiplantibacillus plantarum | China | Asia | Lactobacillaceae | Probiotic |
| GCF_034086125.1 | Lactiplantibacillus plantarum | Turkey | Asia | Lactobacillaceae | Probiotic |
| GCF_019311695.1 | Lactiplantibacillus plantarum | China | Asia | Lactobacillaceae | Probiotic |
| GCF_006494465.1 | Lactiplantibacillus plantarum | China | Asia | Lactobacillaceae | Probiotic |
| GCF_024391105.1 | Lactiplantibacillus plantarum | China | Asia | Lactobacillaceae | Probiotic |
| GCF_024391065.1 | Lactiplantibacillus plantarum | China | Asia | Lactobacillaceae | Probiotic |
| GCF_026156965.1 | Lactiplantibacillus plantarum | China | Asia | Lactobacillaceae | Probiotic |
| GCF_026156925.1 | Lactiplantibacillus plantarum | China | Asia | Lactobacillaceae | Probiotic |
| GCF_007833595.1 | Lactiplantibacillus plantarum | China | Asia | Lactobacillaceae | Probiotic |
| GCF_026240755.1 | Lactiplantibacillus plantarum | China | Asia | Lactobacillaceae | Probiotic |
| GCF_004301205.1 | Lactiplantibacillus plantarum | China | Asia | Lactobacillaceae | Probiotic |
| GCF_025388655.1 | Lactiplantibacillus plantarum | China | Asia | Lactobacillaceae | Probiotic |
| GCF_004301155.1 | Lactiplantibacillus plantarum | China | Asia | Lactobacillaceae | Probiotic |
| GCF_004301145.1 | Lactiplantibacillus plantarum | China | Asia | Lactobacillaceae | Probiotic |
| GCF_004301125.1 | Lactiplantibacillus plantarum | China | Asia | Lactobacillaceae | Probiotic |
| GCF_004301135.1 | Lactiplantibacillus plantarum | China | Asia | Lactobacillaceae | Probiotic |
| GCF_025388695.1 | Lactiplantibacillus plantarum | China | Asia | Lactobacillaceae | Probiotic |
| GCF_004123095.1 | Lactiplantibacillus plantarum | China | Asia | Lactobacillaceae | Probiotic |
| GCF_026156985.1 | Lactiplantibacillus plantarum | China | Asia | Lactobacillaceae | Probiotic |
| GCF_017963505.1 | Lactiplantibacillus plantarum | China | Asia | Lactobacillaceae | Probiotic |
| GCF_017963535.1 | Lactiplantibacillus plantarum | China | Asia | Lactobacillaceae | Probiotic |
| GCF_017963525.1 | Lactiplantibacillus plantarum | China | Asia | Lactobacillaceae | Probiotic |
| GCF_026156915.1 | Lactiplantibacillus plantarum | China | Asia | Lactobacillaceae | Probiotic |
| GCF_001704645.1 | Lactiplantibacillus plantarum | China | Asia | Lactobacillaceae | Probiotic |
| GCF_019076805.1 | Lactiplantibacillus plantarum | China | Asia | Lactobacillaceae | Probiotic |
| GCF_011022295.1 | Lactiplantibacillus plantarum | China | Asia | Lactobacillaceae | Probiotic |
| GCF_002943545.1 | Lactiplantibacillus plantarum | China | Asia | Lactobacillaceae | Probiotic |
| GCF_029544305.1 | Lactiplantibacillus plantarum | China | Asia | Lactobacillaceae | Probiotic |
| GCF_034555095.1 | Lactiplantibacillus plantarum | Spain | Europe | Lactobacillaceae | Probiotic |
| GCF_017580955.1 | Lactiplantibacillus plantarum | USA | North America | Lactobacillaceae | Probiotic |
| GCF_029814785.1 | Lactiplantibacillus plantarum | China | Asia | Lactobacillaceae | Probiotic |
| GCF_001331925.2 | Lactiplantibacillus plantarum | China | Asia | Lactobacillaceae | Probiotic |
| GCF_029854315.1 | Lactiplantibacillus plantarum | China | Asia | Lactobacillaceae | Probiotic |
| GCF_000474695.1 | Lactiplantibacillus plantarum | South Korea | Asia | Lactobacillaceae | Probiotic |
| GCF_001307325.1 | Lactiplantibacillus plantarum | South Korea | Asia | Lactobacillaceae | Probiotic |
| GCF_000648755.1 | Lactiplantibacillus plantarum | South Korea | Asia | Lactobacillaceae | Probiotic |
| GCF_019308385.1 | Lactiplantibacillus plantarum | South Korea | Asia | Lactobacillaceae | Probiotic |
| GCF_033055375.1 | Lactiplantibacillus plantarum | China | Asia | Lactobacillaceae | Probiotic |
| GCF_000203855.3 | Lactiplantibacillus plantarum | United Kingdom | Europe | Lactobacillaceae | Probiotic |
| GCF_021560135.1 | Lactiplantibacillus plantarum | China | Asia | Lactobacillaceae | Probiotic |
| GCF_030464425.1 | Lactiplantibacillus plantarum | China | Asia | Lactobacillaceae | Probiotic |
| GCF_030464565.1 | Lactiplantibacillus plantarum | China | Asia | Lactobacillaceae | Probiotic |
| GCF_023347215.1 | Lactiplantibacillus plantarum | China | Asia | Lactobacillaceae | Probiotic |
| GCF_028869445.1 | Lactiplantibacillus plantarum | China | Asia | Lactobacillaceae | Probiotic |
| GCF_031596315.1 | Lactiplantibacillus plantarum | China | Asia | Lactobacillaceae | Probiotic |
| GCF_031597175.1 | Lactiplantibacillus plantarum | China | Asia | Lactobacillaceae | Probiotic |
| GCF_026016545.1 | Lactiplantibacillus plantarum | China | Asia | Lactobacillaceae | Probiotic |
| GCF_024758745.1 | Lactiplantibacillus plantarum | China | Asia | Lactobacillaceae | Probiotic |
| GCF_024758665.1 | Lactiplantibacillus plantarum | China | Asia | Lactobacillaceae | Probiotic |
| GCF_036259715.1 | Lactiplantibacillus plantarum | China | Asia | Lactobacillaceae | Probiotic |
| GCF_027558615.1 | Lactiplantibacillus plantarum | China | Asia | Lactobacillaceae | Probiotic |
| GCF_026275505.1 | Lactiplantibacillus plantarum | Israel | Asia | Lactobacillaceae | Probiotic |
| GCF_004730965.1 | Lactiplantibacillus plantarum | Argentina | South America | Lactobacillaceae | Probiotic |
| GCF_030709995.1 | Lactiplantibacillus plantarum | Spain | Europe | Lactobacillaceae | Probiotic |
| GCF_030710055.1 | Lactiplantibacillus plantarum | Spain | Europe | Lactobacillaceae | Probiotic |
| GCF_030710035.1 | Lactiplantibacillus plantarum | Spain | Europe | Lactobacillaceae | Probiotic |
| GCF_030710155.1 | Lactiplantibacillus plantarum | Spain | Europe | Lactobacillaceae | Probiotic |
| GCF_030710045.1 | Lactiplantibacillus plantarum | Spain | Europe | Lactobacillaceae | Probiotic |
| GCF_030709915.1 | Lactiplantibacillus plantarum | Spain | Europe | Lactobacillaceae | Probiotic |
| GCF_030710095.1 | Lactiplantibacillus plantarum | Spain | Europe | Lactobacillaceae | Probiotic |
| GCF_000347515.1 | Lactiplantibacillus plantarum | France | Europe | Lactobacillaceae | Probiotic |
| GCF_001643065.1 | Lactiplantibacillus plantarum | Italy | Europe | Lactobacillaceae | Probiotic |
| GCF_003692725.1 | Lactiplantibacillus plantarum | India | Asia | Lactobacillaceae | Probiotic |
| GCF_011040375.1 | Lactiplantibacillus plantarum | Argentina | South America | Lactobacillaceae | Probiotic |
| GCF_029997055.1 | Lactiplantibacillus plantarum | Argentina | South America | Lactobacillaceae | Probiotic |
| GCF_001908455.1 | Lactiplantibacillus plantarum | Malaysia | Asia | Lactobacillaceae | Probiotic |
| GCF_009864015.1 | Lactiplantibacillus plantarum | Germany | Europe | Lactobacillaceae | Probiotic |
| GCF_002117285.1 | Lactiplantibacillus plantarum | Germany | Europe | Lactobacillaceae | Probiotic |
| GCF_002117265.1 | Lactiplantibacillus plantarum | Germany | Europe | Lactobacillaceae | Probiotic |
| GCF_002117245.1 | Lactiplantibacillus plantarum | Germany | Europe | Lactobacillaceae | Probiotic |
| GCF_002117305.1 | Lactiplantibacillus plantarum | Germany | Europe | Lactobacillaceae | Probiotic |
| GCF_003345375.1 | Lactiplantibacillus plantarum | Germany | Europe | Lactobacillaceae | Probiotic |
| GCF_009619495.1 | Lactiplantibacillus plantarum | Germany | Europe | Lactobacillaceae | Probiotic |
| GCF_001675425.1 | Lactiplantibacillus plantarum | Japan | Asia | Lactobacillaceae | Probiotic |
| GCF_015377525.1 | Lactiplantibacillus plantarum | China | Asia | Lactobacillaceae | Probiotic |
| GCF_003545985.1 | Lactiplantibacillus plantarum | Tajikistan | Asia | Lactobacillaceae | Probiotic |
| GCF_001888725.1 | Lactiplantibacillus plantarum | Thailand | Asia | Lactobacillaceae | Probiotic |
| GCF_001005695.1 | Lactiplantibacillus plantarum | France | Europe | Lactobacillaceae | Probiotic |
| GCF_013305265.1 | Lactiplantibacillus plantarum | Taiwan | Asia | Lactobacillaceae | Probiotic |
| GCF_033055425.1 | Lactiplantibacillus plantarum | China | Asia | Lactobacillaceae | Probiotic |
| GCF_004102845.1 | Lactiplantibacillus plantarum | China | Asia | Lactobacillaceae | Probiotic |
| GCF_004212195.1 | Lactiplantibacillus plantarum | China | Asia | Lactobacillaceae | Probiotic |
| GCF_022568865.1 | Lactiplantibacillus plantarum | Italy | Europe | Lactobacillaceae | Probiotic |
| GCF_022568895.1 | Lactiplantibacillus plantarum | Italy | Europe | Lactobacillaceae | Probiotic |
| GCF_022568855.1 | Lactiplantibacillus plantarum | Italy | Europe | Lactobacillaceae | Probiotic |
| GCF_023111055.1 | Lactiplantibacillus plantarum | Indonesia | Asia | Lactobacillaceae | Probiotic |
| GCF_018993325.1 | Lactiplantibacillus plantarum | USA | North America | Lactobacillaceae | Probiotic |
| GCF_001888275.1 | Lactiplantibacillus plantarum | Thailand | Asia | Lactobacillaceae | Probiotic |
| GCF_028895795.1 | Lactiplantibacillus plantarum | Switzerland | Europe | Lactobacillaceae | Probiotic |
| GCF_028895825.1 | Lactiplantibacillus plantarum | Switzerland | Europe | Lactobacillaceae | Probiotic |
| GCF_028895425.1 | Lactiplantibacillus plantarum | Switzerland | Europe | Lactobacillaceae | Probiotic |
| GCF_028895505.1 | Lactiplantibacillus plantarum | Switzerland | Europe | Lactobacillaceae | Probiotic |
| GCF_028895485.1 | Lactiplantibacillus plantarum | Switzerland | Europe | Lactobacillaceae | Probiotic |
| GCF_028895585.1 | Lactiplantibacillus plantarum | Switzerland | Europe | Lactobacillaceae | Probiotic |
| GCF_028895765.1 | Lactiplantibacillus plantarum | Switzerland | Europe | Lactobacillaceae | Probiotic |
| GCF_028895555.1 | Lactiplantibacillus plantarum | Switzerland | Europe | Lactobacillaceae | Probiotic |
| GCF_028895525.1 | Lactiplantibacillus plantarum | Switzerland | Europe | Lactobacillaceae | Probiotic |
| GCF_028895745.1 | Lactiplantibacillus plantarum | Switzerland | Europe | Lactobacillaceae | Probiotic |
| GCF_028895685.1 | Lactiplantibacillus plantarum | Switzerland | Europe | Lactobacillaceae | Probiotic |
| GCF_028895885.1 | Lactiplantibacillus plantarum | Switzerland | Europe | Lactobacillaceae | Probiotic |
| GCF_028895545.1 | Lactiplantibacillus plantarum | Switzerland | Europe | Lactobacillaceae | Probiotic |
| GCF_028895665.1 | Lactiplantibacillus plantarum | Switzerland | Europe | Lactobacillaceae | Probiotic |
| GCF_028895435.1 | Lactiplantibacillus plantarum | Switzerland | Europe | Lactobacillaceae | Probiotic |
| GCF_028895445.1 | Lactiplantibacillus plantarum | Switzerland | Europe | Lactobacillaceae | Probiotic |
| GCF_028895845.1 | Lactiplantibacillus plantarum | Switzerland | Europe | Lactobacillaceae | Probiotic |
| GCF_028895645.1 | Lactiplantibacillus plantarum | Switzerland | Europe | Lactobacillaceae | Probiotic |
| GCF_028895905.1 | Lactiplantibacillus plantarum | Switzerland | Europe | Lactobacillaceae | Probiotic |
| GCF_028895945.1 | Lactiplantibacillus plantarum | Switzerland | Europe | Lactobacillaceae | Probiotic |
| GCF_028895925.1 | Lactiplantibacillus plantarum | Switzerland | Europe | Lactobacillaceae | Probiotic |
| GCF_028895855.1 | Lactiplantibacillus plantarum | Switzerland | Europe | Lactobacillaceae | Probiotic |
| GCF_028895595.1 | Lactiplantibacillus plantarum | Switzerland | Europe | Lactobacillaceae | Probiotic |
| GCF_028895705.1 | Lactiplantibacillus plantarum | Switzerland | Europe | Lactobacillaceae | Probiotic |
| GCF_028895785.1 | Lactiplantibacillus plantarum | Switzerland | Europe | Lactobacillaceae | Probiotic |
| GCF_028895965.1 | Lactiplantibacillus plantarum | Switzerland | Europe | Lactobacillaceae | Probiotic |
| GCF_028895625.1 | Lactiplantibacillus plantarum | Switzerland | Europe | Lactobacillaceae | Probiotic |
| GCF_031877725.1 | Lactiplantibacillus plantarum | China | Asia | Lactobacillaceae | Probiotic |
| GCF_031876755.1 | Lactiplantibacillus plantarum | China | Asia | Lactobacillaceae | Probiotic |
| GCF_000148815.2 | Lactiplantibacillus plantarum | China | Asia | Lactobacillaceae | Probiotic |
| GCF_022558425.1 | Lactiplantibacillus plantarum | China | Asia | Lactobacillaceae | Probiotic |
| GCF_030061985.1 | Lactiplantibacillus plantarum | China | Asia | Lactobacillaceae | Probiotic |
| GCF_033055385.1 | Lactiplantibacillus plantarum | China | Asia | Lactobacillaceae | Probiotic |
| GCF_024970165.1 | Lactiplantibacillus plantarum | South Korea | Asia | Lactobacillaceae | Probiotic |
| GCF_024970145.1 | Lactiplantibacillus plantarum | South Korea | Asia | Lactobacillaceae | Probiotic |
| GCF_024970125.1 | Lactiplantibacillus plantarum | South Korea | Asia | Lactobacillaceae | Probiotic |
| GCF_024969715.1 | Lactiplantibacillus plantarum | South Korea | Asia | Lactobacillaceae | Probiotic |
| GCF_024969905.1 | Lactiplantibacillus plantarum | South Korea | Asia | Lactobacillaceae | Probiotic |
| GCF_024800605.1 | Lactiplantibacillus plantarum | South Korea | Asia | Lactobacillaceae | Probiotic |
| GCF_025245865.1 | Lactiplantibacillus plantarum | South Korea | Asia | Lactobacillaceae | Probiotic |
| GCF_004103515.1 | Lactiplantibacillus plantarum | South Korea | Asia | Lactobacillaceae | Probiotic |
| GCF_004103495.1 | Lactiplantibacillus plantarum | South Korea | Asia | Lactobacillaceae | Probiotic |
| GCF_004101645.1 | Lactiplantibacillus plantarum | South Korea | Asia | Lactobacillaceae | Probiotic |
| GCF_004101625.1 | Lactiplantibacillus plantarum | South Korea | Asia | Lactobacillaceae | Probiotic |
| GCF_004078645.1 | Lactiplantibacillus plantarum | South Korea | Asia | Lactobacillaceae | Probiotic |
| GCF_004078535.1 | Lactiplantibacillus plantarum | South Korea | Asia | Lactobacillaceae | Probiotic |
| GCF_004101605.1 | Lactiplantibacillus plantarum | South Korea | Asia | Lactobacillaceae | Probiotic |
| GCF_004101545.1 | Lactiplantibacillus plantarum | South Korea | Asia | Lactobacillaceae | Probiotic |
| GCF_004101505.1 | Lactiplantibacillus plantarum | South Korea | Asia | Lactobacillaceae | Probiotic |
| GCF_004101325.1 | Lactiplantibacillus plantarum | South Korea | Asia | Lactobacillaceae | Probiotic |
| GCF_004141895.1 | Lactiplantibacillus plantarum | South Korea | Asia | Lactobacillaceae | Probiotic |
| GCF_004141875.1 | Lactiplantibacillus plantarum | South Korea | Asia | Lactobacillaceae | Probiotic |
| GCF_004141755.1 | Lactiplantibacillus plantarum | South Korea | Asia | Lactobacillaceae | Probiotic |
| GCF_004087995.1 | Lactiplantibacillus plantarum | South Korea | Asia | Lactobacillaceae | Probiotic |
| GCF_004054305.1 | Lactiplantibacillus plantarum | South Korea | Asia | Lactobacillaceae | Probiotic |
| GCF_026976315.1 | Lactiplantibacillus plantarum | South Korea | Asia | Lactobacillaceae | Probiotic |
| GCF_002872355.1 | Lactiplantibacillus plantarum | South Korea | Asia | Lactobacillaceae | Probiotic |
| GCF_009913855.1 | Lactiplantibacillus plantarum | South Korea | Asia | Lactobacillaceae | Probiotic |
| GCF_009937825.1 | Lactiplantibacillus plantarum | South Korea | Asia | Lactobacillaceae | Probiotic |
| GCF_009913835.1 | Lactiplantibacillus plantarum | South Korea | Asia | Lactobacillaceae | Probiotic |
| GCF_009914095.1 | Lactiplantibacillus plantarum | South Korea | Asia | Lactobacillaceae | Probiotic |
| GCF_009913695.1 | Lactiplantibacillus plantarum | South Korea | Asia | Lactobacillaceae | Probiotic |
| GCF_001662895.1 | Lactiplantibacillus plantarum | South Korea | Asia | Lactobacillaceae | Probiotic |
| GCF_009913675.1 | Lactiplantibacillus plantarum | South Korea | Asia | Lactobacillaceae | Probiotic |
| GCF_009913655.1 | Lactiplantibacillus plantarum | South Korea | Asia | Lactobacillaceae | Probiotic |
| GCF_009913635.1 | Lactiplantibacillus plantarum | South Korea | Asia | Lactobacillaceae | Probiotic |
| GCF_009913615.1 | Lactiplantibacillus plantarum | South Korea | Asia | Lactobacillaceae | Probiotic |
| GCF_004055415.1 | Lactiplantibacillus plantarum | South Korea | Asia | Lactobacillaceae | Probiotic |
| GCF_004055435.1 | Lactiplantibacillus plantarum | South Korea | Asia | Lactobacillaceae | Probiotic |
| GCF_029637825.1 | Lactiplantibacillus plantarum | South Korea | Asia | Lactobacillaceae | Probiotic |
| GCF_012109355.1 | Lactiplantibacillus plantarum | South Korea | Asia | Lactobacillaceae | Probiotic |
| GCF_026421325.1 | Lactiplantibacillus plantarum | Indonesia | Asia | Lactobacillaceae | Probiotic |
| GCF_003966855.1 | Lactiplantibacillus plantarum | Japan | Asia | Lactobacillaceae | Probiotic |
| GCF_033802805.1 | Lactiplantibacillus plantarum | China | Asia | Lactobacillaceae | Probiotic |
| GCF_028768485.1 | Lactiplantibacillus plantarum | South Korea | Asia | Lactobacillaceae | Probiotic |
| GCF_004025165.1 | Lactiplantibacillus plantarum | China | Asia | Lactobacillaceae | Probiotic |
| GCF_014041895.1 | Lactiplantibacillus plantarum | South Korea | Asia | Lactobacillaceae | Probiotic |
| GCF_003269405.1 | Lactiplantibacillus plantarum | South Korea | Asia | Lactobacillaceae | Probiotic |
| GCF_009914805.1 | Lactiplantibacillus plantarum | Croatia | Europe | Lactobacillaceae | Probiotic |
| GCF_002532125.1 | Lactiplantibacillus plantarum | Croatia | Europe | Lactobacillaceae | Probiotic |
| GCF_035966695.1 | Lactiplantibacillus plantarum | Pakistan | Asia | Lactobacillaceae | Probiotic |
| GCF_022832515.1 | Lactiplantibacillus plantarum | China | Asia | Lactobacillaceae | Probiotic |
| GCF_022810685.1 | Lactiplantibacillus plantarum | China | Asia | Lactobacillaceae | Probiotic |
| GCF_008016855.1 | Lactiplantibacillus plantarum | Spain | Europe | Lactobacillaceae | Probiotic |
| GCF_016775685.1 | Lactiplantibacillus plantarum | China | Asia | Lactobacillaceae | Probiotic |
| GCF_002165655.1 | Lactiplantibacillus plantarum | Italy | Europe | Lactobacillaceae | Probiotic |
| GCF_026156845.1 | Lactiplantibacillus plantarum | China | Asia | Lactobacillaceae | Probiotic |
| GCF_026156855.1 | Lactiplantibacillus plantarum | China | Asia | Lactobacillaceae | Probiotic |
| GCF_026156885.1 | Lactiplantibacillus plantarum | China | Asia | Lactobacillaceae | Probiotic |
| GCF_002165725.1 | Lactiplantibacillus plantarum | Italy | Europe | Lactobacillaceae | Probiotic |
| GCF_030758995.1 | Lactiplantibacillus plantarum | China | Asia | Lactobacillaceae | Probiotic |
| GCF_032911485.1 | Lactiplantibacillus plantarum | China | Asia | Lactobacillaceae | Probiotic |
| GCF_001982265.1 | Lactiplantibacillus plantarum | Missing | Europe | Lactobacillaceae | Probiotic |
| GCF_001982405.1 | Lactiplantibacillus plantarum | Missing | Europe | Lactobacillaceae | Probiotic |
| GCF_001982385.1 | Lactiplantibacillus plantarum | Missing | Europe | Lactobacillaceae | Probiotic |
| GCF_001982245.1 | Lactiplantibacillus plantarum | Missing | Europe | Lactobacillaceae | Probiotic |
| GCF_001982205.1 | Lactiplantibacillus plantarum | Missing | Europe | Lactobacillaceae | Probiotic |
| GCF_001982195.1 | Lactiplantibacillus plantarum | Missing | Europe | Lactobacillaceae | Probiotic |
| GCF_001982335.1 | Lactiplantibacillus plantarum | Missing | Europe | Lactobacillaceae | Probiotic |
| GCF_001982165.1 | Lactiplantibacillus plantarum | Missing | Europe | Lactobacillaceae | Probiotic |
| GCF_001982115.1 | Lactiplantibacillus plantarum | Missing | Europe | Lactobacillaceae | Probiotic |
| GCF_001982345.1 | Lactiplantibacillus plantarum | Missing | Europe | Lactobacillaceae | Probiotic |
| GCF_001981875.1 | Lactiplantibacillus plantarum | Missing | Europe | Lactobacillaceae | Probiotic |
| GCF_001981955.1 | Lactiplantibacillus plantarum | Missing | Europe | Lactobacillaceae | Probiotic |
| GCF_001981785.1 | Lactiplantibacillus plantarum | Missing | Europe | Lactobacillaceae | Probiotic |
| GCF_002749875.1 | Lactiplantibacillus plantarum | Missing | Europe | Lactobacillaceae | Probiotic |
| GCF_001982125.1 | Lactiplantibacillus plantarum | Missing | Europe | Lactobacillaceae | Probiotic |
| GCF_002750695.1 | Lactiplantibacillus plantarum | Missing | Europe | Lactobacillaceae | Probiotic |
| GCF_001981865.1 | Lactiplantibacillus plantarum | Missing | Europe | Lactobacillaceae | Probiotic |
| GCF_002750675.1 | Lactiplantibacillus plantarum | Missing | Europe | Lactobacillaceae | Probiotic |
| GCF_002750575.1 | Lactiplantibacillus plantarum | Missing | Europe | Lactobacillaceae | Probiotic |
| GCF_001981655.1 | Lactiplantibacillus plantarum | Missing | Europe | Lactobacillaceae | Probiotic |
| GCF_001981665.1 | Lactiplantibacillus plantarum | Missing | Europe | Lactobacillaceae | Probiotic |
| GCF_001981575.1 | Lactiplantibacillus plantarum | Missing | Europe | Lactobacillaceae | Probiotic |
| GCF_001981565.1 | Lactiplantibacillus plantarum | Missing | Europe | Lactobacillaceae | Probiotic |
| GCF_001982325.1 | Lactiplantibacillus plantarum | Missing | Europe | Lactobacillaceae | Probiotic |
| GCF_001982305.1 | Lactiplantibacillus plantarum | Missing | Europe | Lactobacillaceae | Probiotic |
| GCF_001982035.1 | Lactiplantibacillus plantarum | Missing | Europe | Lactobacillaceae | Probiotic |
| GCF_001982285.1 | Lactiplantibacillus plantarum | Missing | Europe | Lactobacillaceae | Probiotic |
| GCF_001982005.1 | Lactiplantibacillus plantarum | Missing | Europe | Lactobacillaceae | Probiotic |
| GCF_001990145.1 | Lactiplantibacillus plantarum | Missing | Europe | Lactobacillaceae | Probiotic |
| GCF_001981985.1 | Lactiplantibacillus plantarum | Missing | Europe | Lactobacillaceae | Probiotic |
| GCF_001981585.1 | Lactiplantibacillus plantarum | Missing | Europe | Lactobacillaceae | Probiotic |
| GCF_002751765.1 | Lactiplantibacillus plantarum | Missing | Europe | Lactobacillaceae | Probiotic |
| GCF_001981595.1 | Lactiplantibacillus plantarum | Missing | Europe | Lactobacillaceae | Probiotic |
| GCF_001981645.1 | Lactiplantibacillus plantarum | Missing | Europe | Lactobacillaceae | Probiotic |
| GCF_020552075.1 | Lactiplantibacillus plantarum | China | Asia | Lactobacillaceae | Probiotic |
| GCF_020552045.1 | Lactiplantibacillus plantarum | China | Asia | Lactobacillaceae | Probiotic |
| GCF_020552095.1 | Lactiplantibacillus plantarum | China | Asia | Lactobacillaceae | Probiotic |
| GCF_020552065.1 | Lactiplantibacillus plantarum | China | Asia | Lactobacillaceae | Probiotic |
| GCF_004302625.1 | Lactiplantibacillus plantarum | China | Asia | Lactobacillaceae | Probiotic |
| GCF_004302615.1 | Lactiplantibacillus plantarum | China | Asia | Lactobacillaceae | Probiotic |
| GCF_017654565.1 | Lactiplantibacillus plantarum | China | Asia | Lactobacillaceae | Probiotic |
| GCF_004302585.1 | Lactiplantibacillus plantarum | China | Asia | Lactobacillaceae | Probiotic |
| GCF_004302535.1 | Lactiplantibacillus plantarum | China | Asia | Lactobacillaceae | Probiotic |
| GCF_004302515.1 | Lactiplantibacillus plantarum | China | Asia | Lactobacillaceae | Probiotic |
| GCF_004302495.1 | Lactiplantibacillus plantarum | China | Asia | Lactobacillaceae | Probiotic |
| GCF_004302485.1 | Lactiplantibacillus plantarum | China | Asia | Lactobacillaceae | Probiotic |
| GCF_020552185.1 | Lactiplantibacillus plantarum | China | Asia | Lactobacillaceae | Probiotic |
| GCF_004302505.1 | Lactiplantibacillus plantarum | China | Asia | Lactobacillaceae | Probiotic |
| GCF_004302445.1 | Lactiplantibacillus plantarum | China | Asia | Lactobacillaceae | Probiotic |
| GCF_020552215.1 | Lactiplantibacillus plantarum | China | Asia | Lactobacillaceae | Probiotic |
| GCF_004302405.1 | Lactiplantibacillus plantarum | China | Asia | Lactobacillaceae | Probiotic |
| GCF_004302415.1 | Lactiplantibacillus plantarum | China | Asia | Lactobacillaceae | Probiotic |
| GCF_004302395.1 | Lactiplantibacillus plantarum | China | Asia | Lactobacillaceae | Probiotic |
| GCF_004302385.1 | Lactiplantibacillus plantarum | China | Asia | Lactobacillaceae | Probiotic |
| GCF_004302345.1 | Lactiplantibacillus plantarum | China | Asia | Lactobacillaceae | Probiotic |
| GCF_004302315.1 | Lactiplantibacillus plantarum | China | Asia | Lactobacillaceae | Probiotic |
| GCF_020552235.1 | Lactiplantibacillus plantarum | China | Asia | Lactobacillaceae | Probiotic |
| GCF_020552295.1 | Lactiplantibacillus plantarum | China | Asia | Lactobacillaceae | Probiotic |
| GCF_020552285.1 | Lactiplantibacillus plantarum | China | Asia | Lactobacillaceae | Probiotic |
| GCF_004302285.1 | Lactiplantibacillus plantarum | China | Asia | Lactobacillaceae | Probiotic |
| GCF_004302305.1 | Lactiplantibacillus plantarum | China | Asia | Lactobacillaceae | Probiotic |
| GCF_004302295.1 | Lactiplantibacillus plantarum | China | Asia | Lactobacillaceae | Probiotic |
| GCF_004302255.1 | Lactiplantibacillus plantarum | China | Asia | Lactobacillaceae | Probiotic |
| GCF_004302205.1 | Lactiplantibacillus plantarum | China | Asia | Lactobacillaceae | Probiotic |
| GCF_020552325.1 | Lactiplantibacillus plantarum | China | Asia | Lactobacillaceae | Probiotic |
| GCF_020552405.1 | Lactiplantibacillus plantarum | China | Asia | Lactobacillaceae | Probiotic |
| GCF_004302215.1 | Lactiplantibacillus plantarum | China | Asia | Lactobacillaceae | Probiotic |
| GCF_004302195.1 | Lactiplantibacillus plantarum | China | Asia | Lactobacillaceae | Probiotic |
| GCF_004302185.1 | Lactiplantibacillus plantarum | China | Asia | Lactobacillaceae | Probiotic |
| GCF_004302135.1 | Lactiplantibacillus plantarum | China | Asia | Lactobacillaceae | Probiotic |
| GCF_004302125.1 | Lactiplantibacillus plantarum | China | Asia | Lactobacillaceae | Probiotic |
| GCF_004302115.1 | Lactiplantibacillus plantarum | China | Asia | Lactobacillaceae | Probiotic |
| GCF_004302105.1 | Lactiplantibacillus plantarum | China | Asia | Lactobacillaceae | Probiotic |
| GCF_004302065.1 | Lactiplantibacillus plantarum | China | Asia | Lactobacillaceae | Probiotic |
| GCF_004302055.1 | Lactiplantibacillus plantarum | China | Asia | Lactobacillaceae | Probiotic |
| GCF_004302035.1 | Lactiplantibacillus plantarum | China | Asia | Lactobacillaceae | Probiotic |
| GCF_034929385.1 | Lactiplantibacillus plantarum | China | Asia | Lactobacillaceae | Probiotic |
| GCF_004302005.1 | Lactiplantibacillus plantarum | China | Asia | Lactobacillaceae | Probiotic |
| GCF_004301995.1 | Lactiplantibacillus plantarum | China | Asia | Lactobacillaceae | Probiotic |
| GCF_004302715.1 | Lactiplantibacillus plantarum | China | Asia | Lactobacillaceae | Probiotic |
| GCF_004302745.1 | Lactiplantibacillus plantarum | China | Asia | Lactobacillaceae | Probiotic |
| GCF_004302725.1 | Lactiplantibacillus plantarum | China | Asia | Lactobacillaceae | Probiotic |
| GCF_004302695.1 | Lactiplantibacillus plantarum | China | Asia | Lactobacillaceae | Probiotic |
| GCF_004302685.1 | Lactiplantibacillus plantarum | China | Asia | Lactobacillaceae | Probiotic |
| GCF_004302645.1 | Lactiplantibacillus plantarum | China | Asia | Lactobacillaceae | Probiotic |
| GCF_004301975.1 | Lactiplantibacillus plantarum | China | Asia | Lactobacillaceae | Probiotic |
| GCF_004301955.1 | Lactiplantibacillus plantarum | China | Asia | Lactobacillaceae | Probiotic |
| GCF_004301905.1 | Lactiplantibacillus plantarum | China | Asia | Lactobacillaceae | Probiotic |
| GCF_004301915.1 | Lactiplantibacillus plantarum | China | Asia | Lactobacillaceae | Probiotic |
| GCF_004301885.1 | Lactiplantibacillus plantarum | China | Asia | Lactobacillaceae | Probiotic |
| GCF_004301875.1 | Lactiplantibacillus plantarum | China | Asia | Lactobacillaceae | Probiotic |
| GCF_020552425.1 | Lactiplantibacillus plantarum | China | Asia | Lactobacillaceae | Probiotic |
| GCF_020552435.1 | Lactiplantibacillus plantarum | China | Asia | Lactobacillaceae | Probiotic |
| GCF_020552445.1 | Lactiplantibacillus plantarum | China | Asia | Lactobacillaceae | Probiotic |
| GCF_026156805.1 | Lactiplantibacillus plantarum | China | Asia | Lactobacillaceae | Probiotic |
| GCF_019038995.1 | Lactiplantibacillus plantarum | China | Asia | Lactobacillaceae | Probiotic |
| GCF_003999605.1 | Lactiplantibacillus plantarum | China | Asia | Lactobacillaceae | Probiotic |
| GCF_024732385.1 | Lactiplantibacillus plantarum | South Korea | Asia | Lactobacillaceae | Probiotic |
| GCF_001005805.1 | Lactiplantibacillus plantarum | Taiwan | Asia | Lactobacillaceae | Probiotic |
| GCF_033194695.1 | Lactiplantibacillus plantarum | China | Asia | Lactobacillaceae | Probiotic |
| GCF_014840995.1 | Lactiplantibacillus plantarum | South Korea | Asia | Lactobacillaceae | Probiotic |
| GCF_003076435.1 | Lactiplantibacillus plantarum | China | Asia | Lactobacillaceae | Probiotic |
| GCF_026156815.1 | Lactiplantibacillus plantarum | China | Asia | Lactobacillaceae | Probiotic |
| GCF_026156785.1 | Lactiplantibacillus plantarum | China | Asia | Lactobacillaceae | Probiotic |
| GCF_026156765.1 | Lactiplantibacillus plantarum | China | Asia | Lactobacillaceae | Probiotic |
| GCF_003046075.1 | Lactiplantibacillus plantarum | Turkey | Asia | Lactobacillaceae | Probiotic |
| GCF_030464405.1 | Lactiplantibacillus plantarum | China | Asia | Lactobacillaceae | Probiotic |
| GCF_030464575.1 | Lactiplantibacillus plantarum | China | Asia | Lactobacillaceae | Probiotic |
| GCF_030464605.1 | Lactiplantibacillus plantarum | China | Asia | Lactobacillaceae | Probiotic |
| GCF_030464625.1 | Lactiplantibacillus plantarum | China | Asia | Lactobacillaceae | Probiotic |
| GCF_030464645.1 | Lactiplantibacillus plantarum | China | Asia | Lactobacillaceae | Probiotic |
| GCF_030464665.1 | Lactiplantibacillus plantarum | China | Asia | Lactobacillaceae | Probiotic |
| GCF_030464685.1 | Lactiplantibacillus plantarum | China | Asia | Lactobacillaceae | Probiotic |
| GCF_030464705.1 | Lactiplantibacillus plantarum | China | Asia | Lactobacillaceae | Probiotic |
| GCF_001704595.1 | Lactiplantibacillus plantarum | China | Asia | Lactobacillaceae | Probiotic |
| GCF_002576835.1 | Lactiplantibacillus plantarum | China | Asia | Lactobacillaceae | Probiotic |
| GCF_016598735.1 | Lactiplantibacillus plantarum | China | Asia | Lactobacillaceae | Probiotic |
| GCF_016066915.1 | Lactiplantibacillus plantarum | China | Asia | Lactobacillaceae | Probiotic |
| GCF_006770485.1 | Lactiplantibacillus plantarum | China | Asia | Lactobacillaceae | Probiotic |
| GCF_023507555.1 | Lactiplantibacillus plantarum | China | Asia | Lactobacillaceae | Probiotic |
| GCF_030736815.1 | Lactiplantibacillus plantarum | Malaysia | Asia | Lactobacillaceae | Probiotic |
| GCF_021559675.1 | Lactiplantibacillus plantarum | China | Asia | Lactobacillaceae | Probiotic |
| GCF_024181685.1 | Lactiplantibacillus plantarum | China | Asia | Lactobacillaceae | Probiotic |
| GCF_027585265.1 | Lactiplantibacillus plantarum | China | Asia | Lactobacillaceae | Probiotic |
| GCF_018138245.1 | Lactiplantibacillus plantarum | China | Asia | Lactobacillaceae | Probiotic |
| GCF_027585225.1 | Lactiplantibacillus plantarum | China | Asia | Lactobacillaceae | Probiotic |
| GCF_018138185.1 | Lactiplantibacillus plantarum | China | Asia | Lactobacillaceae | Probiotic |
| GCF_027585215.1 | Lactiplantibacillus plantarum | China | Asia | Lactobacillaceae | Probiotic |
| GCF_018138265.1 | Lactiplantibacillus plantarum | China | Asia | Lactobacillaceae | Probiotic |
| GCF_027585195.1 | Lactiplantibacillus plantarum | China | Asia | Lactobacillaceae | Probiotic |
| GCF_018138205.1 | Lactiplantibacillus plantarum | China | Asia | Lactobacillaceae | Probiotic |
| GCF_027585395.1 | Lactiplantibacillus plantarum | China | Asia | Lactobacillaceae | Probiotic |
| GCF_018138285.1 | Lactiplantibacillus plantarum | China | Asia | Lactobacillaceae | Probiotic |
| GCF_027585315.1 | Lactiplantibacillus plantarum | China | Asia | Lactobacillaceae | Probiotic |
| GCF_018138275.1 | Lactiplantibacillus plantarum | China | Asia | Lactobacillaceae | Probiotic |
| GCF_027585285.1 | Lactiplantibacillus plantarum | China | Asia | Lactobacillaceae | Probiotic |
| GCF_018138165.1 | Lactiplantibacillus plantarum | China | Asia | Lactobacillaceae | Probiotic |
| GCF_027585245.1 | Lactiplantibacillus plantarum | China | Asia | Lactobacillaceae | Probiotic |
| GCF_018138175.1 | Lactiplantibacillus plantarum | China | Asia | Lactobacillaceae | Probiotic |
| GCF_031082525.1 | Lactiplantibacillus plantarum | China | Asia | Lactobacillaceae | Probiotic |
| GCF_031082545.1 | Lactiplantibacillus plantarum | China | Asia | Lactobacillaceae | Probiotic |
| GCF_031082685.1 | Lactiplantibacillus plantarum | China | Asia | Lactobacillaceae | Probiotic |
| GCF_031082555.1 | Lactiplantibacillus plantarum | China | Asia | Lactobacillaceae | Probiotic |
| GCF_031082575.1 | Lactiplantibacillus plantarum | China | Asia | Lactobacillaceae | Probiotic |
| GCF_031082635.1 | Lactiplantibacillus plantarum | China | Asia | Lactobacillaceae | Probiotic |
| GCF_031082645.1 | Lactiplantibacillus plantarum | China | Asia | Lactobacillaceae | Probiotic |
| GCF_031082615.1 | Lactiplantibacillus plantarum | China | Asia | Lactobacillaceae | Probiotic |
| GCF_031082735.1 | Lactiplantibacillus plantarum | China | Asia | Lactobacillaceae | Probiotic |
| GCF_031082465.1 | Lactiplantibacillus plantarum | China | Asia | Lactobacillaceae | Probiotic |
| GCF_031082485.1 | Lactiplantibacillus plantarum | China | Asia | Lactobacillaceae | Probiotic |
| GCF_031082605.1 | Lactiplantibacillus plantarum | China | Asia | Lactobacillaceae | Probiotic |
| GCF_031082705.1 | Lactiplantibacillus plantarum | China | Asia | Lactobacillaceae | Probiotic |
| GCF_031082715.1 | Lactiplantibacillus plantarum | China | Asia | Lactobacillaceae | Probiotic |
| GCF_031082765.1 | Lactiplantibacillus plantarum | China | Asia | Lactobacillaceae | Probiotic |
| GCF_001888415.1 | Lactiplantibacillus plantarum | Thailand | Asia | Lactobacillaceae | Probiotic |
| GCF_001888465.1 | Lactiplantibacillus plantarum | Thailand | Asia | Lactobacillaceae | Probiotic |
| GCF_001888425.1 | Lactiplantibacillus plantarum | Thailand | Asia | Lactobacillaceae | Probiotic |
| GCF_001888405.1 | Lactiplantibacillus plantarum | Thailand | Asia | Lactobacillaceae | Probiotic |
| GCF_001888355.1 | Lactiplantibacillus plantarum | Thailand | Asia | Lactobacillaceae | Probiotic |
| GCF_028201575.1 | Lactiplantibacillus plantarum | China | Asia | Lactobacillaceae | Probiotic |
| GCF_032463585.1 | Lactiplantibacillus plantarum | Mexico | North America | Lactobacillaceae | Probiotic |
| GCF_032463565.1 | Lactiplantibacillus plantarum | Mexico | North America | Lactobacillaceae | Probiotic |
| GCF_001888335.1 | Lactiplantibacillus plantarum | Thailand | Asia | Lactobacillaceae | Probiotic |
| GCF_001888345.1 | Lactiplantibacillus plantarum | Thailand | Asia | Lactobacillaceae | Probiotic |
| GCF_001888255.1 | Lactiplantibacillus plantarum | Thailand | Asia | Lactobacillaceae | Probiotic |
| GCF_001888775.1 | Lactiplantibacillus plantarum | Thailand | Asia | Lactobacillaceae | Probiotic |
| GCF_027691765.1 | Lactiplantibacillus plantarum | China | Asia | Lactobacillaceae | Probiotic |
| GCF_027693035.1 | Lactiplantibacillus plantarum | China | Asia | Lactobacillaceae | Probiotic |
| GCF_002165715.1 | Lactiplantibacillus plantarum | Italy | Europe | Lactobacillaceae | Probiotic |
| GCF_006364975.1 | Lactiplantibacillus plantarum | Viet Nam | Asia | Lactobacillaceae | Probiotic |
| GCF_001649985.1 | Lactiplantibacillus plantarum | India | Asia | Lactobacillaceae | Probiotic |
| GCF_020522885.1 | Lactiplantibacillus plantarum | Japan | Asia | Lactobacillaceae | Probiotic |
| GCF_000966475.1 | Lactiplantibacillus plantarum | China | Asia | Lactobacillaceae | Probiotic |
| GCF_001633725.1 | Lactiplantibacillus plantarum | Missing | Missing | Lactobacillaceae | Probiotic |
| GCF_001633745.1 | Lactiplantibacillus plantarum | France | Europe | Lactobacillaceae | Probiotic |
| GCF_001633765.1 | Lactiplantibacillus plantarum | France | Europe | Lactobacillaceae | Probiotic |
| GCF_001633685.1 | Lactiplantibacillus plantarum | Senegal | Africa | Lactobacillaceae | Probiotic |
| GCF_001633665.1 | Lactiplantibacillus plantarum | Viet Nam | Asia | Lactobacillaceae | Probiotic |
| GCF_001633675.1 | Lactiplantibacillus plantarum | Viet Nam | Asia | Lactobacillaceae | Probiotic |
| GCF_001308305.1 | Lactiplantibacillus plantarum | Viet Nam | Asia | Lactobacillaceae | Probiotic |
| GCF_001633645.1 | Lactiplantibacillus plantarum | Viet Nam | Asia | Lactobacillaceae | Probiotic |
| GCF_001633595.1 | Lactiplantibacillus plantarum | USA | North America | Lactobacillaceae | Probiotic |
| GCF_001633505.1 | Lactiplantibacillus plantarum | Missing | Missing | Lactobacillaceae | Probiotic |
| GCF_001633575.1 | Lactiplantibacillus plantarum | Italy | Europe | Lactobacillaceae | Probiotic |
| GCF_001633605.1 | Lactiplantibacillus plantarum | United Kingdom | Europe | Lactobacillaceae | Probiotic |
| GCF_001633545.1 | Lactiplantibacillus plantarum | Japan | Asia | Lactobacillaceae | Probiotic |
| GCF_001633565.1 | Lactiplantibacillus plantarum | Japan | Asia | Lactobacillaceae | Probiotic |
| GCF_001633455.1 | Lactiplantibacillus plantarum | New Zealand | Oceania | Lactobacillaceae | Probiotic |
| GCF_001633495.1 | Lactiplantibacillus plantarum | Italy | Europe | Lactobacillaceae | Probiotic |
| GCF_001633485.1 | Lactiplantibacillus plantarum | Italy | Europe | Lactobacillaceae | Probiotic |
| GCF_001633435.1 | Lactiplantibacillus plantarum | Italy | Europe | Lactobacillaceae | Probiotic |
| GCF_001633415.1 | Lactiplantibacillus plantarum | Japan | Asia | Lactobacillaceae | Probiotic |
| GCF_001633385.1 | Lactiplantibacillus plantarum | Missing | Missing | Lactobacillaceae | Probiotic |
| GCF_001633405.1 | Lactiplantibacillus plantarum | Viet Nam | Asia | Lactobacillaceae | Probiotic |
| GCF_001633355.1 | Lactiplantibacillus plantarum | Viet Nam | Asia | Lactobacillaceae | Probiotic |
| GCF_001633335.1 | Lactiplantibacillus plantarum | Viet Nam | Asia | Lactobacillaceae | Probiotic |
| GCF_001633325.1 | Lactiplantibacillus plantarum | Viet Nam | Asia | Lactobacillaceae | Probiotic |
| GCF_001639645.1 | Lactiplantibacillus plantarum | Viet Nam | Asia | Lactobacillaceae | Probiotic |
| GCF_001639595.1 | Lactiplantibacillus plantarum | Missing | Missing | Lactobacillaceae | Probiotic |
| GCF_001639585.1 | Lactiplantibacillus plantarum | Missing | Missing | Lactobacillaceae | Probiotic |
| GCF_001639565.1 | Lactiplantibacillus plantarum | United Kingdom | Europe | Lactobacillaceae | Probiotic |
| GCF_001639545.1 | Lactiplantibacillus plantarum | France | Europe | Lactobacillaceae | Probiotic |
| GCF_001639525.1 | Lactiplantibacillus plantarum | France | Europe | Lactobacillaceae | Probiotic |
| GCF_001639505.1 | Lactiplantibacillus plantarum | France | Europe | Lactobacillaceae | Probiotic |
| GCF_001633805.1 | Lactiplantibacillus plantarum | France | Europe | Lactobacillaceae | Probiotic |
| GCF_001639485.1 | Lactiplantibacillus plantarum | Italy | Europe | Lactobacillaceae | Probiotic |
| GCF_001639425.1 | Lactiplantibacillus plantarum | France | Europe | Lactobacillaceae | Probiotic |
| GCF_001651845.1 | Lactiplantibacillus plantarum | United Kingdom | Europe | Lactobacillaceae | Probiotic |
| GCF_002407395.1 | Lactiplantibacillus plantarum | Ireland | Europe | Lactobacillaceae | Probiotic |
| GCF_003709415.1 | Lactiplantibacillus plantarum | China | Asia | Lactobacillaceae | Probiotic |
| GCF_003952885.1 | Lactiplantibacillus plantarum | South Korea | Asia | Lactobacillaceae | Probiotic |
| GCF_003325395.1 | Lactiplantibacillus plantarum | South Korea | Asia | Lactobacillaceae | Probiotic |
| GCF_001672035.1 | Lactiplantibacillus plantarum | China | Asia | Lactobacillaceae | Probiotic |
| GCF_900618215.1 | Lactiplantibacillus plantarum | Missing | Missing | Lactobacillaceae | Probiotic |
| GCF_017798305.1 | Lactiplantibacillus plantarum | USA | North America | Lactobacillaceae | Probiotic |
| GCF_004328745.1 | Lactiplantibacillus plantarum | USA | North America | Lactobacillaceae | Probiotic |
| GCF_003611015.1 | Lactiplantibacillus plantarum | New Zealand | Oceania | Lactobacillaceae | Probiotic |
| GCF_035588615.1 | Lactiplantibacillus plantarum | South Korea | Asia | Lactobacillaceae | Probiotic |
| GCF_000247735.1 | Lactiplantibacillus plantarum | Sweden | Europe | Lactobacillaceae | Probiotic |
| GCF_001633255.1 | Lactiplantibacillus plantarum | Switzerland | Europe | Lactobacillaceae | Probiotic |
| GCF_001633775.1 | Lactiplantibacillus plantarum | Switzerland | Europe | Lactobacillaceae | Probiotic |
| GCF_030644445.1 | Lactiplantibacillus plantarum | Switzerland | Europe | Lactobacillaceae | Probiotic |
| GCF_029854335.1 | Lactiplantibacillus plantarum | China | Asia | Lactobacillaceae | Probiotic |
| GCF_028463965.1 | Lactiplantibacillus plantarum | China | Asia | Lactobacillaceae | Probiotic |
| GCF_023111075.1 | Lactiplantibacillus plantarum | Indonesia | Asia | Lactobacillaceae | Probiotic |
| GCF_032921125.1 | Lactiplantibacillus plantarum | China | Asia | Lactobacillaceae | Probiotic |
| GCF_032920365.1 | Lactiplantibacillus plantarum | China | Asia | Lactobacillaceae | Probiotic |
| GCF_019469465.1 | Lactiplantibacillus plantarum | India | Asia | Lactobacillaceae | Probiotic |
| GCF_026156905.1 | Lactiplantibacillus plantarum | China | Asia | Lactobacillaceae | Probiotic |
| GCF_023278325.1 | Lactiplantibacillus plantarum | China | Asia | Lactobacillaceae | Probiotic |
| GCF_015689055.1 | Lactiplantibacillus plantarum | Turkey | Asia | Lactobacillaceae | Probiotic |
| GCF_001888505.1 | Lactiplantibacillus plantarum | Thailand | Asia | Lactobacillaceae | Probiotic |
| GCF_001888495.1 | Lactiplantibacillus plantarum | Thailand | Asia | Lactobacillaceae | Probiotic |
| GCF_001888485.1 | Lactiplantibacillus plantarum | Thailand | Asia | Lactobacillaceae | Probiotic |
| GCF_025847695.1 | Lactiplantibacillus plantarum | China | Asia | Lactobacillaceae | Probiotic |
| GCF_025402835.1 | Lactiplantibacillus plantarum | South Korea | Asia | Lactobacillaceae | Probiotic |
| GCF_001880185.2 | Lactiplantibacillus plantarum | Norway | Europe | Lactobacillaceae | Probiotic |
| GCF_033024555.1 | Lactiplantibacillus plantarum | Brazil | South America | Lactobacillaceae | Probiotic |
| GCF_018784325.1 | Lactiplantibacillus plantarum | Ireland | Europe | Lactobacillaceae | Probiotic |
| GCF_026156745.1 | Lactiplantibacillus plantarum | China | Asia | Lactobacillaceae | Probiotic |
| GCF_026930305.1 | Lactiplantibacillus plantarum | Croatia | Europe | Lactobacillaceae | Probiotic |
| GCF_029854235.1 | Lactiplantibacillus plantarum | China | Asia | Lactobacillaceae | Probiotic |
| GCF_002532175.1 | Lactiplantibacillus plantarum | Croatia | Europe | Lactobacillaceae | Probiotic |
| GCF_018588665.2 | Lactiplantibacillus plantarum | Iran | Asia | Lactobacillaceae | Probiotic |
| GCF_023108905.1 | Lactiplantibacillus plantarum | China | Asia | Lactobacillaceae | Probiotic |
| GCF_004301075.1 | Lactiplantibacillus plantarum | China | Asia | Lactobacillaceae | Probiotic |
| GCF_004301055.1 | Lactiplantibacillus plantarum | China | Asia | Lactobacillaceae | Probiotic |
| GCF_004301025.1 | Lactiplantibacillus plantarum | China | Asia | Lactobacillaceae | Probiotic |
| GCF_004301045.1 | Lactiplantibacillus plantarum | China | Asia | Lactobacillaceae | Probiotic |
| GCF_018588605.2 | Lactiplantibacillus plantarum | Iran | Asia | Lactobacillaceae | Probiotic |
| GCF_018588615.2 | Lactiplantibacillus plantarum | Iran | Asia | Lactobacillaceae | Probiotic |
| GCF_001484005.1 | Lactiplantibacillus plantarum | China | Asia | Lactobacillaceae | Probiotic |
| GCF_001660025.1 | Lactiplantibacillus plantarum | China | Asia | Lactobacillaceae | Probiotic |
| GCF_001659745.1 | Lactiplantibacillus plantarum | China | Asia | Lactobacillaceae | Probiotic |
| GCF_001715615.1 | Lactiplantibacillus plantarum | China | Asia | Lactobacillaceae | Probiotic |
| GCF_014132175.1 | Lactiplantibacillus plantarum | Japan | Asia | Lactobacillaceae | Probiotic |
| GCF_011304595.2 | Lactiplantibacillus plantarum | Slovakia | Europe | Lactobacillaceae | Probiotic |
| GCF_037094665.1 | Lactiplantibacillus plantarum | China | Asia | Lactobacillaceae | Probiotic |
| GCF_017742875.1 | Lactiplantibacillus plantarum | South Korea | Asia | Lactobacillaceae | Probiotic |
| GCF_019879165.1 | Lactiplantibacillus plantarum | South Korea | Asia | Lactobacillaceae | Probiotic |
| GCF_009807195.1 | Lactiplantibacillus plantarum | China | Asia | Lactobacillaceae | Probiotic |
| GCF_009807205.1 | Lactiplantibacillus plantarum | China | Asia | Lactobacillaceae | Probiotic |
| GCF_028994535.1 | Lactiplantibacillus plantarum | China | Asia | Lactobacillaceae | Probiotic |
| GCF_009807215.1 | Lactiplantibacillus plantarum | China | Asia | Lactobacillaceae | Probiotic |
| GCF_003097595.1 | Lactiplantibacillus plantarum | Japan | Asia | Lactobacillaceae | Probiotic |
| GCF_024442115.1 | Lactiplantibacillus plantarum | China | Asia | Lactobacillaceae | Probiotic |
| GCF_023348525.1 | Lactiplantibacillus plantarum | Ireland | Europe | Lactobacillaceae | Probiotic |
| GCF_009889775.1 | Lactiplantibacillus plantarum | Sweden | Europe | Lactobacillaceae | Probiotic |
| GCF_002205775.2 | Lactiplantibacillus plantarum | China | Asia | Lactobacillaceae | Probiotic |
| GCF_020400695.1 | Lactiplantibacillus plantarum | Australia | Oceania | Lactobacillaceae | Probiotic |
| GCF_023348465.1 | Lactiplantibacillus plantarum | Ireland | Europe | Lactobacillaceae | Probiotic |
| GCF_030061915.1 | Lactiplantibacillus plantarum | China | Asia | Lactobacillaceae | Probiotic |
| GCF_017592585.1 | Lactiplantibacillus plantarum | China | Asia | Lactobacillaceae | Probiotic |
| GCF_023207995.1 | Lactiplantibacillus plantarum | China | Asia | Lactobacillaceae | Probiotic |
| GCF_023348385.1 | Lactiplantibacillus plantarum | Ireland | Europe | Lactobacillaceae | Probiotic |
| GCF_016029535.1 | Lactiplantibacillus plantarum | Brazil | South America | Lactobacillaceae | Probiotic |
| GCF_000473935.1 | Lactiplantibacillus plantarum | India | Asia | Lactobacillaceae | Probiotic |
| GCF_016894405.1 | Lactiplantibacillus plantarum | Nigeria | Africa | Lactobacillaceae | Probiotic |
| GCF_000731855.1 | Lactiplantibacillus plantarum | Italy | Europe | Lactobacillaceae | Probiotic |
| GCF_009889735.1 | Lactiplantibacillus plantarum | China | Asia | Lactobacillaceae | Probiotic |
| GCF_009889825.1 | Lactiplantibacillus plantarum | China | Asia | Lactobacillaceae | Probiotic |
| GCF_009889865.1 | Lactiplantibacillus plantarum | China | Asia | Lactobacillaceae | Probiotic |
| GCF_002370925.1 | Lactiplantibacillus plantarum | China | Asia | Lactobacillaceae | Probiotic |
| GCF_024181705.1 | Lactiplantibacillus plantarum | China | Asia | Lactobacillaceae | Probiotic |
| GCF_009889835.1 | Lactiplantibacillus plantarum | China | Asia | Lactobacillaceae | Probiotic |
| GCF_009889885.1 | Lactiplantibacillus plantarum | China | Asia | Lactobacillaceae | Probiotic |
| GCF_009889895.1 | Lactiplantibacillus plantarum | China | Asia | Lactobacillaceae | Probiotic |
| GCF_009889935.1 | Lactiplantibacillus plantarum | China | Asia | Lactobacillaceae | Probiotic |
| GCF_009889925.1 | Lactiplantibacillus plantarum | China | Asia | Lactobacillaceae | Probiotic |
| GCF_009889965.1 | Lactiplantibacillus plantarum | China | Asia | Lactobacillaceae | Probiotic |
| GCF_009889995.1 | Lactiplantibacillus plantarum | China | Asia | Lactobacillaceae | Probiotic |
| GCF_026127705.1 | Lactiplantibacillus plantarum | Italy | Europe | Lactobacillaceae | Probiotic |
| GCF_026127805.1 | Lactiplantibacillus plantarum | Italy | Europe | Lactobacillaceae | Probiotic |
| GCF_009889975.1 | Lactiplantibacillus plantarum | China | Asia | Lactobacillaceae | Probiotic |
| GCF_009890055.1 | Lactiplantibacillus plantarum | China | Asia | Lactobacillaceae | Probiotic |
| GCF_002286275.1 | Lactiplantibacillus plantarum | South Korea | Asia | Lactobacillaceae | Probiotic |
| GCF_026127545.1 | Lactiplantibacillus plantarum | Italy | Europe | Lactobacillaceae | Probiotic |
| GCF_009890025.1 | Lactiplantibacillus plantarum | China | Asia | Lactobacillaceae | Probiotic |
| GCF_009890045.1 | Lactiplantibacillus plantarum | China | Asia | Lactobacillaceae | Probiotic |
| GCF_002109425.1 | Lactiplantibacillus plantarum | China | Asia | Lactobacillaceae | Probiotic |
| GCF_030063125.1 | Lactiplantibacillus plantarum | China | Asia | Lactobacillaceae | Probiotic |
| GCF_001540925.1 | Lactiplantibacillus plantarum | France | Europe | Lactobacillaceae | Probiotic |
| GCF_012932405.1 | Lactiplantibacillus plantarum | USA | North America | Lactobacillaceae | Probiotic |
| GCF_012935555.1 | Lactiplantibacillus plantarum | USA | North America | Lactobacillaceae | Probiotic |
| GCF_026127665.1 | Lactiplantibacillus plantarum | Italy | Europe | Lactobacillaceae | Probiotic |
| GCF_023168025.1 | Lactiplantibacillus plantarum | Japan | Asia | Lactobacillaceae | Probiotic |
| GCF_003813125.1 | Lactiplantibacillus plantarum | South Korea | Asia | Lactobacillaceae | Probiotic |
| GCF_023195585.1 | Lactiplantibacillus plantarum | Thailand | Asia | Lactobacillaceae | Probiotic |
| GCF_030410355.1 | Lactiplantibacillus plantarum | Viet Nam | Asia | Lactobacillaceae | Probiotic |
| GCF_006770505.1 | Lactiplantibacillus plantarum | China | Asia | Lactobacillaceae | Probiotic |
| GCF_001754025.1 | Lactiplantibacillus plantarum | Spain | Europe | Lactobacillaceae | Probiotic |
| GCF_027474465.1 | Lactiplantibacillus plantarum | Spain | Europe | Lactobacillaceae | Probiotic |
| GCF_034258695.1 | Lactiplantibacillus plantarum | Algeria | Africa | Lactobacillaceae | Probiotic |
| GCF_026156725.1 | Lactiplantibacillus plantarum | China | Asia | Lactobacillaceae | Probiotic |
| GCF_013808525.1 | Lactiplantibacillus plantarum | Algeria | Africa | Lactobacillaceae | Probiotic |
| GCF_013808535.1 | Lactiplantibacillus plantarum | Algeria | Africa | Lactobacillaceae | Probiotic |
| GCF_013808505.1 | Lactiplantibacillus plantarum | Algeria | Africa | Lactobacillaceae | Probiotic |
| GCF_019641415.1 | Lactiplantibacillus plantarum | Denmark | Europe | Lactobacillaceae | Probiotic |
| GCF_022844655.1 | Lactiplantibacillus plantarum | Missing | Missing | Lactobacillaceae | Probiotic |
| GCF_002906875.1 | Lactiplantibacillus plantarum | Philippines | Asia | Lactobacillaceae | Probiotic |
| GCF_036287535.1 | Lactiplantibacillus plantarum | China | Asia | Lactobacillaceae | Probiotic |
| GCF_036431865.1 | Lactiplantibacillus plantarum | Ghana | Africa | Lactobacillaceae | Probiotic |
| GCF_019321805.1 | Lactiplantibacillus plantarum | China | Asia | Lactobacillaceae | Probiotic |
| GCF_026153115.1 | Lactiplantibacillus plantarum | Missing | Missing | Lactobacillaceae | Probiotic |
| GCF_001267905.1 | Lactiplantibacillus plantarum | China | Asia | Lactobacillaceae | Probiotic |
| GCF_036353295.2 | Lactiplantibacillus plantarum | Bulgaria | Europe | Lactobacillaceae | Probiotic |
| GCF_012070635.1 | Lactiplantibacillus plantarum | India | Asia | Lactobacillaceae | Probiotic |
| GCF_020916405.1 | Lactiplantibacillus plantarum | Greece | Europe | Lactobacillaceae | Probiotic |
| GCF_011421665.1 | Lactiplantibacillus plantarum | India | Asia | Lactobacillaceae | Probiotic |
| GCF_025631195.1 | Lactiplantibacillus plantarum | China | Asia | Lactobacillaceae | Probiotic |
| GCF_026930585.1 | Lactiplantibacillus plantarum | Croatia | Europe | Lactobacillaceae | Probiotic |
| GCF_001704315.1 | Lactiplantibacillus plantarum | Canada | North America | Lactobacillaceae | Probiotic |
| GCF_003346075.1 | Lactiplantibacillus plantarum | Slovakia | Europe | Lactobacillaceae | Probiotic |
| GCF_003346235.1 | Lactiplantibacillus plantarum | Slovakia | Europe | Lactobacillaceae | Probiotic |
| GCF_003346085.1 | Lactiplantibacillus plantarum | Slovakia | Europe | Lactobacillaceae | Probiotic |
| GCF_003346105.1 | Lactiplantibacillus plantarum | Slovakia | Europe | Lactobacillaceae | Probiotic |
| GCF_003346175.1 | Lactiplantibacillus plantarum | Slovakia | Europe | Lactobacillaceae | Probiotic |
| GCF_016838645.1 | Lactiplantibacillus plantarum | South Korea | Asia | Lactobacillaceae | Probiotic |
| GCF_017576965.1 | Lactiplantibacillus plantarum | China | Asia | Lactobacillaceae | Probiotic |
| GCF_031296365.1 | Lactiplantibacillus plantarum | Poland | Europe | Lactobacillaceae | Probiotic |
| GCF_027568335.1 | Lactiplantibacillus plantarum | Indonesia | Asia | Lactobacillaceae | Probiotic |
| GCF_029543005.1 | Lactiplantibacillus plantarum | South Korea | Asia | Lactobacillaceae | Probiotic |
| GCF_031348585.1 | Lactiplantibacillus plantarum | South Korea | Asia | Lactobacillaceae | Probiotic |
| GCF_031461015.1 | Lactiplantibacillus plantarum | South Korea | Asia | Lactobacillaceae | Probiotic |
| GCF_031461055.1 | Lactiplantibacillus plantarum | South Korea | Asia | Lactobacillaceae | Probiotic |
| GCF_009720585.1 | Lactiplantibacillus plantarum | South Korea | Asia | Lactobacillaceae | Probiotic |
| GCF_002868755.1 | Lactiplantibacillus plantarum | South Korea | Asia | Lactobacillaceae | Probiotic |
| GCF_002948215.1 | Lactiplantibacillus plantarum | South Korea | Asia | Lactobacillaceae | Probiotic |
| GCF_004000705.1 | Lactiplantibacillus plantarum | Japan | Asia | Lactobacillaceae | Probiotic |
| GCF_001742965.1 | Lactiplantibacillus plantarum | India | Asia | Lactobacillaceae | Probiotic |
| GCF_003692595.1 | Lactiplantibacillus plantarum | South Korea | Asia | Lactobacillaceae | Probiotic |
| GCF_017581025.1 | Lactiplantibacillus plantarum | Bulgaria | Europe | Lactobacillaceae | Probiotic |
| GCF_001888745.1 | Lactiplantibacillus plantarum | Thailand | Asia | Lactobacillaceae | Probiotic |
| GCF_001888265.1 | Lactiplantibacillus plantarum | Thailand | Asia | Lactobacillaceae | Probiotic |
| GCF_002868775.1 | Lactiplantibacillus plantarum | South Korea | Asia | Lactobacillaceae | Probiotic |
| GCF_003020005.1 | Lactiplantibacillus plantarum | China | Asia | Lactobacillaceae | Probiotic |
| GCF_015714775.1 | Lactiplantibacillus plantarum | Ethiopia | Africa | Lactobacillaceae | Probiotic |
| GCF_013307305.1 | Lactiplantibacillus plantarum | Chile | South America | Lactobacillaceae | Probiotic |
| GCF_019599425.1 | Lactiplantibacillus plantarum | China | Asia | Lactobacillaceae | Probiotic |
| GCF_026156705.1 | Lactiplantibacillus plantarum | China | Asia | Lactobacillaceae | Probiotic |
| GCF_001720285.1 | Lactiplantibacillus plantarum | South Korea | Asia | Lactobacillaceae | Probiotic |
| GCF_026156665.1 | Lactiplantibacillus plantarum | China | Asia | Lactobacillaceae | Probiotic |
| GCF_002920935.1 | Lactiplantibacillus plantarum | China | Asia | Lactobacillaceae | Probiotic |
| GCF_031583165.1 | Lactiplantibacillus plantarum | China | Asia | Lactobacillaceae | Probiotic |
| GCF_000023085.1 | Lactiplantibacillus plantarum | China | Asia | Lactobacillaceae | Probiotic |
| GCF_003023825.1 | Lactiplantibacillus plantarum | India | Asia | Lactobacillaceae | Probiotic |
| GCF_002109405.1 | Lactiplantibacillus plantarum | South Korea | Asia | Lactobacillaceae | Probiotic |
| GCF_001596095.1 | Lactiplantibacillus plantarum | South Korea | Asia | Lactobacillaceae | Probiotic |
| GCF_023370155.1 | Lactiplantibacillus plantarum | China | Asia | Lactobacillaceae | Probiotic |
| GCF_034426955.1 | Lactiplantibacillus plantarum | China | Asia | Lactobacillaceae | Probiotic |
| GCF_030297695.1 | Lactiplantibacillus plantarum | Japan | Asia | Lactobacillaceae | Probiotic |
| GCF_011170185.2 | Lactiplantibacillus plantarum | Japan | Asia | Lactobacillaceae | Probiotic |
| GCF_026184455.1 | Lactiplantibacillus plantarum | South Africa | Africa | Lactobacillaceae | Probiotic |
| GCF_032602185.1 | Lactiplantibacillus plantarum | Indonesia | Asia | Lactobacillaceae | Probiotic |
| GCF_004319665.1 | Lactiplantibacillus plantarum | South Korea | Asia | Lactobacillaceae | Probiotic |
| GCF_000410795.1 | Lactiplantibacillus plantarum | Italy | Europe | Lactobacillaceae | Probiotic |
| GCF_004368485.1 | Lactiplantibacillus plantarum | Norway | Europe | Lactobacillaceae | Probiotic |
| GCF_022510025.2 | Lactiplantibacillus plantarum | USA | North America | Lactobacillaceae | Probiotic |
| GCF_021536745.2 | Lactiplantibacillus plantarum | USA | North America | Lactobacillaceae | Probiotic |
| GCF_022631455.1 | Lactiplantibacillus plantarum | USA | North America | Lactobacillaceae | Probiotic |
| GCF_037113575.1 | Lactiplantibacillus plantarum | South Korea | Asia | Lactobacillaceae | Probiotic |
| GCF_036416335.1 | Lactiplantibacillus plantarum | China | Asia | Lactobacillaceae | Probiotic |
| GCF_009766195.1 | Lactiplantibacillus plantarum | China | Asia | Lactobacillaceae | Probiotic |
| GCF_009766165.1 | Lactiplantibacillus plantarum | Mongolia | Asia | Lactobacillaceae | Probiotic |
| GCF_037084175.1 | Lactiplantibacillus plantarum | France | Europe | Lactobacillaceae | Probiotic |
| GCF_003428355.1 | Lactiplantibacillus plantarum | South Korea | Asia | Lactobacillaceae | Probiotic |
| GCF_001888245.1 | Lactiplantibacillus plantarum | Thailand | Asia | Lactobacillaceae | Probiotic |
| GCF_001888565.1 | Lactiplantibacillus plantarum | Thailand | Asia | Lactobacillaceae | Probiotic |
| GCF_022713005.1 | Lactiplantibacillus plantarum | China | Asia | Lactobacillaceae | Probiotic |
| GCF_026459915.1 | Lactiplantibacillus plantarum | China | Asia | Lactobacillaceae | Probiotic |
| GCF_024396815.1 | Lactiplantibacillus plantarum | China | Asia | Lactobacillaceae | Probiotic |
| GCF_029906425.1 | Lactiplantibacillus plantarum | China | Asia | Lactobacillaceae | Probiotic |
| GCF_025144505.1 | Lactiplantibacillus plantarum | China | Asia | Lactobacillaceae | Probiotic |
| GCF_027854125.1 | Lactiplantibacillus plantarum | China | Asia | Lactobacillaceae | Probiotic |
| GCF_001302645.1 | Lactiplantibacillus plantarum | India | Asia | Lactobacillaceae | Probiotic |
| GCF_022815785.1 | Lactiplantibacillus plantarum | Pakistan | Asia | Lactobacillaceae | Probiotic |
| GCF_013367715.1 | Lactiplantibacillus plantarum | Sweden | Europe | Lactobacillaceae | Probiotic |
| GCF_013458335.1 | Lactiplantibacillus plantarum | China | Asia | Lactobacillaceae | Probiotic |
| GCF_030463605.1 | Lactiplantibacillus plantarum | China | Asia | Lactobacillaceae | Probiotic |
| GCF_003143915.1 | Lactiplantibacillus plantarum | South Korea | Asia | Lactobacillaceae | Probiotic |
| GCF_030646455.1 | Lactiplantibacillus plantarum | Iraq | Asia | Lactobacillaceae | Probiotic |
| GCF_030646105.1 | Lactiplantibacillus plantarum | Iraq | Asia | Lactobacillaceae | Probiotic |
| GCF_026156675.1 | Lactiplantibacillus plantarum | China | Asia | Lactobacillaceae | Probiotic |
| GCF_021199095.1 | Lactiplantibacillus plantarum | China | Asia | Lactobacillaceae | Probiotic |
| GCF_019211785.1 | Lactiplantibacillus plantarum | Taiwan | Asia | Lactobacillaceae | Probiotic |
| GCF_021650875.1 | Lactiplantibacillus plantarum | Taiwan | Asia | Lactobacillaceae | Probiotic |
| GCF_019211765.1 | Lactiplantibacillus plantarum | Taiwan | Asia | Lactobacillaceae | Probiotic |
| GCF_017068235.1 | Lactiplantibacillus plantarum | Taiwan | Asia | Lactobacillaceae | Probiotic |
| GCF_029910155.1 | Lactiplantibacillus plantarum | South Korea | Asia | Lactobacillaceae | Probiotic |
| GCF_018395655.2 | Lactiplantibacillus plantarum | South Korea | Asia | Lactobacillaceae | Probiotic |
| GCF_002220815.1 | Lactiplantibacillus plantarum | South Korea | Asia | Lactobacillaceae | Probiotic |
| GCF_036330065.1 | Lactiplantibacillus plantarum | South Korea | Asia | Lactobacillaceae | Probiotic |
| GCF_033802745.1 | Lactiplantibacillus plantarum | South Korea | Asia | Lactobacillaceae | Probiotic |
| GCF_015694325.1 | Lactiplantibacillus plantarum | USA | North America | Lactobacillaceae | Probiotic |
| GCF_013256965.1 | Lactiplantibacillus plantarum | Brazil | South America | Lactobacillaceae | Probiotic |
| GCF_033055465.1 | Lactiplantibacillus plantarum | China | Asia | Lactobacillaceae | Probiotic |
| GCF_004683785.1 | Lactiplantibacillus plantarum | Zimbabwe | Africa | Lactobacillaceae | Probiotic |
| GCF_009863935.1 | Lactiplantibacillus plantarum | Zimbabwe | Africa | Lactobacillaceae | Probiotic |
| GCF_020844665.1 | Lactiplantibacillus plantarum | Italy | Europe | Lactobacillaceae | Probiotic |
| GCF_026127775.1 | Lactiplantibacillus plantarum | Italy | Europe | Lactobacillaceae | Probiotic |
| GCF_026127725.1 | Lactiplantibacillus plantarum | Botswana | Africa | Lactobacillaceae | Probiotic |
| GCF_020844675.1 | Lactiplantibacillus plantarum | Botswana | Africa | Lactobacillaceae | Probiotic |
| GCF_026127635.1 | Lactiplantibacillus plantarum | Botswana | Africa | Lactobacillaceae | Probiotic |
| GCF_020844685.1 | Lactiplantibacillus plantarum | Germany | Europe | Lactobacillaceae | Probiotic |
| GCF_026127685.1 | Lactiplantibacillus plantarum | Germany | Europe | Lactobacillaceae | Probiotic |
| GCF_026127625.1 | Lactiplantibacillus plantarum | Missing | Missing | Lactobacillaceae | Probiotic |
| GCF_026127845.1 | Lactiplantibacillus plantarum | Missing | Missing | Lactobacillaceae | Probiotic |
| GCF_026127605.1 | Lactiplantibacillus plantarum | Botswana | Africa | Lactobacillaceae | Probiotic |
| GCF_026127745.1 | Lactiplantibacillus plantarum | Canada | North America | Lactobacillaceae | Probiotic |
| GCF_026127585.1 | Lactiplantibacillus plantarum | Missing | Missing | Lactobacillaceae | Probiotic |
| GCF_026127765.1 | Lactiplantibacillus plantarum | Missing | Missing | Lactobacillaceae | Probiotic |
| GCF_030464765.1 | Lactiplantibacillus plantarum | China | Asia | Lactobacillaceae | Probiotic |
| GCF_030464725.1 | Lactiplantibacillus plantarum | China | Asia | Lactobacillaceae | Probiotic |
| GCF_030466465.1 | Lactiplantibacillus plantarum | China | Asia | Lactobacillaceae | Probiotic |
| GCF_000764285.1 | Lactiplantibacillus plantarum | China | Asia | Lactobacillaceae | Probiotic |
| GCF_002737925.1 | Lactiplantibacillus plantarum | France | Europe | Lactobacillaceae | Probiotic |
| GCF_002737825.1 | Lactiplantibacillus plantarum | France | Europe | Lactobacillaceae | Probiotic |
| GCF_002738055.1 | Lactiplantibacillus plantarum | France | Europe | Lactobacillaceae | Probiotic |
| GCF_002737885.1 | Lactiplantibacillus plantarum | France | Europe | Lactobacillaceae | Probiotic |
| GCF_002737845.1 | Lactiplantibacillus plantarum | France | Europe | Lactobacillaceae | Probiotic |
| GCF_002735815.1 | Lactiplantibacillus plantarum | France | Europe | Lactobacillaceae | Probiotic |
| GCF_002737905.1 | Lactiplantibacillus plantarum | France | Europe | Lactobacillaceae | Probiotic |
| GCF_002737835.1 | Lactiplantibacillus plantarum | France | Europe | Lactobacillaceae | Probiotic |
| GCF_027557615.1 | Lactiplantibacillus plantarum | China | Asia | Lactobacillaceae | Probiotic |
| GCF_036430805.1 | Lactiplantibacillus plantarum | Ghana | Africa | Lactobacillaceae | Probiotic |
| GCF_036430845.1 | Lactiplantibacillus plantarum | Ghana | Africa | Lactobacillaceae | Probiotic |
| GCF_012689225.1 | Lactiplantibacillus plantarum | France | Europe | Lactobacillaceae | Probiotic |
| GCF_030464385.1 | Lactiplantibacillus plantarum | China | Asia | Lactobacillaceae | Probiotic |
| GCF_030464445.1 | Lactiplantibacillus plantarum | China | Asia | Lactobacillaceae | Probiotic |
| GCF_030464485.1 | Lactiplantibacillus plantarum | China | Asia | Lactobacillaceae | Probiotic |
| GCF_030464465.1 | Lactiplantibacillus plantarum | China | Asia | Lactobacillaceae | Probiotic |
| GCF_030464495.1 | Lactiplantibacillus plantarum | China | Asia | Lactobacillaceae | Probiotic |
| GCF_030464525.1 | Lactiplantibacillus plantarum | China | Asia | Lactobacillaceae | Probiotic |
| GCF_030464545.1 | Lactiplantibacillus plantarum | China | Asia | Lactobacillaceae | Probiotic |
| GCF_023145715.1 | Lactiplantibacillus plantarum | Russia | Europe | Lactobacillaceae | Probiotic |
| GCF_001619295.1 | Lactiplantibacillus plantarum | Netherlands | Europe | Lactobacillaceae | Probiotic |
| GCF_001619265.1 | Lactiplantibacillus plantarum | Netherlands | Europe | Lactobacillaceae | Probiotic |
| GCF_001619275.1 | Lactiplantibacillus plantarum | Netherlands | Europe | Lactobacillaceae | Probiotic |
| GCF_003999275.1 | Lactiplantibacillus plantarum | China | Asia | Lactobacillaceae | Probiotic |
| GCF_026156645.1 | Lactiplantibacillus plantarum | China | Asia | Lactobacillaceae | Probiotic |
| GCF_005864275.1 | Lactiplantibacillus plantarum | Switzerland | Europe | Lactobacillaceae | Probiotic |
| GCF_036353265.1 | Lactiplantibacillus plantarum | Bulgaria | Europe | Lactobacillaceae | Probiotic |
| GCF_001633245.1 | Lactiplantibacillus plantarum | France | Europe | Lactobacillaceae | Probiotic |
| GCF_032818175.1 | Lactiplantibacillus plantarum | China | Asia | Lactobacillaceae | Probiotic |
| GCF_029542245.1 | Lactiplantibacillus plantarum | China | Asia | Lactobacillaceae | Probiotic |
| GCF_008016845.1 | Lactiplantibacillus plantarum | Spain | Europe | Lactobacillaceae | Probiotic |
| GCF_004337615.1 | Lactiplantibacillus plantarum | South Korea | Asia | Lactobacillaceae | Probiotic |
| GCF_029906245.1 | Lactiplantibacillus plantarum | Lithuania | Europe | Lactobacillaceae | Probiotic |
| GCF_029846785.1 | Lactiplantibacillus plantarum | Lithuania | Europe | Lactobacillaceae | Probiotic |
| GCF_029764995.1 | Lactiplantibacillus plantarum | Lithuania | Europe | Lactobacillaceae | Probiotic |
| GCF_030403425.1 | Lactiplantibacillus plantarum | Lithuania | Europe | Lactobacillaceae | Probiotic |
| GCF_030403475.1 | Lactiplantibacillus plantarum | Lithuania | Europe | Lactobacillaceae | Probiotic |
| GCF_017580935.1 | Lactiplantibacillus plantarum | USA | North America | Lactobacillaceae | Probiotic |
| GCF_009935675.1 | Lactiplantibacillus plantarum | South Korea | Asia | Lactobacillaceae | Probiotic |
| GCF_003258615.1 | Lactiplantibacillus plantarum | South Korea | Asia | Lactobacillaceae | Probiotic |
| GCF_030575255.1 | Lactiplantibacillus plantarum | China | Asia | Lactobacillaceae | Probiotic |
| GCF_004301035.1 | Lactiplantibacillus plantarum | China | Asia | Lactobacillaceae | Probiotic |
| GCF_004300955.1 | Lactiplantibacillus plantarum | China | Asia | Lactobacillaceae | Probiotic |
| GCF_004300945.1 | Lactiplantibacillus plantarum | China | Asia | Lactobacillaceae | Probiotic |
| GCF_004300965.1 | Lactiplantibacillus plantarum | China | Asia | Lactobacillaceae | Probiotic |
| GCF_004300935.1 | Lactiplantibacillus plantarum | China | Asia | Lactobacillaceae | Probiotic |
| GCF_004300925.1 | Lactiplantibacillus plantarum | China | Asia | Lactobacillaceae | Probiotic |
| GCF_001597605.1 | Lactiplantibacillus plantarum | India | Asia | Lactobacillaceae | Probiotic |
| GCF_001596195.1 | Lactiplantibacillus plantarum | India | Asia | Lactobacillaceae | Probiotic |
| GCF_026013765.1 | Lactiplantibacillus plantarum | China | Asia | Lactobacillaceae | Probiotic |
| GCF_003429585.1 | Lactiplantibacillus plantarum | India | Asia | Lactobacillaceae | Probiotic |
| GCF_021462365.1 | Lactiplantibacillus plantarum | Turkey | Asia | Lactobacillaceae | Probiotic |
| GCF_019425695.1 | Lactiplantibacillus plantarum | Thailand | Asia | Lactobacillaceae | Probiotic |
| GCF_003286955.1 | Lactiplantibacillus plantarum | South Korea | Asia | Lactobacillaceae | Probiotic |
| GCF_020131335.1 | Lactiplantibacillus plantarum | Germany | Europe | Lactobacillaceae | Probiotic |
| GCF_001888665.1 | Lactiplantibacillus plantarum | Missing | Missing | Lactobacillaceae | Probiotic |
| GCF_003061785.1 | Lactiplantibacillus plantarum | USA | North America | Lactobacillaceae | Probiotic |
| GCF_003053025.1 | Lactiplantibacillus plantarum | USA | North America | Lactobacillaceae | Probiotic |
| GCF_003061725.1 | Lactiplantibacillus plantarum | USA | North America | Lactobacillaceae | Probiotic |
| GCF_003053165.1 | Lactiplantibacillus plantarum | USA | North America | Lactobacillaceae | Probiotic |
| GCF_003053045.1 | Lactiplantibacillus plantarum | USA | North America | Lactobacillaceae | Probiotic |
| GCF_037099845.1 | Lactiplantibacillus plantarum | South Korea | Asia | Lactobacillaceae | Probiotic |
| GCF_037100195.1 | Lactiplantibacillus plantarum | South Korea | Asia | Lactobacillaceae | Probiotic |
| GCF_003053035.1 | Lactiplantibacillus plantarum | USA | North America | Lactobacillaceae | Probiotic |
| GCF_003061765.1 | Lactiplantibacillus plantarum | USA | North America | Lactobacillaceae | Probiotic |
| GCF_003061805.1 | Lactiplantibacillus plantarum | USA | North America | Lactobacillaceae | Probiotic |
| GCF_037414485.1 | Lactiplantibacillus plantarum | South Korea | Asia | Lactobacillaceae | Probiotic |
| GCF_037099865.1 | Lactiplantibacillus plantarum | South Korea | Asia | Lactobacillaceae | Probiotic |
| GCF_023702985.1 | Lactiplantibacillus plantarum | Greece | Europe | Lactobacillaceae | Probiotic |
| GCF_023703015.1 | Lactiplantibacillus plantarum | Greece | Europe | Lactobacillaceae | Probiotic |
| GCF_023703265.1 | Lactiplantibacillus plantarum | Greece | Europe | Lactobacillaceae | Probiotic |
| GCF_023702995.1 | Lactiplantibacillus plantarum | Greece | Europe | Lactobacillaceae | Probiotic |
| GCF_023702965.1 | Lactiplantibacillus plantarum | Greece | Europe | Lactobacillaceae | Probiotic |
| GCF_023702905.1 | Lactiplantibacillus plantarum | Greece | Europe | Lactobacillaceae | Probiotic |
| GCF_023702945.1 | Lactiplantibacillus plantarum | Greece | Europe | Lactobacillaceae | Probiotic |
| GCF_023702925.1 | Lactiplantibacillus plantarum | Greece | Europe | Lactobacillaceae | Probiotic |
| GCF_023703155.1 | Lactiplantibacillus plantarum | Greece | Europe | Lactobacillaceae | Probiotic |
| GCF_023703175.1 | Lactiplantibacillus plantarum | Greece | Europe | Lactobacillaceae | Probiotic |
| GCF_023703125.1 | Lactiplantibacillus plantarum | Greece | Europe | Lactobacillaceae | Probiotic |
| GCF_023703145.1 | Lactiplantibacillus plantarum | Greece | Europe | Lactobacillaceae | Probiotic |
| GCF_023703235.1 | Lactiplantibacillus plantarum | Greece | Europe | Lactobacillaceae | Probiotic |
| GCF_023702795.1 | Lactiplantibacillus plantarum | Greece | Europe | Lactobacillaceae | Probiotic |
| GCF_023703225.1 | Lactiplantibacillus plantarum | Greece | Europe | Lactobacillaceae | Probiotic |
| GCF_023702885.1 | Lactiplantibacillus plantarum | Greece | Europe | Lactobacillaceae | Probiotic |
| GCF_003586485.1 | Lactiplantibacillus plantarum | Malaysia | Asia | Lactobacillaceae | Probiotic |
| GCF_000604105.1 | Lactiplantibacillus plantarum | Missing | Missing | Lactobacillaceae | Probiotic |
| GCF_014878225.1 | Lactiplantibacillus plantarum | India | Asia | Lactobacillaceae | Probiotic |
| GCF_012272935.1 | Lactiplantibacillus plantarum | India | Asia | Lactobacillaceae | Probiotic |
| GCF_029855105.1 | Lactiplantibacillus plantarum | Russia | Europe | Lactobacillaceae | Probiotic |
| GCF_000743895.1 | Lactiplantibacillus plantarum | USA | North America | Lactobacillaceae | Probiotic |
| GCF_018257155.1 | Lactiplantibacillus plantarum | USA | North America | Lactobacillaceae | Probiotic |
| GCF_018257075.1 | Lactiplantibacillus plantarum | USA | North America | Lactobacillaceae | Probiotic |
| GCF_018257135.1 | Lactiplantibacillus plantarum | USA | North America | Lactobacillaceae | Probiotic |
| GCF_018257115.1 | Lactiplantibacillus plantarum | USA | North America | Lactobacillaceae | Probiotic |
| GCF_018257215.1 | Lactiplantibacillus plantarum | USA | North America | Lactobacillaceae | Probiotic |
| GCF_024137845.1 | Lactiplantibacillus plantarum | South Korea | Asia | Lactobacillaceae | Probiotic |
| GCF_002220175.1 | Lactiplantibacillus plantarum | USA | North America | Lactobacillaceae | Probiotic |
| GCF_004118615.1 | Lactiplantibacillus plantarum | India | Asia | Lactobacillaceae | Probiotic |
| GCF_002737995.1 | Lactiplantibacillus plantarum | France | Europe | Lactobacillaceae | Probiotic |
| GCF_002737935.1 | Lactiplantibacillus plantarum | France | Europe | Lactobacillaceae | Probiotic |
| GCF_001704335.1 | Lactiplantibacillus plantarum | Canada | North America | Lactobacillaceae | Probiotic |
| GCF_014324175.1 | Lactiplantibacillus plantarum | Italy | Europe | Lactobacillaceae | Probiotic |
| GCF_033792305.1 | Lactiplantibacillus plantarum | Madagascar | Africa | Lactobacillaceae | Probiotic |
| GCF_030549465.1 | Lactiplantibacillus plantarum | China | Asia | Lactobacillaceae | Probiotic |
| GCF_023195495.1 | Lactiplantibacillus plantarum | China | Asia | Lactobacillaceae | Probiotic |
| GCF_004102885.1 | Lactiplantibacillus plantarum | China | Asia | Lactobacillaceae | Probiotic |
| GCF_034321885.1 | Lactiplantibacillus plantarum | India | Asia | Lactobacillaceae | Probiotic |
| GCF_002532115.1 | Lactiplantibacillus plantarum | Croatia | Europe | Lactobacillaceae | Probiotic |
| GCF_035338015.1 | Lactiplantibacillus plantarum | Taiwan | Asia | Lactobacillaceae | Probiotic |
| GCF_035336205.1 | Lactiplantibacillus plantarum | Taiwan | Asia | Lactobacillaceae | Probiotic |
| GCF_016812075.1 | Lactiplantibacillus plantarum | China | Asia | Lactobacillaceae | Probiotic |
| GCF_003990985.1 | Lactiplantibacillus plantarum | India | Asia | Lactobacillaceae | Probiotic |
| GCF_036281295.1 | Lactiplantibacillus plantarum | Missing | Missing | Lactobacillaceae | Probiotic |
| GCF_036281355.1 | Lactiplantibacillus plantarum | Missing | Missing | Lactobacillaceae | Probiotic |
| GCF_037414365.1 | Lactiplantibacillus plantarum | South Korea | Asia | Lactobacillaceae | Probiotic |
| GCF_037414405.1 | Lactiplantibacillus plantarum | South Korea | Asia | Lactobacillaceae | Probiotic |
| GCF_003325775.1 | Lactiplantibacillus plantarum | Argentina | South America | Lactobacillaceae | Probiotic |
| GCF_001444495.1 | Lactiplantibacillus plantarum | Argentina | South America | Lactobacillaceae | Probiotic |
| GCF_034333585.1 | Lactiplantibacillus plantarum | China | Asia | Lactobacillaceae | Probiotic |
| GCF_018403705.1 | Lactiplantibacillus plantarum | Indonesia | Asia | Lactobacillaceae | Probiotic |
| GCF_001633285.1 | Lactiplantibacillus plantarum | USA | North America | Lactobacillaceae | Probiotic |
| GCF_013694305.1 | Lactiplantibacillus plantarum | Nigeria | Africa | Lactobacillaceae | Probiotic |
| GCF_013155145.1 | Lactiplantibacillus plantarum | Nigeria | Africa | Lactobacillaceae | Probiotic |
| GCF_000762955.1 | Lactiplantibacillus plantarum | Belgium | Europe | Lactobacillaceae | Probiotic |
| GCF_030480525.1 | Lactiplantibacillus plantarum | Mexico | North America | Lactobacillaceae | Probiotic |
| GCF_030480505.1 | Lactiplantibacillus plantarum | Mexico | North America | Lactobacillaceae | Probiotic |
| GCF_030480565.1 | Lactiplantibacillus plantarum | Mexico | North America | Lactobacillaceae | Probiotic |
| GCF_002024845.1 | Lactiplantibacillus plantarum | China | Asia | Lactobacillaceae | Probiotic |
| GCF_025190015.1 | Lactiplantibacillus plantarum | Spain | Europe | Lactobacillaceae | Probiotic |
| GCF_025212005.1 | Lactiplantibacillus plantarum | Egypt | Africa | Lactobacillaceae | Probiotic |
| GCF_025189965.1 | Lactiplantibacillus plantarum | France | Europe | Lactobacillaceae | Probiotic |
| GCF_025189985.1 | Lactiplantibacillus plantarum | Missing | Missing | Lactobacillaceae | Probiotic |
| GCF_025189945.1 | Lactiplantibacillus plantarum | United Kingdom | Europe | Lactobacillaceae | Probiotic |
| GCF_025212155.1 | Lactiplantibacillus plantarum | France | Europe | Lactobacillaceae | Probiotic |
| GCF_025212165.1 | Lactiplantibacillus plantarum | France | Europe | Lactobacillaceae | Probiotic |
| GCF_025190225.1 | Lactiplantibacillus plantarum | France | Europe | Lactobacillaceae | Probiotic |
| GCF_025190145.1 | Lactiplantibacillus plantarum | France | Europe | Lactobacillaceae | Probiotic |
| GCF_025212205.1 | Lactiplantibacillus plantarum | France | Europe | Lactobacillaceae | Probiotic |
| GCF_025190185.1 | Lactiplantibacillus plantarum | France | Europe | Lactobacillaceae | Probiotic |
| GCF_025190205.1 | Lactiplantibacillus plantarum | France | Europe | Lactobacillaceae | Probiotic |
| GCF_025190165.1 | Lactiplantibacillus plantarum | France | Europe | Lactobacillaceae | Probiotic |
| GCF_025189685.1 | Lactiplantibacillus plantarum | Missing | Missing | Lactobacillaceae | Probiotic |
| GCF_025189845.1 | Lactiplantibacillus plantarum | France | Europe | Lactobacillaceae | Probiotic |
| GCF_025189925.1 | Lactiplantibacillus plantarum | France | Europe | Lactobacillaceae | Probiotic |
| GCF_025189885.1 | Lactiplantibacillus plantarum | France | Europe | Lactobacillaceae | Probiotic |
| GCF_025190125.1 | Lactiplantibacillus plantarum | Egypt | Africa | Lactobacillaceae | Probiotic |
| GCF_025190105.1 | Lactiplantibacillus plantarum | Missing | Missing | Lactobacillaceae | Probiotic |
| GCF_025189815.1 | Lactiplantibacillus plantarum | France | Europe | Lactobacillaceae | Probiotic |
| GCF_025189865.1 | Lactiplantibacillus plantarum | France | Europe | Lactobacillaceae | Probiotic |
| GCF_025194145.1 | Lactiplantibacillus plantarum | France | Europe | Lactobacillaceae | Probiotic |
| GCF_025189905.1 | Lactiplantibacillus plantarum | France | Europe | Lactobacillaceae | Probiotic |
| GCF_025190005.1 | Lactiplantibacillus plantarum | France | Europe | Lactobacillaceae | Probiotic |
| GCF_025190035.1 | Lactiplantibacillus plantarum | France | Europe | Lactobacillaceae | Probiotic |
| GCF_025190025.1 | Lactiplantibacillus plantarum | Italy | Europe | Lactobacillaceae | Probiotic |
| GCF_000956195.1 | Lactiplantibacillus plantarum | France | Europe | Lactobacillaceae | Probiotic |
| GCF_003344845.1 | Lactiplantibacillus plantarum | China | Asia | Lactobacillaceae | Probiotic |
| GCF_001754005.1 | Lactiplantibacillus plantarum | China | Asia | Lactobacillaceae | Probiotic |
| GCF_036321605.1 | Lactiplantibacillus plantarum | China | Asia | Lactobacillaceae | Probiotic |
| GCF_036321505.1 | Lactiplantibacillus plantarum | China | Asia | Lactobacillaceae | Probiotic |
| GCF_001272315.2 | Lactiplantibacillus plantarum | China | Asia | Lactobacillaceae | Probiotic |
| GCF_900695365.1 | Lactiplantibacillus plantarum | Argentina | South America | Lactobacillaceae | Probiotic |
| GCF_900695295.1 | Lactiplantibacillus plantarum | Argentina | South America | Lactobacillaceae | Probiotic |
| GCF_902825385.1 | Lactiplantibacillus plantarum | Argentina | South America | Lactobacillaceae | Probiotic |
| GCF_900700275.1 | Lactiplantibacillus plantarum | Argentina | South America | Lactobacillaceae | Probiotic |
| GCF_900695425.1 | Lactiplantibacillus plantarum | Argentina | South America | Lactobacillaceae | Probiotic |
| GCF_900290085.1 | Lactiplantibacillus plantarum | Argentina | South America | Lactobacillaceae | Probiotic |
| GCF_900290125.1 | Lactiplantibacillus plantarum | Argentina | South America | Lactobacillaceae | Probiotic |
| GCF_900290105.1 | Lactiplantibacillus plantarum | Argentina | South America | Lactobacillaceae | Probiotic |
| GCF_900290135.1 | Lactiplantibacillus plantarum | Argentina | South America | Lactobacillaceae | Probiotic |
| GCF_900289155.1 | Lactiplantibacillus plantarum | Argentina | South America | Lactobacillaceae | Probiotic |
| GCF_029623555.1 | Lactiplantibacillus plantarum | South Korea | Asia | Lactobacillaceae | Probiotic |
| GCF_003344825.1 | Lactiplantibacillus plantarum | China | Asia | Lactobacillaceae | Probiotic |
| GCF_001617525.2 | Lactiplantibacillus plantarum | China | Asia | Lactobacillaceae | Probiotic |
| GCF_010092485.1 | Lactiplantibacillus plantarum | South Korea | Asia | Lactobacillaceae | Probiotic |
| GCF_020552525.1 | Lactiplantibacillus plantarum | China | Asia | Lactobacillaceae | Probiotic |
| GCF_020552635.1 | Lactiplantibacillus plantarum | China | Asia | Lactobacillaceae | Probiotic |
| GCF_020552625.1 | Lactiplantibacillus plantarum | China | Asia | Lactobacillaceae | Probiotic |
| GCF_020552595.1 | Lactiplantibacillus plantarum | China | Asia | Lactobacillaceae | Probiotic |
| GCF_020552585.1 | Lactiplantibacillus plantarum | China | Asia | Lactobacillaceae | Probiotic |
| GCF_001874125.1 | Lactiplantibacillus plantarum | China | Asia | Lactobacillaceae | Probiotic |
| GCF_002994725.1 | Lactiplantibacillus plantarum | Spain | Europe | Lactobacillaceae | Probiotic |
| GCF_030237845.1 | Lactiplantibacillus plantarum | China | Asia | Lactobacillaceae | Probiotic |
| GCF_030237865.1 | Lactiplantibacillus plantarum | China | Asia | Lactobacillaceae | Probiotic |
| GCF_003577505.1 | Lactiplantibacillus plantarum | South Korea | Asia | Lactobacillaceae | Probiotic |
| GCF_030549385.1 | Lactiplantibacillus plantarum | China | Asia | Lactobacillaceae | Probiotic |
| GCF_008016925.1 | Lactiplantibacillus plantarum | Spain | Europe | Lactobacillaceae | Probiotic |
| GCF_031032805.1 | Lactiplantibacillus plantarum | Philippines | Asia | Lactobacillaceae | Probiotic |
| GCF_031085385.1 | Lactiplantibacillus plantarum | India | Asia | Lactobacillaceae | Probiotic |
| GCF_026183415.1 | Lactiplantibacillus plantarum | South Korea | Asia | Lactobacillaceae | Probiotic |
| GCF_002914965.1 | Lactiplantibacillus plantarum | Italy | Europe | Lactobacillaceae | Probiotic |
| GCF_002116955.1 | Lactiplantibacillus plantarum | South Korea | Asia | Lactobacillaceae | Probiotic |
| GCF_009756965.1 | Lactiplantibacillus plantarum | South Korea | Asia | Lactobacillaceae | Probiotic |
| GCF_013487805.1 | Lactiplantibacillus plantarum | South Korea | Asia | Lactobacillaceae | Probiotic |
| GCF_008868495.1 | Lactiplantibacillus plantarum | Missing | Missing | Lactobacillaceae | Probiotic |
| GCF_025122345.1 | Lactiplantibacillus plantarum | France | Europe | Lactobacillaceae | Probiotic |
| GCF_030504815.1 | Lactiplantibacillus plantarum | Serbia | Europe | Lactobacillaceae | Probiotic |
| GCF_017580975.1 | Lactiplantibacillus plantarum | USA | North America | Lactobacillaceae | Probiotic |
| GCF_029269595.1 | Lactiplantibacillus plantarum | Serbia | Europe | Lactobacillaceae | Probiotic |
| GCF_900078525.1 | Lactiplantibacillus plantarum | Kenya | Africa | Lactobacillaceae | Probiotic |
| GCF_034555115.1 | Lactiplantibacillus plantarum | Spain | Europe | Lactobacillaceae | Probiotic |
| GCF_025723165.1 | Lactiplantibacillus plantarum | China | Asia | Lactobacillaceae | Probiotic |
| GCF_033546835.1 | Lactiplantibacillus plantarum | South Korea | Asia | Lactobacillaceae | Probiotic |
| GCF_002290185.1 | Lactiplantibacillus plantarum | USA | North America | Lactobacillaceae | Probiotic |
| GCF_014084065.1 | Lactiplantibacillus plantarum | Thailand | Asia | Lactobacillaceae | Probiotic |
| GCF_030503695.1 | Lactiplantibacillus plantarum | China | Asia | Lactobacillaceae | Probiotic |
| GCF_026689375.1 | Lactiplantibacillus plantarum | China | Asia | Lactobacillaceae | Probiotic |
| GCF_032248515.1 | Lactiplantibacillus plantarum | Taiwan | Asia | Lactobacillaceae | Probiotic |
| GCF_026622935.1 | Lactiplantibacillus plantarum | China | Asia | Lactobacillaceae | Probiotic |
| GCF_004301795.1 | Lactiplantibacillus plantarum | China | Asia | Lactobacillaceae | Probiotic |
| GCF_004301805.1 | Lactiplantibacillus plantarum | China | Asia | Lactobacillaceae | Probiotic |
| GCF_004301775.1 | Lactiplantibacillus plantarum | China | Asia | Lactobacillaceae | Probiotic |
| GCF_004301765.1 | Lactiplantibacillus plantarum | China | Asia | Lactobacillaceae | Probiotic |
| GCF_004301725.1 | Lactiplantibacillus plantarum | China | Asia | Lactobacillaceae | Probiotic |
| GCF_004301695.1 | Lactiplantibacillus plantarum | China | Asia | Lactobacillaceae | Probiotic |
| GCF_004301705.1 | Lactiplantibacillus plantarum | China | Asia | Lactobacillaceae | Probiotic |
| GCF_004301685.1 | Lactiplantibacillus plantarum | China | Asia | Lactobacillaceae | Probiotic |
| GCF_004301655.1 | Lactiplantibacillus plantarum | China | Asia | Lactobacillaceae | Probiotic |
| GCF_004301645.1 | Lactiplantibacillus plantarum | China | Asia | Lactobacillaceae | Probiotic |
| GCF_030061935.1 | Lactiplantibacillus plantarum | China | Asia | Lactobacillaceae | Probiotic |
| GCF_004301565.1 | Lactiplantibacillus plantarum | China | Asia | Lactobacillaceae | Probiotic |
| GCF_004301595.1 | Lactiplantibacillus plantarum | China | Asia | Lactobacillaceae | Probiotic |
| GCF_000931425.2 | Lactiplantibacillus plantarum | Viet Nam | Asia | Lactobacillaceae | Probiotic |
| GCF_003352125.1 | Lactiplantibacillus plantarum | China | Asia | Lactobacillaceae | Probiotic |
| GCF_004301865.1 | Lactiplantibacillus plantarum | China | Asia | Lactobacillaceae | Probiotic |
| GCF_004301815.1 | Lactiplantibacillus plantarum | China | Asia | Lactobacillaceae | Probiotic |
| GCF_004301545.1 | Lactiplantibacillus plantarum | China | Asia | Lactobacillaceae | Probiotic |
| GCF_017580905.1 | Lactiplantibacillus plantarum | Ethiopia | Africa | Lactobacillaceae | Probiotic |
| GCF_017580925.1 | Lactiplantibacillus plantarum | Ethiopia | Africa | Lactobacillaceae | Probiotic |
| GCF_004122965.1 | Lactiplantibacillus plantarum | China | Asia | Lactobacillaceae | Probiotic |
| GCF_030297735.1 | Lactiplantibacillus plantarum | Japan | Asia | Lactobacillaceae | Probiotic |
| GCF_030297715.1 | Lactiplantibacillus plantarum | Japan | Asia | Lactobacillaceae | Probiotic |
| GCF_003597615.1 | Lactiplantibacillus plantarum | South Korea | Asia | Lactobacillaceae | Probiotic |
| GCF_003597595.1 | Lactiplantibacillus plantarum | South Korea | Asia | Lactobacillaceae | Probiotic |
| GCF_003597635.1 | Lactiplantibacillus plantarum | South Korea | Asia | Lactobacillaceae | Probiotic |
| GCF_002631775.1 | Lactiplantibacillus plantarum | New Zealand | Oceania | Lactobacillaceae | Probiotic |
| GCF_002370965.1 | Lactiplantibacillus plantarum | Missing | Missing | Lactobacillaceae | Probiotic |
| GCF_002749655.1 | Lactiplantibacillus plantarum | Missing | Missing | Lactobacillaceae | Probiotic |
| GCF_013753885.1 | Lactiplantibacillus plantarum | Sweden | Europe | Lactobacillaceae | Probiotic |
| GCF_004354995.1 | Lactiplantibacillus plantarum | USA | North America | Lactobacillaceae | Probiotic |
| GCF_018351295.1 | Lactiplantibacillus plantarum | USA | North America | Lactobacillaceae | Probiotic |
| GCF_003045705.1 | Lactiplantibacillus plantarum | Pakistan | Asia | Lactobacillaceae | Probiotic |
| GCF_003045665.1 | Lactiplantibacillus plantarum | Pakistan | Asia | Lactobacillaceae | Probiotic |
| GCF_003045725.1 | Lactiplantibacillus plantarum | Pakistan | Asia | Lactobacillaceae | Probiotic |
| GCF_003045645.1 | Lactiplantibacillus plantarum | Pakistan | Asia | Lactobacillaceae | Probiotic |
| GCF_017351995.1 | Lactiplantibacillus plantarum | China | Asia | Lactobacillaceae | Probiotic |
| GCF_026156615.1 | Lactiplantibacillus plantarum | China | Asia | Lactobacillaceae | Probiotic |
| GCF_012974545.1 | Lactiplantibacillus plantarum | South Korea | Asia | Lactobacillaceae | Probiotic |
| GCF_027698045.1 | Lactiplantibacillus plantarum | China | Asia | Lactobacillaceae | Probiotic |
| GCF_027698165.1 | Lactiplantibacillus plantarum | China | Asia | Lactobacillaceae | Probiotic |
| GCF_027698905.1 | Lactiplantibacillus plantarum | China | Asia | Lactobacillaceae | Probiotic |
| GCF_027671205.1 | Lactiplantibacillus plantarum | China | Asia | Lactobacillaceae | Probiotic |
| GCF_003469805.1 | Lactiplantibacillus plantarum | China | Asia | Lactobacillaceae | Probiotic |
| GCF_003470765.1 | Lactiplantibacillus plantarum | China | Asia | Lactobacillaceae | Probiotic |
| GCF_027661015.1 | Lactiplantibacillus plantarum | China | Asia | Lactobacillaceae | Probiotic |
| GCF_027661665.1 | Lactiplantibacillus plantarum | China | Asia | Lactobacillaceae | Probiotic |
| GCF_033055415.1 | Lactiplantibacillus plantarum | China | Asia | Lactobacillaceae | Probiotic |
| GCF_017581005.1 | Lactiplantibacillus plantarum | USA | North America | Lactobacillaceae | Probiotic |
| GCF_000687495.1 | Lactiplantibacillus plantarum | China | Asia | Lactobacillaceae | Probiotic |
| GCF_029767725.1 | Lactiplantibacillus plantarum | Russia | Europe | Lactobacillaceae | Probiotic |
| GCF_027664985.1 | Lactiplantibacillus plantarum | China | Asia | Lactobacillaceae | Probiotic |
| GCF_021559915.1 | Lactiplantibacillus plantarum | China | Asia | Lactobacillaceae | Probiotic |
| GCF_004301455.1 | Lactiplantibacillus plantarum | China | Asia | Lactobacillaceae | Probiotic |
| GCF_004301435.1 | Lactiplantibacillus plantarum | China | Asia | Lactobacillaceae | Probiotic |
| GCF_004301445.1 | Lactiplantibacillus plantarum | China | Asia | Lactobacillaceae | Probiotic |
| GCF_004301425.1 | Lactiplantibacillus plantarum | China | Asia | Lactobacillaceae | Probiotic |
| GCF_002249825.1 | Lactiplantibacillus plantarum | Viet Nam | Asia | Lactobacillaceae | Probiotic |
| GCF_004301395.1 | Lactiplantibacillus plantarum | China | Asia | Lactobacillaceae | Probiotic |
| GCF_004301355.1 | Lactiplantibacillus plantarum | China | Asia | Lactobacillaceae | Probiotic |
| GCF_004301345.1 | Lactiplantibacillus plantarum | China | Asia | Lactobacillaceae | Probiotic |
| GCF_004301335.1 | Lactiplantibacillus plantarum | China | Asia | Lactobacillaceae | Probiotic |
| GCF_004301325.1 | Lactiplantibacillus plantarum | China | Asia | Lactobacillaceae | Probiotic |
| GCF_004301305.1 | Lactiplantibacillus plantarum | China | Asia | Lactobacillaceae | Probiotic |
| GCF_001888525.1 | Lactiplantibacillus plantarum | Thailand | Asia | Lactobacillaceae | Probiotic |
| GCF_004301255.1 | Lactiplantibacillus plantarum | China | Asia | Lactobacillaceae | Probiotic |
| GCF_004301235.1 | Lactiplantibacillus plantarum | China | Asia | Lactobacillaceae | Probiotic |
| GCF_004301555.1 | Lactiplantibacillus plantarum | China | Asia | Lactobacillaceae | Probiotic |
| GCF_004301485.1 | Lactiplantibacillus plantarum | China | Asia | Lactobacillaceae | Probiotic |
| GCF_004301245.1 | Lactiplantibacillus plantarum | China | Asia | Lactobacillaceae | Probiotic |
| GCF_004301225.1 | Lactiplantibacillus plantarum | China | Asia | Lactobacillaceae | Probiotic |
| GCF_028462625.1 | Lactiplantibacillus plantarum | Germany | Europe | Lactobacillaceae | Probiotic |
| GCF_028462565.1 | Lactiplantibacillus plantarum | Germany | Europe | Lactobacillaceae | Probiotic |
| GCF_028462545.1 | Lactiplantibacillus plantarum | Germany | Europe | Lactobacillaceae | Probiotic |
| GCF_000830535.1 | Lactiplantibacillus plantarum | Russia | Europe | Lactobacillaceae | Probiotic |
| GCF_019311725.2 | Lactiplantibacillus plantarum | Russia | Europe | Lactobacillaceae | Probiotic |
| GCF_004404125.1 | Lactiplantibacillus plantarum | Russia | Europe | Lactobacillaceae | Probiotic |
| GCF_004403045.2 | Lactiplantibacillus plantarum | Russia | Europe | Lactobacillaceae | Probiotic |
| GCF_009762745.1 | Lactiplantibacillus plantarum | Russia | Europe | Lactobacillaceae | Probiotic |
| GCF_009759825.1 | Lactiplantibacillus plantarum | Russia | Europe | Lactobacillaceae | Probiotic |
| GCF_001368775.1 | Lactiplantibacillus plantarum | Ghana | Africa | Lactobacillaceae | Probiotic |
| GCF_017581045.1 | Lactiplantibacillus plantarum | Bulgaria | Europe | Lactobacillaceae | Probiotic |
| GCF_001010175.1 | Lactiplantibacillus plantarum | Russia | Europe | Lactobacillaceae | Probiotic |
| GCF_002234395.1 | Lactiplantibacillus plantarum | Russia | Europe | Lactobacillaceae | Probiotic |
| GCF_018993285.1 | Lactiplantibacillus plantarum | USA | North America | Lactobacillaceae | Probiotic |
| GCF_032190715.1 | Lactiplantibacillus plantarum | USA | North America | Lactobacillaceae | Probiotic |
| GCF_001278015.1 | Lactiplantibacillus plantarum | China | Asia | Lactobacillaceae | Probiotic |
| GCF_003347445.1 | Lactiplantibacillus plantarum | Brazil | South America | Lactobacillaceae | Probiotic |
| GCF_024999925.1 | Lactiplantibacillus plantarum | Missing | Missing | Lactobacillaceae | Probiotic |
| GCF_001595615.1 | Lactiplantibacillus plantarum | Kazakhstan | Asia | Lactobacillaceae | Probiotic |
| GCF_019399915.1 | Lactiplantibacillus plantarum | Ireland | Europe | Lactobacillaceae | Probiotic |
| GCF_030504705.1 | Lactiplantibacillus plantarum | Lithuania | Europe | Lactobacillaceae | Probiotic |
| GCF_000507045.1 | Lactiplantibacillus plantarum | China | Asia | Lactobacillaceae | Probiotic |
| GCF_019390145.1 | Lactiplantibacillus plantarum | Ireland | Europe | Lactobacillaceae | Probiotic |
| GCF_029849115.1 | Lactiplantibacillus plantarum | Lithuania | Europe | Lactobacillaceae | Probiotic |
| GCF_024137985.1 | Lactiplantibacillus plantarum | China | Asia | Lactobacillaceae | Probiotic |
| GCF_018991195.1 | Lactiplantibacillus plantarum | USA | North America | Lactobacillaceae | Probiotic |
| GCF_001888735.1 | Lactiplantibacillus plantarum | Sweden | Europe | Lactobacillaceae | Probiotic |
| GCF_028656165.1 | Lactiplantibacillus plantarum | China | Asia | Lactobacillaceae | Probiotic |
| GCF_036898925.1 | Lactiplantibacillus plantarum | China | Asia | Lactobacillaceae | Probiotic |
| GCF_000466905.3 | Lactiplantibacillus plantarum | Russia | Europe | Lactobacillaceae | Probiotic |
| GCF_010586945.1 | Lactiplantibacillus plantarum | Denmark | Europe | Lactobacillaceae | Probiotic |
| GCF_003347455.1 | Lactiplantibacillus plantarum | Brazil | South America | Lactobacillaceae | Probiotic |
| GCF_017581065.1 | Lactiplantibacillus plantarum | USA | North America | Lactobacillaceae | Probiotic |
| GCF_000604365.1 | Lactiplantibacillus plantarum | Slovakia | Europe | Lactobacillaceae | Probiotic |
| GCF_025129295.1 | Lactiplantibacillus plantarum | Missing | Missing | Lactobacillaceae | Probiotic |
| GCF_001633265.1 | Lactiplantibacillus plantarum | Israel | Asia | Lactobacillaceae | Probiotic |
| GCF_000412205.1 | Lactiplantibacillus plantarum | Ireland | Europe | Lactobacillaceae | Probiotic |
| GCF_036762805.1 | Lactiplantibacillus plantarum | Missing | Missing | Lactobacillaceae | Probiotic |
| GCF_025133105.1 | Lactiplantibacillus plantarum | Mali | Africa | Lactobacillaceae | Probiotic |
| GCF_025133155.1 | Lactiplantibacillus plantarum | Switzerland | Europe | Lactobacillaceae | Probiotic |
| GCF_025131215.1 | Lactiplantibacillus plantarum | Switzerland | Europe | Lactobacillaceae | Probiotic |
| GCF_019390095.1 | Lactiplantibacillus plantarum | Ireland | Europe | Lactobacillaceae | Probiotic |
| GCF_004028315.1 | Lactiplantibacillus plantarum | China | Asia | Lactobacillaceae | Probiotic |
| GCF_004028335.1 | Lactiplantibacillus plantarum | China | Asia | Lactobacillaceae | Probiotic |
| GCF_017301935.1 | Lactiplantibacillus plantarum | China | Asia | Lactobacillaceae | Probiotic |
| GCF_019890755.1 | Lactiplantibacillus plantarum | Brazil | South America | Lactobacillaceae | Probiotic |
| GCF_002005385.2 | Lactiplantibacillus plantarum | United Kingdom | Europe | Lactobacillaceae | Probiotic |
| GCF_025129255.1 | Lactiplantibacillus plantarum | Ethiopia | Africa | Lactobacillaceae | Probiotic |
| GCF_028322885.1 | Lactiplantibacillus plantarum | USA | North America | Lactobacillaceae | Probiotic |
| GCF_015558185.1 | Lactiplantibacillus plantarum | USA | North America | Lactobacillaceae | Probiotic |
| GCF_028321915.1 | Lactiplantibacillus plantarum | USA | North America | Lactobacillaceae | Probiotic |
| GCF_015549145.1 | Lactiplantibacillus plantarum | USA | North America | Lactobacillaceae | Probiotic |
| GCF_015561225.1 | Lactiplantibacillus plantarum | USA | North America | Lactobacillaceae | Probiotic |
| GCF_015556355.1 | Lactiplantibacillus plantarum | USA | North America | Lactobacillaceae | Probiotic |
| GCF_028321905.1 | Lactiplantibacillus plantarum | USA | North America | Lactobacillaceae | Probiotic |
| GCF_028323095.1 | Lactiplantibacillus plantarum | USA | North America | Lactobacillaceae | Probiotic |
| GCF_028321145.1 | Lactiplantibacillus plantarum | USA | North America | Lactobacillaceae | Probiotic |
| GCF_015559875.1 | Lactiplantibacillus plantarum | USA | North America | Lactobacillaceae | Probiotic |
| GCF_015547405.1 | Lactiplantibacillus plantarum | USA | North America | Lactobacillaceae | Probiotic |
| GCF_021279005.2 | Lactiplantibacillus plantarum | India | Asia | Lactobacillaceae | Probiotic |
| GCF_019655875.1 | Lactiplantibacillus plantarum | Thailand | Asia | Lactobacillaceae | Probiotic |
| GCF_900176235.1 | Lactiplantibacillus plantarum | Missing | Africa | Lactobacillaceae | Probiotic |
| GCF_900200135.1 | Lactiplantibacillus plantarum | Missing | Missing | Lactobacillaceae | Probiotic |
| GCF_925286555.1 | Lactiplantibacillus plantarum | Slovenia | Europe | Lactobacillaceae | Probiotic |
| GCF_925301115.1 | Lactiplantibacillus plantarum | Italy | Europe | Lactobacillaceae | Probiotic |
| GCF_925320525.1 | Lactiplantibacillus plantarum | Italy | Europe | Lactobacillaceae | Probiotic |
| GCF_925291875.1 | Lactiplantibacillus plantarum | Slovenia | Europe | Lactobacillaceae | Probiotic |
| GCF_925301295.1 | Lactiplantibacillus plantarum | Missing | Missing | Lactobacillaceae | Probiotic |
| GCF_925281465.1 | Lactiplantibacillus plantarum | Missing | Missing | Lactobacillaceae | Probiotic |
| GCF_900080205.1 | Lactiplantibacillus plantarum | Kenya | Africa | Lactobacillaceae | Probiotic |
| GCF_900095045.1 | Lactiplantibacillus plantarum | Italy | Europe | Lactobacillaceae | Probiotic |
| GCF_900095065.1 | Lactiplantibacillus plantarum | Italy | Europe | Lactobacillaceae | Probiotic |
| GCF_900095055.1 | Lactiplantibacillus plantarum | Italy | Europe | Lactobacillaceae | Probiotic |
| GCF_901830435.1 | Lactiplantibacillus plantarum | Belgium | Europe | Lactobacillaceae | Probiotic |
| GCF_902386645.1 | Lactiplantibacillus plantarum | China | Asia | Lactobacillaceae | Probiotic |
| GCF_002174195.1 | Lactiplantibacillus plantarum | South Korea | Asia | Lactobacillaceae | Probiotic |
| GCF_001436855.1 | Lactiplantibacillus plantarum | USA | North America | Lactobacillaceae | Probiotic |
| GCF_000143745.1 | Lactiplantibacillus plantarum | Missing | Missing | Lactobacillaceae | Probiotic |
| GCF_003952845.1 | Lactobacillus acidophilus | South Korea | Asia | Lactobacillaceae | Probiotic |
| GCF_001639165.1 | Lactobacillus acidophilus | Missing | Missing | Lactobacillaceae | Probiotic |
| GCF_030369715.1 | Lactobacillus acidophilus | China | Asia | Lactobacillaceae | Probiotic |
| GCF_003641085.1 | Lactobacillus acidophilus | India | Asia | Lactobacillaceae | Probiotic |
| GCF_027690645.1 | Lactobacillus acidophilus | China | Asia | Lactobacillaceae | Probiotic |
| GCF_027690935.1 | Lactobacillus acidophilus | China | Asia | Lactobacillaceae | Probiotic |
| GCF_013867555.1 | Lactobacillus acidophilus | China | Asia | Lactobacillaceae | Probiotic |
| GCF_013867605.1 | Lactobacillus acidophilus | China | Asia | Lactobacillaceae | Probiotic |
| GCF_022509485.1 | Lactobacillus acidophilus | Pakistan | Asia | Lactobacillaceae | Probiotic |
| GCF_023093425.1 | Lactobacillus acidophilus | Canada | North America | Lactobacillaceae | Probiotic |
| GCF_033569435.1 | Lactobacillus acidophilus | USA | North America | Lactobacillaceae | Probiotic |
| GCF_002406675.1 | Lactobacillus acidophilus | Canada | North America | Lactobacillaceae | Probiotic |
| GCF_027692675.1 | Lactobacillus acidophilus | China | Asia | Lactobacillaceae | Probiotic |
| GCF_033598375.1 | Lactobacillus acidophilus | South Africa | Africa | Lactobacillaceae | Probiotic |
| GCF_000011985.1 | Lactobacillus acidophilus | USA | North America | Lactobacillaceae | Probiotic |
| GCF_025665255.1 | Lactobacillus acidophilus | China | Asia | Lactobacillaceae | Probiotic |
| GCF_020883435.1 | Lactobacillus acidophilus | China | Asia | Lactobacillaceae | Probiotic |
| GCF_021229035.1 | Lactobacillus acidophilus | Bulgaria | Europe | Lactobacillaceae | Probiotic |
| GCF_029334915.1 | Lactobacillus acidophilus | Bangladesh | Asia | Lactobacillaceae | Probiotic |
| GCF_036350535.1 | Lactobacillus acidophilus | India | Asia | Lactobacillaceae | Probiotic |
| GCF_036347815.1 | Lactobacillus acidophilus | India | Asia | Lactobacillaceae | Probiotic |
| GCF_013342945.1 | Lactobacillus acidophilus | Missing | Missing | Lactobacillaceae | Probiotic |
| GCF_018252545.1 | Lactobacillus acidophilus | China | Asia | Lactobacillaceae | Probiotic |
| GCF_017009715.1 | Lactobacillus acidophilus | Missing | Missing | Lactobacillaceae | Probiotic |
| GCF_024665555.1 | Lactobacillus acidophilus | Missing | Missing | Lactobacillaceae | Probiotic |
| GCF_024665075.1 | Lactobacillus acidophilus | Missing | Missing | Lactobacillaceae | Probiotic |
| GCF_000389675.2 | Lactobacillus acidophilus | Missing | Missing | Lactobacillaceae | Probiotic |
| GCF_017009725.1 | Lactobacillus acidophilus | Taiwan | Asia | Lactobacillaceae | Probiotic |
| GCF_002286215.1 | Lactobacillus acidophilus | South Korea | Asia | Lactobacillaceae | Probiotic |
| GCF_032917925.1 | Lactobacillus acidophilus | China | Asia | Lactobacillaceae | Probiotic |
| GCF_009741835.1 | Lactobacillus acidophilus | Missing | Missing | Lactobacillaceae | Probiotic |
| GCF_009742735.1 | Lactobacillus acidophilus | Missing | Missing | Lactobacillaceae | Probiotic |
| GCF_001950045.1 | Lactobacillus acidophilus | Japan | Asia | Lactobacillaceae | Probiotic |
| GCF_001868765.1 | Lactobacillus acidophilus | China | Asia | Lactobacillaceae | Probiotic |
| GCF_024397395.1 | Lactobacillus acidophilus | New Zealand | Oceania | Lactobacillaceae | Probiotic |
| GCF_000934625.1 | Lactobacillus acidophilus | USA | North America | Lactobacillaceae | Probiotic |
| GCF_000469745.1 | Lactobacillus acidophilus | Missing | Missing | Lactobacillaceae | Probiotic |
| GCF_000442825.1 | Lactobacillus acidophilus | Missing | Missing | Lactobacillaceae | Probiotic |
| GCF_003061925.1 | Lactobacillus acidophilus | USA | North America | Lactobacillaceae | Probiotic |
| GCF_003061945.1 | Lactobacillus acidophilus | USA | North America | Lactobacillaceae | Probiotic |
| GCF_003061985.1 | Lactobacillus acidophilus | USA | North America | Lactobacillaceae | Probiotic |
| GCF_003053135.1 | Lactobacillus acidophilus | USA | North America | Lactobacillaceae | Probiotic |
| GCF_003061885.1 | Lactobacillus acidophilus | USA | North America | Lactobacillaceae | Probiotic |
| GCF_003062005.1 | Lactobacillus acidophilus | USA | North America | Lactobacillaceae | Probiotic |
| GCF_003061905.1 | Lactobacillus acidophilus | USA | North America | Lactobacillaceae | Probiotic |
| GCF_003061965.1 | Lactobacillus acidophilus | USA | North America | Lactobacillaceae | Probiotic |
| GCF_003062025.1 | Lactobacillus acidophilus | USA | North America | Lactobacillaceae | Probiotic |
| GCF_003053245.1 | Lactobacillus acidophilus | USA | North America | Lactobacillaceae | Probiotic |
| GCF_003062045.1 | Lactobacillus acidophilus | USA | North America | Lactobacillaceae | Probiotic |
| GCF_025495965.1 | Lactobacillus acidophilus | United Kingdom | Europe | Lactobacillaceae | Probiotic |
| GCF_025194725.1 | Lactobacillus acidophilus | Mongolia | Asia | Lactobacillaceae | Probiotic |
| GCF_025194705.1 | Lactobacillus acidophilus | France | Europe | Lactobacillaceae | Probiotic |
| GCF_025194745.1 | Lactobacillus acidophilus | France | Europe | Lactobacillaceae | Probiotic |
| GCF_025194905.1 | Lactobacillus acidophilus | Taiwan | Asia | Lactobacillaceae | Probiotic |
| GCF_025194845.1 | Lactobacillus acidophilus | Taiwan | Asia | Lactobacillaceae | Probiotic |
| GCF_025194875.1 | Lactobacillus acidophilus | Taiwan | Asia | Lactobacillaceae | Probiotic |
| GCF_025194925.1 | Lactobacillus acidophilus | Taiwan | Asia | Lactobacillaceae | Probiotic |
| GCF_025194805.1 | Lactobacillus acidophilus | United Kingdom | Europe | Lactobacillaceae | Probiotic |
| GCF_000442865.1 | Lactobacillus acidophilus | USA | North America | Lactobacillaceae | Probiotic |
| GCF_025194825.1 | Lactobacillus acidophilus | USA | North America | Lactobacillaceae | Probiotic |
| GCF_025194865.1 | Lactobacillus acidophilus | USA | North America | Lactobacillaceae | Probiotic |
| GCF_025194665.1 | Lactobacillus acidophilus | Missing | Missing | Lactobacillaceae | Probiotic |
| GCF_025194775.1 | Lactobacillus acidophilus | USA | North America | Lactobacillaceae | Probiotic |
| GCF_025194685.1 | Lactobacillus acidophilus | USA | North America | Lactobacillaceae | Probiotic |
| GCF_032463485.1 | Lactobacillus acidophilus | China | Asia | Lactobacillaceae | Probiotic |
| GCF_000497795.1 | Lactobacillus acidophilus | USA | North America | Lactobacillaceae | Probiotic |
| GCF_008868625.1 | Lactobacillus acidophilus | Missing | Missing | Lactobacillaceae | Probiotic |
| GCF_017009695.1 | Lactobacillus acidophilus | Taiwan | Asia | Lactobacillaceae | Probiotic |
| GCF_017009585.1 | Lactobacillus acidophilus | Missing | Missing | Lactobacillaceae | Probiotic |
| GCF_017009655.1 | Lactobacillus acidophilus | Missing | Missing | Lactobacillaceae | Probiotic |
| GCF_017009595.1 | Lactobacillus acidophilus | USA | North America | Lactobacillaceae | Probiotic |
| GCF_017009605.1 | Lactobacillus acidophilus | USA | North America | Lactobacillaceae | Probiotic |
| GCF_017009575.1 | Lactobacillus acidophilus | Missing | Europe | Lactobacillaceae | Probiotic |
| GCF_017009475.1 | Lactobacillus acidophilus | Missing | Missing | Lactobacillaceae | Probiotic |
| GCF_017009515.1 | Lactobacillus acidophilus | Missing | Missing | Lactobacillaceae | Probiotic |
| GCF_017009485.1 | Lactobacillus acidophilus | Missing | Missing | Lactobacillaceae | Probiotic |
| GCF_002914945.1 | Lactobacillus acidophilus | Italy | Europe | Lactobacillaceae | Probiotic |
| GCF_030520065.1 | Lactobacillus acidophilus | Missing | Missing | Lactobacillaceae | Probiotic |
| GCF_002224305.1 | Lactobacillus acidophilus | USA | North America | Lactobacillaceae | Probiotic |
| GCF_000159715.1 | Lactobacillus acidophilus | Missing | Missing | Lactobacillaceae | Probiotic |
| GCF_030520045.1 | Lactobacillus acidophilus | Missing | Missing | Lactobacillaceae | Probiotic |
| GCF_017695935.1 | Lactobacillus acidophilus | Ireland | Europe | Lactobacillaceae | Probiotic |
| GCF_027672145.1 | Lactobacillus acidophilus | China | Asia | Lactobacillaceae | Probiotic |
| GCF_027672405.1 | Lactobacillus acidophilus | China | Asia | Lactobacillaceae | Probiotic |
| GCF_027659195.1 | Lactobacillus acidophilus | China | Asia | Lactobacillaceae | Probiotic |
| GCF_027659185.1 | Lactobacillus acidophilus | China | Asia | Lactobacillaceae | Probiotic |
| GCF_027659735.1 | Lactobacillus acidophilus | China | Asia | Lactobacillaceae | Probiotic |
| GCF_027660295.1 | Lactobacillus acidophilus | China | Asia | Lactobacillaceae | Probiotic |
| GCF_027660125.1 | Lactobacillus acidophilus | China | Asia | Lactobacillaceae | Probiotic |
| GCF_027660145.1 | Lactobacillus acidophilus | China | Asia | Lactobacillaceae | Probiotic |
| GCF_027660185.1 | Lactobacillus acidophilus | China | Asia | Lactobacillaceae | Probiotic |
| GCF_027662945.1 | Lactobacillus acidophilus | China | Asia | Lactobacillaceae | Probiotic |
| GCF_027680805.1 | Lactobacillus acidophilus | China | Asia | Lactobacillaceae | Probiotic |
| GCF_027684755.1 | Lactobacillus acidophilus | China | Asia | Lactobacillaceae | Probiotic |
| GCF_027687975.1 | Lactobacillus acidophilus | China | Asia | Lactobacillaceae | Probiotic |
| GCF_021432145.1 | Lactobacillus acidophilus | Russia | Europe | Lactobacillaceae | Probiotic |
| GCF_902386525.1 | Lactobacillus acidophilus | Missing | Missing | Lactobacillaceae | Probiotic |
| GCF_003047065.1 | Lactobacillus acidophilus | France | Europe | Lactobacillaceae | Probiotic |
| GCF_012843585.1 | Lactobacillus crispatus | Germany | Europe | Lactobacillaceae | Probiotic |
| GCF_029011475.1 | Lactobacillus crispatus | USA | North America | Lactobacillaceae | Probiotic |
| GCF_029011495.1 | Lactobacillus crispatus | USA | North America | Lactobacillaceae | Probiotic |
| GCF_029011595.1 | Lactobacillus crispatus | USA | North America | Lactobacillaceae | Probiotic |
| GCF_029011765.1 | Lactobacillus crispatus | USA | North America | Lactobacillaceae | Probiotic |
| GCF_029011155.1 | Lactobacillus crispatus | USA | North America | Lactobacillaceae | Probiotic |
| GCF_001541585.1 | Lactobacillus crispatus | USA | North America | Lactobacillaceae | Probiotic |
| GCF_001541535.1 | Lactobacillus crispatus | USA | North America | Lactobacillaceae | Probiotic |
| GCF_001541505.1 | Lactobacillus crispatus | USA | North America | Lactobacillaceae | Probiotic |
| GCF_001541515.1 | Lactobacillus crispatus | USA | North America | Lactobacillaceae | Probiotic |
| GCF_001541405.1 | Lactobacillus crispatus | USA | North America | Lactobacillaceae | Probiotic |
| GCF_001541385.1 | Lactobacillus crispatus | USA | North America | Lactobacillaceae | Probiotic |
| GCF_001546025.1 | Lactobacillus crispatus | USA | North America | Lactobacillaceae | Probiotic |
| GCF_001546015.1 | Lactobacillus crispatus | USA | North America | Lactobacillaceae | Probiotic |
| GCF_011029265.1 | Lactobacillus crispatus | Thailand | Asia | Lactobacillaceae | Probiotic |
| GCF_004681235.1 | Lactobacillus crispatus | France | Europe | Lactobacillaceae | Probiotic |
| GCF_002218735.1 | Lactobacillus crispatus | USA | North America | Lactobacillaceae | Probiotic |
| GCF_002218775.1 | Lactobacillus crispatus | USA | North America | Lactobacillaceae | Probiotic |
| GCF_002218695.1 | Lactobacillus crispatus | USA | North America | Lactobacillaceae | Probiotic |
| GCF_002218765.1 | Lactobacillus crispatus | USA | North America | Lactobacillaceae | Probiotic |
| GCF_002218655.1 | Lactobacillus crispatus | USA | North America | Lactobacillaceae | Probiotic |
| GCF_002218645.1 | Lactobacillus crispatus | USA | North America | Lactobacillaceae | Probiotic |
| GCF_002219055.1 | Lactobacillus crispatus | USA | North America | Lactobacillaceae | Probiotic |
| GCF_002219015.1 | Lactobacillus crispatus | USA | North America | Lactobacillaceae | Probiotic |
| GCF_002219085.1 | Lactobacillus crispatus | USA | North America | Lactobacillaceae | Probiotic |
| GCF_002219045.1 | Lactobacillus crispatus | USA | North America | Lactobacillaceae | Probiotic |
| GCF_002219005.1 | Lactobacillus crispatus | USA | North America | Lactobacillaceae | Probiotic |
| GCF_002218975.1 | Lactobacillus crispatus | USA | North America | Lactobacillaceae | Probiotic |
| GCF_002218565.1 | Lactobacillus crispatus | USA | North America | Lactobacillaceae | Probiotic |
| GCF_002218945.1 | Lactobacillus crispatus | USA | North America | Lactobacillaceae | Probiotic |
| GCF_002218925.1 | Lactobacillus crispatus | USA | North America | Lactobacillaceae | Probiotic |
| GCF_002218895.1 | Lactobacillus crispatus | USA | North America | Lactobacillaceae | Probiotic |
| GCF_002218885.1 | Lactobacillus crispatus | USA | North America | Lactobacillaceae | Probiotic |
| GCF_002218845.1 | Lactobacillus crispatus | USA | North America | Lactobacillaceae | Probiotic |
| GCF_002218855.1 | Lactobacillus crispatus | USA | North America | Lactobacillaceae | Probiotic |
| GCF_002218815.1 | Lactobacillus crispatus | USA | North America | Lactobacillaceae | Probiotic |
| GCF_002218805.1 | Lactobacillus crispatus | USA | North America | Lactobacillaceae | Probiotic |
| GCF_002218615.1 | Lactobacillus crispatus | USA | North America | Lactobacillaceae | Probiotic |
| GCF_030212455.1 | Lactobacillus crispatus | USA | North America | Lactobacillaceae | Probiotic |
| GCF_030212645.1 | Lactobacillus crispatus | USA | North America | Lactobacillaceae | Probiotic |
| GCF_030213555.1 | Lactobacillus crispatus | USA | North America | Lactobacillaceae | Probiotic |
| GCF_030230695.1 | Lactobacillus crispatus | USA | North America | Lactobacillaceae | Probiotic |
| GCF_030214445.1 | Lactobacillus crispatus | USA | North America | Lactobacillaceae | Probiotic |
| GCF_030219065.1 | Lactobacillus crispatus | USA | North America | Lactobacillaceae | Probiotic |
| GCF_030219025.1 | Lactobacillus crispatus | USA | North America | Lactobacillaceae | Probiotic |
| GCF_030226265.1 | Lactobacillus crispatus | USA | North America | Lactobacillaceae | Probiotic |
| GCF_030216545.1 | Lactobacillus crispatus | USA | North America | Lactobacillaceae | Probiotic |
| GCF_032376885.1 | Lactobacillus crispatus | USA | North America | Lactobacillaceae | Probiotic |
| GCF_033803945.1 | Lactobacillus crispatus | USA | North America | Lactobacillaceae | Probiotic |
| GCF_033803985.1 | Lactobacillus crispatus | USA | North America | Lactobacillaceae | Probiotic |
| GCF_033803925.1 | Lactobacillus crispatus | USA | North America | Lactobacillaceae | Probiotic |
| GCF_032376945.1 | Lactobacillus crispatus | USA | North America | Lactobacillaceae | Probiotic |
| GCF_033803625.1 | Lactobacillus crispatus | USA | North America | Lactobacillaceae | Probiotic |
| GCF_033803605.1 | Lactobacillus crispatus | USA | North America | Lactobacillaceae | Probiotic |
| GCF_032376685.1 | Lactobacillus crispatus | USA | North America | Lactobacillaceae | Probiotic |
| GCF_033803885.1 | Lactobacillus crispatus | USA | North America | Lactobacillaceae | Probiotic |
| GCF_033804265.1 | Lactobacillus crispatus | USA | North America | Lactobacillaceae | Probiotic |
| GCF_033804285.1 | Lactobacillus crispatus | USA | North America | Lactobacillaceae | Probiotic |
| GCF_033804305.1 | Lactobacillus crispatus | USA | North America | Lactobacillaceae | Probiotic |
| GCF_033803845.1 | Lactobacillus crispatus | USA | North America | Lactobacillaceae | Probiotic |
| GCF_033803565.1 | Lactobacillus crispatus | USA | North America | Lactobacillaceae | Probiotic |
| GCF_002863505.1 | Lactobacillus crispatus | USA | North America | Lactobacillaceae | Probiotic |
| GCF_012030075.1 | Lactobacillus crispatus | USA | North America | Lactobacillaceae | Probiotic |
| GCF_030230975.1 | Lactobacillus crispatus | USA | North America | Lactobacillaceae | Probiotic |
| GCF_030213125.1 | Lactobacillus crispatus | USA | North America | Lactobacillaceae | Probiotic |
| GCF_030230815.1 | Lactobacillus crispatus | USA | North America | Lactobacillaceae | Probiotic |
| GCF_030225085.1 | Lactobacillus crispatus | USA | North America | Lactobacillaceae | Probiotic |
| GCF_002861805.1 | Lactobacillus crispatus | USA | North America | Lactobacillaceae | Probiotic |
| GCF_002861765.1 | Lactobacillus crispatus | USA | North America | Lactobacillaceae | Probiotic |
| GCF_002861815.1 | Lactobacillus crispatus | USA | North America | Lactobacillaceae | Probiotic |
| GCF_030218605.1 | Lactobacillus crispatus | USA | North America | Lactobacillaceae | Probiotic |
| GCF_002861775.1 | Lactobacillus crispatus | USA | North America | Lactobacillaceae | Probiotic |
| GCF_030227885.1 | Lactobacillus crispatus | USA | North America | Lactobacillaceae | Probiotic |
| GCF_030218685.1 | Lactobacillus crispatus | USA | North America | Lactobacillaceae | Probiotic |
| GCF_002863245.1 | Lactobacillus crispatus | USA | North America | Lactobacillaceae | Probiotic |
| GCF_030226455.1 | Lactobacillus crispatus | USA | North America | Lactobacillaceae | Probiotic |
| GCF_030218845.1 | Lactobacillus crispatus | USA | North America | Lactobacillaceae | Probiotic |
| GCF_030222225.1 | Lactobacillus crispatus | USA | North America | Lactobacillaceae | Probiotic |
| GCF_030218935.1 | Lactobacillus crispatus | USA | North America | Lactobacillaceae | Probiotic |
| GCF_019537355.1 | Lactobacillus crispatus | India | Asia | Lactobacillaceae | Probiotic |
| GCF_000091765.1 | Lactobacillus crispatus | Missing | Europe | Lactobacillaceae | Probiotic |
| GCF_000176975.2 | Lactobacillus crispatus | Iran | Asia | Lactobacillaceae | Probiotic |
| GCF_004361395.1 | Lactobacillus crispatus | Netherlands | Europe | Lactobacillaceae | Probiotic |
| GCF_004361075.1 | Lactobacillus crispatus | Netherlands | Europe | Lactobacillaceae | Probiotic |
| GCF_004361095.1 | Lactobacillus crispatus | Netherlands | Europe | Lactobacillaceae | Probiotic |
| GCF_004361455.1 | Lactobacillus crispatus | Netherlands | Europe | Lactobacillaceae | Probiotic |
| GCF_004361115.1 | Lactobacillus crispatus | Netherlands | Europe | Lactobacillaceae | Probiotic |
| GCF_004361125.1 | Lactobacillus crispatus | Netherlands | Europe | Lactobacillaceae | Probiotic |
| GCF_004361445.1 | Lactobacillus crispatus | Netherlands | Europe | Lactobacillaceae | Probiotic |
| GCF_004361175.1 | Lactobacillus crispatus | Netherlands | Europe | Lactobacillaceae | Probiotic |
| GCF_004361465.1 | Lactobacillus crispatus | Netherlands | Europe | Lactobacillaceae | Probiotic |
| GCF_004361185.1 | Lactobacillus crispatus | Netherlands | Europe | Lactobacillaceae | Probiotic |
| GCF_004361475.1 | Lactobacillus crispatus | Netherlands | Europe | Lactobacillaceae | Probiotic |
| GCF_004361195.1 | Lactobacillus crispatus | Netherlands | Europe | Lactobacillaceae | Probiotic |
| GCF_004361515.1 | Lactobacillus crispatus | Netherlands | Europe | Lactobacillaceae | Probiotic |
| GCF_004361205.1 | Lactobacillus crispatus | Netherlands | Europe | Lactobacillaceae | Probiotic |
| GCF_004361215.1 | Lactobacillus crispatus | Netherlands | Europe | Lactobacillaceae | Probiotic |
| GCF_004361545.1 | Lactobacillus crispatus | Netherlands | Europe | Lactobacillaceae | Probiotic |
| GCF_004361245.1 | Lactobacillus crispatus | Netherlands | Europe | Lactobacillaceae | Probiotic |
| GCF_004361555.1 | Lactobacillus crispatus | Netherlands | Europe | Lactobacillaceae | Probiotic |
| GCF_004361265.1 | Lactobacillus crispatus | Netherlands | Europe | Lactobacillaceae | Probiotic |
| GCF_004361295.1 | Lactobacillus crispatus | Netherlands | Europe | Lactobacillaceae | Probiotic |
| GCF_004361565.1 | Lactobacillus crispatus | Netherlands | Europe | Lactobacillaceae | Probiotic |
| GCF_004361575.1 | Lactobacillus crispatus | Netherlands | Europe | Lactobacillaceae | Probiotic |
| GCF_004361315.1 | Lactobacillus crispatus | Netherlands | Europe | Lactobacillaceae | Probiotic |
| GCF_004361355.1 | Lactobacillus crispatus | Netherlands | Europe | Lactobacillaceae | Probiotic |
| GCF_004361635.1 | Lactobacillus crispatus | Netherlands | Europe | Lactobacillaceae | Probiotic |
| GCF_004361345.1 | Lactobacillus crispatus | Netherlands | Europe | Lactobacillaceae | Probiotic |
| GCF_004361385.1 | Lactobacillus crispatus | Netherlands | Europe | Lactobacillaceae | Probiotic |
| GCF_004361375.1 | Lactobacillus crispatus | Netherlands | Europe | Lactobacillaceae | Probiotic |
| GCF_001563615.1 | Lactobacillus crispatus | Missing | Missing | Lactobacillaceae | Probiotic |
| GCF_016767795.1 | Lactobacillus crispatus | Italy | Europe | Lactobacillaceae | Probiotic |
| GCF_018885325.1 | Lactobacillus crispatus | South Korea | Asia | Lactobacillaceae | Probiotic |
| GCF_008694785.1 | Lactobacillus crispatus | USA | North America | Lactobacillaceae | Probiotic |
| GCF_008694765.1 | Lactobacillus crispatus | USA | North America | Lactobacillaceae | Probiotic |
| GCF_008694875.1 | Lactobacillus crispatus | USA | North America | Lactobacillaceae | Probiotic |
| GCF_008694845.1 | Lactobacillus crispatus | USA | North America | Lactobacillaceae | Probiotic |
| GCF_008694865.1 | Lactobacillus crispatus | USA | North America | Lactobacillaceae | Probiotic |
| GCF_008694885.1 | Lactobacillus crispatus | USA | North America | Lactobacillaceae | Probiotic |
| GCF_008694205.1 | Lactobacillus crispatus | USA | North America | Lactobacillaceae | Probiotic |
| GCF_008694925.1 | Lactobacillus crispatus | USA | North America | Lactobacillaceae | Probiotic |
| GCF_008694935.1 | Lactobacillus crispatus | USA | North America | Lactobacillaceae | Probiotic |
| GCF_008694985.1 | Lactobacillus crispatus | USA | North America | Lactobacillaceae | Probiotic |
| GCF_008694975.1 | Lactobacillus crispatus | USA | North America | Lactobacillaceae | Probiotic |
| GCF_008694775.1 | Lactobacillus crispatus | USA | North America | Lactobacillaceae | Probiotic |
| GCF_008694745.1 | Lactobacillus crispatus | USA | North America | Lactobacillaceae | Probiotic |
| GCF_007713895.1 | Lactobacillus crispatus | USA | North America | Lactobacillaceae | Probiotic |
| GCF_000162315.1 | Lactobacillus crispatus | USA | North America | Lactobacillaceae | Probiotic |
| GCF_000161915.2 | Lactobacillus crispatus | USA | North America | Lactobacillaceae | Probiotic |
| GCF_022454025.1 | Lactobacillus crispatus | USA | North America | Lactobacillaceae | Probiotic |
| GCF_022453955.1 | Lactobacillus crispatus | USA | North America | Lactobacillaceae | Probiotic |
| GCF_022454015.1 | Lactobacillus crispatus | USA | North America | Lactobacillaceae | Probiotic |
| GCF_022454075.1 | Lactobacillus crispatus | USA | North America | Lactobacillaceae | Probiotic |
| GCF_022454115.1 | Lactobacillus crispatus | USA | North America | Lactobacillaceae | Probiotic |
| GCF_022454135.1 | Lactobacillus crispatus | USA | North America | Lactobacillaceae | Probiotic |
| GCF_022454175.1 | Lactobacillus crispatus | USA | North America | Lactobacillaceae | Probiotic |
| GCF_022454205.1 | Lactobacillus crispatus | USA | North America | Lactobacillaceae | Probiotic |
| GCF_020224235.1 | Lactobacillus crispatus | Italy | Europe | Lactobacillaceae | Probiotic |
| GCF_026740115.1 | Lactobacillus crispatus | Italy | Europe | Lactobacillaceae | Probiotic |
| GCF_027695305.1 | Lactobacillus crispatus | China | Asia | Lactobacillaceae | Probiotic |
| GCF_027271175.1 | Lactobacillus crispatus | China | Asia | Lactobacillaceae | Probiotic |
| GCF_017599225.1 | Lactobacillus crispatus | China | Asia | Lactobacillaceae | Probiotic |
| GCF_021278925.1 | Lactobacillus crispatus | China | Asia | Lactobacillaceae | Probiotic |
| GCF_021278945.1 | Lactobacillus crispatus | China | Asia | Lactobacillaceae | Probiotic |
| GCF_020042005.1 | Lactobacillus crispatus | USA | North America | Lactobacillaceae | Probiotic |
| GCF_020042125.1 | Lactobacillus crispatus | USA | North America | Lactobacillaceae | Probiotic |
| GCF_020042225.1 | Lactobacillus crispatus | USA | North America | Lactobacillaceae | Probiotic |
| GCF_016161925.1 | Lactobacillus crispatus | Italy | Europe | Lactobacillaceae | Probiotic |
| GCF_016161975.1 | Lactobacillus crispatus | Italy | Europe | Lactobacillaceae | Probiotic |
| GCF_016161995.1 | Lactobacillus crispatus | Italy | Europe | Lactobacillaceae | Probiotic |
| GCF_016162005.1 | Lactobacillus crispatus | Italy | Europe | Lactobacillaceae | Probiotic |
| GCF_016162045.1 | Lactobacillus crispatus | Italy | Europe | Lactobacillaceae | Probiotic |
| GCF_016162105.1 | Lactobacillus crispatus | Italy | Europe | Lactobacillaceae | Probiotic |
| GCF_016162055.1 | Lactobacillus crispatus | Italy | Europe | Lactobacillaceae | Probiotic |
| GCF_016162065.1 | Lactobacillus crispatus | Italy | Europe | Lactobacillaceae | Probiotic |
| GCF_016162125.1 | Lactobacillus crispatus | Italy | Europe | Lactobacillaceae | Probiotic |
| GCF_016162155.1 | Lactobacillus crispatus | Italy | Europe | Lactobacillaceae | Probiotic |
| GCF_016162165.1 | Lactobacillus crispatus | Italy | Europe | Lactobacillaceae | Probiotic |
| GCF_016162145.1 | Lactobacillus crispatus | Italy | Europe | Lactobacillaceae | Probiotic |
| GCF_016162185.1 | Lactobacillus crispatus | Italy | Europe | Lactobacillaceae | Probiotic |
| GCF_016162195.1 | Lactobacillus crispatus | Italy | Europe | Lactobacillaceae | Probiotic |
| GCF_004103355.1 | Lactobacillus crispatus | China | Asia | Lactobacillaceae | Probiotic |
| GCF_020887095.1 | Lactobacillus crispatus | Japan | Asia | Lactobacillaceae | Probiotic |
| GCF_000160515.1 | Lactobacillus crispatus | USA | North America | Lactobacillaceae | Probiotic |
| GCF_022455895.1 | Lactobacillus crispatus | South Africa | Africa | Lactobacillaceae | Probiotic |
| GCF_022455915.1 | Lactobacillus crispatus | South Africa | Africa | Lactobacillaceae | Probiotic |
| GCF_022455875.1 | Lactobacillus crispatus | South Africa | Africa | Lactobacillaceae | Probiotic |
| GCF_022455855.1 | Lactobacillus crispatus | South Africa | Africa | Lactobacillaceae | Probiotic |
| GCF_022455935.1 | Lactobacillus crispatus | South Africa | Africa | Lactobacillaceae | Probiotic |
| GCF_022455955.1 | Lactobacillus crispatus | South Africa | Africa | Lactobacillaceae | Probiotic |
| GCF_022455975.1 | Lactobacillus crispatus | South Africa | Africa | Lactobacillaceae | Probiotic |
| GCF_022456015.1 | Lactobacillus crispatus | South Africa | Africa | Lactobacillaceae | Probiotic |
| GCF_022455985.1 | Lactobacillus crispatus | South Africa | Africa | Lactobacillaceae | Probiotic |
| GCF_022456035.1 | Lactobacillus crispatus | South Africa | Africa | Lactobacillaceae | Probiotic |
| GCF_001567095.1 | Lactobacillus crispatus | Japan | Asia | Lactobacillaceae | Probiotic |
| GCF_009857395.1 | Lactobacillus crispatus | India | Asia | Lactobacillaceae | Probiotic |
| GCF_009730275.1 | Lactobacillus crispatus | USA | North America | Lactobacillaceae | Probiotic |
| GCF_000301135.1 | Lactobacillus crispatus | Missing | Missing | Lactobacillaceae | Probiotic |
| GCF_000301115.1 | Lactobacillus crispatus | Missing | Missing | Lactobacillaceae | Probiotic |
| GCF_022456175.1 | Lactobacillus crispatus | South Africa | Africa | Lactobacillaceae | Probiotic |
| GCF_000497065.1 | Lactobacillus crispatus | Ireland | Europe | Lactobacillaceae | Probiotic |
| GCF_022456995.1 | Lactobacillus crispatus | USA | North America | Lactobacillaceae | Probiotic |
| GCF_022454285.1 | Lactobacillus crispatus | USA | North America | Lactobacillaceae | Probiotic |
| GCF_022454315.1 | Lactobacillus crispatus | USA | North America | Lactobacillaceae | Probiotic |
| GCF_022454275.1 | Lactobacillus crispatus | USA | North America | Lactobacillaceae | Probiotic |
| GCF_022454355.1 | Lactobacillus crispatus | USA | North America | Lactobacillaceae | Probiotic |
| GCF_022454335.1 | Lactobacillus crispatus | USA | North America | Lactobacillaceae | Probiotic |
| GCF_022454395.1 | Lactobacillus crispatus | USA | North America | Lactobacillaceae | Probiotic |
| GCF_022454385.1 | Lactobacillus crispatus | USA | North America | Lactobacillaceae | Probiotic |
| GCF_022454375.1 | Lactobacillus crispatus | USA | North America | Lactobacillaceae | Probiotic |
| GCF_022454435.1 | Lactobacillus crispatus | USA | North America | Lactobacillaceae | Probiotic |
| GCF_022454495.1 | Lactobacillus crispatus | USA | North America | Lactobacillaceae | Probiotic |
| GCF_022454515.1 | Lactobacillus crispatus | USA | North America | Lactobacillaceae | Probiotic |
| GCF_022454475.1 | Lactobacillus crispatus | USA | North America | Lactobacillaceae | Probiotic |
| GCF_022454535.1 | Lactobacillus crispatus | USA | North America | Lactobacillaceae | Probiotic |
| GCF_022454565.1 | Lactobacillus crispatus | USA | North America | Lactobacillaceae | Probiotic |
| GCF_022454555.1 | Lactobacillus crispatus | USA | North America | Lactobacillaceae | Probiotic |
| GCF_022454655.1 | Lactobacillus crispatus | USA | North America | Lactobacillaceae | Probiotic |
| GCF_022454675.1 | Lactobacillus crispatus | USA | North America | Lactobacillaceae | Probiotic |
| GCF_022454705.1 | Lactobacillus crispatus | USA | North America | Lactobacillaceae | Probiotic |
| GCF_022454615.1 | Lactobacillus crispatus | USA | North America | Lactobacillaceae | Probiotic |
| GCF_022454755.1 | Lactobacillus crispatus | USA | North America | Lactobacillaceae | Probiotic |
| GCF_013778545.1 | Lactobacillus crispatus | Finland | Europe | Lactobacillaceae | Probiotic |
| GCF_014982905.1 | Lactobacillus crispatus | Germany | Europe | Lactobacillaceae | Probiotic |
| GCF_002811165.1 | Lactobacillus crispatus | India | Asia | Lactobacillaceae | Probiotic |
| GCF_009769205.1 | Lactobacillus crispatus | United Kingdom | Europe | Lactobacillaceae | Probiotic |
| GCF_015669875.1 | Lactobacillus crispatus | USA | North America | Lactobacillaceae | Probiotic |
| GCF_015708075.1 | Lactobacillus crispatus | Brazil | South America | Lactobacillaceae | Probiotic |
| GCF_015708105.1 | Lactobacillus crispatus | Brazil | South America | Lactobacillaceae | Probiotic |
| GCF_015708055.1 | Lactobacillus crispatus | Brazil | South America | Lactobacillaceae | Probiotic |
| GCF_015708065.1 | Lactobacillus crispatus | Brazil | South America | Lactobacillaceae | Probiotic |
| GCF_003795065.1 | Lactobacillus crispatus | USA | North America | Lactobacillaceae | Probiotic |
| GCF_022456935.1 | Lactobacillus crispatus | USA | North America | Lactobacillaceae | Probiotic |
| GCF_022454765.1 | Lactobacillus crispatus | USA | North America | Lactobacillaceae | Probiotic |
| GCF_022454815.1 | Lactobacillus crispatus | USA | North America | Lactobacillaceae | Probiotic |
| GCF_022454795.1 | Lactobacillus crispatus | USA | North America | Lactobacillaceae | Probiotic |
| GCF_022454845.1 | Lactobacillus crispatus | USA | North America | Lactobacillaceae | Probiotic |
| GCF_022454835.1 | Lactobacillus crispatus | USA | North America | Lactobacillaceae | Probiotic |
| GCF_022454865.1 | Lactobacillus crispatus | USA | North America | Lactobacillaceae | Probiotic |
| GCF_022454895.1 | Lactobacillus crispatus | USA | North America | Lactobacillaceae | Probiotic |
| GCF_022454915.1 | Lactobacillus crispatus | USA | North America | Lactobacillaceae | Probiotic |
| GCF_022454955.1 | Lactobacillus crispatus | USA | North America | Lactobacillaceae | Probiotic |
| GCF_022454965.1 | Lactobacillus crispatus | USA | North America | Lactobacillaceae | Probiotic |
| GCF_022455005.1 | Lactobacillus crispatus | USA | North America | Lactobacillaceae | Probiotic |
| GCF_022455035.1 | Lactobacillus crispatus | USA | North America | Lactobacillaceae | Probiotic |
| GCF_022455115.1 | Lactobacillus crispatus | USA | North America | Lactobacillaceae | Probiotic |
| GCF_022455055.1 | Lactobacillus crispatus | USA | North America | Lactobacillaceae | Probiotic |
| GCF_022455065.1 | Lactobacillus crispatus | USA | North America | Lactobacillaceae | Probiotic |
| GCF_022455135.1 | Lactobacillus crispatus | USA | North America | Lactobacillaceae | Probiotic |
| GCF_022455185.1 | Lactobacillus crispatus | USA | North America | Lactobacillaceae | Probiotic |
| GCF_022455155.1 | Lactobacillus crispatus | USA | North America | Lactobacillaceae | Probiotic |
| GCF_022455205.1 | Lactobacillus crispatus | USA | North America | Lactobacillaceae | Probiotic |
| GCF_022455175.1 | Lactobacillus crispatus | USA | North America | Lactobacillaceae | Probiotic |
| GCF_022455235.1 | Lactobacillus crispatus | USA | North America | Lactobacillaceae | Probiotic |
| GCF_022455265.1 | Lactobacillus crispatus | USA | North America | Lactobacillaceae | Probiotic |
| GCF_022455295.1 | Lactobacillus crispatus | USA | North America | Lactobacillaceae | Probiotic |
| GCF_022455255.1 | Lactobacillus crispatus | USA | North America | Lactobacillaceae | Probiotic |
| GCF_022455315.1 | Lactobacillus crispatus | USA | North America | Lactobacillaceae | Probiotic |
| GCF_022455325.1 | Lactobacillus crispatus | USA | North America | Lactobacillaceae | Probiotic |
| GCF_022455375.1 | Lactobacillus crispatus | USA | North America | Lactobacillaceae | Probiotic |
| GCF_022455355.1 | Lactobacillus crispatus | USA | North America | Lactobacillaceae | Probiotic |
| GCF_022455395.1 | Lactobacillus crispatus | USA | North America | Lactobacillaceae | Probiotic |
| GCF_022455415.1 | Lactobacillus crispatus | USA | North America | Lactobacillaceae | Probiotic |
| GCF_022455475.1 | Lactobacillus crispatus | USA | North America | Lactobacillaceae | Probiotic |
| GCF_022456295.1 | Lactobacillus crispatus | USA | North America | Lactobacillaceae | Probiotic |
| GCF_022455535.1 | Lactobacillus crispatus | USA | North America | Lactobacillaceae | Probiotic |
| GCF_022455455.1 | Lactobacillus crispatus | USA | North America | Lactobacillaceae | Probiotic |
| GCF_022455515.1 | Lactobacillus crispatus | USA | North America | Lactobacillaceae | Probiotic |
| GCF_022455555.1 | Lactobacillus crispatus | USA | North America | Lactobacillaceae | Probiotic |
| GCF_022455575.1 | Lactobacillus crispatus | USA | North America | Lactobacillaceae | Probiotic |
| GCF_022455595.1 | Lactobacillus crispatus | USA | North America | Lactobacillaceae | Probiotic |
| GCF_022455615.1 | Lactobacillus crispatus | USA | North America | Lactobacillaceae | Probiotic |
| GCF_022455635.1 | Lactobacillus crispatus | USA | North America | Lactobacillaceae | Probiotic |
| GCF_022455675.1 | Lactobacillus crispatus | USA | North America | Lactobacillaceae | Probiotic |
| GCF_022455655.1 | Lactobacillus crispatus | USA | North America | Lactobacillaceae | Probiotic |
| GCF_022455735.1 | Lactobacillus crispatus | USA | North America | Lactobacillaceae | Probiotic |
| GCF_022455705.1 | Lactobacillus crispatus | USA | North America | Lactobacillaceae | Probiotic |
| GCF_022455695.1 | Lactobacillus crispatus | USA | North America | Lactobacillaceae | Probiotic |
| GCF_022455755.1 | Lactobacillus crispatus | USA | North America | Lactobacillaceae | Probiotic |
| GCF_025194115.1 | Lactobacillus crispatus | USA | North America | Lactobacillaceae | Probiotic |
| GCF_025194045.1 | Lactobacillus crispatus | France | Europe | Lactobacillaceae | Probiotic |
| GCF_025194105.1 | Lactobacillus crispatus | United Kingdom | Europe | Lactobacillaceae | Probiotic |
| GCF_025194085.1 | Lactobacillus crispatus | United Kingdom | Europe | Lactobacillaceae | Probiotic |
| GCF_008079315.1 | Lactobacillus crispatus | France | Europe | Lactobacillaceae | Probiotic |
| GCF_004334905.1 | Lactobacillus crispatus | USA | North America | Lactobacillaceae | Probiotic |
| GCF_030869985.1 | Lactobacillus crispatus | Portugal | Europe | Lactobacillaceae | Probiotic |
| GCF_001704465.1 | Lactobacillus crispatus | USA | North America | Lactobacillaceae | Probiotic |
| GCF_009933525.1 | Lactobacillus crispatus | USA | North America | Lactobacillaceae | Probiotic |
| GCF_001700475.1 | Lactobacillus crispatus | USA | North America | Lactobacillaceae | Probiotic |
| GCF_027153465.1 | Lactobacillus crispatus | USA | North America | Lactobacillaceae | Probiotic |
| GCF_027153425.1 | Lactobacillus crispatus | USA | North America | Lactobacillaceae | Probiotic |
| GCF_027153485.1 | Lactobacillus crispatus | USA | North America | Lactobacillaceae | Probiotic |
| GCF_027153525.1 | Lactobacillus crispatus | USA | North America | Lactobacillaceae | Probiotic |
| GCF_016093195.1 | Lactobacillus crispatus | USA | North America | Lactobacillaceae | Probiotic |
| GCF_027153545.1 | Lactobacillus crispatus | USA | North America | Lactobacillaceae | Probiotic |
| GCF_027153565.1 | Lactobacillus crispatus | USA | North America | Lactobacillaceae | Probiotic |
| GCF_027153605.1 | Lactobacillus crispatus | USA | North America | Lactobacillaceae | Probiotic |
| GCF_027153625.1 | Lactobacillus crispatus | USA | North America | Lactobacillaceae | Probiotic |
| GCF_027153645.1 | Lactobacillus crispatus | USA | North America | Lactobacillaceae | Probiotic |
| GCF_027153585.1 | Lactobacillus crispatus | USA | North America | Lactobacillaceae | Probiotic |
| GCF_027153665.1 | Lactobacillus crispatus | USA | North America | Lactobacillaceae | Probiotic |
| GCF_027153715.1 | Lactobacillus crispatus | USA | North America | Lactobacillaceae | Probiotic |
| GCF_027153685.1 | Lactobacillus crispatus | USA | North America | Lactobacillaceae | Probiotic |
| GCF_027153745.1 | Lactobacillus crispatus | USA | North America | Lactobacillaceae | Probiotic |
| GCF_027153755.1 | Lactobacillus crispatus | USA | North America | Lactobacillaceae | Probiotic |
| GCF_027153805.1 | Lactobacillus crispatus | USA | North America | Lactobacillaceae | Probiotic |
| GCF_027153925.1 | Lactobacillus crispatus | USA | North America | Lactobacillaceae | Probiotic |
| GCF_027153965.1 | Lactobacillus crispatus | USA | North America | Lactobacillaceae | Probiotic |
| GCF_027154005.1 | Lactobacillus crispatus | USA | North America | Lactobacillaceae | Probiotic |
| GCF_027154025.1 | Lactobacillus crispatus | USA | North America | Lactobacillaceae | Probiotic |
| GCF_027154035.1 | Lactobacillus crispatus | USA | North America | Lactobacillaceae | Probiotic |
| GCF_027154065.1 | Lactobacillus crispatus | USA | North America | Lactobacillaceae | Probiotic |
| GCF_027154085.1 | Lactobacillus crispatus | USA | North America | Lactobacillaceae | Probiotic |
| GCF_027154125.1 | Lactobacillus crispatus | USA | North America | Lactobacillaceae | Probiotic |
| GCF_027154285.1 | Lactobacillus crispatus | USA | North America | Lactobacillaceae | Probiotic |
| GCF_027154295.1 | Lactobacillus crispatus | USA | North America | Lactobacillaceae | Probiotic |
| GCF_027155005.1 | Lactobacillus crispatus | USA | North America | Lactobacillaceae | Probiotic |
| GCF_027155045.1 | Lactobacillus crispatus | USA | North America | Lactobacillaceae | Probiotic |
| GCF_027155075.1 | Lactobacillus crispatus | USA | North America | Lactobacillaceae | Probiotic |
| GCF_027155105.1 | Lactobacillus crispatus | USA | North America | Lactobacillaceae | Probiotic |
| GCF_027155375.1 | Lactobacillus crispatus | USA | North America | Lactobacillaceae | Probiotic |
| GCF_027155435.1 | Lactobacillus crispatus | USA | North America | Lactobacillaceae | Probiotic |
| GCF_027155425.1 | Lactobacillus crispatus | USA | North America | Lactobacillaceae | Probiotic |
| GCF_027155545.1 | Lactobacillus crispatus | USA | North America | Lactobacillaceae | Probiotic |
| GCF_027155575.1 | Lactobacillus crispatus | USA | North America | Lactobacillaceae | Probiotic |
| GCF_027158685.1 | Lactobacillus crispatus | USA | North America | Lactobacillaceae | Probiotic |
| GCF_027158725.1 | Lactobacillus crispatus | USA | North America | Lactobacillaceae | Probiotic |
| GCF_027158735.1 | Lactobacillus crispatus | USA | North America | Lactobacillaceae | Probiotic |
| GCF_027158705.1 | Lactobacillus crispatus | USA | North America | Lactobacillaceae | Probiotic |
| GCF_027157665.1 | Lactobacillus crispatus | USA | North America | Lactobacillaceae | Probiotic |
| GCF_027158765.1 | Lactobacillus crispatus | USA | North America | Lactobacillaceae | Probiotic |
| GCF_027157565.1 | Lactobacillus crispatus | USA | North America | Lactobacillaceae | Probiotic |
| GCF_027158795.1 | Lactobacillus crispatus | USA | North America | Lactobacillaceae | Probiotic |
| GCF_027158785.1 | Lactobacillus crispatus | USA | North America | Lactobacillaceae | Probiotic |
| GCF_027158845.1 | Lactobacillus crispatus | USA | North America | Lactobacillaceae | Probiotic |
| GCF_027158835.1 | Lactobacillus crispatus | USA | North America | Lactobacillaceae | Probiotic |
| GCF_027158825.1 | Lactobacillus crispatus | USA | North America | Lactobacillaceae | Probiotic |
| GCF_027158885.1 | Lactobacillus crispatus | USA | North America | Lactobacillaceae | Probiotic |
| GCF_027158905.1 | Lactobacillus crispatus | USA | North America | Lactobacillaceae | Probiotic |
| GCF_027158925.1 | Lactobacillus crispatus | USA | North America | Lactobacillaceae | Probiotic |
| GCF_027159065.1 | Lactobacillus crispatus | USA | North America | Lactobacillaceae | Probiotic |
| GCF_027159085.1 | Lactobacillus crispatus | USA | North America | Lactobacillaceae | Probiotic |
| GCF_027159095.1 | Lactobacillus crispatus | USA | North America | Lactobacillaceae | Probiotic |
| GCF_027160525.1 | Lactobacillus crispatus | USA | North America | Lactobacillaceae | Probiotic |
| GCF_027160555.1 | Lactobacillus crispatus | USA | North America | Lactobacillaceae | Probiotic |
| GCF_027160545.1 | Lactobacillus crispatus | USA | North America | Lactobacillaceae | Probiotic |
| GCF_027160595.1 | Lactobacillus crispatus | USA | North America | Lactobacillaceae | Probiotic |
| GCF_027160585.1 | Lactobacillus crispatus | USA | North America | Lactobacillaceae | Probiotic |
| GCF_027160805.1 | Lactobacillus crispatus | USA | North America | Lactobacillaceae | Probiotic |
| GCF_027160825.1 | Lactobacillus crispatus | USA | North America | Lactobacillaceae | Probiotic |
| GCF_027160865.1 | Lactobacillus crispatus | USA | North America | Lactobacillaceae | Probiotic |
| GCF_027160845.1 | Lactobacillus crispatus | USA | North America | Lactobacillaceae | Probiotic |
| GCF_027161085.1 | Lactobacillus crispatus | USA | North America | Lactobacillaceae | Probiotic |
| GCF_027161105.1 | Lactobacillus crispatus | USA | North America | Lactobacillaceae | Probiotic |
| GCF_027161115.1 | Lactobacillus crispatus | USA | North America | Lactobacillaceae | Probiotic |
| GCF_027161125.1 | Lactobacillus crispatus | USA | North America | Lactobacillaceae | Probiotic |
| GCF_027161165.1 | Lactobacillus crispatus | USA | North America | Lactobacillaceae | Probiotic |
| GCF_027583895.1 | Lactobacillus crispatus | USA | North America | Lactobacillaceae | Probiotic |
| GCF_027583915.1 | Lactobacillus crispatus | USA | North America | Lactobacillaceae | Probiotic |
| GCF_027583955.1 | Lactobacillus crispatus | USA | North America | Lactobacillaceae | Probiotic |
| GCF_027583975.1 | Lactobacillus crispatus | USA | North America | Lactobacillaceae | Probiotic |
| GCF_027583995.1 | Lactobacillus crispatus | USA | North America | Lactobacillaceae | Probiotic |
| GCF_027584035.1 | Lactobacillus crispatus | USA | North America | Lactobacillaceae | Probiotic |
| GCF_027584095.1 | Lactobacillus crispatus | USA | North America | Lactobacillaceae | Probiotic |
| GCF_027584215.1 | Lactobacillus crispatus | USA | North America | Lactobacillaceae | Probiotic |
| GCF_027153305.1 | Lactobacillus crispatus | USA | North America | Lactobacillaceae | Probiotic |
| GCF_027153355.1 | Lactobacillus crispatus | USA | North America | Lactobacillaceae | Probiotic |
| GCF_027153325.1 | Lactobacillus crispatus | USA | North America | Lactobacillaceae | Probiotic |
| GCF_027156725.1 | Lactobacillus crispatus | USA | North America | Lactobacillaceae | Probiotic |
| GCF_027156705.1 | Lactobacillus crispatus | USA | North America | Lactobacillaceae | Probiotic |
| GCF_027153785.1 | Lactobacillus crispatus | USA | North America | Lactobacillaceae | Probiotic |
| GCF_027153705.1 | Lactobacillus crispatus | USA | North America | Lactobacillaceae | Probiotic |
| GCF_027153945.1 | Lactobacillus crispatus | USA | North America | Lactobacillaceae | Probiotic |
| GCF_027153845.1 | Lactobacillus crispatus | USA | North America | Lactobacillaceae | Probiotic |
| GCF_027153815.1 | Lactobacillus crispatus | USA | North America | Lactobacillaceae | Probiotic |
| GCF_027153255.1 | Lactobacillus crispatus | USA | North America | Lactobacillaceae | Probiotic |
| GCF_027153385.1 | Lactobacillus crispatus | USA | North America | Lactobacillaceae | Probiotic |
| GCF_027158945.1 | Lactobacillus crispatus | USA | North America | Lactobacillaceae | Probiotic |
| GCF_027159005.1 | Lactobacillus crispatus | USA | North America | Lactobacillaceae | Probiotic |
| GCF_027158985.1 | Lactobacillus crispatus | USA | North America | Lactobacillaceae | Probiotic |
| GCF_027159025.1 | Lactobacillus crispatus | USA | North America | Lactobacillaceae | Probiotic |
| GCF_027159035.1 | Lactobacillus crispatus | USA | North America | Lactobacillaceae | Probiotic |
| GCF_027160095.1 | Lactobacillus crispatus | USA | North America | Lactobacillaceae | Probiotic |
| GCF_027160085.1 | Lactobacillus crispatus | USA | North America | Lactobacillaceae | Probiotic |
| GCF_027160165.1 | Lactobacillus crispatus | USA | North America | Lactobacillaceae | Probiotic |
| GCF_027160215.1 | Lactobacillus crispatus | USA | North America | Lactobacillaceae | Probiotic |
| GCF_027160225.1 | Lactobacillus crispatus | USA | North America | Lactobacillaceae | Probiotic |
| GCF_027152805.1 | Lactobacillus crispatus | USA | North America | Lactobacillaceae | Probiotic |
| GCF_027152845.1 | Lactobacillus crispatus | USA | North America | Lactobacillaceae | Probiotic |
| GCF_027152885.1 | Lactobacillus crispatus | USA | North America | Lactobacillaceae | Probiotic |
| GCF_027160325.1 | Lactobacillus crispatus | USA | North America | Lactobacillaceae | Probiotic |
| GCF_027160285.1 | Lactobacillus crispatus | USA | North America | Lactobacillaceae | Probiotic |
| GCF_008868575.1 | Lactobacillus crispatus | Missing | Missing | Lactobacillaceae | Probiotic |
| GCF_014654865.1 | Lactobacillus crispatus | Italy | Europe | Lactobacillaceae | Probiotic |
| GCF_013456995.1 | Lactobacillus crispatus | China | Asia | Lactobacillaceae | Probiotic |
| GCF_016901535.1 | Lactobacillus crispatus | Missing | Missing | Lactobacillaceae | Probiotic |
| GCF_027680585.1 | Lactobacillus crispatus | China | Asia | Lactobacillaceae | Probiotic |
| GCF_027682305.1 | Lactobacillus crispatus | China | Asia | Lactobacillaceae | Probiotic |
| GCF_003971565.1 | Lactobacillus crispatus | South Korea | Asia | Lactobacillaceae | Probiotic |
| GCF_019278075.1 | Lactobacillus crispatus | India | Asia | Lactobacillaceae | Probiotic |
| GCF_019278055.1 | Lactobacillus crispatus | India | Asia | Lactobacillaceae | Probiotic |
| GCF_019278135.1 | Lactobacillus crispatus | India | Asia | Lactobacillaceae | Probiotic |
| GCF_019278105.1 | Lactobacillus crispatus | India | Asia | Lactobacillaceae | Probiotic |
| GCF_022456975.1 | Lactobacillus crispatus | USA | North America | Lactobacillaceae | Probiotic |
| GCF_000177575.1 | Lactobacillus crispatus | Missing | Missing | Lactobacillaceae | Probiotic |
| GCF_000466885.3 | Lactobacillus crispatus | Russia | Europe | Lactobacillaceae | Probiotic |
| GCF_013487905.1 | Lactobacillus crispatus | USA | North America | Lactobacillaceae | Probiotic |
| GCF_030373605.1 | Lactobacillus crispatus | Czech Republic | Europe | Lactobacillaceae | Probiotic |
| GCF_000162255.1 | Lactobacillus crispatus | China | Asia | Lactobacillaceae | Probiotic |
| GCF_019277925.1 | Lactobacillus crispatus | India | Asia | Lactobacillaceae | Probiotic |
| GCF_022456575.1 | Lactobacillus crispatus | South Africa | Africa | Lactobacillaceae | Probiotic |
| GCF_022456595.1 | Lactobacillus crispatus | South Africa | Africa | Lactobacillaceae | Probiotic |
| GCF_022456605.1 | Lactobacillus crispatus | South Africa | Africa | Lactobacillaceae | Probiotic |
| GCF_022456655.1 | Lactobacillus crispatus | South Africa | Africa | Lactobacillaceae | Probiotic |
| GCF_022456705.1 | Lactobacillus crispatus | South Africa | Africa | Lactobacillaceae | Probiotic |
| GCF_022456735.1 | Lactobacillus crispatus | South Africa | Africa | Lactobacillaceae | Probiotic |
| GCF_022456695.1 | Lactobacillus crispatus | South Africa | Africa | Lactobacillaceae | Probiotic |
| GCF_022456755.1 | Lactobacillus crispatus | South Africa | Africa | Lactobacillaceae | Probiotic |
| GCF_022456775.1 | Lactobacillus crispatus | South Africa | Africa | Lactobacillaceae | Probiotic |
| GCF_022456835.1 | Lactobacillus crispatus | South Africa | Africa | Lactobacillaceae | Probiotic |
| GCF_022456795.1 | Lactobacillus crispatus | South Africa | Africa | Lactobacillaceae | Probiotic |
| GCF_022456875.1 | Lactobacillus crispatus | South Africa | Africa | Lactobacillaceae | Probiotic |
| GCF_022456895.1 | Lactobacillus crispatus | South Africa | Africa | Lactobacillaceae | Probiotic |
| GCF_902386155.1 | Lactobacillus crispatus | Ireland | Europe | Lactobacillaceae | Probiotic |
| GCF_008694755.1 | Lactobacillus crispatus | USA | North America | Lactobacillaceae | Probiotic |
| GCF_029010235.1 | Lactobacillus gasseri | USA | North America | Lactobacillaceae | Probiotic |
| GCF_029011455.1 | Lactobacillus gasseri | USA | North America | Lactobacillaceae | Probiotic |
| GCF_029011785.1 | Lactobacillus gasseri | USA | North America | Lactobacillaceae | Probiotic |
| GCF_029011805.1 | Lactobacillus gasseri | USA | North America | Lactobacillaceae | Probiotic |
| GCF_030213495.1 | Lactobacillus gasseri | USA | North America | Lactobacillaceae | Probiotic |
| GCF_030224355.1 | Lactobacillus gasseri | USA | North America | Lactobacillaceae | Probiotic |
| GCF_033803995.1 | Lactobacillus gasseri | USA | North America | Lactobacillaceae | Probiotic |
| GCF_033803965.1 | Lactobacillus gasseri | USA | North America | Lactobacillaceae | Probiotic |
| GCF_030216645.1 | Lactobacillus gasseri | USA | North America | Lactobacillaceae | Probiotic |
| GCF_030216925.1 | Lactobacillus gasseri | USA | North America | Lactobacillaceae | Probiotic |
| GCF_032432755.1 | Lactobacillus gasseri | USA | North America | Lactobacillaceae | Probiotic |
| GCF_032377295.1 | Lactobacillus gasseri | USA | North America | Lactobacillaceae | Probiotic |
| GCF_033804545.1 | Lactobacillus gasseri | USA | North America | Lactobacillaceae | Probiotic |
| GCF_033804425.1 | Lactobacillus gasseri | USA | North America | Lactobacillaceae | Probiotic |
| GCF_033804445.1 | Lactobacillus gasseri | USA | North America | Lactobacillaceae | Probiotic |
| GCF_007786195.1 | Lactobacillus gasseri | USA | North America | Lactobacillaceae | Probiotic |
| GCF_007785975.1 | Lactobacillus gasseri | USA | North America | Lactobacillaceae | Probiotic |
| GCF_030224655.1 | Lactobacillus gasseri | USA | North America | Lactobacillaceae | Probiotic |
| GCF_033803545.1 | Lactobacillus gasseri | USA | North America | Lactobacillaceae | Probiotic |
| GCF_032432735.1 | Lactobacillus gasseri | USA | North America | Lactobacillaceae | Probiotic |
| GCF_032432775.1 | Lactobacillus gasseri | USA | North America | Lactobacillaceae | Probiotic |
| GCF_032377135.1 | Lactobacillus gasseri | USA | North America | Lactobacillaceae | Probiotic |
| GCF_030217385.1 | Lactobacillus gasseri | USA | North America | Lactobacillaceae | Probiotic |
| GCF_030217485.1 | Lactobacillus gasseri | USA | North America | Lactobacillaceae | Probiotic |
| GCF_030226605.1 | Lactobacillus gasseri | USA | North America | Lactobacillaceae | Probiotic |
| GCF_030217755.1 | Lactobacillus gasseri | USA | North America | Lactobacillaceae | Probiotic |
| GCF_002863445.1 | Lactobacillus gasseri | USA | North America | Lactobacillaceae | Probiotic |
| GCF_030218505.1 | Lactobacillus gasseri | USA | North America | Lactobacillaceae | Probiotic |
| GCF_007785995.1 | Lactobacillus gasseri | USA | North America | Lactobacillaceae | Probiotic |
| GCF_002863455.1 | Lactobacillus gasseri | USA | North America | Lactobacillaceae | Probiotic |
| GCF_002884735.1 | Lactobacillus gasseri | USA | North America | Lactobacillaceae | Probiotic |
| GCF_002940965.1 | Lactobacillus gasseri | USA | North America | Lactobacillaceae | Probiotic |
| GCF_002863425.1 | Lactobacillus gasseri | USA | North America | Lactobacillaceae | Probiotic |
| GCF_027677045.1 | Lactobacillus gasseri | China | Asia | Lactobacillaceae | Probiotic |
| GCF_003437055.1 | Lactobacillus gasseri | China | Asia | Lactobacillaceae | Probiotic |
| GCF_035621655.1 | Lactobacillus gasseri | China | Asia | Lactobacillaceae | Probiotic |
| GCF_000177035.2 | Lactobacillus gasseri | USA | North America | Lactobacillaceae | Probiotic |
| GCF_000176995.2 | Lactobacillus gasseri | USA | North America | Lactobacillaceae | Probiotic |
| GCF_017565825.1 | Lactobacillus gasseri | USA | North America | Lactobacillaceae | Probiotic |
| GCF_001546525.1 | Lactobacillus gasseri | USA | North America | Lactobacillaceae | Probiotic |
| GCF_018728605.1 | Lactobacillus gasseri | Spain | Europe | Lactobacillaceae | Probiotic |
| GCF_028872155.1 | Lactobacillus gasseri | South Korea | Asia | Lactobacillaceae | Probiotic |
| GCF_020991205.1 | Lactobacillus gasseri | USA | North America | Lactobacillaceae | Probiotic |
| GCF_020991185.1 | Lactobacillus gasseri | USA | North America | Lactobacillaceae | Probiotic |
| GCF_020995385.1 | Lactobacillus gasseri | USA | North America | Lactobacillaceae | Probiotic |
| GCF_030876865.1 | Lactobacillus gasseri | Missing | North America | Lactobacillaceae | Probiotic |
| GCF_022642475.1 | Lactobacillus gasseri | Canada | North America | Lactobacillaceae | Probiotic |
| GCF_003315575.1 | Lactobacillus gasseri | USA | North America | Lactobacillaceae | Probiotic |
| GCF_003307315.1 | Lactobacillus gasseri | Missing | Missing | Lactobacillaceae | Probiotic |
| GCF_030704365.1 | Lactobacillus gasseri | South Korea | Asia | Lactobacillaceae | Probiotic |
| GCF_017498665.1 | Lactobacillus gasseri | South Korea | Asia | Lactobacillaceae | Probiotic |
| GCF_017840575.1 | Lactobacillus gasseri | South Korea | Asia | Lactobacillaceae | Probiotic |
| GCF_017638885.1 | Lactobacillus gasseri | South Korea | Asia | Lactobacillaceae | Probiotic |
| GCF_018389265.1 | Lactobacillus gasseri | USA | North America | Lactobacillaceae | Probiotic |
| GCF_013363915.1 | Lactobacillus gasseri | South Korea | Asia | Lactobacillaceae | Probiotic |
| GCF_002287905.1 | Lactobacillus gasseri | Missing | Missing | Lactobacillaceae | Probiotic |
| GCF_037100225.1 | Lactobacillus gasseri | South Korea | Asia | Lactobacillaceae | Probiotic |
| GCF_024463435.1 | Lactobacillus gasseri | USA | North America | Lactobacillaceae | Probiotic |
| GCF_037414355.1 | Lactobacillus gasseri | South Korea | Asia | Lactobacillaceae | Probiotic |
| GCF_022456925.1 | Lactobacillus gasseri | USA | North America | Lactobacillaceae | Probiotic |
| GCF_000283135.1 | Lactobacillus gasseri | Spain | Europe | Lactobacillaceae | Probiotic |
| GCF_019192945.1 | Lactobacillus gasseri | Spain | Europe | Lactobacillaceae | Probiotic |
| GCF_027584195.1 | Lactobacillus gasseri | USA | North America | Lactobacillaceae | Probiotic |
| GCF_008868535.1 | Lactobacillus gasseri | Missing | Missing | Lactobacillaceae | Probiotic |
| GCF_014654855.1 | Lactobacillus gasseri | Italy | Europe | Lactobacillaceae | Probiotic |
| GCF_002003555.1 | Lactobacillus gasseri | Italy | Europe | Lactobacillaceae | Probiotic |
| GCF_002007185.1 | Lactobacillus gasseri | Italy | Europe | Lactobacillaceae | Probiotic |
| GCF_027681405.1 | Lactobacillus gasseri | China | Asia | Lactobacillaceae | Probiotic |
| GCF_027681485.1 | Lactobacillus gasseri | China | Asia | Lactobacillaceae | Probiotic |
| GCF_027685305.1 | Lactobacillus gasseri | China | Asia | Lactobacillaceae | Probiotic |
| GCF_028864305.1 | Lactobacillus gasseri | Russia | Europe | Lactobacillaceae | Probiotic |
| GCF_001068345.1 | Lactobacillus gasseri | USA | North America | Lactobacillaceae | Probiotic |
| GCF_001066235.1 | Lactobacillus gasseri | USA | North America | Lactobacillaceae | Probiotic |
| GCF_006981945.1 | Lactobacillus gasseri | Canada | North America | Lactobacillaceae | Probiotic |
| GCF_006982025.1 | Lactobacillus gasseri | Canada | North America | Lactobacillaceae | Probiotic |
| GCF_001676665.1 | Lactobacillus gasseri | South Korea | Asia | Lactobacillaceae | Probiotic |
| GCF_002158885.1 | Lactobacillus gasseri | South Korea | Asia | Lactobacillaceae | Probiotic |
| GCF_001063505.1 | Lactobacillus gasseri | USA | North America | Lactobacillaceae | Probiotic |
| GCF_001064985.1 | Lactobacillus gasseri | USA | North America | Lactobacillaceae | Probiotic |
| GCF_001063065.1 | Lactobacillus gasseri | USA | North America | Lactobacillaceae | Probiotic |
| GCF_001063045.1 | Lactobacillus gasseri | USA | North America | Lactobacillaceae | Probiotic |
| GCF_000175055.1 | Lactobacillus gasseri | Missing | Missing | Lactobacillaceae | Probiotic |
| GCF_000439915.2 | Lactobacillus gasseri | Russia | Europe | Lactobacillaceae | Probiotic |
| GCF_028864285.1 | Lactobacillus gasseri | Russia | Europe | Lactobacillaceae | Probiotic |
| GCF_015546835.1 | Lactobacillus gasseri | USA | North America | Lactobacillaceae | Probiotic |
| GCF_925297925.1 | Lactobacillus gasseri | Missing | Missing | Lactobacillaceae | Probiotic |
| GCF_902167745.1 | Lactobacillus gasseri | Missing | Missing | Lactobacillaceae | Probiotic |
| GCF_902386655.1 | Lactobacillus gasseri | Missing | Missing | Lactobacillaceae | Probiotic |
| GCF_902399865.1 | Lactobacillus gasseri | China | Asia | Lactobacillaceae | Probiotic |
| GCF_900452355.1 | Lactobacillus gasseri | United Kingdom | Europe | Lactobacillaceae | Probiotic |
| GCF_015698105.1 | Lactobacillus helveticus | Japan | Asia | Lactobacillaceae | Probiotic |
| GCF_013280815.1 | Lactobacillus helveticus | China | Asia | Lactobacillaceae | Probiotic |
| GCF_015698085.1 | Lactobacillus helveticus | Japan | Asia | Lactobacillaceae | Probiotic |
| GCF_024927945.1 | Lactobacillus helveticus | China | Asia | Lactobacillaceae | Probiotic |
| GCF_011392295.1 | Lactobacillus helveticus | Italy | Europe | Lactobacillaceae | Probiotic |
| GCF_011392325.1 | Lactobacillus helveticus | Italy | Europe | Lactobacillaceae | Probiotic |
| GCF_011392375.1 | Lactobacillus helveticus | Italy | Europe | Lactobacillaceae | Probiotic |
| GCF_011392415.1 | Lactobacillus helveticus | Italy | Europe | Lactobacillaceae | Probiotic |
| GCF_002287645.1 | Lactobacillus helveticus | Italy | Europe | Lactobacillaceae | Probiotic |
| GCF_011392385.1 | Lactobacillus helveticus | Italy | Europe | Lactobacillaceae | Probiotic |
| GCF_019455965.1 | Lactobacillus helveticus | Sudan | Africa | Lactobacillaceae | Probiotic |
| GCF_019455865.1 | Lactobacillus helveticus | China | Asia | Lactobacillaceae | Probiotic |
| GCF_019455925.1 | Lactobacillus helveticus | Germany | Europe | Lactobacillaceae | Probiotic |
| GCF_019455905.1 | Lactobacillus helveticus | Germany | Europe | Lactobacillaceae | Probiotic |
| GCF_019455975.1 | Lactobacillus helveticus | Germany | Europe | Lactobacillaceae | Probiotic |
| GCF_019456005.1 | Lactobacillus helveticus | Germany | Europe | Lactobacillaceae | Probiotic |
| GCF_019456065.1 | Lactobacillus helveticus | Germany | Europe | Lactobacillaceae | Probiotic |
| GCF_019456025.1 | Lactobacillus helveticus | Germany | Europe | Lactobacillaceae | Probiotic |
| GCF_015709185.1 | Lactobacillus helveticus | China | Asia | Lactobacillaceae | Probiotic |
| GCF_003545995.1 | Lactobacillus helveticus | Tajikistan | Asia | Lactobacillaceae | Probiotic |
| GCF_022832545.1 | Lactobacillus helveticus | China | Asia | Lactobacillaceae | Probiotic |
| GCF_022811585.1 | Lactobacillus helveticus | China | Asia | Lactobacillaceae | Probiotic |
| GCF_000165775.1 | Lactobacillus helveticus | France | Europe | Lactobacillaceae | Probiotic |
| GCF_019295325.1 | Lactobacillus helveticus | USA | North America | Lactobacillaceae | Probiotic |
| GCF_029888175.1 | Lactobacillus helveticus | USA | North America | Lactobacillaceae | Probiotic |
| GCF_018967085.1 | Lactobacillus helveticus | Russia | Europe | Lactobacillaceae | Probiotic |
| GCF_021229065.1 | Lactobacillus helveticus | Bulgaria | Europe | Lactobacillaceae | Probiotic |
| GCF_001006025.1 | Lactobacillus helveticus | China | Asia | Lactobacillaceae | Probiotic |
| GCF_002532085.1 | Lactobacillus helveticus | Croatia | Europe | Lactobacillaceae | Probiotic |
| GCF_000765455.1 | Lactobacillus helveticus | Italy | Europe | Lactobacillaceae | Probiotic |
| GCF_009498395.1 | Lactobacillus helveticus | China | Asia | Lactobacillaceae | Probiotic |
| GCF_015698125.1 | Lactobacillus helveticus | Japan | Asia | Lactobacillaceae | Probiotic |
| GCF_019455785.1 | Lactobacillus helveticus | United Kingdom | Europe | Lactobacillaceae | Probiotic |
| GCF_003610975.1 | Lactobacillus helveticus | Bulgaria | Europe | Lactobacillaceae | Probiotic |
| GCF_003955865.1 | Lactobacillus helveticus | South Korea | Asia | Lactobacillaceae | Probiotic |
| GCF_001572815.1 | Lactobacillus helveticus | Italy | Europe | Lactobacillaceae | Probiotic |
| GCF_001572805.1 | Lactobacillus helveticus | Italy | Europe | Lactobacillaceae | Probiotic |
| GCF_019455835.1 | Lactobacillus helveticus | Benin | Africa | Lactobacillaceae | Probiotic |
| GCF_019455825.1 | Lactobacillus helveticus | Benin | Africa | Lactobacillaceae | Probiotic |
| GCF_000961015.1 | Lactobacillus helveticus | China | Asia | Lactobacillaceae | Probiotic |
| GCF_034044615.1 | Lactobacillus helveticus | Russia | Europe | Lactobacillaceae | Probiotic |
| GCF_034045205.1 | Lactobacillus helveticus | Russia | Europe | Lactobacillaceae | Probiotic |
| GCF_035284085.1 | Lactobacillus helveticus | Russia | Europe | Lactobacillaceae | Probiotic |
| GCF_034045235.1 | Lactobacillus helveticus | Russia | Europe | Lactobacillaceae | Probiotic |
| GCF_023893735.1 | Lactobacillus helveticus | Russia | Europe | Lactobacillaceae | Probiotic |
| GCF_024329685.1 | Lactobacillus helveticus | Singapore | Asia | Lactobacillaceae | Probiotic |
| GCF_018408455.1 | Lactobacillus helveticus | Japan | Asia | Lactobacillaceae | Probiotic |
| GCF_015698245.1 | Lactobacillus helveticus | Japan | Asia | Lactobacillaceae | Probiotic |
| GCF_015698205.1 | Lactobacillus helveticus | Japan | Asia | Lactobacillaceae | Probiotic |
| GCF_015698185.1 | Lactobacillus helveticus | Japan | Asia | Lactobacillaceae | Probiotic |
| GCF_015698165.1 | Lactobacillus helveticus | Japan | Asia | Lactobacillaceae | Probiotic |
| GCF_015698145.1 | Lactobacillus helveticus | Japan | Asia | Lactobacillaceae | Probiotic |
| GCF_013280655.1 | Lactobacillus helveticus | China | Asia | Lactobacillaceae | Probiotic |
| GCF_013280625.1 | Lactobacillus helveticus | China | Asia | Lactobacillaceae | Probiotic |
| GCF_013280665.1 | Lactobacillus helveticus | China | Asia | Lactobacillaceae | Probiotic |
| GCF_013280675.1 | Lactobacillus helveticus | China | Asia | Lactobacillaceae | Probiotic |
| GCF_013280615.1 | Lactobacillus helveticus | China | Asia | Lactobacillaceae | Probiotic |
| GCF_013280715.1 | Lactobacillus helveticus | China | Asia | Lactobacillaceae | Probiotic |
| GCF_013280735.1 | Lactobacillus helveticus | China | Asia | Lactobacillaceae | Probiotic |
| GCF_013280775.1 | Lactobacillus helveticus | China | Asia | Lactobacillaceae | Probiotic |
| GCF_013280755.1 | Lactobacillus helveticus | China | Asia | Lactobacillaceae | Probiotic |
| GCF_013280765.1 | Lactobacillus helveticus | China | Asia | Lactobacillaceae | Probiotic |
| GCF_013280825.1 | Lactobacillus helveticus | China | Asia | Lactobacillaceae | Probiotic |
| GCF_013280875.1 | Lactobacillus helveticus | China | Asia | Lactobacillaceae | Probiotic |
| GCF_013280855.1 | Lactobacillus helveticus | China | Asia | Lactobacillaceae | Probiotic |
| GCF_013280865.1 | Lactobacillus helveticus | China | Asia | Lactobacillaceae | Probiotic |
| GCF_013280915.1 | Lactobacillus helveticus | China | Asia | Lactobacillaceae | Probiotic |
| GCF_013280925.1 | Lactobacillus helveticus | China | Asia | Lactobacillaceae | Probiotic |
| GCF_013280955.1 | Lactobacillus helveticus | China | Asia | Lactobacillaceae | Probiotic |
| GCF_013280975.1 | Lactobacillus helveticus | China | Asia | Lactobacillaceae | Probiotic |
| GCF_013280985.1 | Lactobacillus helveticus | China | Asia | Lactobacillaceae | Probiotic |
| GCF_013281005.1 | Lactobacillus helveticus | China | Asia | Lactobacillaceae | Probiotic |
| GCF_013280995.1 | Lactobacillus helveticus | China | Asia | Lactobacillaceae | Probiotic |
| GCF_013281095.1 | Lactobacillus helveticus | China | Asia | Lactobacillaceae | Probiotic |
| GCF_013281075.1 | Lactobacillus helveticus | China | Asia | Lactobacillaceae | Probiotic |
| GCF_013281055.1 | Lactobacillus helveticus | China | Asia | Lactobacillaceae | Probiotic |
| GCF_013281085.1 | Lactobacillus helveticus | China | Asia | Lactobacillaceae | Probiotic |
| GCF_013281065.1 | Lactobacillus helveticus | China | Asia | Lactobacillaceae | Probiotic |
| GCF_013281165.1 | Lactobacillus helveticus | China | Asia | Lactobacillaceae | Probiotic |
| GCF_013281155.1 | Lactobacillus helveticus | China | Asia | Lactobacillaceae | Probiotic |
| GCF_013281175.1 | Lactobacillus helveticus | China | Asia | Lactobacillaceae | Probiotic |
| GCF_013281185.1 | Lactobacillus helveticus | China | Asia | Lactobacillaceae | Probiotic |
| GCF_013281205.1 | Lactobacillus helveticus | China | Asia | Lactobacillaceae | Probiotic |
| GCF_013281295.1 | Lactobacillus helveticus | China | Asia | Lactobacillaceae | Probiotic |
| GCF_013281265.1 | Lactobacillus helveticus | China | Asia | Lactobacillaceae | Probiotic |
| GCF_013281285.1 | Lactobacillus helveticus | China | Asia | Lactobacillaceae | Probiotic |
| GCF_013281255.1 | Lactobacillus helveticus | China | Asia | Lactobacillaceae | Probiotic |
| GCF_013281315.1 | Lactobacillus helveticus | China | Asia | Lactobacillaceae | Probiotic |
| GCF_013281355.1 | Lactobacillus helveticus | China | Asia | Lactobacillaceae | Probiotic |
| GCF_013281415.1 | Lactobacillus helveticus | China | Asia | Lactobacillaceae | Probiotic |
| GCF_013281365.1 | Lactobacillus helveticus | China | Asia | Lactobacillaceae | Probiotic |
| GCF_013281405.1 | Lactobacillus helveticus | China | Asia | Lactobacillaceae | Probiotic |
| GCF_013281395.1 | Lactobacillus helveticus | China | Asia | Lactobacillaceae | Probiotic |
| GCF_013281455.1 | Lactobacillus helveticus | China | Asia | Lactobacillaceae | Probiotic |
| GCF_013281465.1 | Lactobacillus helveticus | China | Asia | Lactobacillaceae | Probiotic |
| GCF_013281495.1 | Lactobacillus helveticus | China | Asia | Lactobacillaceae | Probiotic |
| GCF_013281505.1 | Lactobacillus helveticus | China | Asia | Lactobacillaceae | Probiotic |
| GCF_013281475.1 | Lactobacillus helveticus | China | Asia | Lactobacillaceae | Probiotic |
| GCF_013281575.1 | Lactobacillus helveticus | China | Asia | Lactobacillaceae | Probiotic |
| GCF_013281555.1 | Lactobacillus helveticus | China | Asia | Lactobacillaceae | Probiotic |
| GCF_013281585.1 | Lactobacillus helveticus | China | Asia | Lactobacillaceae | Probiotic |
| GCF_013281635.1 | Lactobacillus helveticus | Mongolia | Asia | Lactobacillaceae | Probiotic |
| GCF_013281605.1 | Lactobacillus helveticus | Mongolia | Asia | Lactobacillaceae | Probiotic |
| GCF_013281645.1 | Lactobacillus helveticus | Mongolia | Asia | Lactobacillaceae | Probiotic |
| GCF_013281695.1 | Lactobacillus helveticus | Mongolia | Asia | Lactobacillaceae | Probiotic |
| GCF_013281675.1 | Lactobacillus helveticus | China | Asia | Lactobacillaceae | Probiotic |
| GCF_013281685.1 | Lactobacillus helveticus | China | Asia | Lactobacillaceae | Probiotic |
| GCF_013281755.1 | Lactobacillus helveticus | China | Asia | Lactobacillaceae | Probiotic |
| GCF_013281795.1 | Lactobacillus helveticus | China | Asia | Lactobacillaceae | Probiotic |
| GCF_004114755.1 | Lactobacillus helveticus | South Korea | Asia | Lactobacillaceae | Probiotic |
| GCF_017565805.1 | Lactobacillus helveticus | USA | North America | Lactobacillaceae | Probiotic |
| GCF_018967005.1 | Lactobacillus helveticus | Russia | Europe | Lactobacillaceae | Probiotic |
| GCF_000525715.1 | Lactobacillus helveticus | China | Asia | Lactobacillaceae | Probiotic |
| GCF_015698065.1 | Lactobacillus helveticus | Missing | Asia | Lactobacillaceae | Probiotic |
| GCF_000189515.1 | Lactobacillus helveticus | China | Asia | Lactobacillaceae | Probiotic |
| GCF_002849915.1 | Lactobacillus helveticus | Switzerland | Europe | Lactobacillaceae | Probiotic |
| GCF_003204055.1 | Lactobacillus helveticus | Switzerland | Europe | Lactobacillaceae | Probiotic |
| GCF_002849935.1 | Lactobacillus helveticus | Switzerland | Europe | Lactobacillaceae | Probiotic |
| GCF_003203975.1 | Lactobacillus helveticus | Switzerland | Europe | Lactobacillaceae | Probiotic |
| GCF_003203915.1 | Lactobacillus helveticus | Switzerland | Europe | Lactobacillaceae | Probiotic |
| GCF_003203815.1 | Lactobacillus helveticus | Switzerland | Europe | Lactobacillaceae | Probiotic |
| GCF_002849955.1 | Lactobacillus helveticus | Switzerland | Europe | Lactobacillaceae | Probiotic |
| GCF_003203795.1 | Lactobacillus helveticus | Switzerland | Europe | Lactobacillaceae | Probiotic |
| GCF_003233715.1 | Lactobacillus helveticus | Switzerland | Europe | Lactobacillaceae | Probiotic |
| GCF_003203905.1 | Lactobacillus helveticus | Switzerland | Europe | Lactobacillaceae | Probiotic |
| GCF_003203955.1 | Lactobacillus helveticus | Switzerland | Europe | Lactobacillaceae | Probiotic |
| GCF_003203985.1 | Lactobacillus helveticus | Switzerland | Europe | Lactobacillaceae | Probiotic |
| GCF_003204025.1 | Lactobacillus helveticus | Switzerland | Europe | Lactobacillaceae | Probiotic |
| GCF_003203805.1 | Lactobacillus helveticus | Switzerland | Europe | Lactobacillaceae | Probiotic |
| GCF_003203875.1 | Lactobacillus helveticus | Switzerland | Europe | Lactobacillaceae | Probiotic |
| GCF_003203895.1 | Lactobacillus helveticus | Switzerland | Europe | Lactobacillaceae | Probiotic |
| GCF_005864165.1 | Lactobacillus helveticus | Switzerland | Europe | Lactobacillaceae | Probiotic |
| GCF_003053085.1 | Lactobacillus helveticus | USA | North America | Lactobacillaceae | Probiotic |
| GCF_003061825.1 | Lactobacillus helveticus | USA | North America | Lactobacillaceae | Probiotic |
| GCF_003053205.1 | Lactobacillus helveticus | USA | North America | Lactobacillaceae | Probiotic |
| GCF_003061845.1 | Lactobacillus helveticus | USA | North America | Lactobacillaceae | Probiotic |
| GCF_000015385.1 | Lactobacillus helveticus | Switzerland | Europe | Lactobacillaceae | Probiotic |
| GCF_009881115.1 | Lactobacillus helveticus | Indonesia | Asia | Lactobacillaceae | Probiotic |
| GCF_009901645.1 | Lactobacillus helveticus | Indonesia | Asia | Lactobacillaceae | Probiotic |
| GCF_001702095.1 | Lactobacillus helveticus | Russia | Europe | Lactobacillaceae | Probiotic |
| GCF_001746265.1 | Lactobacillus helveticus | Russia | Europe | Lactobacillaceae | Probiotic |
| GCF_000422165.1 | Lactobacillus helveticus | USA | North America | Lactobacillaceae | Probiotic |
| GCF_025191335.1 | Lactobacillus helveticus | France | Europe | Lactobacillaceae | Probiotic |
| GCF_000493515.1 | Lactobacillus helveticus | France | Europe | Lactobacillaceae | Probiotic |
| GCF_000493455.1 | Lactobacillus helveticus | France | Europe | Lactobacillaceae | Probiotic |
| GCF_025191285.1 | Lactobacillus helveticus | Russia | Europe | Lactobacillaceae | Probiotic |
| GCF_025191325.1 | Lactobacillus helveticus | France | Europe | Lactobacillaceae | Probiotic |
| GCF_025191365.1 | Lactobacillus helveticus | Switzerland | Europe | Lactobacillaceae | Probiotic |
| GCF_025122455.1 | Lactobacillus helveticus | Finland | Europe | Lactobacillaceae | Probiotic |
| GCF_025191425.1 | Lactobacillus helveticus | France | Europe | Lactobacillaceae | Probiotic |
| GCF_025191185.1 | Lactobacillus helveticus | Switzerland | Europe | Lactobacillaceae | Probiotic |
| GCF_025191155.1 | Lactobacillus helveticus | Italy | Europe | Lactobacillaceae | Probiotic |
| GCF_025191405.1 | Lactobacillus helveticus | Italy | Europe | Lactobacillaceae | Probiotic |
| GCF_025191485.1 | Lactobacillus helveticus | Finland | Europe | Lactobacillaceae | Probiotic |
| GCF_025191435.1 | Lactobacillus helveticus | Italy | Europe | Lactobacillaceae | Probiotic |
| GCF_025191465.1 | Lactobacillus helveticus | Finland | Europe | Lactobacillaceae | Probiotic |
| GCF_025191205.1 | Lactobacillus helveticus | France | Europe | Lactobacillaceae | Probiotic |
| GCF_025191215.1 | Lactobacillus helveticus | France | Europe | Lactobacillaceae | Probiotic |
| GCF_025191305.1 | Lactobacillus helveticus | Finland | Europe | Lactobacillaceae | Probiotic |
| GCF_025191255.1 | Lactobacillus helveticus | France | Europe | Lactobacillaceae | Probiotic |
| GCF_025191245.1 | Lactobacillus helveticus | Russia | Europe | Lactobacillaceae | Probiotic |
| GCF_025191145.1 | Lactobacillus helveticus | France | Europe | Lactobacillaceae | Probiotic |
| GCF_000493555.1 | Lactobacillus helveticus | France | Europe | Lactobacillaceae | Probiotic |
| GCF_000493335.1 | Lactobacillus helveticus | France | Europe | Lactobacillaceae | Probiotic |
| GCF_000493575.1 | Lactobacillus helveticus | France | Europe | Lactobacillaceae | Probiotic |
| GCF_001308285.1 | Lactobacillus helveticus | China | Asia | Lactobacillaceae | Probiotic |
| GCF_013249215.1 | Lactobacillus helveticus | Missing | Europe | Lactobacillaceae | Probiotic |
| GCF_023614485.1 | Lactobacillus helveticus | Belarus | Europe | Lactobacillaceae | Probiotic |
| GCF_002914985.1 | Lactobacillus helveticus | Italy | Europe | Lactobacillaceae | Probiotic |
| GCF_002868275.1 | Lactobacillus helveticus | France | Europe | Lactobacillaceae | Probiotic |
| GCF_019455935.1 | Lactobacillus helveticus | Switzerland | Europe | Lactobacillaceae | Probiotic |
| GCF_000765465.1 | Lactobacillus helveticus | Switzerland | Europe | Lactobacillaceae | Probiotic |
| GCF_001895375.1 | Lactobacillus helveticus | China | Asia | Lactobacillaceae | Probiotic |
| GCF_022833865.1 | Lactobacillus helveticus | Japan | Asia | Lactobacillaceae | Probiotic |
| GCF_017154195.1 | Lactobacillus helveticus | Nigeria | Africa | Lactobacillaceae | Probiotic |
| GCF_009362875.1 | Lactobacillus helveticus | Nigeria | Africa | Lactobacillaceae | Probiotic |
| GCF_025129275.1 | Lactobacillus helveticus | Kenya | Africa | Lactobacillaceae | Probiotic |
| GCF_025129315.1 | Lactobacillus helveticus | Kenya | Africa | Lactobacillaceae | Probiotic |
| GCF_025129335.1 | Lactobacillus helveticus | Kenya | Africa | Lactobacillaceae | Probiotic |
| GCF_900248185.1 | Lactobacillus helveticus | Finland | Europe | Lactobacillaceae | Probiotic |
| GCF_902386585.1 | Lactobacillus helveticus | Russia | Europe | Lactobacillaceae | Probiotic |
| GCF_029823695.1 | Lactobacillus helveticus | Italy | Europe | Lactobacillaceae | Probiotic |
| GCF_011029225.1 | Lactobacillus jensenii | Thailand | Asia | Lactobacillaceae | Probiotic |
| GCF_026184075.1 | Lactobacillus jensenii | USA | North America | Lactobacillaceae | Probiotic |
| GCF_008726325.1 | Lactobacillus jensenii | USA | North America | Lactobacillaceae | Probiotic |
| GCF_007785935.1 | Lactobacillus jensenii | USA | North America | Lactobacillaceae | Probiotic |
| GCF_021495305.1 | Lactobacillus jensenii | USA | North America | Lactobacillaceae | Probiotic |
| GCF_030215165.1 | Lactobacillus jensenii | USA | North America | Lactobacillaceae | Probiotic |
| GCF_030229985.1 | Lactobacillus jensenii | USA | North America | Lactobacillaceae | Probiotic |
| GCF_030215235.1 | Lactobacillus jensenii | USA | North America | Lactobacillaceae | Probiotic |
| GCF_012030285.1 | Lactobacillus jensenii | USA | North America | Lactobacillaceae | Probiotic |
| GCF_033804025.1 | Lactobacillus jensenii | USA | North America | Lactobacillaceae | Probiotic |
| GCF_033804045.1 | Lactobacillus jensenii | USA | North America | Lactobacillaceae | Probiotic |
| GCF_021494985.1 | Lactobacillus jensenii | USA | North America | Lactobacillaceae | Probiotic |
| GCF_021495105.1 | Lactobacillus jensenii | USA | North America | Lactobacillaceae | Probiotic |
| GCF_033804065.1 | Lactobacillus jensenii | USA | North America | Lactobacillaceae | Probiotic |
| GCF_033804105.1 | Lactobacillus jensenii | USA | North America | Lactobacillaceae | Probiotic |
| GCF_032376755.1 | Lactobacillus jensenii | USA | North America | Lactobacillaceae | Probiotic |
| GCF_008727025.1 | Lactobacillus jensenii | USA | North America | Lactobacillaceae | Probiotic |
| GCF_033803585.1 | Lactobacillus jensenii | USA | North America | Lactobacillaceae | Probiotic |
| GCF_008726555.1 | Lactobacillus jensenii | USA | North America | Lactobacillaceae | Probiotic |
| GCF_033804085.1 | Lactobacillus jensenii | USA | North America | Lactobacillaceae | Probiotic |
| GCF_033804125.1 | Lactobacillus jensenii | USA | North America | Lactobacillaceae | Probiotic |
| GCF_033804145.1 | Lactobacillus jensenii | USA | North America | Lactobacillaceae | Probiotic |
| GCF_008726585.1 | Lactobacillus jensenii | USA | North America | Lactobacillaceae | Probiotic |
| GCF_032377035.1 | Lactobacillus jensenii | USA | North America | Lactobacillaceae | Probiotic |
| GCF_030217465.1 | Lactobacillus jensenii | USA | North America | Lactobacillaceae | Probiotic |
| GCF_007786135.1 | Lactobacillus jensenii | USA | North America | Lactobacillaceae | Probiotic |
| GCF_007786155.1 | Lactobacillus jensenii | USA | North America | Lactobacillaceae | Probiotic |
| GCF_026183995.1 | Lactobacillus jensenii | USA | North America | Lactobacillaceae | Probiotic |
| GCF_030226935.1 | Lactobacillus jensenii | USA | North America | Lactobacillaceae | Probiotic |
| GCF_026184135.1 | Lactobacillus jensenii | USA | North America | Lactobacillaceae | Probiotic |
| GCF_030218205.1 | Lactobacillus jensenii | USA | North America | Lactobacillaceae | Probiotic |
| GCF_026184025.1 | Lactobacillus jensenii | USA | North America | Lactobacillaceae | Probiotic |
| GCF_030211725.1 | Lactobacillus jensenii | USA | North America | Lactobacillaceae | Probiotic |
| GCF_032377315.1 | Lactobacillus jensenii | USA | North America | Lactobacillaceae | Probiotic |
| GCF_012029675.1 | Lactobacillus jensenii | USA | North America | Lactobacillaceae | Probiotic |
| GCF_012029775.1 | Lactobacillus jensenii | USA | North America | Lactobacillaceae | Probiotic |
| GCF_030218485.1 | Lactobacillus jensenii | USA | North America | Lactobacillaceae | Probiotic |
| GCF_007785825.1 | Lactobacillus jensenii | USA | North America | Lactobacillaceae | Probiotic |
| GCF_002848045.1 | Lactobacillus jensenii | USA | North America | Lactobacillaceae | Probiotic |
| GCF_030218635.1 | Lactobacillus jensenii | USA | North America | Lactobacillaceae | Probiotic |
| GCF_007786035.1 | Lactobacillus jensenii | USA | North America | Lactobacillaceae | Probiotic |
| GCF_030218735.1 | Lactobacillus jensenii | USA | North America | Lactobacillaceae | Probiotic |
| GCF_007785915.1 | Lactobacillus jensenii | USA | North America | Lactobacillaceae | Probiotic |
| GCF_030218705.1 | Lactobacillus jensenii | USA | North America | Lactobacillaceae | Probiotic |
| GCF_007786085.1 | Lactobacillus jensenii | USA | North America | Lactobacillaceae | Probiotic |
| GCF_021495005.1 | Lactobacillus jensenii | USA | North America | Lactobacillaceae | Probiotic |
| GCF_030226565.1 | Lactobacillus jensenii | USA | North America | Lactobacillaceae | Probiotic |
| GCF_002863405.1 | Lactobacillus jensenii | USA | North America | Lactobacillaceae | Probiotic |
| GCF_019459545.1 | Lactobacillus jensenii | India | Asia | Lactobacillaceae | Probiotic |
| GCF_001936235.1 | Lactobacillus jensenii | South Korea | Asia | Lactobacillaceae | Probiotic |
| GCF_000162335.1 | Lactobacillus jensenii | USA | North America | Lactobacillaceae | Probiotic |
| GCF_022453965.1 | Lactobacillus jensenii | USA | North America | Lactobacillaceae | Probiotic |
| GCF_011714655.1 | Lactobacillus jensenii | USA | North America | Lactobacillaceae | Probiotic |
| GCF_011714625.1 | Lactobacillus jensenii | USA | North America | Lactobacillaceae | Probiotic |
| GCF_022455835.1 | Lactobacillus jensenii | South Africa | Africa | Lactobacillaceae | Probiotic |
| GCF_001012685.1 | Lactobacillus jensenii | Missing | Missing | Lactobacillaceae | Probiotic |
| GCF_001012665.1 | Lactobacillus jensenii | Missing | Missing | Lactobacillaceae | Probiotic |
| GCF_022456225.1 | Lactobacillus jensenii | South Africa | Africa | Lactobacillaceae | Probiotic |
| GCF_022454195.1 | Lactobacillus jensenii | USA | North America | Lactobacillaceae | Probiotic |
| GCF_022454235.1 | Lactobacillus jensenii | USA | North America | Lactobacillaceae | Probiotic |
| GCF_022454245.1 | Lactobacillus jensenii | USA | North America | Lactobacillaceae | Probiotic |
| GCF_022454595.1 | Lactobacillus jensenii | USA | North America | Lactobacillaceae | Probiotic |
| GCF_022456915.1 | Lactobacillus jensenii | USA | North America | Lactobacillaceae | Probiotic |
| GCF_022454995.1 | Lactobacillus jensenii | USA | North America | Lactobacillaceae | Probiotic |
| GCF_027155585.1 | Lactobacillus jensenii | USA | North America | Lactobacillaceae | Probiotic |
| GCF_027155725.1 | Lactobacillus jensenii | USA | North America | Lactobacillaceae | Probiotic |
| GCF_027155905.1 | Lactobacillus jensenii | USA | North America | Lactobacillaceae | Probiotic |
| GCF_027155785.1 | Lactobacillus jensenii | USA | North America | Lactobacillaceae | Probiotic |
| GCF_027155805.1 | Lactobacillus jensenii | USA | North America | Lactobacillaceae | Probiotic |
| GCF_027160725.1 | Lactobacillus jensenii | USA | North America | Lactobacillaceae | Probiotic |
| GCF_027160745.1 | Lactobacillus jensenii | USA | North America | Lactobacillaceae | Probiotic |
| GCF_027583935.1 | Lactobacillus jensenii | USA | North America | Lactobacillaceae | Probiotic |
| GCF_027584115.1 | Lactobacillus jensenii | USA | North America | Lactobacillaceae | Probiotic |
| GCF_027584135.1 | Lactobacillus jensenii | USA | North America | Lactobacillaceae | Probiotic |
| GCF_027584175.1 | Lactobacillus jensenii | USA | North America | Lactobacillaceae | Probiotic |
| GCF_027155795.1 | Lactobacillus jensenii | USA | North America | Lactobacillaceae | Probiotic |
| GCF_027155885.1 | Lactobacillus jensenii | USA | North America | Lactobacillaceae | Probiotic |
| GCF_027155915.1 | Lactobacillus jensenii | USA | North America | Lactobacillaceae | Probiotic |
| GCF_027155925.1 | Lactobacillus jensenii | USA | North America | Lactobacillaceae | Probiotic |
| GCF_027155965.1 | Lactobacillus jensenii | USA | North America | Lactobacillaceae | Probiotic |
| GCF_000175035.1 | Lactobacillus jensenii | Missing | Missing | Lactobacillaceae | Probiotic |
| GCF_022456535.1 | Lactobacillus jensenii | South Africa | Africa | Lactobacillaceae | Probiotic |
| GCF_022456555.1 | Lactobacillus jensenii | South Africa | Africa | Lactobacillaceae | Probiotic |
| GCF_022456585.1 | Lactobacillus jensenii | South Africa | Africa | Lactobacillaceae | Probiotic |
| GCF_022456675.1 | Lactobacillus jensenii | South Africa | Africa | Lactobacillaceae | Probiotic |
| GCF_951863445.1 | Lactobacillus jensenii | Ireland | Europe | Lactobacillaceae | Probiotic |
| GCF_951863455.1 | Lactobacillus jensenii | Ireland | Europe | Lactobacillaceae | Probiotic |
| GCF_951856975.1 | Lactobacillus jensenii | Ireland | Europe | Lactobacillaceae | Probiotic |
| GCF_951863465.1 | Lactobacillus jensenii | Ireland | Europe | Lactobacillaceae | Probiotic |
| GCF_951863495.1 | Lactobacillus jensenii | Ireland | Europe | Lactobacillaceae | Probiotic |
| GCF_951856945.1 | Lactobacillus jensenii | Ireland | Europe | Lactobacillaceae | Probiotic |
| GCF_951856875.1 | Lactobacillus jensenii | Ireland | Europe | Lactobacillaceae | Probiotic |
| GCF_951857035.1 | Lactobacillus jensenii | Ireland | Europe | Lactobacillaceae | Probiotic |
| GCF_951857025.1 | Lactobacillus jensenii | Ireland | Europe | Lactobacillaceae | Probiotic |
| GCF_951863505.1 | Lactobacillus jensenii | Ireland | Europe | Lactobacillaceae | Probiotic |
| GCF_951863425.1 | Lactobacillus jensenii | Ireland | Europe | Lactobacillaceae | Probiotic |
| GCF_018094625.1 | Lactobacillus jensenii | Missing | Missing | Lactobacillaceae | Probiotic |
| GCF_004011315.1 | Lactobacillus johnsonii | China | Asia | Lactobacillaceae | Probiotic |
| GCF_012843525.1 | Lactobacillus johnsonii | Germany | Europe | Lactobacillaceae | Probiotic |
| GCF_001572665.1 | Lactobacillus johnsonii | China | Asia | Lactobacillaceae | Probiotic |
| GCF_002270045.1 | Lactobacillus johnsonii | USA | North America | Lactobacillaceae | Probiotic |
| GCF_002553695.1 | Lactobacillus johnsonii | USA | North America | Lactobacillaceae | Probiotic |
| GCF_002269975.1 | Lactobacillus johnsonii | USA | North America | Lactobacillaceae | Probiotic |
| GCF_002176835.1 | Lactobacillus johnsonii | USA | North America | Lactobacillaceae | Probiotic |
| GCF_002176855.1 | Lactobacillus johnsonii | USA | North America | Lactobacillaceae | Probiotic |
| GCF_002269965.1 | Lactobacillus johnsonii | USA | North America | Lactobacillaceae | Probiotic |
| GCF_002553705.1 | Lactobacillus johnsonii | USA | North America | Lactobacillaceae | Probiotic |
| GCF_032376765.1 | Lactobacillus johnsonii | USA | North America | Lactobacillaceae | Probiotic |
| GCF_019459625.1 | Lactobacillus johnsonii | India | Asia | Lactobacillaceae | Probiotic |
| GCF_004684975.1 | Lactobacillus johnsonii | USA | North America | Lactobacillaceae | Probiotic |
| GCF_023483905.1 | Lactobacillus johnsonii | Russia | Europe | Lactobacillaceae | Probiotic |
| GCF_029662885.1 | Lactobacillus johnsonii | USA | North America | Lactobacillaceae | Probiotic |
| GCF_004793575.1 | Lactobacillus johnsonii | Canada | North America | Lactobacillaceae | Probiotic |
| GCF_014058685.1 | Lactobacillus johnsonii | USA | North America | Lactobacillaceae | Probiotic |
| GCF_000008065.1 | Lactobacillus johnsonii | Missing | Europe | Lactobacillaceae | Probiotic |
| GCF_032463545.1 | Lactobacillus johnsonii | China | Asia | Lactobacillaceae | Probiotic |
| GCF_000498675.1 | Lactobacillus johnsonii | Missing | North America | Lactobacillaceae | Probiotic |
| GCF_032463685.1 | Lactobacillus johnsonii | China | Asia | Lactobacillaceae | Probiotic |
| GCF_018917265.1 | Lactobacillus johnsonii | China | Asia | Lactobacillaceae | Probiotic |
| GCF_021464365.1 | Lactobacillus johnsonii | USA | North America | Lactobacillaceae | Probiotic |
| GCF_022213385.1 | Lactobacillus johnsonii | USA | North America | Lactobacillaceae | Probiotic |
| GCF_010206295.1 | Lactobacillus johnsonii | France | Europe | Lactobacillaceae | Probiotic |
| GCF_009708135.1 | Lactobacillus johnsonii | USA | North America | Lactobacillaceae | Probiotic |
| GCF_002156645.1 | Lactobacillus johnsonii | USA | North America | Lactobacillaceae | Probiotic |
| GCF_007876425.1 | Lactobacillus johnsonii | USA | North America | Lactobacillaceae | Probiotic |
| GCF_022643205.1 | Lactobacillus johnsonii | Canada | North America | Lactobacillaceae | Probiotic |
| GCF_033704075.1 | Lactobacillus johnsonii | Thailand | Asia | Lactobacillaceae | Probiotic |
| GCF_034808125.1 | Lactobacillus johnsonii | South Korea | Asia | Lactobacillaceae | Probiotic |
| GCF_003428395.1 | Lactobacillus johnsonii | South Korea | Asia | Lactobacillaceae | Probiotic |
| GCF_014841035.1 | Lactobacillus johnsonii | China | Asia | Lactobacillaceae | Probiotic |
| GCF_010586925.1 | Lactobacillus johnsonii | USA | North America | Lactobacillaceae | Probiotic |
| GCF_000091405.1 | Lactobacillus johnsonii | United Kingdom | Europe | Lactobacillaceae | Probiotic |
| GCF_024622485.1 | Lactobacillus johnsonii | Germany | Europe | Lactobacillaceae | Probiotic |
| GCF_000204985.1 | Lactobacillus johnsonii | Ireland | Europe | Lactobacillaceae | Probiotic |
| GCF_009769185.1 | Lactobacillus johnsonii | United Kingdom | Europe | Lactobacillaceae | Probiotic |
| GCF_021442345.1 | Lactobacillus johnsonii | France | Europe | Lactobacillaceae | Probiotic |
| GCF_025191025.1 | Lactobacillus johnsonii | France | Europe | Lactobacillaceae | Probiotic |
| GCF_025190845.1 | Lactobacillus johnsonii | Germany | Europe | Lactobacillaceae | Probiotic |
| GCF_025191045.1 | Lactobacillus johnsonii | France | Europe | Lactobacillaceae | Probiotic |
| GCF_025190945.1 | Lactobacillus johnsonii | Japan | Asia | Lactobacillaceae | Probiotic |
| GCF_025190925.1 | Lactobacillus johnsonii | United Kingdom | Europe | Lactobacillaceae | Probiotic |
| GCF_025191055.1 | Lactobacillus johnsonii | USA | North America | Lactobacillaceae | Probiotic |
| GCF_025190905.1 | Lactobacillus johnsonii | France | Europe | Lactobacillaceae | Probiotic |
| GCF_025190885.1 | Lactobacillus johnsonii | France | Europe | Lactobacillaceae | Probiotic |
| GCF_025190965.1 | Lactobacillus johnsonii | USA | North America | Lactobacillaceae | Probiotic |
| GCF_025190985.1 | Lactobacillus johnsonii | USA | North America | Lactobacillaceae | Probiotic |
| GCF_025191005.1 | Lactobacillus johnsonii | USA | North America | Lactobacillaceae | Probiotic |
| GCF_004010015.1 | Lactobacillus johnsonii | South Korea | Asia | Lactobacillaceae | Probiotic |
| GCF_004569425.1 | Lactobacillus johnsonii | United Kingdom | Europe | Lactobacillaceae | Probiotic |
| GCF_030237765.1 | Lactobacillus johnsonii | China | Asia | Lactobacillaceae | Probiotic |
| GCF_003316915.1 | Lactobacillus johnsonii | South Korea | Asia | Lactobacillaceae | Probiotic |
| GCF_015560715.1 | Lactobacillus johnsonii | USA | North America | Lactobacillaceae | Probiotic |
| GCF_008868555.1 | Lactobacillus johnsonii | Missing | Missing | Lactobacillaceae | Probiotic |
| GCF_002160105.1 | Lactobacillus johnsonii | Czech Republic | Europe | Lactobacillaceae | Probiotic |
| GCF_027666205.1 | Lactobacillus johnsonii | China | Asia | Lactobacillaceae | Probiotic |
| GCF_027665575.1 | Lactobacillus johnsonii | China | Asia | Lactobacillaceae | Probiotic |
| GCF_027682595.1 | Lactobacillus johnsonii | China | Asia | Lactobacillaceae | Probiotic |
| GCF_020026535.1 | Lactobacillus johnsonii | USA | North America | Lactobacillaceae | Probiotic |
| GCF_020026475.1 | Lactobacillus johnsonii | USA | North America | Lactobacillaceae | Probiotic |
| GCF_022810665.1 | Lactobacillus johnsonii | South Korea | Asia | Lactobacillaceae | Probiotic |
| GCF_013487865.1 | Lactobacillus johnsonii | USA | North America | Lactobacillaceae | Probiotic |
| GCF_015235065.1 | Lactobacillus johnsonii | United Kingdom | Europe | Lactobacillaceae | Probiotic |
| GCF_001270785.1 | Lactobacillus johnsonii | Israel | Asia | Lactobacillaceae | Probiotic |
| GCF_002253185.1 | Lactobacillus johnsonii | United Kingdom | Europe | Lactobacillaceae | Probiotic |
| GCF_002253165.1 | Lactobacillus johnsonii | United Kingdom | Europe | Lactobacillaceae | Probiotic |
| GCF_002253205.1 | Lactobacillus johnsonii | United Kingdom | Europe | Lactobacillaceae | Probiotic |
| GCF_002253245.1 | Lactobacillus johnsonii | United Kingdom | Europe | Lactobacillaceae | Probiotic |
| GCF_002253275.1 | Lactobacillus johnsonii | United Kingdom | Europe | Lactobacillaceae | Probiotic |
| GCF_002253285.1 | Lactobacillus johnsonii | United Kingdom | Europe | Lactobacillaceae | Probiotic |
| GCF_011959745.1 | Lactobacillus johnsonii | Japan | Asia | Lactobacillaceae | Probiotic |
| GCF_902362355.1 | Lactobacillus johnsonii | United Kingdom | Europe | Lactobacillaceae | Probiotic |
| GCF_910576225.1 | Lactobacillus johnsonii | United Kingdom | Europe | Lactobacillaceae | Probiotic |
| GCF_910574655.1 | Lactobacillus johnsonii | United Kingdom | Europe | Lactobacillaceae | Probiotic |
| GCF_910574385.1 | Lactobacillus johnsonii | United Kingdom | Europe | Lactobacillaceae | Probiotic |
| GCF_910574395.1 | Lactobacillus johnsonii | United Kingdom | Europe | Lactobacillaceae | Probiotic |
| GCF_000159355.1 | Lactobacillus johnsonii | Belgium | Europe | Lactobacillaceae | Probiotic |
| GCF_003184305.1 | Levilactobacillus brevis | China | Asia | Lactobacillaceae | Probiotic |
| GCF_002532185.1 | Levilactobacillus brevis | Croatia | Europe | Lactobacillaceae | Probiotic |
| GCF_022350025.1 | Levilactobacillus brevis | China | Asia | Lactobacillaceae | Probiotic |
| GCF_000784455.1 | Levilactobacillus brevis | South Korea | Asia | Lactobacillaceae | Probiotic |
| GCF_003345725.1 | Levilactobacillus brevis | South Korea | Asia | Lactobacillaceae | Probiotic |
| GCF_006228285.1 | Levilactobacillus brevis | Netherlands | Europe | Lactobacillaceae | Probiotic |
| GCF_006228205.1 | Levilactobacillus brevis | Singapore | Asia | Lactobacillaceae | Probiotic |
| GCF_006228265.1 | Levilactobacillus brevis | Netherlands | Europe | Lactobacillaceae | Probiotic |
| GCF_006228245.1 | Levilactobacillus brevis | Netherlands | Europe | Lactobacillaceae | Probiotic |
| GCF_006228305.1 | Levilactobacillus brevis | Netherlands | Europe | Lactobacillaceae | Probiotic |
| GCF_003289085.1 | Levilactobacillus brevis | Chile | South America | Lactobacillaceae | Probiotic |
| GCF_006381875.1 | Levilactobacillus brevis | Ireland | Europe | Lactobacillaceae | Probiotic |
| GCF_006382065.1 | Levilactobacillus brevis | Ireland | Europe | Lactobacillaceae | Probiotic |
| GCF_006381935.1 | Levilactobacillus brevis | Ireland | Europe | Lactobacillaceae | Probiotic |
| GCF_030361325.1 | Levilactobacillus brevis | Russia | Europe | Lactobacillaceae | Probiotic |
| GCF_030361165.1 | Levilactobacillus brevis | Russia | Europe | Lactobacillaceae | Probiotic |
| GCF_000833415.1 | Levilactobacillus brevis | Missing | Europe | Lactobacillaceae | Probiotic |
| GCF_000833395.1 | Levilactobacillus brevis | Missing | Missing | Lactobacillaceae | Probiotic |
| GCF_002117325.1 | Levilactobacillus brevis | Germany | Europe | Lactobacillaceae | Probiotic |
| GCF_002117345.1 | Levilactobacillus brevis | Germany | Europe | Lactobacillaceae | Probiotic |
| GCF_030378305.1 | Levilactobacillus brevis | South Korea | Asia | Lactobacillaceae | Probiotic |
| GCF_025447255.1 | Levilactobacillus brevis | South Korea | Asia | Lactobacillaceae | Probiotic |
| GCF_002173555.1 | Levilactobacillus brevis | South Korea | Asia | Lactobacillaceae | Probiotic |
| GCF_002174235.1 | Levilactobacillus brevis | South Korea | Asia | Lactobacillaceae | Probiotic |
| GCF_004055405.1 | Levilactobacillus brevis | South Korea | Asia | Lactobacillaceae | Probiotic |
| GCF_018798865.1 | Levilactobacillus brevis | South Korea | Asia | Lactobacillaceae | Probiotic |
| GCF_026626025.1 | Levilactobacillus brevis | South Korea | Asia | Lactobacillaceae | Probiotic |
| GCF_002532245.1 | Levilactobacillus brevis | Croatia | Europe | Lactobacillaceae | Probiotic |
| GCF_002532155.1 | Levilactobacillus brevis | Croatia | Europe | Lactobacillaceae | Probiotic |
| GCF_026639055.1 | Levilactobacillus brevis | China | Asia | Lactobacillaceae | Probiotic |
| GCF_006228225.1 | Levilactobacillus brevis | Ireland | Europe | Lactobacillaceae | Probiotic |
| GCF_024800685.1 | Levilactobacillus brevis | South Korea | Asia | Lactobacillaceae | Probiotic |
| GCF_027692245.1 | Levilactobacillus brevis | China | Asia | Lactobacillaceae | Probiotic |
| GCF_014905055.1 | Levilactobacillus brevis | South Korea | Asia | Lactobacillaceae | Probiotic |
| GCF_001676805.1 | Levilactobacillus brevis | Hong Kong | Asia | Lactobacillaceae | Probiotic |
| GCF_900452615.1 | Levilactobacillus brevis | Missing | Europe | Lactobacillaceae | Probiotic |
| GCF_006538845.1 | Levilactobacillus brevis | Missing | Missing | Lactobacillaceae | Probiotic |
| GCF_002933755.1 | Levilactobacillus brevis | Missing | Missing | Lactobacillaceae | Probiotic |
| GCF_006539265.1 | Levilactobacillus brevis | Missing | Missing | Lactobacillaceae | Probiotic |
| GCF_006538905.1 | Levilactobacillus brevis | Missing | Missing | Lactobacillaceae | Probiotic |
| GCF_018916925.1 | Levilactobacillus brevis | China | Asia | Lactobacillaceae | Probiotic |
| GCF_032841255.1 | Levilactobacillus brevis | Greece | Europe | Lactobacillaceae | Probiotic |
| GCF_032841235.1 | Levilactobacillus brevis | Greece | Europe | Lactobacillaceae | Probiotic |
| GCF_018784425.1 | Levilactobacillus brevis | Ireland | Europe | Lactobacillaceae | Probiotic |
| GCF_026930365.1 | Levilactobacillus brevis | Croatia | Europe | Lactobacillaceae | Probiotic |
| GCF_026930445.1 | Levilactobacillus brevis | Croatia | Europe | Lactobacillaceae | Probiotic |
| GCF_027108875.1 | Levilactobacillus brevis | Croatia | Europe | Lactobacillaceae | Probiotic |
| GCF_026930425.1 | Levilactobacillus brevis | Croatia | Europe | Lactobacillaceae | Probiotic |
| GCF_026410225.1 | Levilactobacillus brevis | China | Asia | Lactobacillaceae | Probiotic |
| GCF_027591835.1 | Levilactobacillus brevis | Thailand | Asia | Lactobacillaceae | Probiotic |
| GCF_003813165.1 | Levilactobacillus brevis | South Korea | Asia | Lactobacillaceae | Probiotic |
| GCF_037201785.1 | Levilactobacillus brevis | China | Asia | Lactobacillaceae | Probiotic |
| GCF_020509325.1 | Levilactobacillus brevis | India | Asia | Lactobacillaceae | Probiotic |
| GCF_019693335.1 | Levilactobacillus brevis | USA | North America | Lactobacillaceae | Probiotic |
| GCF_018588725.1 | Levilactobacillus brevis | Mexico | North America | Lactobacillaceae | Probiotic |
| GCF_025122425.1 | Levilactobacillus brevis | France | Europe | Lactobacillaceae | Probiotic |
| GCF_001540905.1 | Levilactobacillus brevis | France | Europe | Lactobacillaceae | Probiotic |
| GCF_009741845.1 | Levilactobacillus brevis | Missing | Missing | Lactobacillaceae | Probiotic |
| GCF_003346245.1 | Levilactobacillus brevis | Slovakia | Europe | Lactobacillaceae | Probiotic |
| GCF_003346095.1 | Levilactobacillus brevis | Slovakia | Europe | Lactobacillaceae | Probiotic |
| GCF_000359625.1 | Levilactobacillus brevis | Japan | Asia | Lactobacillaceae | Probiotic |
| GCF_015238595.1 | Levilactobacillus brevis | China | Asia | Lactobacillaceae | Probiotic |
| GCF_026622905.1 | Levilactobacillus brevis | China | Asia | Lactobacillaceae | Probiotic |
| GCF_000474675.1 | Levilactobacillus brevis | South Korea | Asia | Lactobacillaceae | Probiotic |
| GCF_001953605.1 | Levilactobacillus brevis | Canada | North America | Lactobacillaceae | Probiotic |
| GCF_003053125.1 | Levilactobacillus brevis | USA | North America | Lactobacillaceae | Probiotic |
| GCF_023744095.1 | Levilactobacillus brevis | Greece | Europe | Lactobacillaceae | Probiotic |
| GCF_023743375.1 | Levilactobacillus brevis | Greece | Europe | Lactobacillaceae | Probiotic |
| GCF_023743355.1 | Levilactobacillus brevis | Greece | Europe | Lactobacillaceae | Probiotic |
| GCF_023743515.1 | Levilactobacillus brevis | Greece | Europe | Lactobacillaceae | Probiotic |
| GCF_023743475.1 | Levilactobacillus brevis | Greece | Europe | Lactobacillaceae | Probiotic |
| GCF_023744005.1 | Levilactobacillus brevis | Greece | Europe | Lactobacillaceae | Probiotic |
| GCF_023744425.1 | Levilactobacillus brevis | Greece | Europe | Lactobacillaceae | Probiotic |
| GCF_023744035.1 | Levilactobacillus brevis | Greece | Europe | Lactobacillaceae | Probiotic |
| GCF_023744105.1 | Levilactobacillus brevis | Greece | Europe | Lactobacillaceae | Probiotic |
| GCF_023744455.1 | Levilactobacillus brevis | Greece | Europe | Lactobacillaceae | Probiotic |
| GCF_023743495.1 | Levilactobacillus brevis | Greece | Europe | Lactobacillaceae | Probiotic |
| GCF_001722065.1 | Levilactobacillus brevis | Ireland | Europe | Lactobacillaceae | Probiotic |
| GCF_000814725.1 | Levilactobacillus brevis | USA | North America | Lactobacillaceae | Probiotic |
| GCF_003060705.1 | Levilactobacillus brevis | China | Asia | Lactobacillaceae | Probiotic |
| GCF_018257245.1 | Levilactobacillus brevis | USA | North America | Lactobacillaceae | Probiotic |
| GCF_018257185.1 | Levilactobacillus brevis | USA | North America | Lactobacillaceae | Probiotic |
| GCF_018257295.1 | Levilactobacillus brevis | USA | North America | Lactobacillaceae | Probiotic |
| GCF_018257275.1 | Levilactobacillus brevis | USA | North America | Lactobacillaceae | Probiotic |
| GCF_018257235.1 | Levilactobacillus brevis | USA | North America | Lactobacillaceae | Probiotic |
| GCF_002318935.1 | Levilactobacillus brevis | South Korea | Asia | Lactobacillaceae | Probiotic |
| GCF_001541605.1 | Levilactobacillus brevis | Croatia | Europe | Lactobacillaceae | Probiotic |
| GCF_002093065.1 | Levilactobacillus brevis | Argentina | South America | Lactobacillaceae | Probiotic |
| GCF_025194485.1 | Levilactobacillus brevis | Poland | Europe | Lactobacillaceae | Probiotic |
| GCF_025194515.1 | Levilactobacillus brevis | France | Europe | Lactobacillaceae | Probiotic |
| GCF_025194505.1 | Levilactobacillus brevis | Italy | Europe | Lactobacillaceae | Probiotic |
| GCF_025194325.1 | Levilactobacillus brevis | France | Europe | Lactobacillaceae | Probiotic |
| GCF_025194285.1 | Levilactobacillus brevis | France | Europe | Lactobacillaceae | Probiotic |
| GCF_025194385.1 | Levilactobacillus brevis | Poland | Europe | Lactobacillaceae | Probiotic |
| GCF_025194405.1 | Levilactobacillus brevis | Poland | Europe | Lactobacillaceae | Probiotic |
| GCF_025194465.1 | Levilactobacillus brevis | Poland | Europe | Lactobacillaceae | Probiotic |
| GCF_025194545.1 | Levilactobacillus brevis | Italy | Europe | Lactobacillaceae | Probiotic |
| GCF_025194305.1 | Levilactobacillus brevis | France | Europe | Lactobacillaceae | Probiotic |
| GCF_025000005.1 | Levilactobacillus brevis | France | Europe | Lactobacillaceae | Probiotic |
| GCF_000807975.1 | Levilactobacillus brevis | United Kingdom | Europe | Lactobacillaceae | Probiotic |
| GCF_001923045.1 | Levilactobacillus brevis | United Kingdom | Europe | Lactobacillaceae | Probiotic |
| GCF_013249075.1 | Levilactobacillus brevis | Missing | Missing | Lactobacillaceae | Probiotic |
| GCF_002762175.1 | Levilactobacillus brevis | USA | North America | Lactobacillaceae | Probiotic |
| GCF_000014465.1 | Levilactobacillus brevis | Missing | Missing | Lactobacillaceae | Probiotic |
| GCF_020532035.1 | Levilactobacillus brevis | Thailand | Asia | Lactobacillaceae | Probiotic |
| GCF_027698185.1 | Levilactobacillus brevis | China | Asia | Lactobacillaceae | Probiotic |
| GCF_027698385.1 | Levilactobacillus brevis | China | Asia | Lactobacillaceae | Probiotic |
| GCF_027698465.1 | Levilactobacillus brevis | China | Asia | Lactobacillaceae | Probiotic |
| GCF_027698855.1 | Levilactobacillus brevis | China | Asia | Lactobacillaceae | Probiotic |
| GCF_027698985.1 | Levilactobacillus brevis | China | Asia | Lactobacillaceae | Probiotic |
| GCF_027699065.1 | Levilactobacillus brevis | China | Asia | Lactobacillaceae | Probiotic |
| GCF_000526755.1 | Levilactobacillus brevis | Missing | Missing | Lactobacillaceae | Probiotic |
| GCF_027664805.1 | Levilactobacillus brevis | China | Asia | Lactobacillaceae | Probiotic |
| GCF_027686645.1 | Levilactobacillus brevis | China | Asia | Lactobacillaceae | Probiotic |
| GCF_025186345.1 | Levilactobacillus brevis | Switzerland | Europe | Lactobacillaceae | Probiotic |
| GCF_021384515.1 | Levilactobacillus brevis | USA | North America | Lactobacillaceae | Probiotic |
| GCF_021384565.1 | Levilactobacillus brevis | USA | North America | Lactobacillaceae | Probiotic |
| GCF_021384555.1 | Levilactobacillus brevis | USA | North America | Lactobacillaceae | Probiotic |
| GCF_021384505.1 | Levilactobacillus brevis | USA | North America | Lactobacillaceae | Probiotic |
| GCF_021384405.1 | Levilactobacillus brevis | USA | North America | Lactobacillaceae | Probiotic |
| GCF_021384445.1 | Levilactobacillus brevis | USA | North America | Lactobacillaceae | Probiotic |
| GCF_021384495.1 | Levilactobacillus brevis | USA | North America | Lactobacillaceae | Probiotic |
| GCF_021384395.1 | Levilactobacillus brevis | USA | North America | Lactobacillaceae | Probiotic |
| GCF_001010995.2 | Levilactobacillus brevis | Russia | Europe | Lactobacillaceae | Probiotic |
| GCF_002179515.1 | Levilactobacillus brevis | China | Asia | Lactobacillaceae | Probiotic |
| GCF_021384595.1 | Levilactobacillus brevis | USA | North America | Lactobacillaceae | Probiotic |
| GCF_021384615.1 | Levilactobacillus brevis | USA | North America | Lactobacillaceae | Probiotic |
| GCF_021384415.1 | Levilactobacillus brevis | USA | North America | Lactobacillaceae | Probiotic |
| GCF_018991675.1 | Levilactobacillus brevis | USA | North America | Lactobacillaceae | Probiotic |
| GCF_000875905.1 | Levilactobacillus brevis | Russia | Europe | Lactobacillaceae | Probiotic |
| GCF_018993685.1 | Levilactobacillus brevis | USA | North America | Lactobacillaceae | Probiotic |
| GCF_018991505.1 | Levilactobacillus brevis | USA | North America | Lactobacillaceae | Probiotic |
| GCF_002138395.1 | Levilactobacillus brevis | South Korea | Asia | Lactobacillaceae | Probiotic |
| GCF_925285815.1 | Levilactobacillus brevis | Slovenia | Europe | Lactobacillaceae | Probiotic |
| GCF_925285845.1 | Levilactobacillus brevis | Slovenia | Europe | Lactobacillaceae | Probiotic |
| GCF_925281805.1 | Levilactobacillus brevis | Missing | Missing | Lactobacillaceae | Probiotic |
| GCF_925279105.1 | Levilactobacillus brevis | Missing | Missing | Lactobacillaceae | Probiotic |
| GCF_900116975.1 | Levilactobacillus brevis | Denmark | Europe | Lactobacillaceae | Probiotic |
| GCF_900116995.1 | Levilactobacillus brevis | Denmark | Europe | Lactobacillaceae | Probiotic |
| GCF_900117025.1 | Levilactobacillus brevis | Denmark | Europe | Lactobacillaceae | Probiotic |
| GCF_900117015.1 | Levilactobacillus brevis | Denmark | Europe | Lactobacillaceae | Probiotic |
| GCF_900117045.1 | Levilactobacillus brevis | Denmark | Europe | Lactobacillaceae | Probiotic |
| GCF_900116945.1 | Levilactobacillus brevis | Czech Republic | Europe | Lactobacillaceae | Probiotic |
| GCF_902386565.1 | Levilactobacillus brevis | Russia | Europe | Lactobacillaceae | Probiotic |
| GCF_000469365.1 | Levilactobacillus brevis | Missing | Missing | Lactobacillaceae | Probiotic |
| GCF_030169145.1 | Ligilactobacillus salivarius | China | Asia | Lactobacillaceae | Probiotic |
| GCF_002162055.1 | Ligilactobacillus salivarius | China | Asia | Lactobacillaceae | Probiotic |
| GCF_014841055.1 | Ligilactobacillus salivarius | China | Asia | Lactobacillaceae | Probiotic |
| GCF_030518315.1 | Ligilactobacillus salivarius | China | Asia | Lactobacillaceae | Probiotic |
| GCF_012843615.1 | Ligilactobacillus salivarius | Germany | Europe | Lactobacillaceae | Probiotic |
| GCF_029872075.1 | Ligilactobacillus salivarius | Russia | Europe | Lactobacillaceae | Probiotic |
| GCF_024637975.1 | Ligilactobacillus salivarius | China | Asia | Lactobacillaceae | Probiotic |
| GCF_011029235.1 | Ligilactobacillus salivarius | Thailand | Asia | Lactobacillaceae | Probiotic |
| GCF_000008925.1 | Ligilactobacillus salivarius | Ireland | Europe | Lactobacillaceae | Probiotic |
| GCF_015159735.1 | Ligilactobacillus salivarius | India | Asia | Lactobacillaceae | Probiotic |
| GCF_016742795.1 | Ligilactobacillus salivarius | Canada | North America | Lactobacillaceae | Probiotic |
| GCF_024665615.1 | Ligilactobacillus salivarius | Missing | Missing | Lactobacillaceae | Probiotic |
| GCF_030434115.1 | Ligilactobacillus salivarius | South Korea | Asia | Lactobacillaceae | Probiotic |
| GCF_028656255.1 | Ligilactobacillus salivarius | South Korea | Asia | Lactobacillaceae | Probiotic |
| GCF_030434015.1 | Ligilactobacillus salivarius | South Korea | Asia | Lactobacillaceae | Probiotic |
| GCF_024397675.1 | Ligilactobacillus salivarius | China | Asia | Lactobacillaceae | Probiotic |
| GCF_000260335.1 | Ligilactobacillus salivarius | France | Europe | Lactobacillaceae | Probiotic |
| GCF_002738245.1 | Ligilactobacillus salivarius | Italy | Europe | Lactobacillaceae | Probiotic |
| GCF_029350895.1 | Ligilactobacillus salivarius | USA | North America | Lactobacillaceae | Probiotic |
| GCF_030062805.1 | Ligilactobacillus salivarius | China | Asia | Lactobacillaceae | Probiotic |
| GCF_030062935.1 | Ligilactobacillus salivarius | China | Asia | Lactobacillaceae | Probiotic |
| GCF_030062785.1 | Ligilactobacillus salivarius | China | Asia | Lactobacillaceae | Probiotic |
| GCF_030062915.1 | Ligilactobacillus salivarius | China | Asia | Lactobacillaceae | Probiotic |
| GCF_030062895.1 | Ligilactobacillus salivarius | China | Asia | Lactobacillaceae | Probiotic |
| GCF_030062875.1 | Ligilactobacillus salivarius | China | Asia | Lactobacillaceae | Probiotic |
| GCF_030062845.1 | Ligilactobacillus salivarius | China | Asia | Lactobacillaceae | Probiotic |
| GCF_030062825.1 | Ligilactobacillus salivarius | China | Asia | Lactobacillaceae | Probiotic |
| GCF_031557155.1 | Ligilactobacillus salivarius | China | Asia | Lactobacillaceae | Probiotic |
| GCF_023573545.1 | Ligilactobacillus salivarius | China | Asia | Lactobacillaceae | Probiotic |
| GCF_001011095.1 | Ligilactobacillus salivarius | China | Asia | Lactobacillaceae | Probiotic |
| GCF_029743055.1 | Ligilactobacillus salivarius | Spain | Europe | Lactobacillaceae | Probiotic |
| GCF_014336755.1 | Ligilactobacillus salivarius | USA | North America | Lactobacillaceae | Probiotic |
| GCF_027691665.1 | Ligilactobacillus salivarius | China | Asia | Lactobacillaceae | Probiotic |
| GCF_003438595.1 | Ligilactobacillus salivarius | China | Asia | Lactobacillaceae | Probiotic |
| GCF_027692205.1 | Ligilactobacillus salivarius | China | Asia | Lactobacillaceae | Probiotic |
| GCF_027692505.1 | Ligilactobacillus salivarius | China | Asia | Lactobacillaceae | Probiotic |
| GCF_009865705.1 | Ligilactobacillus salivarius | China | Asia | Lactobacillaceae | Probiotic |
| GCF_009870175.1 | Ligilactobacillus salivarius | China | Asia | Lactobacillaceae | Probiotic |
| GCF_009865735.1 | Ligilactobacillus salivarius | China | Asia | Lactobacillaceae | Probiotic |
| GCF_009865755.1 | Ligilactobacillus salivarius | China | Asia | Lactobacillaceae | Probiotic |
| GCF_009865785.1 | Ligilactobacillus salivarius | China | Asia | Lactobacillaceae | Probiotic |
| GCF_000215465.1 | Ligilactobacillus salivarius | South Korea | Asia | Lactobacillaceae | Probiotic |
| GCF_002079335.1 | Ligilactobacillus salivarius | Italy | Europe | Lactobacillaceae | Probiotic |
| GCF_002079925.1 | Ligilactobacillus salivarius | Missing | Missing | Lactobacillaceae | Probiotic |
| GCF_002079365.1 | Ligilactobacillus salivarius | United Kingdom | Europe | Lactobacillaceae | Probiotic |
| GCF_002079325.1 | Ligilactobacillus salivarius | Missing | Missing | Lactobacillaceae | Probiotic |
| GCF_002079795.1 | Ligilactobacillus salivarius | Missing | Missing | Lactobacillaceae | Probiotic |
| GCF_002079385.1 | Ligilactobacillus salivarius | Missing | Missing | Lactobacillaceae | Probiotic |
| GCF_027695365.1 | Ligilactobacillus salivarius | China | Asia | Lactobacillaceae | Probiotic |
| GCF_027695745.1 | Ligilactobacillus salivarius | China | Asia | Lactobacillaceae | Probiotic |
| GCF_002250405.1 | Ligilactobacillus salivarius | USA | North America | Lactobacillaceae | Probiotic |
| GCF_002079435.1 | Ligilactobacillus salivarius | Missing | Missing | Lactobacillaceae | Probiotic |
| GCF_024466835.1 | Ligilactobacillus salivarius | China | Asia | Lactobacillaceae | Probiotic |
| GCF_013391745.1 | Ligilactobacillus salivarius | Kazakhstan | Asia | Lactobacillaceae | Probiotic |
| GCF_002289925.1 | Ligilactobacillus salivarius | South Korea | Asia | Lactobacillaceae | Probiotic |
| GCF_002289905.1 | Ligilactobacillus salivarius | South Korea | Asia | Lactobacillaceae | Probiotic |
| GCF_002289875.1 | Ligilactobacillus salivarius | South Korea | Asia | Lactobacillaceae | Probiotic |
| GCF_002289865.1 | Ligilactobacillus salivarius | South Korea | Asia | Lactobacillaceae | Probiotic |
| GCF_002289845.1 | Ligilactobacillus salivarius | South Korea | Asia | Lactobacillaceae | Probiotic |
| GCF_002289795.1 | Ligilactobacillus salivarius | South Korea | Asia | Lactobacillaceae | Probiotic |
| GCF_002289785.1 | Ligilactobacillus salivarius | South Korea | Asia | Lactobacillaceae | Probiotic |
| GCF_002135095.1 | Ligilactobacillus salivarius | South Korea | Asia | Lactobacillaceae | Probiotic |
| GCF_002289745.1 | Ligilactobacillus salivarius | South Korea | Asia | Lactobacillaceae | Probiotic |
| GCF_002289725.1 | Ligilactobacillus salivarius | South Korea | Asia | Lactobacillaceae | Probiotic |
| GCF_002289735.1 | Ligilactobacillus salivarius | South Korea | Asia | Lactobacillaceae | Probiotic |
| GCF_002289685.1 | Ligilactobacillus salivarius | South Korea | Asia | Lactobacillaceae | Probiotic |
| GCF_002289665.1 | Ligilactobacillus salivarius | South Korea | Asia | Lactobacillaceae | Probiotic |
| GCF_002289675.1 | Ligilactobacillus salivarius | South Korea | Asia | Lactobacillaceae | Probiotic |
| GCF_002289965.1 | Ligilactobacillus salivarius | South Korea | Asia | Lactobacillaceae | Probiotic |
| GCF_002289625.1 | Ligilactobacillus salivarius | South Korea | Asia | Lactobacillaceae | Probiotic |
| GCF_002289605.1 | Ligilactobacillus salivarius | South Korea | Asia | Lactobacillaceae | Probiotic |
| GCF_002289985.1 | Ligilactobacillus salivarius | South Korea | Asia | Lactobacillaceae | Probiotic |
| GCF_009865815.1 | Ligilactobacillus salivarius | China | Asia | Lactobacillaceae | Probiotic |
| GCF_000758365.1 | Ligilactobacillus salivarius | Japan | Asia | Lactobacillaceae | Probiotic |
| GCF_002079405.1 | Ligilactobacillus salivarius | Missing | Missing | Lactobacillaceae | Probiotic |
| GCF_002079425.1 | Ligilactobacillus salivarius | Missing | Missing | Lactobacillaceae | Probiotic |
| GCF_002079465.1 | Ligilactobacillus salivarius | Missing | Missing | Lactobacillaceae | Probiotic |
| GCF_002079485.1 | Ligilactobacillus salivarius | Missing | Missing | Lactobacillaceae | Probiotic |
| GCF_002079505.1 | Ligilactobacillus salivarius | Missing | Missing | Lactobacillaceae | Probiotic |
| GCF_002079525.1 | Ligilactobacillus salivarius | Missing | Missing | Lactobacillaceae | Probiotic |
| GCF_011045395.1 | Ligilactobacillus salivarius | Poland | Europe | Lactobacillaceae | Probiotic |
| GCF_009865805.1 | Ligilactobacillus salivarius | China | Asia | Lactobacillaceae | Probiotic |
| GCF_009865795.1 | Ligilactobacillus salivarius | China | Asia | Lactobacillaceae | Probiotic |
| GCF_009869955.1 | Ligilactobacillus salivarius | China | Asia | Lactobacillaceae | Probiotic |
| GCF_002079545.1 | Ligilactobacillus salivarius | Missing | Missing | Lactobacillaceae | Probiotic |
| GCF_002079565.1 | Ligilactobacillus salivarius | Missing | Missing | Lactobacillaceae | Probiotic |
| GCF_000217735.1 | Ligilactobacillus salivarius | Missing | Missing | Lactobacillaceae | Probiotic |
| GCF_009865865.1 | Ligilactobacillus salivarius | China | Asia | Lactobacillaceae | Probiotic |
| GCF_009865885.1 | Ligilactobacillus salivarius | China | Asia | Lactobacillaceae | Probiotic |
| GCF_009865895.1 | Ligilactobacillus salivarius | China | Asia | Lactobacillaceae | Probiotic |
| GCF_009869985.1 | Ligilactobacillus salivarius | China | Asia | Lactobacillaceae | Probiotic |
| GCF_009865925.1 | Ligilactobacillus salivarius | China | Asia | Lactobacillaceae | Probiotic |
| GCF_009865965.1 | Ligilactobacillus salivarius | China | Asia | Lactobacillaceae | Probiotic |
| GCF_009865995.1 | Ligilactobacillus salivarius | China | Asia | Lactobacillaceae | Probiotic |
| GCF_009865985.1 | Ligilactobacillus salivarius | China | Asia | Lactobacillaceae | Probiotic |
| GCF_009866035.1 | Ligilactobacillus salivarius | China | Asia | Lactobacillaceae | Probiotic |
| GCF_009866025.1 | Ligilactobacillus salivarius | China | Asia | Lactobacillaceae | Probiotic |
| GCF_009866015.1 | Ligilactobacillus salivarius | China | Asia | Lactobacillaceae | Probiotic |
| GCF_009866085.1 | Ligilactobacillus salivarius | China | Asia | Lactobacillaceae | Probiotic |
| GCF_009866105.1 | Ligilactobacillus salivarius | China | Asia | Lactobacillaceae | Probiotic |
| GCF_009870215.1 | Ligilactobacillus salivarius | China | Asia | Lactobacillaceae | Probiotic |
| GCF_009866115.1 | Ligilactobacillus salivarius | China | Asia | Lactobacillaceae | Probiotic |
| GCF_009870225.1 | Ligilactobacillus salivarius | China | Asia | Lactobacillaceae | Probiotic |
| GCF_009866125.1 | Ligilactobacillus salivarius | China | Asia | Lactobacillaceae | Probiotic |
| GCF_009866185.1 | Ligilactobacillus salivarius | China | Asia | Lactobacillaceae | Probiotic |
| GCF_009870255.1 | Ligilactobacillus salivarius | China | Asia | Lactobacillaceae | Probiotic |
| GCF_009866215.1 | Ligilactobacillus salivarius | China | Asia | Lactobacillaceae | Probiotic |
| GCF_009866205.1 | Ligilactobacillus salivarius | China | Asia | Lactobacillaceae | Probiotic |
| GCF_009866235.1 | Ligilactobacillus salivarius | China | Asia | Lactobacillaceae | Probiotic |
| GCF_009869975.1 | Ligilactobacillus salivarius | China | Asia | Lactobacillaceae | Probiotic |
| GCF_009866265.1 | Ligilactobacillus salivarius | China | Asia | Lactobacillaceae | Probiotic |
| GCF_009863295.1 | Ligilactobacillus salivarius | China | Asia | Lactobacillaceae | Probiotic |
| GCF_009863375.1 | Ligilactobacillus salivarius | China | Asia | Lactobacillaceae | Probiotic |
| GCF_009870295.1 | Ligilactobacillus salivarius | China | Asia | Lactobacillaceae | Probiotic |
| GCF_009866275.1 | Ligilactobacillus salivarius | China | Asia | Lactobacillaceae | Probiotic |
| GCF_009870285.1 | Ligilactobacillus salivarius | China | Asia | Lactobacillaceae | Probiotic |
| GCF_009863365.1 | Ligilactobacillus salivarius | China | Asia | Lactobacillaceae | Probiotic |
| GCF_009863405.1 | Ligilactobacillus salivarius | China | Asia | Lactobacillaceae | Probiotic |
| GCF_009863435.1 | Ligilactobacillus salivarius | China | Asia | Lactobacillaceae | Probiotic |
| GCF_009863415.1 | Ligilactobacillus salivarius | China | Asia | Lactobacillaceae | Probiotic |
| GCF_009863465.1 | Ligilactobacillus salivarius | China | Asia | Lactobacillaceae | Probiotic |
| GCF_009863485.1 | Ligilactobacillus salivarius | China | Asia | Lactobacillaceae | Probiotic |
| GCF_009870365.1 | Ligilactobacillus salivarius | China | Asia | Lactobacillaceae | Probiotic |
| GCF_009863515.1 | Ligilactobacillus salivarius | China | Asia | Lactobacillaceae | Probiotic |
| GCF_009863495.1 | Ligilactobacillus salivarius | China | Asia | Lactobacillaceae | Probiotic |
| GCF_009870355.1 | Ligilactobacillus salivarius | China | Asia | Lactobacillaceae | Probiotic |
| GCF_009863535.1 | Ligilactobacillus salivarius | China | Asia | Lactobacillaceae | Probiotic |
| GCF_009863555.1 | Ligilactobacillus salivarius | China | Asia | Lactobacillaceae | Probiotic |
| GCF_009863595.1 | Ligilactobacillus salivarius | China | Asia | Lactobacillaceae | Probiotic |
| GCF_009863605.1 | Ligilactobacillus salivarius | China | Asia | Lactobacillaceae | Probiotic |
| GCF_009863635.1 | Ligilactobacillus salivarius | China | Asia | Lactobacillaceae | Probiotic |
| GCF_009863585.1 | Ligilactobacillus salivarius | China | Asia | Lactobacillaceae | Probiotic |
| GCF_009863625.1 | Ligilactobacillus salivarius | China | Asia | Lactobacillaceae | Probiotic |
| GCF_009863695.1 | Ligilactobacillus salivarius | China | Asia | Lactobacillaceae | Probiotic |
| GCF_009863685.1 | Ligilactobacillus salivarius | China | Asia | Lactobacillaceae | Probiotic |
| GCF_009863735.1 | Ligilactobacillus salivarius | China | Asia | Lactobacillaceae | Probiotic |
| GCF_009863705.1 | Ligilactobacillus salivarius | China | Asia | Lactobacillaceae | Probiotic |
| GCF_009863715.1 | Ligilactobacillus salivarius | China | Asia | Lactobacillaceae | Probiotic |
| GCF_009863785.1 | Ligilactobacillus salivarius | China | Asia | Lactobacillaceae | Probiotic |
| GCF_009863805.1 | Ligilactobacillus salivarius | China | Asia | Lactobacillaceae | Probiotic |
| GCF_013401855.1 | Ligilactobacillus salivarius | Japan | Asia | Lactobacillaceae | Probiotic |
| GCF_013401965.1 | Ligilactobacillus salivarius | Japan | Asia | Lactobacillaceae | Probiotic |
| GCF_013401955.1 | Ligilactobacillus salivarius | Japan | Asia | Lactobacillaceae | Probiotic |
| GCF_013401895.1 | Ligilactobacillus salivarius | Japan | Asia | Lactobacillaceae | Probiotic |
| GCF_009863815.1 | Ligilactobacillus salivarius | China | Asia | Lactobacillaceae | Probiotic |
| GCF_009863835.1 | Ligilactobacillus salivarius | China | Asia | Lactobacillaceae | Probiotic |
| GCF_009863825.1 | Ligilactobacillus salivarius | China | Asia | Lactobacillaceae | Probiotic |
| GCF_009863885.1 | Ligilactobacillus salivarius | China | Asia | Lactobacillaceae | Probiotic |
| GCF_009863905.1 | Ligilactobacillus salivarius | China | Asia | Lactobacillaceae | Probiotic |
| GCF_030263175.1 | Ligilactobacillus salivarius | India | Asia | Lactobacillaceae | Probiotic |
| GCF_009870335.1 | Ligilactobacillus salivarius | China | Asia | Lactobacillaceae | Probiotic |
| GCF_030372485.1 | Ligilactobacillus salivarius | Missing | Missing | Lactobacillaceae | Probiotic |
| GCF_009863915.1 | Ligilactobacillus salivarius | China | Asia | Lactobacillaceae | Probiotic |
| GCF_002079595.1 | Ligilactobacillus salivarius | Missing | Missing | Lactobacillaceae | Probiotic |
| GCF_014982915.1 | Ligilactobacillus salivarius | Germany | Europe | Lactobacillaceae | Probiotic |
| GCF_003052845.1 | Ligilactobacillus salivarius | USA | North America | Lactobacillaceae | Probiotic |
| GCF_003061585.1 | Ligilactobacillus salivarius | USA | North America | Lactobacillaceae | Probiotic |
| GCF_003052875.1 | Ligilactobacillus salivarius | USA | North America | Lactobacillaceae | Probiotic |
| GCF_003061545.1 | Ligilactobacillus salivarius | USA | North America | Lactobacillaceae | Probiotic |
| GCF_015552175.1 | Ligilactobacillus salivarius | USA | North America | Lactobacillaceae | Probiotic |
| GCF_028746125.1 | Ligilactobacillus salivarius | China | Asia | Lactobacillaceae | Probiotic |
| GCF_001723525.1 | Ligilactobacillus salivarius | China | Asia | Lactobacillaceae | Probiotic |
| GCF_000143435.1 | Ligilactobacillus salivarius | Spain | Europe | Lactobacillaceae | Probiotic |
| GCF_002079625.1 | Ligilactobacillus salivarius | Missing | Missing | Lactobacillaceae | Probiotic |
| GCF_002079655.1 | Ligilactobacillus salivarius | Missing | Missing | Lactobacillaceae | Probiotic |
| GCF_002079645.1 | Ligilactobacillus salivarius | Missing | Missing | Lactobacillaceae | Probiotic |
| GCF_002079685.1 | Ligilactobacillus salivarius | Missing | Missing | Lactobacillaceae | Probiotic |
| GCF_002079705.1 | Ligilactobacillus salivarius | Missing | Missing | Lactobacillaceae | Probiotic |
| GCF_002079715.1 | Ligilactobacillus salivarius | Missing | Missing | Lactobacillaceae | Probiotic |
| GCF_002079745.1 | Ligilactobacillus salivarius | Missing | Missing | Lactobacillaceae | Probiotic |
| GCF_033194755.1 | Ligilactobacillus salivarius | USA | North America | Lactobacillaceae | Probiotic |
| GCF_029070905.1 | Ligilactobacillus salivarius | India | Asia | Lactobacillaceae | Probiotic |
| GCF_030237775.1 | Ligilactobacillus salivarius | China | Asia | Lactobacillaceae | Probiotic |
| GCF_019593595.1 | Ligilactobacillus salivarius | Spain | Europe | Lactobacillaceae | Probiotic |
| GCF_033344095.1 | Ligilactobacillus salivarius | China | Asia | Lactobacillaceae | Probiotic |
| GCF_008016915.1 | Ligilactobacillus salivarius | Spain | Europe | Lactobacillaceae | Probiotic |
| GCF_008016885.1 | Ligilactobacillus salivarius | Spain | Europe | Lactobacillaceae | Probiotic |
| GCF_021266585.1 | Ligilactobacillus salivarius | China | Asia | Lactobacillaceae | Probiotic |
| GCF_013249205.1 | Ligilactobacillus salivarius | Missing | Missing | Lactobacillaceae | Probiotic |
| GCF_033344995.1 | Ligilactobacillus salivarius | Thailand | Asia | Lactobacillaceae | Probiotic |
| GCF_002736025.1 | Ligilactobacillus salivarius | USA | North America | Lactobacillaceae | Probiotic |
| GCF_002735985.1 | Ligilactobacillus salivarius | USA | North America | Lactobacillaceae | Probiotic |
| GCF_035231985.1 | Ligilactobacillus salivarius | South Korea | Asia | Lactobacillaceae | Probiotic |
| GCF_024125465.1 | Ligilactobacillus salivarius | China | Asia | Lactobacillaceae | Probiotic |
| GCF_030876765.1 | Ligilactobacillus salivarius | Missing | North America | Lactobacillaceae | Probiotic |
| GCF_020535185.1 | Ligilactobacillus salivarius | China | Asia | Lactobacillaceae | Probiotic |
| GCF_029917105.1 | Ligilactobacillus salivarius | China | Asia | Lactobacillaceae | Probiotic |
| GCF_021531535.1 | Ligilactobacillus salivarius | Missing | Missing | Lactobacillaceae | Probiotic |
| GCF_002161265.1 | Ligilactobacillus salivarius | Missing | Missing | Lactobacillaceae | Probiotic |
| GCF_016900035.1 | Ligilactobacillus salivarius | Missing | Missing | Lactobacillaceae | Probiotic |
| GCF_016900465.1 | Ligilactobacillus salivarius | Missing | Missing | Lactobacillaceae | Probiotic |
| GCF_002159345.1 | Ligilactobacillus salivarius | Missing | Missing | Lactobacillaceae | Probiotic |
| GCF_016902115.1 | Ligilactobacillus salivarius | Missing | Missing | Lactobacillaceae | Probiotic |
| GCF_027698205.1 | Ligilactobacillus salivarius | China | Asia | Lactobacillaceae | Probiotic |
| GCF_027665935.1 | Ligilactobacillus salivarius | China | Asia | Lactobacillaceae | Probiotic |
| GCF_027668985.1 | Ligilactobacillus salivarius | China | Asia | Lactobacillaceae | Probiotic |
| GCF_003470145.1 | Ligilactobacillus salivarius | China | Asia | Lactobacillaceae | Probiotic |
| GCF_027674525.1 | Ligilactobacillus salivarius | China | Asia | Lactobacillaceae | Probiotic |
| GCF_027674565.1 | Ligilactobacillus salivarius | China | Asia | Lactobacillaceae | Probiotic |
| GCF_003472615.1 | Ligilactobacillus salivarius | China | Asia | Lactobacillaceae | Probiotic |
| GCF_002079785.1 | Ligilactobacillus salivarius | Missing | Missing | Lactobacillaceae | Probiotic |
| GCF_002079765.1 | Ligilactobacillus salivarius | Missing | Missing | Lactobacillaceae | Probiotic |
| GCF_002079845.1 | Ligilactobacillus salivarius | Missing | Missing | Lactobacillaceae | Probiotic |
| GCF_027680645.1 | Ligilactobacillus salivarius | China | Asia | Lactobacillaceae | Probiotic |
| GCF_027681385.1 | Ligilactobacillus salivarius | China | Asia | Lactobacillaceae | Probiotic |
| GCF_027681505.1 | Ligilactobacillus salivarius | China | Asia | Lactobacillaceae | Probiotic |
| GCF_027685795.1 | Ligilactobacillus salivarius | China | Asia | Lactobacillaceae | Probiotic |
| GCF_027685885.1 | Ligilactobacillus salivarius | China | Asia | Lactobacillaceae | Probiotic |
| GCF_027685965.1 | Ligilactobacillus salivarius | China | Asia | Lactobacillaceae | Probiotic |
| GCF_027686005.1 | Ligilactobacillus salivarius | China | Asia | Lactobacillaceae | Probiotic |
| GCF_020026555.1 | Ligilactobacillus salivarius | USA | North America | Lactobacillaceae | Probiotic |
| GCF_020026485.1 | Ligilactobacillus salivarius | USA | North America | Lactobacillaceae | Probiotic |
| GCF_020026515.1 | Ligilactobacillus salivarius | USA | North America | Lactobacillaceae | Probiotic |
| GCF_000179475.1 | Ligilactobacillus salivarius | Missing | Missing | Lactobacillaceae | Probiotic |
| GCF_028864335.1 | Ligilactobacillus salivarius | Russia | Europe | Lactobacillaceae | Probiotic |
| GCF_028864355.1 | Ligilactobacillus salivarius | Russia | Europe | Lactobacillaceae | Probiotic |
| GCF_028864415.1 | Ligilactobacillus salivarius | Russia | Europe | Lactobacillaceae | Probiotic |
| GCF_028864405.1 | Ligilactobacillus salivarius | Russia | Europe | Lactobacillaceae | Probiotic |
| GCF_003129685.1 | Ligilactobacillus salivarius | Argentina | South America | Lactobacillaceae | Probiotic |
| GCF_016742875.1 | Ligilactobacillus salivarius | Canada | North America | Lactobacillaceae | Probiotic |
| GCF_030371315.1 | Ligilactobacillus salivarius | Missing | Missing | Lactobacillaceae | Probiotic |
| GCF_001066665.1 | Ligilactobacillus salivarius | USA | North America | Lactobacillaceae | Probiotic |
| GCF_001067265.1 | Ligilactobacillus salivarius | USA | North America | Lactobacillaceae | Probiotic |
| GCF_001063855.1 | Ligilactobacillus salivarius | USA | North America | Lactobacillaceae | Probiotic |
| GCF_009863925.1 | Ligilactobacillus salivarius | China | Asia | Lactobacillaceae | Probiotic |
| GCF_030371905.1 | Ligilactobacillus salivarius | Missing | Missing | Lactobacillaceae | Probiotic |
| GCF_013487885.1 | Ligilactobacillus salivarius | USA | North America | Lactobacillaceae | Probiotic |
| GCF_002848245.1 | Ligilactobacillus salivarius | Denmark | Europe | Lactobacillaceae | Probiotic |
| GCF_021432185.1 | Ligilactobacillus salivarius | Russia | Europe | Lactobacillaceae | Probiotic |
| GCF_028864445.1 | Ligilactobacillus salivarius | Russia | Europe | Lactobacillaceae | Probiotic |
| GCF_030372045.1 | Ligilactobacillus salivarius | Missing | Missing | Lactobacillaceae | Probiotic |
| GCF_030373745.1 | Ligilactobacillus salivarius | Missing | Missing | Lactobacillaceae | Probiotic |
| GCF_028864385.1 | Ligilactobacillus salivarius | Russia | Europe | Lactobacillaceae | Probiotic |
| GCF_015070845.1 | Ligilactobacillus salivarius | South Korea | Asia | Lactobacillaceae | Probiotic |
| GCF_015557145.1 | Ligilactobacillus salivarius | USA | North America | Lactobacillaceae | Probiotic |
| GCF_015560055.1 | Ligilactobacillus salivarius | USA | North America | Lactobacillaceae | Probiotic |
| GCF_015560415.1 | Ligilactobacillus salivarius | USA | North America | Lactobacillaceae | Probiotic |
| GCF_015548605.1 | Ligilactobacillus salivarius | USA | North America | Lactobacillaceae | Probiotic |
| GCF_028864315.1 | Ligilactobacillus salivarius | Russia | Europe | Lactobacillaceae | Probiotic |
| GCF_002079905.1 | Ligilactobacillus salivarius | Missing | Missing | Lactobacillaceae | Probiotic |
| GCF_947381595.1 | Ligilactobacillus salivarius | Missing | Europe | Lactobacillaceae | Probiotic |
| GCF_947381735.1 | Ligilactobacillus salivarius | Missing | Europe | Lactobacillaceae | Probiotic |
| GCF_925291425.1 | Ligilactobacillus salivarius | Slovenia | Europe | Lactobacillaceae | Probiotic |
| GCF_900094615.1 | Ligilactobacillus salivarius | Missing | Missing | Lactobacillaceae | Probiotic |
| GCF_902385835.1 | Ligilactobacillus salivarius | China | Asia | Lactobacillaceae | Probiotic |
| GCF_001435955.1 | Ligilactobacillus salivarius | USA | North America | Lactobacillaceae | Probiotic |
| GCF_002079585.1 | Ligilactobacillus salivarius | Missing | Missing | Lactobacillaceae | Probiotic |
| GCF_001618905.1 | Limosilactobacillus reuteri | China | Asia | Lactobacillaceae | Probiotic |
| GCF_012971015.1 | Limosilactobacillus reuteri | China | Asia | Lactobacillaceae | Probiotic |
| GCF_012971005.1 | Limosilactobacillus reuteri | China | Asia | Lactobacillaceae | Probiotic |
| GCF_012971025.1 | Limosilactobacillus reuteri | China | Asia | Lactobacillaceae | Probiotic |
| GCF_012970985.1 | Limosilactobacillus reuteri | China | Asia | Lactobacillaceae | Probiotic |
| GCF_006874665.1 | Limosilactobacillus reuteri | China | Asia | Lactobacillaceae | Probiotic |
| GCF_018884225.1 | Limosilactobacillus reuteri | China | Asia | Lactobacillaceae | Probiotic |
| GCF_003072625.1 | Limosilactobacillus reuteri | China | Asia | Lactobacillaceae | Probiotic |
| GCF_020784725.1 | Limosilactobacillus reuteri | Lithuania | Europe | Lactobacillaceae | Probiotic |
| GCF_020784695.1 | Limosilactobacillus reuteri | Lithuania | Europe | Lactobacillaceae | Probiotic |
| GCF_012843635.1 | Limosilactobacillus reuteri | Germany | Europe | Lactobacillaceae | Probiotic |
| GCF_035928175.1 | Limosilactobacillus reuteri | China | Asia | Lactobacillaceae | Probiotic |
| GCF_021228055.1 | Limosilactobacillus reuteri | China | Asia | Lactobacillaceae | Probiotic |
| GCF_021165875.1 | Limosilactobacillus reuteri | China | Asia | Lactobacillaceae | Probiotic |
| GCF_030226325.1 | Limosilactobacillus reuteri | USA | North America | Lactobacillaceae | Probiotic |
| GCF_003719715.1 | Limosilactobacillus reuteri | India | Asia | Lactobacillaceae | Probiotic |
| GCF_020785715.1 | Limosilactobacillus reuteri | Sweden | Europe | Lactobacillaceae | Probiotic |
| GCF_036903235.1 | Limosilactobacillus reuteri | China | Asia | Lactobacillaceae | Probiotic |
| GCF_000722535.2 | Limosilactobacillus reuteri | Germany | Europe | Lactobacillaceae | Probiotic |
| GCF_015377805.1 | Limosilactobacillus reuteri | China | Asia | Lactobacillaceae | Probiotic |
| GCF_000439275.1 | Limosilactobacillus reuteri | USA | North America | Lactobacillaceae | Probiotic |
| GCF_020785665.1 | Limosilactobacillus reuteri | USA | North America | Lactobacillaceae | Probiotic |
| GCF_020785675.1 | Limosilactobacillus reuteri | USA | North America | Lactobacillaceae | Probiotic |
| GCF_028656225.1 | Limosilactobacillus reuteri | South Korea | Asia | Lactobacillaceae | Probiotic |
| GCF_028656195.1 | Limosilactobacillus reuteri | South Korea | Asia | Lactobacillaceae | Probiotic |
| GCF_028656395.1 | Limosilactobacillus reuteri | South Korea | Asia | Lactobacillaceae | Probiotic |
| GCF_028656315.1 | Limosilactobacillus reuteri | South Korea | Asia | Lactobacillaceae | Probiotic |
| GCF_028656325.1 | Limosilactobacillus reuteri | South Korea | Asia | Lactobacillaceae | Probiotic |
| GCF_028656265.1 | Limosilactobacillus reuteri | South Korea | Asia | Lactobacillaceae | Probiotic |
| GCF_025515425.1 | Limosilactobacillus reuteri | South Korea | Asia | Lactobacillaceae | Probiotic |
| GCF_020785595.1 | Limosilactobacillus reuteri | Ireland | Europe | Lactobacillaceae | Probiotic |
| GCF_020785025.1 | Limosilactobacillus reuteri | USA | North America | Lactobacillaceae | Probiotic |
| GCF_003316935.1 | Limosilactobacillus reuteri | South Korea | Asia | Lactobacillaceae | Probiotic |
| GCF_020023755.1 | Limosilactobacillus reuteri | France | Europe | Lactobacillaceae | Probiotic |
| GCF_020023775.1 | Limosilactobacillus reuteri | Italy | Europe | Lactobacillaceae | Probiotic |
| GCF_000159455.2 | Limosilactobacillus reuteri | Peru | South America | Lactobacillaceae | Probiotic |
| GCF_020784395.1 | Limosilactobacillus reuteri | USA | North America | Lactobacillaceae | Probiotic |
| GCF_020784415.1 | Limosilactobacillus reuteri | USA | North America | Lactobacillaceae | Probiotic |
| GCF_020784355.1 | Limosilactobacillus reuteri | USA | North America | Lactobacillaceae | Probiotic |
| GCF_033181025.1 | Limosilactobacillus reuteri | China | Asia | Lactobacillaceae | Probiotic |
| GCF_030721705.1 | Limosilactobacillus reuteri | China | Asia | Lactobacillaceae | Probiotic |
| GCF_002762415.1 | Limosilactobacillus reuteri | India | Asia | Lactobacillaceae | Probiotic |
| GCF_009649095.1 | Limosilactobacillus reuteri | Missing | Asia | Lactobacillaceae | Probiotic |
| GCF_003703875.1 | Limosilactobacillus reuteri | Missing | Europe | Lactobacillaceae | Probiotic |
| GCF_020784465.1 | Limosilactobacillus reuteri | Canada | North America | Lactobacillaceae | Probiotic |
| GCF_020784375.1 | Limosilactobacillus reuteri | Canada | North America | Lactobacillaceae | Probiotic |
| GCF_020784435.1 | Limosilactobacillus reuteri | Canada | North America | Lactobacillaceae | Probiotic |
| GCF_022509245.1 | Limosilactobacillus reuteri | Pakistan | Asia | Lactobacillaceae | Probiotic |
| GCF_009389465.1 | Limosilactobacillus reuteri | Switzerland | Europe | Lactobacillaceae | Probiotic |
| GCF_009389005.1 | Limosilactobacillus reuteri | Switzerland | Europe | Lactobacillaceae | Probiotic |
| GCF_009389035.1 | Limosilactobacillus reuteri | Switzerland | Europe | Lactobacillaceae | Probiotic |
| GCF_009389015.1 | Limosilactobacillus reuteri | Switzerland | Europe | Lactobacillaceae | Probiotic |
| GCF_009389105.1 | Limosilactobacillus reuteri | Switzerland | Europe | Lactobacillaceae | Probiotic |
| GCF_009389115.1 | Limosilactobacillus reuteri | Switzerland | Europe | Lactobacillaceae | Probiotic |
| GCF_009389285.1 | Limosilactobacillus reuteri | Switzerland | Europe | Lactobacillaceae | Probiotic |
| GCF_009389215.1 | Limosilactobacillus reuteri | Switzerland | Europe | Lactobacillaceae | Probiotic |
| GCF_009389255.1 | Limosilactobacillus reuteri | Switzerland | Europe | Lactobacillaceae | Probiotic |
| GCF_009389245.1 | Limosilactobacillus reuteri | Switzerland | Europe | Lactobacillaceae | Probiotic |
| GCF_009389155.1 | Limosilactobacillus reuteri | Switzerland | Europe | Lactobacillaceae | Probiotic |
| GCF_009389205.1 | Limosilactobacillus reuteri | Switzerland | Europe | Lactobacillaceae | Probiotic |
| GCF_009389165.1 | Limosilactobacillus reuteri | Switzerland | Europe | Lactobacillaceae | Probiotic |
| GCF_009389475.1 | Limosilactobacillus reuteri | Switzerland | Europe | Lactobacillaceae | Probiotic |
| GCF_009389305.1 | Limosilactobacillus reuteri | Switzerland | Europe | Lactobacillaceae | Probiotic |
| GCF_009389325.1 | Limosilactobacillus reuteri | Switzerland | Europe | Lactobacillaceae | Probiotic |
| GCF_009389335.1 | Limosilactobacillus reuteri | Switzerland | Europe | Lactobacillaceae | Probiotic |
| GCF_009389375.1 | Limosilactobacillus reuteri | Switzerland | Europe | Lactobacillaceae | Probiotic |
| GCF_009389365.1 | Limosilactobacillus reuteri | Switzerland | Europe | Lactobacillaceae | Probiotic |
| GCF_009389385.1 | Limosilactobacillus reuteri | Switzerland | Europe | Lactobacillaceae | Probiotic |
| GCF_009389435.1 | Limosilactobacillus reuteri | Switzerland | Europe | Lactobacillaceae | Probiotic |
| GCF_009389425.1 | Limosilactobacillus reuteri | Switzerland | Europe | Lactobacillaceae | Probiotic |
| GCF_020785255.1 | Limosilactobacillus reuteri | Papua New Guinea | Oceania | Lactobacillaceae | Probiotic |
| GCF_020785325.1 | Limosilactobacillus reuteri | Papua New Guinea | Oceania | Lactobacillaceae | Probiotic |
| GCF_020785335.1 | Limosilactobacillus reuteri | Papua New Guinea | Oceania | Lactobacillaceae | Probiotic |
| GCF_020785415.1 | Limosilactobacillus reuteri | Papua New Guinea | Oceania | Lactobacillaceae | Probiotic |
| GCF_020785475.1 | Limosilactobacillus reuteri | Papua New Guinea | Oceania | Lactobacillaceae | Probiotic |
| GCF_020784805.1 | Limosilactobacillus reuteri | USA | North America | Lactobacillaceae | Probiotic |
| GCF_020784895.1 | Limosilactobacillus reuteri | USA | North America | Lactobacillaceae | Probiotic |
| GCF_020784875.1 | Limosilactobacillus reuteri | USA | North America | Lactobacillaceae | Probiotic |
| GCF_023896335.1 | Limosilactobacillus reuteri | Russia | Europe | Lactobacillaceae | Probiotic |
| GCF_036621915.1 | Limosilactobacillus reuteri | Missing | North America | Lactobacillaceae | Probiotic |
| GCF_020785295.1 | Limosilactobacillus reuteri | Papua New Guinea | Oceania | Lactobacillaceae | Probiotic |
| GCF_020785355.1 | Limosilactobacillus reuteri | Papua New Guinea | Oceania | Lactobacillaceae | Probiotic |
| GCF_022586935.1 | Limosilactobacillus reuteri | Canada | North America | Lactobacillaceae | Probiotic |
| GCF_001705505.1 | Limosilactobacillus reuteri | USA | North America | Lactobacillaceae | Probiotic |
| GCF_033570435.1 | Limosilactobacillus reuteri | USA | North America | Lactobacillaceae | Probiotic |
| GCF_014336805.1 | Limosilactobacillus reuteri | USA | North America | Lactobacillaceae | Probiotic |
| GCF_009649535.1 | Limosilactobacillus reuteri | Missing | North America | Lactobacillaceae | Probiotic |
| GCF_020784715.1 | Limosilactobacillus reuteri | USA | North America | Lactobacillaceae | Probiotic |
| GCF_020784315.1 | Limosilactobacillus reuteri | USA | North America | Lactobacillaceae | Probiotic |
| GCF_020784755.1 | Limosilactobacillus reuteri | USA | North America | Lactobacillaceae | Probiotic |
| GCF_020784775.1 | Limosilactobacillus reuteri | USA | North America | Lactobacillaceae | Probiotic |
| GCF_004794115.1 | Limosilactobacillus reuteri | Canada | North America | Lactobacillaceae | Probiotic |
| GCF_004793875.1 | Limosilactobacillus reuteri | Canada | North America | Lactobacillaceae | Probiotic |
| GCF_030585585.1 | Limosilactobacillus reuteri | China | Asia | Lactobacillaceae | Probiotic |
| GCF_012971035.1 | Limosilactobacillus reuteri | China | Asia | Lactobacillaceae | Probiotic |
| GCF_012971105.1 | Limosilactobacillus reuteri | China | Asia | Lactobacillaceae | Probiotic |
| GCF_012971095.1 | Limosilactobacillus reuteri | China | Asia | Lactobacillaceae | Probiotic |
| GCF_009649605.1 | Limosilactobacillus reuteri | Missing | Europe | Lactobacillaceae | Probiotic |
| GCF_012971085.1 | Limosilactobacillus reuteri | China | Asia | Lactobacillaceae | Probiotic |
| GCF_012971115.1 | Limosilactobacillus reuteri | China | Asia | Lactobacillaceae | Probiotic |
| GCF_009649475.1 | Limosilactobacillus reuteri | Missing | Europe | Lactobacillaceae | Probiotic |
| GCF_012971125.1 | Limosilactobacillus reuteri | China | Asia | Lactobacillaceae | Probiotic |
| GCF_018917025.1 | Limosilactobacillus reuteri | China | Asia | Lactobacillaceae | Probiotic |
| GCF_012971185.1 | Limosilactobacillus reuteri | China | Asia | Lactobacillaceae | Probiotic |
| GCF_020785395.1 | Limosilactobacillus reuteri | Finland | Europe | Lactobacillaceae | Probiotic |
| GCF_020784835.1 | Limosilactobacillus reuteri | USA | North America | Lactobacillaceae | Probiotic |
| GCF_020784845.1 | Limosilactobacillus reuteri | USA | North America | Lactobacillaceae | Probiotic |
| GCF_000159475.2 | Limosilactobacillus reuteri | Missing | Europe | Lactobacillaceae | Probiotic |
| GCF_020785455.1 | Limosilactobacillus reuteri | Finland | Europe | Lactobacillaceae | Probiotic |
| GCF_002112805.1 | Limosilactobacillus reuteri | Missing | Europe | Lactobacillaceae | Probiotic |
| GCF_000160715.1 | Limosilactobacillus reuteri | Missing | Europe | Lactobacillaceae | Probiotic |
| GCF_000179435.1 | Limosilactobacillus reuteri | Missing | North America | Lactobacillaceae | Probiotic |
| GCF_020785535.1 | Limosilactobacillus reuteri | Japan | Asia | Lactobacillaceae | Probiotic |
| GCF_020785505.1 | Limosilactobacillus reuteri | Japan | Asia | Lactobacillaceae | Probiotic |
| GCF_010206285.1 | Limosilactobacillus reuteri | France | Europe | Lactobacillaceae | Probiotic |
| GCF_002007085.2 | Limosilactobacillus reuteri | Russia | Europe | Lactobacillaceae | Probiotic |
| GCF_901600675.1 | Limosilactobacillus reuteri | France | Europe | Lactobacillaceae | Probiotic |
| GCF_901600695.1 | Limosilactobacillus reuteri | France | Europe | Lactobacillaceae | Probiotic |
| GCF_901600705.1 | Limosilactobacillus reuteri | France | Europe | Lactobacillaceae | Probiotic |
| GCF_901600665.1 | Limosilactobacillus reuteri | France | Europe | Lactobacillaceae | Probiotic |
| GCF_020785435.1 | Limosilactobacillus reuteri | Japan | Asia | Lactobacillaceae | Probiotic |
| GCF_002112195.1 | Limosilactobacillus reuteri | South Africa | Africa | Lactobacillaceae | Probiotic |
| GCF_021459965.1 | Limosilactobacillus reuteri | China | Asia | Lactobacillaceae | Probiotic |
| GCF_030345055.1 | Limosilactobacillus reuteri | China | Asia | Lactobacillaceae | Probiotic |
| GCF_020784915.1 | Limosilactobacillus reuteri | USA | North America | Lactobacillaceae | Probiotic |
| GCF_020784935.1 | Limosilactobacillus reuteri | USA | North America | Lactobacillaceae | Probiotic |
| GCF_020784275.1 | Limosilactobacillus reuteri | Japan | Asia | Lactobacillaceae | Probiotic |
| GCF_020784305.1 | Limosilactobacillus reuteri | Japan | Asia | Lactobacillaceae | Probiotic |
| GCF_020784295.1 | Limosilactobacillus reuteri | Japan | Asia | Lactobacillaceae | Probiotic |
| GCF_020784235.1 | Limosilactobacillus reuteri | USA | North America | Lactobacillaceae | Probiotic |
| GCF_020785015.1 | Limosilactobacillus reuteri | USA | North America | Lactobacillaceae | Probiotic |
| GCF_020785035.1 | Limosilactobacillus reuteri | USA | North America | Lactobacillaceae | Probiotic |
| GCF_020785095.1 | Limosilactobacillus reuteri | USA | North America | Lactobacillaceae | Probiotic |
| GCF_000758185.1 | Limosilactobacillus reuteri | Germany | Europe | Lactobacillaceae | Probiotic |
| GCF_036432135.1 | Limosilactobacillus reuteri | Ghana | Africa | Lactobacillaceae | Probiotic |
| GCF_036432025.1 | Limosilactobacillus reuteri | Ghana | Africa | Lactobacillaceae | Probiotic |
| GCF_036323925.1 | Limosilactobacillus reuteri | China | Asia | Lactobacillaceae | Probiotic |
| GCF_003174865.1 | Limosilactobacillus reuteri | China | Asia | Lactobacillaceae | Probiotic |
| GCF_003174875.1 | Limosilactobacillus reuteri | China | Asia | Lactobacillaceae | Probiotic |
| GCF_003175085.1 | Limosilactobacillus reuteri | China | Asia | Lactobacillaceae | Probiotic |
| GCF_003175115.1 | Limosilactobacillus reuteri | China | Asia | Lactobacillaceae | Probiotic |
| GCF_009649565.1 | Limosilactobacillus reuteri | Missing | North America | Lactobacillaceae | Probiotic |
| GCF_003174855.1 | Limosilactobacillus reuteri | China | Asia | Lactobacillaceae | Probiotic |
| GCF_003174935.1 | Limosilactobacillus reuteri | China | Asia | Lactobacillaceae | Probiotic |
| GCF_003175015.1 | Limosilactobacillus reuteri | China | Asia | Lactobacillaceae | Probiotic |
| GCF_003175025.1 | Limosilactobacillus reuteri | China | Asia | Lactobacillaceae | Probiotic |
| GCF_003174815.1 | Limosilactobacillus reuteri | China | Asia | Lactobacillaceae | Probiotic |
| GCF_003174915.1 | Limosilactobacillus reuteri | China | Asia | Lactobacillaceae | Probiotic |
| GCF_003174955.1 | Limosilactobacillus reuteri | China | Asia | Lactobacillaceae | Probiotic |
| GCF_003174945.1 | Limosilactobacillus reuteri | China | Asia | Lactobacillaceae | Probiotic |
| GCF_003175035.1 | Limosilactobacillus reuteri | China | Asia | Lactobacillaceae | Probiotic |
| GCF_003174995.1 | Limosilactobacillus reuteri | China | Asia | Lactobacillaceae | Probiotic |
| GCF_003175075.1 | Limosilactobacillus reuteri | China | Asia | Lactobacillaceae | Probiotic |
| GCF_003175125.1 | Limosilactobacillus reuteri | China | Asia | Lactobacillaceae | Probiotic |
| GCF_018966925.1 | Limosilactobacillus reuteri | Russia | Europe | Lactobacillaceae | Probiotic |
| GCF_002156605.1 | Limosilactobacillus reuteri | USA | North America | Lactobacillaceae | Probiotic |
| GCF_003413665.1 | Limosilactobacillus reuteri | China | Asia | Lactobacillaceae | Probiotic |
| GCF_036621875.1 | Limosilactobacillus reuteri | Missing | North America | Lactobacillaceae | Probiotic |
| GCF_020785575.1 | Limosilactobacillus reuteri | USA | North America | Lactobacillaceae | Probiotic |
| GCF_011065435.1 | Limosilactobacillus reuteri | Italy | Europe | Lactobacillaceae | Probiotic |
| GCF_036431965.1 | Limosilactobacillus reuteri | Ghana | Africa | Lactobacillaceae | Probiotic |
| GCF_021229095.1 | Limosilactobacillus reuteri | Bulgaria | Europe | Lactobacillaceae | Probiotic |
| GCF_007633215.1 | Limosilactobacillus reuteri | USA | North America | Lactobacillaceae | Probiotic |
| GCF_022642805.1 | Limosilactobacillus reuteri | Canada | North America | Lactobacillaceae | Probiotic |
| GCF_900093565.1 | Limosilactobacillus reuteri | Missing | North America | Lactobacillaceae | Probiotic |
| GCF_020785815.1 | Limosilactobacillus reuteri | Brazil | South America | Lactobacillaceae | Probiotic |
| GCF_009649545.1 | Limosilactobacillus reuteri | Missing | North America | Lactobacillaceae | Probiotic |
| GCF_009649445.1 | Limosilactobacillus reuteri | Missing | North America | Lactobacillaceae | Probiotic |
| GCF_002128705.1 | Limosilactobacillus reuteri | South Korea | Asia | Lactobacillaceae | Probiotic |
| GCF_002128765.1 | Limosilactobacillus reuteri | South Korea | Asia | Lactobacillaceae | Probiotic |
| GCF_002128685.1 | Limosilactobacillus reuteri | South Korea | Asia | Lactobacillaceae | Probiotic |
| GCF_002128655.1 | Limosilactobacillus reuteri | South Korea | Asia | Lactobacillaceae | Probiotic |
| GCF_002128635.1 | Limosilactobacillus reuteri | South Korea | Asia | Lactobacillaceae | Probiotic |
| GCF_002128615.1 | Limosilactobacillus reuteri | South Korea | Asia | Lactobacillaceae | Probiotic |
| GCF_002128745.1 | Limosilactobacillus reuteri | South Korea | Asia | Lactobacillaceae | Probiotic |
| GCF_002128605.1 | Limosilactobacillus reuteri | South Korea | Asia | Lactobacillaceae | Probiotic |
| GCF_002128755.1 | Limosilactobacillus reuteri | South Korea | Asia | Lactobacillaceae | Probiotic |
| GCF_002128715.1 | Limosilactobacillus reuteri | South Korea | Asia | Lactobacillaceae | Probiotic |
| GCF_002128585.1 | Limosilactobacillus reuteri | South Korea | Asia | Lactobacillaceae | Probiotic |
| GCF_002128555.1 | Limosilactobacillus reuteri | South Korea | Asia | Lactobacillaceae | Probiotic |
| GCF_002128495.1 | Limosilactobacillus reuteri | South Korea | Asia | Lactobacillaceae | Probiotic |
| GCF_002128525.1 | Limosilactobacillus reuteri | South Korea | Asia | Lactobacillaceae | Probiotic |
| GCF_002128515.1 | Limosilactobacillus reuteri | South Korea | Asia | Lactobacillaceae | Probiotic |
| GCF_002128485.1 | Limosilactobacillus reuteri | South Korea | Asia | Lactobacillaceae | Probiotic |
| GCF_034744995.1 | Limosilactobacillus reuteri | USA | North America | Lactobacillaceae | Probiotic |
| GCF_034745035.1 | Limosilactobacillus reuteri | USA | North America | Lactobacillaceae | Probiotic |
| GCF_034745005.1 | Limosilactobacillus reuteri | USA | North America | Lactobacillaceae | Probiotic |
| GCF_034745055.1 | Limosilactobacillus reuteri | USA | North America | Lactobacillaceae | Probiotic |
| GCF_034745075.1 | Limosilactobacillus reuteri | USA | North America | Lactobacillaceae | Probiotic |
| GCF_034745095.1 | Limosilactobacillus reuteri | USA | North America | Lactobacillaceae | Probiotic |
| GCF_034745115.1 | Limosilactobacillus reuteri | USA | North America | Lactobacillaceae | Probiotic |
| GCF_034745135.1 | Limosilactobacillus reuteri | USA | North America | Lactobacillaceae | Probiotic |
| GCF_034745155.1 | Limosilactobacillus reuteri | USA | North America | Lactobacillaceae | Probiotic |
| GCF_034745175.1 | Limosilactobacillus reuteri | USA | North America | Lactobacillaceae | Probiotic |
| GCF_034745195.1 | Limosilactobacillus reuteri | USA | North America | Lactobacillaceae | Probiotic |
| GCF_034745215.1 | Limosilactobacillus reuteri | USA | North America | Lactobacillaceae | Probiotic |
| GCF_034745275.1 | Limosilactobacillus reuteri | USA | North America | Lactobacillaceae | Probiotic |
| GCF_034745245.1 | Limosilactobacillus reuteri | USA | North America | Lactobacillaceae | Probiotic |
| GCF_034745295.1 | Limosilactobacillus reuteri | USA | North America | Lactobacillaceae | Probiotic |
| GCF_034745315.1 | Limosilactobacillus reuteri | USA | North America | Lactobacillaceae | Probiotic |
| GCF_034745355.1 | Limosilactobacillus reuteri | USA | North America | Lactobacillaceae | Probiotic |
| GCF_034745375.1 | Limosilactobacillus reuteri | USA | North America | Lactobacillaceae | Probiotic |
| GCF_034745335.1 | Limosilactobacillus reuteri | USA | North America | Lactobacillaceae | Probiotic |
| GCF_034745415.1 | Limosilactobacillus reuteri | USA | North America | Lactobacillaceae | Probiotic |
| GCF_034745395.1 | Limosilactobacillus reuteri | USA | North America | Lactobacillaceae | Probiotic |
| GCF_034745435.1 | Limosilactobacillus reuteri | USA | North America | Lactobacillaceae | Probiotic |
| GCF_002112225.1 | Limosilactobacillus reuteri | Japan | Asia | Lactobacillaceae | Probiotic |
| GCF_001046835.1 | Limosilactobacillus reuteri | South Korea | Asia | Lactobacillaceae | Probiotic |
| GCF_901971765.1 | Limosilactobacillus reuteri | Spain | Europe | Lactobacillaceae | Probiotic |
| GCF_034259105.1 | Limosilactobacillus reuteri | China | Asia | Lactobacillaceae | Probiotic |
| GCF_004684995.1 | Limosilactobacillus reuteri | USA | North America | Lactobacillaceae | Probiotic |
| GCF_000410995.1 | Limosilactobacillus reuteri | China | Asia | Lactobacillaceae | Probiotic |
| GCF_002221655.1 | Limosilactobacillus reuteri | Switzerland | Europe | Lactobacillaceae | Probiotic |
| GCF_001688685.2 | Limosilactobacillus reuteri | Switzerland | Europe | Lactobacillaceae | Probiotic |
| GCF_016697045.1 | Limosilactobacillus reuteri | France | Europe | Lactobacillaceae | Probiotic |
| GCF_020785765.1 | Limosilactobacillus reuteri | USA | North America | Lactobacillaceae | Probiotic |
| GCF_020785795.1 | Limosilactobacillus reuteri | USA | North America | Lactobacillaceae | Probiotic |
| GCF_020785755.1 | Limosilactobacillus reuteri | USA | North America | Lactobacillaceae | Probiotic |
| GCF_020785835.1 | Limosilactobacillus reuteri | USA | North America | Lactobacillaceae | Probiotic |
| GCF_020785865.1 | Limosilactobacillus reuteri | USA | North America | Lactobacillaceae | Probiotic |
| GCF_020785855.1 | Limosilactobacillus reuteri | USA | North America | Lactobacillaceae | Probiotic |
| GCF_009733645.1 | Limosilactobacillus reuteri | Ecuador | South America | Lactobacillaceae | Probiotic |
| GCF_009733655.1 | Limosilactobacillus reuteri | Ecuador | South America | Lactobacillaceae | Probiotic |
| GCF_009733615.2 | Limosilactobacillus reuteri | Ecuador | South America | Lactobacillaceae | Probiotic |
| GCF_020785075.1 | Limosilactobacillus reuteri | USA | North America | Lactobacillaceae | Probiotic |
| GCF_020784495.1 | Limosilactobacillus reuteri | Canada | North America | Lactobacillaceae | Probiotic |
| GCF_020784515.1 | Limosilactobacillus reuteri | Canada | North America | Lactobacillaceae | Probiotic |
| GCF_019336465.1 | Limosilactobacillus reuteri | China | Asia | Lactobacillaceae | Probiotic |
| GCF_024652885.1 | Limosilactobacillus reuteri | China | Asia | Lactobacillaceae | Probiotic |
| GCF_020785495.1 | Limosilactobacillus reuteri | Japan | Asia | Lactobacillaceae | Probiotic |
| GCF_036431005.1 | Limosilactobacillus reuteri | Ghana | Africa | Lactobacillaceae | Probiotic |
| GCF_036431025.1 | Limosilactobacillus reuteri | Ghana | Africa | Lactobacillaceae | Probiotic |
| GCF_036430925.1 | Limosilactobacillus reuteri | Ghana | Africa | Lactobacillaceae | Probiotic |
| GCF_036430905.1 | Limosilactobacillus reuteri | Ghana | Africa | Lactobacillaceae | Probiotic |
| GCF_036431085.1 | Limosilactobacillus reuteri | Ghana | Africa | Lactobacillaceae | Probiotic |
| GCF_036430965.1 | Limosilactobacillus reuteri | Ghana | Africa | Lactobacillaceae | Probiotic |
| GCF_036430985.1 | Limosilactobacillus reuteri | Ghana | Africa | Lactobacillaceae | Probiotic |
| GCF_023078415.1 | Limosilactobacillus reuteri | South Korea | Asia | Lactobacillaceae | Probiotic |
| GCF_020785895.1 | Limosilactobacillus reuteri | USA | North America | Lactobacillaceae | Probiotic |
| GCF_020785995.1 | Limosilactobacillus reuteri | USA | North America | Lactobacillaceae | Probiotic |
| GCF_020785915.1 | Limosilactobacillus reuteri | USA | North America | Lactobacillaceae | Probiotic |
| GCF_003046135.1 | Limosilactobacillus reuteri | Turkey | Asia | Lactobacillaceae | Probiotic |
| GCF_019061135.1 | Limosilactobacillus reuteri | Slovakia | Europe | Lactobacillaceae | Probiotic |
| GCF_024622285.1 | Limosilactobacillus reuteri | Germany | Europe | Lactobacillaceae | Probiotic |
| GCF_024622305.1 | Limosilactobacillus reuteri | Germany | Europe | Lactobacillaceae | Probiotic |
| GCF_024622315.1 | Limosilactobacillus reuteri | Germany | Europe | Lactobacillaceae | Probiotic |
| GCF_003064585.1 | Limosilactobacillus reuteri | USA | North America | Lactobacillaceae | Probiotic |
| GCF_003065345.1 | Limosilactobacillus reuteri | USA | North America | Lactobacillaceae | Probiotic |
| GCF_003061685.1 | Limosilactobacillus reuteri | USA | North America | Lactobacillaceae | Probiotic |
| GCF_003061745.1 | Limosilactobacillus reuteri | USA | North America | Lactobacillaceae | Probiotic |
| GCF_003053005.1 | Limosilactobacillus reuteri | USA | North America | Lactobacillaceae | Probiotic |
| GCF_021398615.1 | Limosilactobacillus reuteri | South Korea | Asia | Lactobacillaceae | Probiotic |
| GCF_011009765.1 | Limosilactobacillus reuteri | United Kingdom | Europe | Lactobacillaceae | Probiotic |
| GCF_020784635.1 | Limosilactobacillus reuteri | Canada | North America | Lactobacillaceae | Probiotic |
| GCF_020784555.1 | Limosilactobacillus reuteri | Canada | North America | Lactobacillaceae | Probiotic |
| GCF_037414455.1 | Limosilactobacillus reuteri | South Korea | Asia | Lactobacillaceae | Probiotic |
| GCF_002112245.1 | Limosilactobacillus reuteri | USA | North America | Lactobacillaceae | Probiotic |
| GCF_001657495.1 | Limosilactobacillus reuteri | Germany | Europe | Lactobacillaceae | Probiotic |
| GCF_009649505.1 | Limosilactobacillus reuteri | Missing | North America | Lactobacillaceae | Probiotic |
| GCF_020785735.1 | Limosilactobacillus reuteri | Missing | South America | Lactobacillaceae | Probiotic |
| GCF_020785635.1 | Limosilactobacillus reuteri | Missing | South America | Lactobacillaceae | Probiotic |
| GCF_020785655.1 | Limosilactobacillus reuteri | Missing | Africa | Lactobacillaceae | Probiotic |
| GCF_012275185.1 | Limosilactobacillus reuteri | Nigeria | Africa | Lactobacillaceae | Probiotic |
| GCF_013694365.1 | Limosilactobacillus reuteri | Nigeria | Africa | Lactobacillaceae | Probiotic |
| GCF_028369155.1 | Limosilactobacillus reuteri | China | Asia | Lactobacillaceae | Probiotic |
| GCF_025189765.1 | Limosilactobacillus reuteri | France | Europe | Lactobacillaceae | Probiotic |
| GCF_025189785.1 | Limosilactobacillus reuteri | Italy | Europe | Lactobacillaceae | Probiotic |
| GCF_025189725.1 | Limosilactobacillus reuteri | France | Europe | Lactobacillaceae | Probiotic |
| GCF_025189695.1 | Limosilactobacillus reuteri | USA | North America | Lactobacillaceae | Probiotic |
| GCF_025189625.1 | Limosilactobacillus reuteri | Missing | Missing | Lactobacillaceae | Probiotic |
| GCF_025189805.1 | Limosilactobacillus reuteri | Italy | Europe | Lactobacillaceae | Probiotic |
| GCF_025189745.1 | Limosilactobacillus reuteri | France | Europe | Lactobacillaceae | Probiotic |
| GCF_025189645.1 | Limosilactobacillus reuteri | Missing | Missing | Lactobacillaceae | Probiotic |
| GCF_000159615.1 | Limosilactobacillus reuteri | Missing | Europe | Lactobacillaceae | Probiotic |
| GCF_002027295.1 | Limosilactobacillus reuteri | Spain | Europe | Lactobacillaceae | Probiotic |
| GCF_004349655.1 | Limosilactobacillus reuteri | USA | North America | Lactobacillaceae | Probiotic |
| GCF_004349685.1 | Limosilactobacillus reuteri | USA | North America | Lactobacillaceae | Probiotic |
| GCF_035200785.1 | Limosilactobacillus reuteri | China | Asia | Lactobacillaceae | Probiotic |
| GCF_020784595.1 | Limosilactobacillus reuteri | Canada | North America | Lactobacillaceae | Probiotic |
| GCF_034424825.1 | Limosilactobacillus reuteri | South Africa | Africa | Lactobacillaceae | Probiotic |
| GCF_020784605.1 | Limosilactobacillus reuteri | Canada | North America | Lactobacillaceae | Probiotic |
| GCF_020784575.1 | Limosilactobacillus reuteri | Canada | North America | Lactobacillaceae | Probiotic |
| GCF_003316895.1 | Limosilactobacillus reuteri | South Korea | Asia | Lactobacillaceae | Probiotic |
| GCF_015552675.1 | Limosilactobacillus reuteri | USA | North America | Lactobacillaceae | Probiotic |
| GCF_026183435.1 | Limosilactobacillus reuteri | South Korea | Asia | Lactobacillaceae | Probiotic |
| GCF_022511545.1 | Limosilactobacillus reuteri | USA | North America | Lactobacillaceae | Probiotic |
| GCF_030262475.1 | Limosilactobacillus reuteri | France | Europe | Lactobacillaceae | Probiotic |
| GCF_007280535.1 | Limosilactobacillus reuteri | France | Europe | Lactobacillaceae | Probiotic |
| GCF_020784195.1 | Limosilactobacillus reuteri | USA | North America | Lactobacillaceae | Probiotic |
| GCF_020785145.1 | Limosilactobacillus reuteri | USA | North America | Lactobacillaceae | Probiotic |
| GCF_020785135.1 | Limosilactobacillus reuteri | USA | North America | Lactobacillaceae | Probiotic |
| GCF_020785115.1 | Limosilactobacillus reuteri | USA | North America | Lactobacillaceae | Probiotic |
| GCF_004208615.1 | Limosilactobacillus reuteri | South Korea | Asia | Lactobacillaceae | Probiotic |
| GCF_030418275.1 | Limosilactobacillus reuteri | Finland | Europe | Lactobacillaceae | Probiotic |
| GCF_020784795.1 | Limosilactobacillus reuteri | Sweden | Europe | Lactobacillaceae | Probiotic |
| GCF_020785975.1 | Limosilactobacillus reuteri | USA | North America | Lactobacillaceae | Probiotic |
| GCF_014145445.1 | Limosilactobacillus reuteri | USA | North America | Lactobacillaceae | Probiotic |
| GCF_020786015.1 | Limosilactobacillus reuteri | USA | North America | Lactobacillaceae | Probiotic |
| GCF_020786095.1 | Limosilactobacillus reuteri | USA | North America | Lactobacillaceae | Probiotic |
| GCF_016900715.1 | Limosilactobacillus reuteri | Czech Republic | Europe | Lactobacillaceae | Probiotic |
| GCF_002159305.1 | Limosilactobacillus reuteri | Czech Republic | Europe | Lactobacillaceae | Probiotic |
| GCF_013348825.1 | Limosilactobacillus reuteri | South Korea | Asia | Lactobacillaceae | Probiotic |
| GCF_002160565.1 | Limosilactobacillus reuteri | Czech Republic | Europe | Lactobacillaceae | Probiotic |
| GCF_025369755.1 | Limosilactobacillus reuteri | USA | North America | Lactobacillaceae | Probiotic |
| GCF_020785185.1 | Limosilactobacillus reuteri | USA | North America | Lactobacillaceae | Probiotic |
| GCF_020784205.1 | Limosilactobacillus reuteri | USA | North America | Lactobacillaceae | Probiotic |
| GCF_020785175.1 | Limosilactobacillus reuteri | USA | North America | Lactobacillaceae | Probiotic |
| GCF_020785305.1 | Limosilactobacillus reuteri | USA | North America | Lactobacillaceae | Probiotic |
| GCF_009649595.1 | Limosilactobacillus reuteri | Missing | Europe | Lactobacillaceae | Probiotic |
| GCF_028462705.1 | Limosilactobacillus reuteri | Germany | Europe | Lactobacillaceae | Probiotic |
| GCF_028462635.1 | Limosilactobacillus reuteri | Germany | Europe | Lactobacillaceae | Probiotic |
| GCF_028462605.1 | Limosilactobacillus reuteri | Germany | Europe | Lactobacillaceae | Probiotic |
| GCF_020784945.1 | Limosilactobacillus reuteri | Canada | North America | Lactobacillaceae | Probiotic |
| GCF_020784975.1 | Limosilactobacillus reuteri | Canada | North America | Lactobacillaceae | Probiotic |
| GCF_020784995.1 | Limosilactobacillus reuteri | Canada | North America | Lactobacillaceae | Probiotic |
| GCF_009649645.1 | Limosilactobacillus reuteri | Missing | North America | Lactobacillaceae | Probiotic |
| GCF_002253665.1 | Limosilactobacillus reuteri | France | Europe | Lactobacillaceae | Probiotic |
| GCF_002253625.1 | Limosilactobacillus reuteri | France | Europe | Lactobacillaceae | Probiotic |
| GCF_002253685.1 | Limosilactobacillus reuteri | France | Europe | Lactobacillaceae | Probiotic |
| GCF_002253705.1 | Limosilactobacillus reuteri | France | Europe | Lactobacillaceae | Probiotic |
| GCF_002253745.1 | Limosilactobacillus reuteri | France | Europe | Lactobacillaceae | Probiotic |
| GCF_002253725.1 | Limosilactobacillus reuteri | France | Europe | Lactobacillaceae | Probiotic |
| GCF_002253755.1 | Limosilactobacillus reuteri | France | Europe | Lactobacillaceae | Probiotic |
| GCF_020786045.1 | Limosilactobacillus reuteri | Canada | North America | Lactobacillaceae | Probiotic |
| GCF_001703935.1 | Limosilactobacillus reuteri | Israel | Asia | Lactobacillaceae | Probiotic |
| GCF_001703875.1 | Limosilactobacillus reuteri | Israel | Asia | Lactobacillaceae | Probiotic |
| GCF_001703885.1 | Limosilactobacillus reuteri | Israel | Asia | Lactobacillaceae | Probiotic |
| GCF_001703865.1 | Limosilactobacillus reuteri | Israel | Asia | Lactobacillaceae | Probiotic |
| GCF_001703855.1 | Limosilactobacillus reuteri | Israel | Asia | Lactobacillaceae | Probiotic |
| GCF_020784525.1 | Limosilactobacillus reuteri | Canada | North America | Lactobacillaceae | Probiotic |
| GCF_020784255.1 | Limosilactobacillus reuteri | Canada | North America | Lactobacillaceae | Probiotic |
| GCF_020785215.1 | Limosilactobacillus reuteri | Canada | North America | Lactobacillaceae | Probiotic |
| GCF_020785225.1 | Limosilactobacillus reuteri | Canada | North America | Lactobacillaceae | Probiotic |
| GCF_020785275.1 | Limosilactobacillus reuteri | Canada | North America | Lactobacillaceae | Probiotic |
| GCF_002888655.1 | Limosilactobacillus reuteri | Germany | Europe | Lactobacillaceae | Probiotic |
| GCF_003703885.1 | Limosilactobacillus reuteri | Missing | North America | Lactobacillaceae | Probiotic |
| GCF_013487925.1 | Limosilactobacillus reuteri | USA | North America | Lactobacillaceae | Probiotic |
| GCF_020412465.1 | Limosilactobacillus reuteri | United Kingdom | Europe | Lactobacillaceae | Probiotic |
| GCF_020412485.1 | Limosilactobacillus reuteri | United Kingdom | Europe | Lactobacillaceae | Probiotic |
| GCF_002112185.1 | Limosilactobacillus reuteri | Denmark | Europe | Lactobacillaceae | Probiotic |
| GCF_001889975.1 | Limosilactobacillus reuteri | Netherlands | Europe | Lactobacillaceae | Probiotic |
| GCF_020785935.1 | Limosilactobacillus reuteri | Canada | North America | Lactobacillaceae | Probiotic |
| GCF_020785945.1 | Limosilactobacillus reuteri | Canada | North America | Lactobacillaceae | Probiotic |
| GCF_002253765.1 | Limosilactobacillus reuteri | United Kingdom | Europe | Lactobacillaceae | Probiotic |
| GCF_002253785.1 | Limosilactobacillus reuteri | United Kingdom | Europe | Lactobacillaceae | Probiotic |
| GCF_002253875.1 | Limosilactobacillus reuteri | United Kingdom | Europe | Lactobacillaceae | Probiotic |
| GCF_002253825.1 | Limosilactobacillus reuteri | France | Europe | Lactobacillaceae | Probiotic |
| GCF_002253835.1 | Limosilactobacillus reuteri | France | Europe | Lactobacillaceae | Probiotic |
| GCF_002253905.1 | Limosilactobacillus reuteri | United Kingdom | Europe | Lactobacillaceae | Probiotic |
| GCF_002253945.1 | Limosilactobacillus reuteri | United Kingdom | Europe | Lactobacillaceae | Probiotic |
| GCF_002253975.1 | Limosilactobacillus reuteri | United Kingdom | Europe | Lactobacillaceae | Probiotic |
| GCF_002253965.1 | Limosilactobacillus reuteri | United Kingdom | Europe | Lactobacillaceae | Probiotic |
| GCF_002253925.1 | Limosilactobacillus reuteri | France | Europe | Lactobacillaceae | Probiotic |
| GCF_002253955.1 | Limosilactobacillus reuteri | France | Europe | Lactobacillaceae | Probiotic |
| GCF_002254035.1 | Limosilactobacillus reuteri | France | Europe | Lactobacillaceae | Probiotic |
| GCF_002254095.1 | Limosilactobacillus reuteri | United Kingdom | Europe | Lactobacillaceae | Probiotic |
| GCF_002254025.1 | Limosilactobacillus reuteri | United Kingdom | Europe | Lactobacillaceae | Probiotic |
| GCF_002254045.1 | Limosilactobacillus reuteri | United Kingdom | Europe | Lactobacillaceae | Probiotic |
| GCF_002254105.1 | Limosilactobacillus reuteri | United Kingdom | Europe | Lactobacillaceae | Probiotic |
| GCF_002254085.1 | Limosilactobacillus reuteri | United Kingdom | Europe | Lactobacillaceae | Probiotic |
| GCF_002254115.1 | Limosilactobacillus reuteri | United Kingdom | Europe | Lactobacillaceae | Probiotic |
| GCF_002254175.1 | Limosilactobacillus reuteri | United Kingdom | Europe | Lactobacillaceae | Probiotic |
| GCF_002254165.1 | Limosilactobacillus reuteri | United Kingdom | Europe | Lactobacillaceae | Probiotic |
| GCF_002254185.1 | Limosilactobacillus reuteri | United Kingdom | Europe | Lactobacillaceae | Probiotic |
| GCF_002254195.1 | Limosilactobacillus reuteri | United Kingdom | Europe | Lactobacillaceae | Probiotic |
| GCF_002254245.1 | Limosilactobacillus reuteri | United Kingdom | Europe | Lactobacillaceae | Probiotic |
| GCF_002254255.1 | Limosilactobacillus reuteri | United Kingdom | Europe | Lactobacillaceae | Probiotic |
| GCF_009649105.1 | Limosilactobacillus reuteri | New Zealand | Oceania | Lactobacillaceae | Probiotic |
| GCF_030517815.1 | Limosilactobacillus reuteri | China | Asia | Lactobacillaceae | Probiotic |
| GCF_947381675.1 | Limosilactobacillus reuteri | Germany | Europe | Lactobacillaceae | Probiotic |
| GCF_947381575.1 | Limosilactobacillus reuteri | Germany | Europe | Lactobacillaceae | Probiotic |
| GCF_925280345.1 | Limosilactobacillus reuteri | Slovenia | Europe | Lactobacillaceae | Probiotic |
| GCF_925285275.1 | Limosilactobacillus reuteri | Missing | Europe | Lactobacillaceae | Probiotic |
| GCF_940926095.1 | Limosilactobacillus reuteri | Belgium | Europe | Lactobacillaceae | Probiotic |
| GCF_910576155.1 | Limosilactobacillus reuteri | United Kingdom | Europe | Lactobacillaceae | Probiotic |
| GCF_910574215.1 | Limosilactobacillus reuteri | United Kingdom | Europe | Lactobacillaceae | Probiotic |
| GCF_910574795.1 | Limosilactobacillus reuteri | United Kingdom | Europe | Lactobacillaceae | Probiotic |
| GCF_910574445.1 | Limosilactobacillus reuteri | United Kingdom | Europe | Lactobacillaceae | Probiotic |
| GCF_020978285.1 | Limosilactobacillus reuteri | Germany | Europe | Lactobacillaceae | Probiotic |
| GCF_020978225.1 | Limosilactobacillus reuteri | Germany | Europe | Lactobacillaceae | Probiotic |
| GCF_910574725.1 | Limosilactobacillus reuteri | United Kingdom | Europe | Lactobacillaceae | Probiotic |
| GCF_000016825.1 | Limosilactobacillus reuteri | Missing | Europe | Lactobacillaceae | Probiotic |
| GCF_000179455.1 | Limosilactobacillus reuteri | Missing | North America | Lactobacillaceae | Probiotic |
| GCF_036621895.1 | Limosilactobacillus reuteri | Missing | Oceania | Lactobacillaceae | Probiotic |
| GCF_000168255.1 | Limosilactobacillus reuteri | New Zealand | Oceania | Lactobacillaceae | Probiotic |
| GCF_026935945.1 | Limosilactobacillus vaginalis | Brazil | South America | Lactobacillaceae | Probiotic |
| GCF_009362935.1 | Limosilactobacillus vaginalis | Korea | Asia | Lactobacillaceae | Probiotic |
| GCF_028607005.1 | Limosilactobacillus vaginalis | USA | North America | Lactobacillaceae | Probiotic |
| GCF_025677785.1 | Limosilactobacillus vaginalis | China | Asia | Lactobacillaceae | Probiotic |
| GCF_003833155.1 | Limosilactobacillus vaginalis | Italy | Europe | Lactobacillaceae | Probiotic |
| GCF_027156105.1 | Limosilactobacillus vaginalis | USA | North America | Lactobacillaceae | Probiotic |
| GCF_027156125.1 | Limosilactobacillus vaginalis | USA | North America | Lactobacillaceae | Probiotic |
| GCF_027156165.1 | Limosilactobacillus vaginalis | USA | North America | Lactobacillaceae | Probiotic |
| GCF_027156215.1 | Limosilactobacillus vaginalis | USA | North America | Lactobacillaceae | Probiotic |
| GCF_027156205.1 | Limosilactobacillus vaginalis | USA | North America | Lactobacillaceae | Probiotic |
| GCF_027156305.1 | Limosilactobacillus vaginalis | USA | North America | Lactobacillaceae | Probiotic |
| GCF_027154405.1 | Limosilactobacillus vaginalis | USA | North America | Lactobacillaceae | Probiotic |
| GCF_027156545.1 | Limosilactobacillus vaginalis | USA | North America | Lactobacillaceae | Probiotic |
| GCF_027156605.1 | Limosilactobacillus vaginalis | USA | North America | Lactobacillaceae | Probiotic |
| GCF_027156755.1 | Limosilactobacillus vaginalis | USA | North America | Lactobacillaceae | Probiotic |
| GCF_027156625.1 | Limosilactobacillus vaginalis | USA | North America | Lactobacillaceae | Probiotic |
| GCF_027156665.1 | Limosilactobacillus vaginalis | USA | North America | Lactobacillaceae | Probiotic |
| GCF_027156685.1 | Limosilactobacillus vaginalis | USA | North America | Lactobacillaceae | Probiotic |
| GCF_025311515.1 | Limosilactobacillus vaginalis | USA | North America | Lactobacillaceae | Probiotic |
| GCF_027682185.1 | Limosilactobacillus vaginalis | China | Asia | Lactobacillaceae | Probiotic |
| GCF_030371355.1 | Limosilactobacillus vaginalis | Czech Republic | Europe | Lactobacillaceae | Probiotic |
| GCF_030371915.1 | Limosilactobacillus vaginalis | Czech Republic | Europe | Lactobacillaceae | Probiotic |
| GCF_030372015.1 | Limosilactobacillus vaginalis | Czech Republic | Europe | Lactobacillaceae | Probiotic |
| GCF_030371875.1 | Limosilactobacillus vaginalis | Czech Republic | Europe | Lactobacillaceae | Probiotic |
| GCF_030371965.1 | Limosilactobacillus vaginalis | Czech Republic | Europe | Lactobacillaceae | Probiotic |
| GCF_030373865.1 | Limosilactobacillus vaginalis | Czech Republic | Europe | Lactobacillaceae | Probiotic |
| GCF_022456495.1 | Limosilactobacillus vaginalis | South Africa | Africa | Lactobacillaceae | Probiotic |
| GCF_022456515.1 | Limosilactobacillus vaginalis | South Africa | Africa | Lactobacillaceae | Probiotic |
| GCF_944325995.1 | Limosilactobacillus vaginalis | France | Europe | Lactobacillaceae | Probiotic |
| GCF_947381825.1 | Limosilactobacillus vaginalis | Germany | Europe | Lactobacillaceae | Probiotic |
| GCF_947381785.1 | Limosilactobacillus vaginalis | Germany | Europe | Lactobacillaceae | Probiotic |
| GCF_958412415.1 | Limosilactobacillus vaginalis | Kenya | Africa | Lactobacillaceae | Probiotic |
| GCF_963510615.1 | Limosilactobacillus vaginalis | USA | North America | Lactobacillaceae | Probiotic |
| GCF_925282335.1 | Limosilactobacillus vaginalis | Slovenia | Europe | Lactobacillaceae | Probiotic |
| GCF_904382385.1 | Limosilactobacillus vaginalis | United Kingdom | Europe | Lactobacillaceae | Probiotic |
| GCF_022775235.1 | Limosilactobacillus vaginalis | Canada | North America | Lactobacillaceae | Probiotic |
| GCF_022756045.1 | Limosilactobacillus vaginalis | USA | North America | Lactobacillaceae | Probiotic |
| GCF_020743255.1 | Limosilactobacillus vaginalis | China | Asia | Lactobacillaceae | Probiotic |
| GCF_000159435.1 | Limosilactobacillus vaginalis | Switzerland | Europe | Lactobacillaceae | Probiotic |

**Supplementary Table 1C. RefSeq *Lactobacillaceae* sources**

| Accession_number | Species | Host | Host_site | Country | UN Region | strain |
| --- | --- | --- | --- | --- | --- | --- |
| GCF_018458775.1 | Lacticaseibacillus rhamnosus | Homo sapiens | NA | USA | North America | strain: RAB2019A |
| GCF_020826335.1 | Lacticaseibacillus rhamnosus | Homo sapiens | NA | South Korea | Asia | strain: PMC203 |
| GCF_001656575.1 | Lacticaseibacillus rhamnosus | Homo sapiens | NA | Missing | Europe | strain: Lrh31 |
| GCF_033977045.1 | Lacticaseibacillus rhamnosus | Homo sapiens | NA | South Korea | Asia | strain: LR6 |
| GCF_002238035.1 | Lacticaseibacillus rhamnosus | Homo sapiens | NA | Argentina | South America | strain: IBL027 |
| GCF_900070175.1 | Lacticaseibacillus rhamnosus | NA | NA | Missing | Europe | strain: BPL5 |
| GCF_000699985.1 | Lacticaseibacillus rhamnosus | Homo sapiens | NA | Russia | Europe | strain: 51B |
| GCF_024391105.1 | Lactiplantibacillus plantarum | Homo sapiens | NA | China | Asia | strain: YD2 |
| GCF_024391065.1 | Lactiplantibacillus plantarum | Homo sapiens | NA | China | Asia | strain: YD1 |
| GCF_033024555.1 | Lactiplantibacillus plantarum | NA | NA | Brazil | South America | strain: MDBL 269 |
| GCF_000762955.1 | Lactiplantibacillus plantarum | Homo sapiens | NA | Belgium | Europe | strain: CMPG5300 |
| GCF_001541585.1 | Lactobacillus crispatus | Homo sapiens | NA | USA | North America | strain: VMC8 |
| GCF_001541535.1 | Lactobacillus crispatus | Homo sapiens | NA | USA | North America | strain: VMC7 |
| GCF_001541505.1 | Lactobacillus crispatus | Homo sapiens | NA | USA | North America | strain: VMC6 |
| GCF_001541515.1 | Lactobacillus crispatus | Homo sapiens | NA | USA | North America | strain: VMC5 |
| GCF_001541405.1 | Lactobacillus crispatus | Homo sapiens | NA | USA | North America | strain: VMC4 |
| GCF_001541385.1 | Lactobacillus crispatus | Homo sapiens | NA | USA | North America | strain: VMC3 |
| GCF_001546025.1 | Lactobacillus crispatus | Homo sapiens | NA | USA | North America | strain: VMC2 |
| GCF_001546015.1 | Lactobacillus crispatus | Homo sapiens | NA | USA | North America | strain: VMC1 |
| GCF_011029265.1 | Lactobacillus crispatus | Homo sapiens | NA | Thailand | Asia | strain: VA50-4AN |
| GCF_004681235.1 | Lactobacillus crispatus | Homo sapiens | NA | France | Europe | strain: V4 |
| GCF_019537355.1 | Lactobacillus crispatus | Homo sapiens | NA | India | Asia | strain: UBLCp-01 |
| GCF_018885325.1 | Lactobacillus crispatus | NA | NA | South Korea | Asia | strain: PMC201 |
| GCF_027271175.1 | Lactobacillus crispatus | Homo sapiens | NA | China | Asia | strain: Lcr-MH175 |
| GCF_021278925.1 | Lactobacillus crispatus | Homo sapiens | NA | China | Asia | strain: lc83 |
| GCF_021278945.1 | Lactobacillus crispatus | Homo sapiens | NA | China | Asia | strain: lc31 |
| GCF_016162065.1 | Lactobacillus crispatus | Homo sapiens | NA | Italy | Europe | strain: LB63 |
| GCF_016162125.1 | Lactobacillus crispatus | Homo sapiens | NA | Italy | Europe | strain: LB62 |
| GCF_016162155.1 | Lactobacillus crispatus | Homo sapiens | NA | Italy | Europe | strain: LB61 |
| GCF_016162165.1 | Lactobacillus crispatus | Homo sapiens | NA | Italy | Europe | strain: LB59 |
| GCF_016162145.1 | Lactobacillus crispatus | Homo sapiens | NA | Italy | Europe | strain: LB58 |
| GCF_016162185.1 | Lactobacillus crispatus | Homo sapiens | NA | Italy | Europe | strain: LB57 |
| GCF_016162195.1 | Lactobacillus crispatus | Homo sapiens | NA | Italy | Europe | strain: LB56 |
| GCF_004103355.1 | Lactobacillus crispatus | Homo sapiens | NA | China | Asia | strain: L49 |
| GCF_009730275.1 | Lactobacillus crispatus | Homo sapiens | NA | USA | North America | strain: FDAARGOS_743 |
| GCF_015708075.1 | Lactobacillus crispatus | Homo sapiens | NA | Brazil | South America | strain: CRI8 |
| GCF_015708105.1 | Lactobacillus crispatus | Homo sapiens | NA | Brazil | South America | strain: CRI4 |
| GCF_015708055.1 | Lactobacillus crispatus | Homo sapiens | NA | Brazil | South America | strain: CRI17 |
| GCF_015708065.1 | Lactobacillus crispatus | Homo sapiens | NA | Brazil | South America | strain: CRI10 |
| GCF_003795065.1 | Lactobacillus crispatus | Homo sapiens | NA | USA | North America | strain: CO3MRSI1 |
| GCF_008079315.1 | Lactobacillus crispatus | Homo sapiens | NA | France | Europe | strain: CIP 104459 |
| GCF_016093195.1 | Lactobacillus crispatus | Homo sapiens | NA | USA | North America | strain: C0176A1 |
| GCF_008868575.1 | Lactobacillus crispatus | Homo sapiens | NA | Missing | Missing | strain: BIO6272 |
| GCF_014654865.1 | Lactobacillus crispatus | Homo sapiens | NA | Italy | Europe | strain: BC5 |
| GCF_014654855.1 | Lactobacillus gasseri | Homo sapiens | NA | Italy | Europe | strain: BC12 |
| GCF_000439915.2 | Lactobacillus gasseri | Homo sapiens | NA | Russia | Europe | strain: 2016 |
| GCF_011029225.1 | Lactobacillus jensenii | Homo sapiens | NA | Thailand | Asia | strain: VA04-2AN |
| GCF_019459545.1 | Lactobacillus jensenii | Homo sapiens | NA | India | Asia | strain: UBLJe-01 |
| GCF_001936235.1 | Lactobacillus jensenii | Homo sapiens | NA | South Korea | Asia | strain: SNUV360 |
| GCF_019459625.1 | Lactobacillus johnsonii | Homo sapiens | NA | India | Asia | strain: UBLJ-01 |
| GCF_011029235.1 | Ligilactobacillus salivarius | Homo sapiens | NA | Thailand | Asia | strain: VA40-10 |
| GCF_026935945.1 | Limosilactobacillus vaginalis | Homo sapiens | NA | Brazil | South America | strain: VAG1 |
| GCF_009362935.1 | Limosilactobacillus vaginalis | NA | NA | Korea | Asia | strain: LV515 |

**Supplementary Table 1D. RefSeq *Prevotella* spp. sources**

| Accession_number | Species | Host | Country | Region | strain | Type_strain |
| --- | --- | --- | --- | --- | --- | --- |
| GCF_946997305.1 | Prevotella disiens | NA | USA | North America | NA | NA |
| GCF_000262545.1 | Prevotella bivia | Homo sapiens | USA | North America | strain: DSM 20514 | type strain of Prevotella bivia |
| GCF_000467875.1 | Prevotella disiens | Homo sapiens | USA | North America | strain: ATCC 29426 | type strain of Prevotella disiens |
| GCF_000759045.1 | Prevotella bivia | Homo sapiens | USA | North America | strain: DNF00188 | NA |
| GCF_000759315.1 | Prevotella amnii | Homo sapiens | USA | North America | strain: DNF00058 | NA |
| GCF_000759245.1 | Prevotella bivia | Homo sapiens | Missing | Missing | strain: DNF00320 | NA |
| GCF_000759165.1 | Prevotella bivia | Homo sapiens | USA | North America | strain: DNF00650 | NA |
| GCF_000759305.1 | Prevotella melaninogenica | Homo sapiens | Missing | Missing | strain: DNF00666 | NA |
| GCF_000759225.1 | Prevotella disiens | Homo sapiens | USA | North America | strain: DNF00882 | NA |
| GCF_001546565.2 | Prevotella bivia | Homo sapiens | USA | North America | strain: GED7760C | NA |
| GCF_001574405.1 | Prevotella bivia | Homo sapiens | USA | North America | strain: GED7880 | NA |
| GCF_001553225.1 | Prevotella amnii | Homo sapiens | Missing | Missing | strain: DNF00307 | NA |
| GCF_946998675.1 | Hoylesella timonensis | NA | USA | North America | NA | NA |
| GCF_947041285.1 | Hoylesella timonensis | NA | Denmark | Europe | NA | NA |
| GCF_000762405.1 | Hoylesella timonensis | Homo sapiens | USA | North America | strain: S9-PR14 | NA |
| GCF_002894165.1 | Hoylesella timonensis | Homo sapiens | USA | North America | strain: DNF00076 | NA |

**Supplementary Table 1E. RefSeq *Gardnerella* spp. sources**

| Accession_number | Species | Host | Country | Region | strain | Type_strain |
| --- | --- | --- | --- | --- | --- | --- |
| GCF_001042655.1 | Gardnerella vaginalis | Human (Female) | USA | North America | strain: JCM 11026 | type strain of Gardnerella vaginalis |
| GCF_000414625.1 | Gardnerella pickettii | Homo sapiens | USA | North America | strain: JCP7719 | NA |
| GCF_000414585.1 | Gardnerella pickettii | Homo sapiens | USA | North America | strain: JCP8017B | NA |
| GCF_000414445.1 | Gardnerella vaginalis | Homo sapiens | USA | North America | strain: JCP8481B | NA |
| GCF_000414525.1 | Gardnerella vaginalis | Homo sapiens | USA | North America | strain: JCP8108 | NA |
| GCF_000414645.1 | Gardnerella vaginalis | Homo sapiens | USA | North America | strain: JCP7672 | NA |
| GCF_000414705.1 | Gardnerella vaginalis | Homo sapiens | USA | North America | strain: JCP7275 | NA |
| GCF_000414685.1 | Gardnerella vaginalis | Homo sapiens | USA | North America | strain: JCP7276 | NA |
| GCF_000414465.1 | Gardnerella vaginalis | Homo sapiens | USA | North America | strain: JCP8481A | NA |
| GCF_000414605.1 | Gardnerella pickettii | Homo sapiens | USA | North America | strain: JCP8017A | NA |
| GCF_003369965.1 | Gardnerella vaginalis | NA | Kenya | Africa | strain: N95 | NA |
| GCF_003369895.1 | Gardnerella vaginalis | NA | Kenya | Africa | strain: N101 | NA |
| GCF_003369935.1 | Gardnerella vaginalis | NA | Kenya | Africa | strain: N153 | NA |
| GCF_003369875.1 | Gardnerella vaginalis | NA | Canada | North America | strain: W11 | NA |
| GCF_001563665.1 | Gardnerella vaginalis | Homo sapiens | Missing | Missing | strain: CMW7778B | NA |
| GCF_001546445.1 | Gardnerella pickettii | Homo sapiens | USA | North America | strain: GED7275B | NA |
| GCF_001546455.1 | Gardnerella vaginalis | Homo sapiens | USA | North America | strain: GED7760B | NA |
| GCF_002896555.1 | Gardnerella vaginalis | Homo sapiens | USA | North America | strain: KA00225 | NA |
| GCF_002894085.1 | Gardnerella sp. KA00735 | Homo sapiens | USA | North America | strain: KA00735 | NA |
| GCF_002894105.1 | Gardnerella vaginalis | Homo sapiens | USA | North America | strain: DNF01149 | NA |
| GCF_002894125.1 | Gardnerella sp. DNF01162 | Homo sapiens | USA | North America | strain: DNF01162 | NA |
| GCF_003426405.1 | Gardnerella piotii | Homo sapiens | Belgium | Europe | strain: GH007 | NA |
| GCF_003426385.1 | Gardnerella piotii | Homo sapiens | Belgium | Europe | strain: GH020 | NA |
| GCF_003585655.1 | Gardnerella vaginalis | Homo sapiens | Kenya | Africa | strain: NR038 | NA |
| GCF_003585755.1 | Gardnerella vaginalis | Homo sapiens | Kenya | Africa | strain: NR039 | NA |
| GCF_003397705.1 | Gardnerella swidsinskii | Homo sapiens | Russia | Europe | strain: GS 9838-1 | type strain of Gardnerella swidsinskii |
| GCF_003397685.1 | Gardnerella vaginalis | Homo sapiens | USA | North America | strain: ATCC 14018 | type strain of Gardnerella vaginalis |
| GCF_003293675.1 | Gardnerella leopoldii | Homo sapiens | Belgium | Europe | strain: UGent 06.41 | type strain of Gardnerella leopoldii |
| GCF_003397585.1 | Gardnerella piotii | Homo sapiens | Belgium | Europe | strain: UGent 18.01 | type strain of Gardnerella piotii |
| GCF_003397605.1 | Gardnerella vaginalis | Homo sapiens | Belgium | Europe | strain: UGent 25.49 | NA |
| GCF_014857145.1 | Gardnerella vaginalis | Homo sapiens | Russia | Europe | strain: 06-12-0010 | NA |
| GCF_023277725.1 | Gardnerella vaginalis | Homo-sapiens | China | Asia | strain: JNFY1 | NA |
| GCF_023277705.1 | Gardnerella swidsinskii | Homo-sapiens | China | Asia | strain: JNFY3 | NA |
| GCF_023277685.1 | Gardnerella vaginalis | Homo-sapiens | China | Asia | strain: JNFY4 | NA |
| GCF_023277665.1 | Gardnerella vaginalis | Homo-sapiens | China | Asia | strain: JNFY9 | NA |
| GCF_023277645.1 | Gardnerella vaginalis | Homo-sapiens | China | Asia | strain: JNFY11 | NA |
| GCF_023277625.1 | Gardnerella vaginalis | Homo-sapiens | China | Asia | strain: JNFY13 | NA |
| GCF_023277605.1 | Gardnerella vaginalis | Homo-sapiens | China | Asia | strain: JNFY14 | NA |
| GCF_023277585.1 | Gardnerella piotii | Homo-sapiens | China | Asia | strain: JNFY15 | NA |
| GCF_023277565.1 | Gardnerella vaginalis | Homo-sapiens | China | Asia | strain: JNFY17 | NA |
| GCF_023016205.1 | Gardnerella vaginalis | Homo sapiens | UK | Europe | strain: KC1 | NA |
| GCF_023016185.1 | Gardnerella vaginalis | Homo sapiens | UK | Europe | strain: KC2 | NA |
| GCF_023016245.1 | Gardnerella vaginalis | Homo sapiens | UK | Europe | strain: KC3 | NA |
| GCF_023016225.1 | Gardnerella vaginalis | Homo sapiens | UK | Europe | strain: KC4 | NA |

**Supplementary Table 1F. RefSeq *Fannyhessea vaginae* sources**

| Accession_number | Species | Host | Country | UN region | strain | Type_strain |
| --- | --- | --- | --- | --- | --- | --- |
| GCF_900445305.1 | Fannyhessea vaginae | Homo sapiens | Sweden | Europe | strain: NCTC13935 | type strain of Atopobium vaginae |
| GCF_001049775.1 | Fannyhessea vaginae | Homo sapiens | Sweden | Europe | strain: 44061 | NA |
| GCF_019400185.1 | Fannyhessea vaginae | Homo sapiens | China | Asia | isolate: C17-1 | NA |
| GCF_019400195.1 | Fannyhessea vaginae | Homo sapiens | China | Asia | isolate: C17-2 | NA |
| GCF_019400135.1 | Fannyhessea vaginae | Homo sapiens | China | Asia | isolate: C17-4 | NA |
| GCF_019400085.1 | Fannyhessea vaginae | Homo sapiens | China | Asia | isolate: C17-5 | NA |
| GCF_019400095.1 | Fannyhessea vaginae | Homo sapiens | China | Asia | isolate: C17-7 | NA |
